# Neuroprotective Effects of Oligosaccharides From *Periplaneta Americana* on Parkinson’s Disease Models *In Vitro* and *In Vivo*

**DOI:** 10.3389/fphar.2022.936818

**Published:** 2022-07-18

**Authors:** Miao-Miao Liu, Nan Zhou, Na Jiang, Kai-Min Lu, Chuan-Fang Wu, Jin-Ku Bao

**Affiliations:** ^1^ Key Laboratory of Bio-Resource and Eco-Environment of Ministry of Education, College of Life Sciences, Sichuan University, Chengdu, China; ^2^ Pharmacy Research Center, Binzhou Medical University, Yantai, China

**Keywords:** Parkinson’s disease, *Periplaneta Americana*, oligosaccharides, apoptosis, neuroprotective effects

## Abstract

Parkinson’s disease (PD) is one of the neurodegenerative diseases that is characterized by obvious motor and some nonmotor symptoms. Various therapeutics failed in the effective treatment of PD because of impaired neurological function in the brain and various complications. *Periplaneta Americana* oligosaccharides (OPA), the main active ingredients extracted from the medicine residues of *Periplaneta Americana* (*P. Americana*), have been reported to exert anti-inflammatory effects. The purpose of this study was to evaluate the possible mechanisms of OPA against 1-methyl-4-phenylpyridinium (MPP^+^)-induced apotosis in SH-SY5Y cells and its potential neuroprotective effects in 1-methyl-4-phenyl-1,2,3,6-tetrahydropyridine (MPTP)-induced PD subacute model mice. The data demonstrated that OPA significantly reversed the MPP^+^-induced decrease in SH-SY5Y cell viability, reduced the proportion of apoptotic cells, and protected SH-SY5Y cells from apoptosis in a dose-dependent manner by regulating the expression of apoptosis-related genes. Furthermore, OPA also alleviated the motor dysfunction of PD model mice, prevented the loss of tyrosine hydroxylase positive cells, suppressed the apoptosis of substantia nigra cells, and improved the dysbiosis of gut microbiota *in vivo*, suggesting that OPA demonstrated a significantly neuroprotective effect on PD model mice. These results indicated that OPA might be the possibility of PD therapeutics with economic utility and high safety.

## 1 Introduction

Parkinson’s disease (PD), a progressive neurological disorder characterized by tremor, rigidity, bradykinesia, and postural instability, is mainly featured by pathological irreversible loss of dopaminergic (DAergic) neurons in the substantia nigra pars compacta (SNpc) and affects 2–3% of the population over 65 years of age (Gao et al., 2014). In addition to movement disorder, PD patients might also experience nonmotor symptoms, including depression, cognitive impairment, sleep disorders, hyposmia, and, most generally, gastrointestinal dysfunction (Aubignat et al., 2020; Auger et al., 2020; Mozaffari et al., 2020; Kumaresan and Khan, 2021; Roy et al., 2021). Although the trigger for PD remains limited knowledge, several biochemical mechanisms related to the pathogenesis of PD, which is believed that both endogenous and exogenous factors are involved, including oxidative stress, mitochondrial dysfunction, protein aggregation and misfolding, apoptosis, excitotoxicity, and neuroinflammation, are beginning to be understood. For example, as a key form of programmed cell death, the apoptosis of midbrain DAergic neurons could lead to a significant decrease in striatal dopamine (DA), which subsequently promoted the pathogenesis of PD (Li et al., 2022). At present, PD therapeutics mostly advance symptom management rather than blocking disease progression (Wang et al., 2009). As the commonly used medicines, traditional anticholinergic drugs, such as trihexyphenidyl hydrochloride, amantadine, and levodopa preparations, have been found to exert serious side effects. Among them, the discomfort caused by the patient’s gastrointestinal tract is the most common, namely indigestion, vomiting, and constipation (Sheu et al., 2019; Nawaz et al., 2022). Until now, none of therapies have been convincingly shown to slow down or prevent the progression of PD. Therefore, searching for novel therapeutic agents with higher effectiveness and less side effects is urgently needed (Kuang et al., 2017).

Traditional Chinese medicine, an important part of the healthcare system with a long history in China, have been reported to possess good efficacy and high safety in the treatment of chronic diseases with cost-effective benefits (Lee et al., 2014). Clinical studies showed that in the progression of PD, both Chinese herbal compounds/extracts and herb formulas, as the adjunct, could improve motor and nonmotor symptoms and even reduce dose of DAergic drugs and occurrence of dyskinesia (Zeng, 2017). For instance, in MPP^+^- and MPTP-induced PD models, Kukoamine A, a main bioactive ingredient in Cortex Lycii Radicis, presented significantly neuroprotective effects by inhibiting oxidative stress, reducing toxic EAA, and suppressing neuronal apoptosis (Hu et al., 2018). Furthermore, the medicinal insects, whose species are more than twice as many as medical plants (Ma et al., 2018), also received widespread concern in the procession of PD. Batryticatus Bombyx have been reported to protect DAergic neurons against MPTP-induced neurotoxicity by alleviating oxidative damage and improving behavioral impairments in PD model mice (Lim et al., 2019). Up to now, a series of studies demonstrated that polysaccharides or oligosaccharides with bioactivities from plants, animals, and fungi exerted promising therapeutic effects for preventing neurodegenerative disorders mainly by the microbiota-gut-brain axis in MPTP-induced PD model, such as Astragalus polysaccharides (Li et al., 2016; Souza et al., 2017), fucoidan (Kim et al., 2017), and oligomannurarate 971 (Liu et al., 2020a). Growing evidence implicated that dysbiosis in the abundance and composition of gut microbiota could affect both the central nervous system (CNS) and the enteric nervous system (ENS) (Liu et al., 2022). Experimental and clinical results indicated the existence of dysbiosis of gut microbiota and microbial metabolites in various CNS diseases. In Alzheimer’s disease (AD), the decreased gut microbial diversity was observed, compared with control sex- and age-matched individuals, in which increased Bacteroidetes and decreased Firmicutes and Bifidobacterium were obviously perceived. Also, certain species of decreased Bifidobacterium were related with anti-inflammatory properties and reduced intestinal permeability in AD (Vogt et al., 2017). Besides, gut dysbiosis associated with motor deficits and body weight impairment has recently been reported in Huntington’s disease (HD) transgenic mice, in which an increase in Bacteroidetes and a proportional decrease in Firmicutes were found (Kong et al., 2018; Wasser et al., 2020). In HD patients, the abundance of the genus Intestinimonas was observed to be higher than that of healthy controls, which was reported to show a key role in anti-inflammation (Cai et al., 2020; Zhuang et al., 2020). Furthermore, gut microbial dysbiosis not only existed in PD patients, but related to many PD animal models. Among PD patients, a high prevalence of Helicobacter pylori (H. Pylori) infection was reported many years ago (Charlett et al., 1999). In the meantime, bacteria more generally correlative with anti-inflammatory properties, such as genus, Coprococcus, Blautia, and Roseburia, were markedly reduced in fecal samples of PD patients, along with an increase in the genus Ralstonia and a decrease in the genus Faecalibacterium in the mucosa of PD subjects, which potentially transformed the microbial balance within the colon to a more inflammatory phenotype (Keshavarzian et al., 2015). *Periplaneta Americana* (*P. Americana*), commonly known as the cockroach, has been a noted traditional Chinese medicinal material for thousands of years (Ma et al., 2018). Studies showed that the *P. Americana*, which is widely distributed in tropical areas, contains a variety of small molecule peptides and amino acids, polysaccharides, oligosaccharides, clusterins, pheromones, and other components, which can promote tissue repair, enhance myocardial contractility, protect liver and resist damage, and improve immunity (Sheu et al., 2019; Nawaz et al., 2022). According to the results of preliminary research in the laboratory (Lu et al., 2021), researchers verified that the oligosaccharides extracted from the dregs of *P. Americana* (OPA), which were composed of 83% glucose, 11% xylose and 6% galactose, could effectively improve enteritis of model mice and demonstrated certain antioxidant activity. However, how OPA regulates the compositions of intestinal flora and what role OPA plays in PD is unclear.

Based on these findings, the present study was designed to verify the potential neuroprotective effects of OPA and the underlying mechanisms with a focus on apoptosis both in SH-SY5Y cells and in PD model mice. To test this hypothesis, we first investigated the effects of OPA on the percentage of apoptotic SH-SY5Y cells and the activity of apoptosis-related genes expression. Next, an in-depth exploration about the biological functions and regulatory mechanisms of OPA was used to illustrate the effects on motor functions, tyrosine hydroxylase (TH) expression, and dopaminergic neurons viability in PD model mice. In addition, we examined the alteration of gut microbial compositions at the family and genus level to disclose the role of OPA in the microbiome of the PD mice. These studies may not only provide a new way for developing effective drugs for PD treatment, but also pave the basis for further analyses of OPA in the future.

## 2 Materials and Methods

### 2.1 Materials and Chemicals

The *Periplaneta Americana* residues were collected from the Sichuan Gooddoctor-Panxi Phar-maceutical Company (Xichang, Sichuan Province, China). The purification of OPA has been performed according to the method previously described in our laboratory (Lu et al., 2021).

### 2.2 Animals

Male C57BL/6J mice (eight-weeks-old), purchased from Chengdu Dashuo Company in Chengdu (Sichuan Province, China), were housed for at least a week at 25°C under 12 h light/dark cycles with access to water and pellet food ahead of formal experiments. All animal experiments were preformed according to the Animal Care and Use Committee of China guidelines.

### 2.3 SH-SY5Y Cell Culture and Treatment

SH-SY5Y cells were cultured in DMEM medium containing 10% heat-inactivated horse serum, 5% heat-inactivated FCS, 100 IU/ml penicillin, and 100 μg/ml streptomycin. The cultures were maintained with 5% CO_2_ humidified-airatmosphere at 37°C. Well-cultivated SH-SY5Y cells were divided into several groups according to treatments of different concentrations of MPP^+^ (Sigma, United States) or OPA. A normal group was left with treatment of neither MPP^+^ nor OPA.

### 2.4 Cell Viability

Cell viability was measured by cell count kit 8 (CCK-8). After seeded into 96-well plates, well-cultivated SH-SY5Y cells were treated with various concentrations of MPP^+^ (0, 0.05, 0.1, 0.25, 0.5, 1, and 2 mM) for 24 h to investigate the neurotoxicity of MPP^+^. Meanwhile, the toxicity tests of OPA to SH-SY5Y cells were performed in the control group and oligosaccharide groups (50 μg/ml, 100 μg/ml, 500 μg/ml, 1000 μg/ml, and 1500 μg/ml). To further explore the protective concentration of OPA on MPP^+^-induced PD model, SH-SY5Y cells were pretreated with OPA (0 μg/ml, 50 μg/ml, 100 μg/ml, and 500 μg/ml) for 24 h and then incubated with 0.5 mM MPP^+^ for an additional 24 h. Among them, control cells were treated in the same way without OPA or MPP^+^. The absorbance was determined at 450 nm using a microplate reader (Thermo Fisher Scientific, United States), and the ratio of viability of control groups was considered as 100%.

### 2.5 Morphological Changes

SH-SY5Y cells were seeded in six-well plates with approximately 1 × 10^5^ cells per well. After being completely attached, SH-SY5Y cells were divided into control group and experimental groups, which were pretreated by different concentrations of OPA (0 μg/ml, 50 μg/ml, 100 μg/ml, and 500 μg/ml) for 24 h prior to treatment with 0.5 mM MPP^+^ for the other 24 h. Next, SH-SY5Y cells were fixed with 4% paraformaldehyde (PFA) at room temperature for 30 min and then washed three times with PBS. After incubation by a Hoechst 33342 staining solution (Beyotime, China) for 3–5 min at room temperature, PBS-rinsed SH-SY5Y cells were observed under the fluorescence microscope. Compared to normal cells, the apoptotic cells exhibited brighter fluorescence and densely stained nuclei.

### 2.6 Flow Cytometric Analysis

Apoptotic and necrotic cells were determined by using annexin V-FITC apoptosis detection kit (BD Biosciences, United States). In brief, SH-SY5Y cells were treated with MPP^+^ or OPA, followed by centrifugation at 700 g for 5 min and resuspending in 100 μL binding buffer. Then, SH-SY5Y cells were coincubated with 5 μL annexin V and 5 μL PI for 15 min at room temperature in the dark. Last, 400 μL 1 × annexin V Binding Buffer was added for fully mixing before tests.

### 2.7 Establishment of PD Mice Model

Eight-weeks-old healthy C57BL/6J male mice were chosen to give OPA (once a day, from day-1 to the final day, continuously 14 days) or the same amount of a standard suspension vehicle (0.9% NaCl, w/v) as the control group, after 1 h granted MPTP (30 mg/kg, once a day, from day-8 to day-14, continuously 7 days) or the same amount of saline subcutaneously to establish a MPTP subacute PD mice inflammation model. After that, to explore the effects of MPTP on the dietary intake or food digestion, the mice were weighed at ten o’clock every morning. On day-15, behavioral tests were conducted on all the groups. Then, all mice were sacrificed quickly by euthanasia, and the serum was collected for evaluating cytokines. Some mice of each group were perfused intracardially with saline (0.9%) followed by 4% PFA and brains collection. Meantime, ventral midbrain, striatum, and stool in the intestine were dissected rapidly and stored at −80°C until used, while the spleen and thymus were weighed and recorded.

### 2.8 Behavioral Tests

#### 2.8.1 Climbing Pole Tests

The experiment is performed to test the coordination of the mice’s limb movement. In brief, mice were placed head-down on the top of the 50 cm long pole, and their descent back into the home cage was timed. The time was recorded from the experimenter that released the animal until one hind-limb reached the cage base.

#### 2.8.2 Suspension Time Tests

The mouse’s forelimbs were suspended on a metal wire with a horizontal height of 100 cm and observed for 2 min, while the limbs placements were scored from 0 to 4, with the lowest score indicating the most severe deficit. Animals were assigned a score of 4 for gripping the wire with both hind paws, 3 for gripping the wire with one hind paw, 2 for gripping the wire with both front paws, 1 for gripping the wire with one front paw, and 0 for mouse falling. The test was performed three times for each animal, and the average was taken.

### 2.9 Immunohistochemical Analysis

Immunohistochemistry was taken for TH expression in substantia nigra of PD mice model. After perfusion from the left ventricle, the brains of mice were rapidly removed and placed in 4% PFA for overnight fixation. Sucrose solution was used for gradient dehydration, and OCT gel was used for embedding. Next, we prepared brain tissue sections (30 microns) with a frozen slicer and selected the brain slices of the substantia nigra according to the mouse brain atlas. TH-positive neurons and GFAP positive astrocytes were quantified using Image-Pro Express 6 (Media Cybernetics), and the mean number of them in each brain was picked up. Olympus camera (DP72, Olympus) was used to take images at an original magnification of 100×.

### 2.10 TUNEL Staining

TUNEL staining was performed using the *In Situ* cell death detection kit (Roche, Swit) for the detection and quantification of apoptosis in substantia nigra cells of PD mice model. In brief, apoptotic cells were detected after being incubated by 3,3′-diaminobenzidine (DAB) chromogen (DAKO, United States), and slides were counter-stained with Hematoxylin solution (Solarbio, China).

### 2.11 Profiling of Intestinal Microorganism

The purification of gDNA in mice feces was carried out according to the instructions of ZymoBIOMICS™ DNA microprep kit (Zymo Research, United States), and then, the target bands were collected from samples qualified by PCR. Next, the hypervariable V4 region of 16S rRNA gene was amplified using barcoded primers (Forward primer, 5′-GTGYCAGCMGCCGCGGTAA-3′; Reverse primer, 5′-GGACT ACHVGGGTWTCTAAT-3′). After constructing the DNA library by next ultra II DNA library prep kit (NEB, United States) for Illumina, we performed cluster processing on the sequences to analyze the similarity of species, genera, and other information of the intestinal microorganism based on sequenced reads and operational taxonomic units.

### 2.12 Quantitative Real-Time PCR

RNA was extracted from MPP^+^- or OPA-treated cells using TRIzol™ Reagent (Invitrogen, United States) and then converted to cDNA by the PrimeScript™ RT reagent Kit with gDNA Eraser (TaKaRa, Japan). The levels of mRNA were measured by quantitative real-time PCR (qPCR) using the TB Green™ Premix Ex Taq™ II (TaKaRa, Japan). Primer sequences were shown in Table 1. Β-actin was amplified as the reference mRNA. Relative expression was calculated using the ΔC_T_ method.

**TABLE 1:** Primer sequences of qPCR.

Gene name	Primer sequnces
*BAX*	F: GAT​GCG​TCC​ACC​AAG​AAG​CTG​AG
	R: CAC​GGC​GGC​AAT​CAT​CCT​CTG
*BCL-2*	F: GTG​GAT​GAC​TGA​GTA​CCT​GAA​CCG
	R: AGA​GTC​TTC​AGA​GAC​AGC​CAG​GAG
*CASPASE3*	F: TGG​AAG​CGA​ATC​AAT​GGA​CTC​TGG
	R: CCA​GAC​CGA​GAT​GTC​ATT​CCA​GTG
*ACTB*	F: GAT​GAG​ATT​GGC​ATG​GCT​TT
	R: CAC​CTT​CAC​CGT​TCC​AGT​TT

### 2.13 Western Blot

After treated with MPP^+^ or OPA, collected SH-SY5Y cells were lysed by RIPA (Beyotime, China). The lysate was incubated on ice for 30 min and then centrifuged at 12,000 g for 5 min at 4°C. Meanwhile, the taken-out striatum of PD mice model was homogenized by adding an appropriate amount of RIPA according to the quality, and then, it was centrifuged at 1,000 g for 15 min at 4°C. Both supernatants were collected, and the concentration of total protein in supernatants was quantified using BCA protein assay kit (Beyotime, China). Next, the protein was subjected to 12% polyacrylamide gels and transferred onto a polyvinylidene fluoride (PVDF) (Millipore, United States). After blocking for 1 h with 5% non-fat milk at room temperature, the membrane was incubated with primary antibodies, including anti-Bax, anti-Bcl-2, anti-cleaved-caspase3, anti-β-actin (Cell Signalling Technology, United States), and anti-TH (Abcam, United Kingdom) overnight at 4°C. Following being washed by PBS/Tween-20 for three times, the membranes were incubated in the secondary antibody (1:2,000) at room temperature for 1 h. After being rinsed, bands were visualized using western blotting detection system (Bio-rad ChemiDoc Touch, United States) and quantified with NIH ImageJ software (National Institutes of Health, Bethesda, United States). β-actin was named as the internal control.

### 2.14 Statistical Analysis

All experiments were performed at least three times. Data were shown as mean ± SEM (standard error of mean). Depending on the total number of groups being analyzed, one way analysis of variance and Student *t* tests were performed to determine the *p* values. Differences in the mean values were considered to be significant at *p* < 0.05 and *p* < 0.01.

## 3 Results

### 3.1 OPA Rescued MPP^+^-Induced Death of SH-SY5Y Cells

**FIGURE 1:**
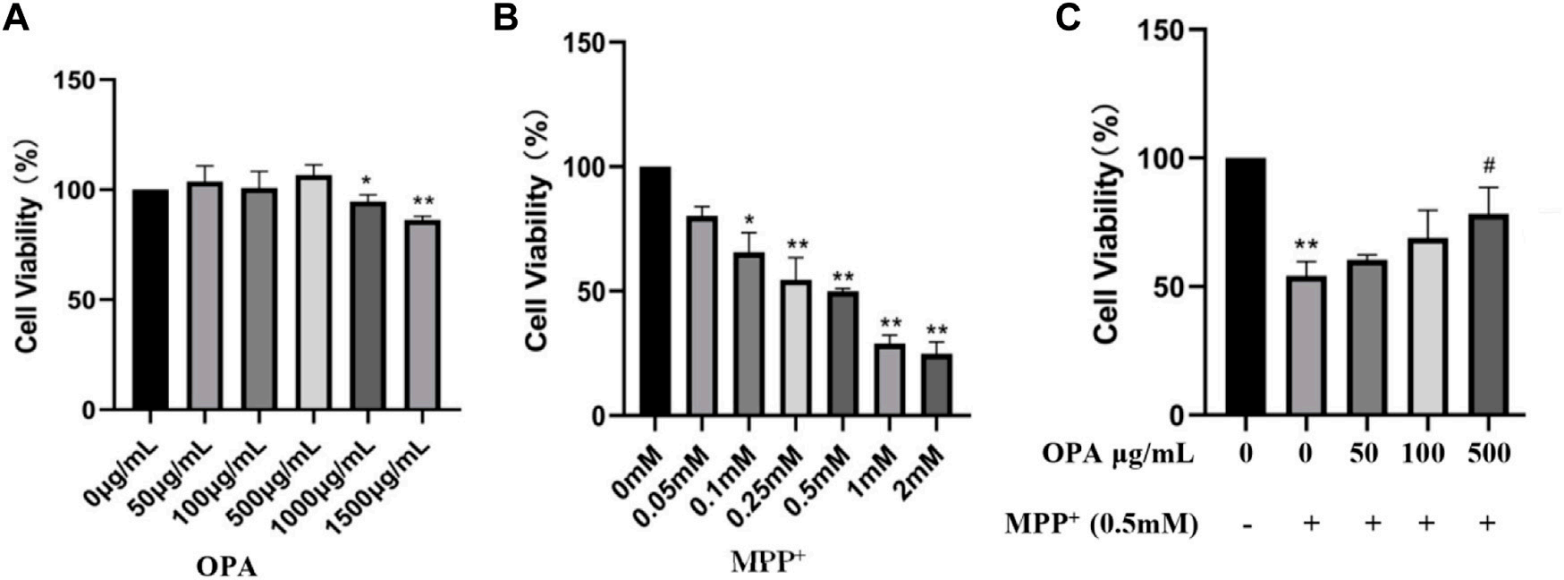
Effects of OPA on MPP^+^-induced death of SH-SY5Y cells. SH-SY5Y cells were incubated for 24 h with different concentrations of **(A)** OPA and **(B)** MPP^+^ alone. **(C)** SH-SY5Y cells were pretreated with different concentrations of OPA for 24 h and then exposed to MPP^+^ (0.5 mM) for an additional 24 h. The viability of SH-SY5Y cells that untreated with OPA and MPP^+^ was considered as control group. Data were presented as mean ± SD for three independent experiments. *p < 0.05, **p < 0.01 compared with control cells untreated with OPA and MPP^+^; ^#^ *p* < 0.05 compared with MPP^+^-treated cells.

To determine whether OPA make a difference to cell viability, SH-SY5Y cells were treated with various concentrations of OPA for 24 h, and then, CCK-8 assay was conducted to exclude the influence of OPA. As displayed in Figure 1A, when the concentrations of OPA was less than 1,000 μg/ml, the activity of SH-SY5Y cells was almost the same as that of the control group, while a slight decrease of cell viability occurred as the concentrations of OPA increased to 1,000 μg/ml and 1,500 μg/ml, suggesting OPA (< 1,000 μg/ml) itself demonstrated no significant effects on cell viability. However, after incubation with various concentrations of MPP^+^ for 24 h, a dose-dependent decrease occurred in viability of SH-SY5Y cells. Among them, the activity of SH-SY5Y cells incubated with 0.5 mM MPP^+^ for 24 h was reduced to 49.89%, and thus, this concentration was used in the following experiments (Figure 1B). Next, in order to observe the effects of OPA on activity of SH-SY5Y cells that suffered from MPP^+^-mediated death, SH-SY5Y cells were respectively pretreated with different concentrations of OPA for 24 h and then incubated with 0.5 mM MPP^+^ for another 24 h. Compared with SH-SY5Y cells treated with MPP^+^ only, the cell viability that pre-exposed to different concentrations of OPA (50–500 μg/ml) was obviously higher (Figure 1C). Together, these results discerned that OPA could effectively protect SH-SY5Y cells against MPP^+^-induced death.

### 3.2 OPA Relieved MPP^+^-Induced Apoptosis of SH-SY5Y Cells

**FIGURE 2:**
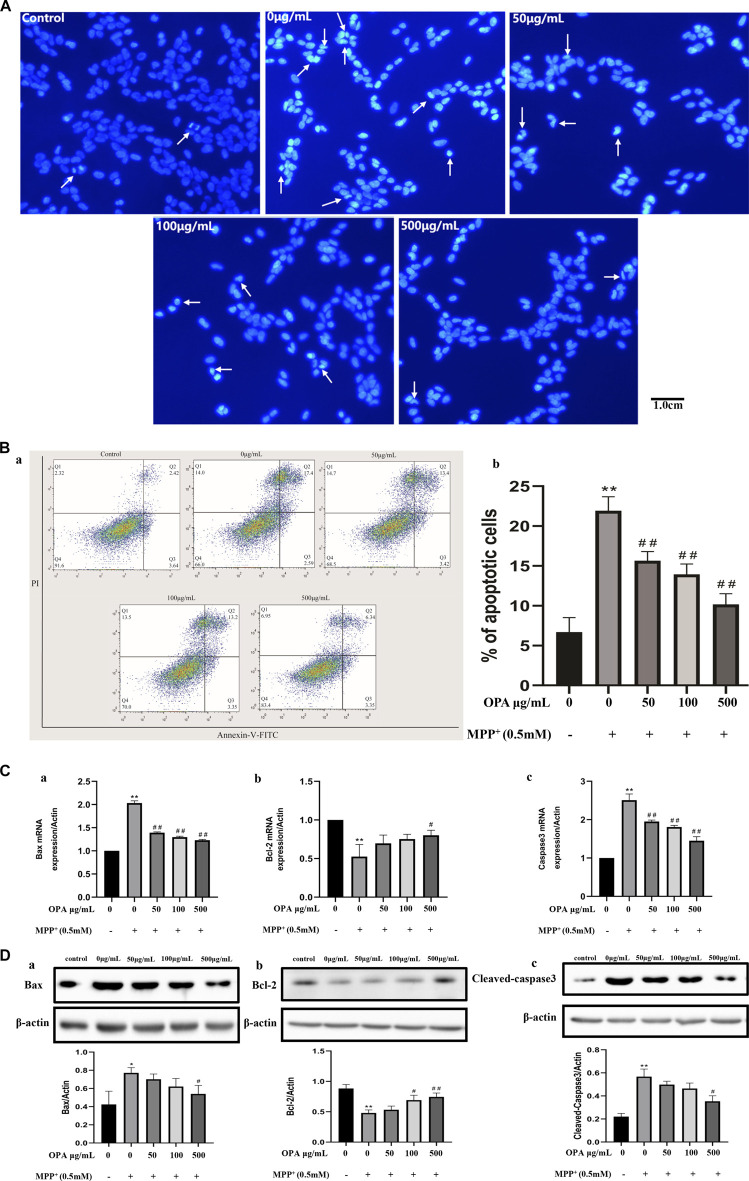
Effects of OPA on the MPP^+^-induced apoptosis of SH-SY5Y cells. SH-SY5Y cells were treated with and without various concentrations of OPA for 24 h before exposed to MPP+ (0.5 mM) for an additional 24 h. The apoptotic cells were tested by **(A)** Hoechst 33,342 staining and **(B)** flow cytometry analyses, in which (a) was the representative image, and (b) indicated the percentage of apoptotic cells. White arrows represented the location of apoptotic cells. **(C)** qPCR was conducted to analyze the mRNA levels of (a) Bax, (b) Bcl-2 and (c) Caspase3. **(D)** protein levels of (a) Bax, (b) Bcl-2 and (c) Cleaved-Caspase3 were detected by western blot. The density of bands was normalized with that of β-actin. Data were presented as mean ± SD for three independent experiments. *p < 0.05, **p < 0.01 compared with control cells untreated with OPA and MPP^+^; ^#^ *p* < 0.05, ^##^ *p* < 0.01 compared with MPP^+^-treated cells.

Hoechst 33342 staining was performed to assess nuclear morphology. Apoptotic nuclei characterized with condensed nuclei and nuclear fragmentation were apparent after treatment with 0.5 mM MPP^+^ in SH-SY5Y cells. These changes in nuclear characteristics of apoptosis were rescued significantly in the cells pretreated with the different concentrations of OPA (50–500 μg/ml) (Figure 2A). The results showed that pretreatment with OPA reduced the damage of MPP^+^ to SH-SY5Y cells. The annexin-V^−^/PI^−^ population was made up of normal healthy cells, and annexin-V^+^/PI^−^ cells existed in early apoptotic stage, while annexin-V^+^/PI^+^ cells were present in late apoptotic/necrotic stage. Treatment with 0.5 mM MPP^+^ increased the percentage of apoptotic cells (19.99%) compared with the control group (6.06%). Treatment with OPA (50–500 μg/ml) prior to MPP^+^ reduced the percentage of apoptotic cells to 16.82%, 16.55%, and 9.69%, respectively (Figure 2B). To test whether OPA could attenuate MPP + -induced apoptosis, the activity of Bax, Bcl-2, caspase3, as well as cleaved-caspase3 protein expression, was detected. The results of qPCR showed that the mRNA levels of Bax and Caspase3 were significantly upregulated, while the levels of Bcl-2 mRNA remarkably declined in SH-SY5Y cells exposed to 0.5 mM MPP^+^ compared with the control group (*p* < 0.01). However, pretreatment with OPA (50–500 μg/ml) showed a reverse effect on the relative levels of mRNA in a dose-dependent manner, compared with the MPP^+^ group (*p* < 0.01) (Figure 2C). As shown in Figure 2D, the protein levels of Bax and cleaved-caspase3 in MPP^+^ goup were distinctly higher than those of control group. As for the levels of Bcl-2 protein, a significant loss of Bcl-2 expression occurred in SH-SY5Y cells that were treated with MPP^+^ (*p* < 0.01). The above changes were also observably reversed after SH-SY5Y cells were pretreated with various concentrations of OPA for 24 h, which suggested that OPA could prevent SH-SY5Y cells from MPP^+^-induced apoptosis by regulating the expression of apoptosis-related genes.

### 3.3 OPA Influenced the General Conditions of PD Model Mice

**FIGURE 3:**
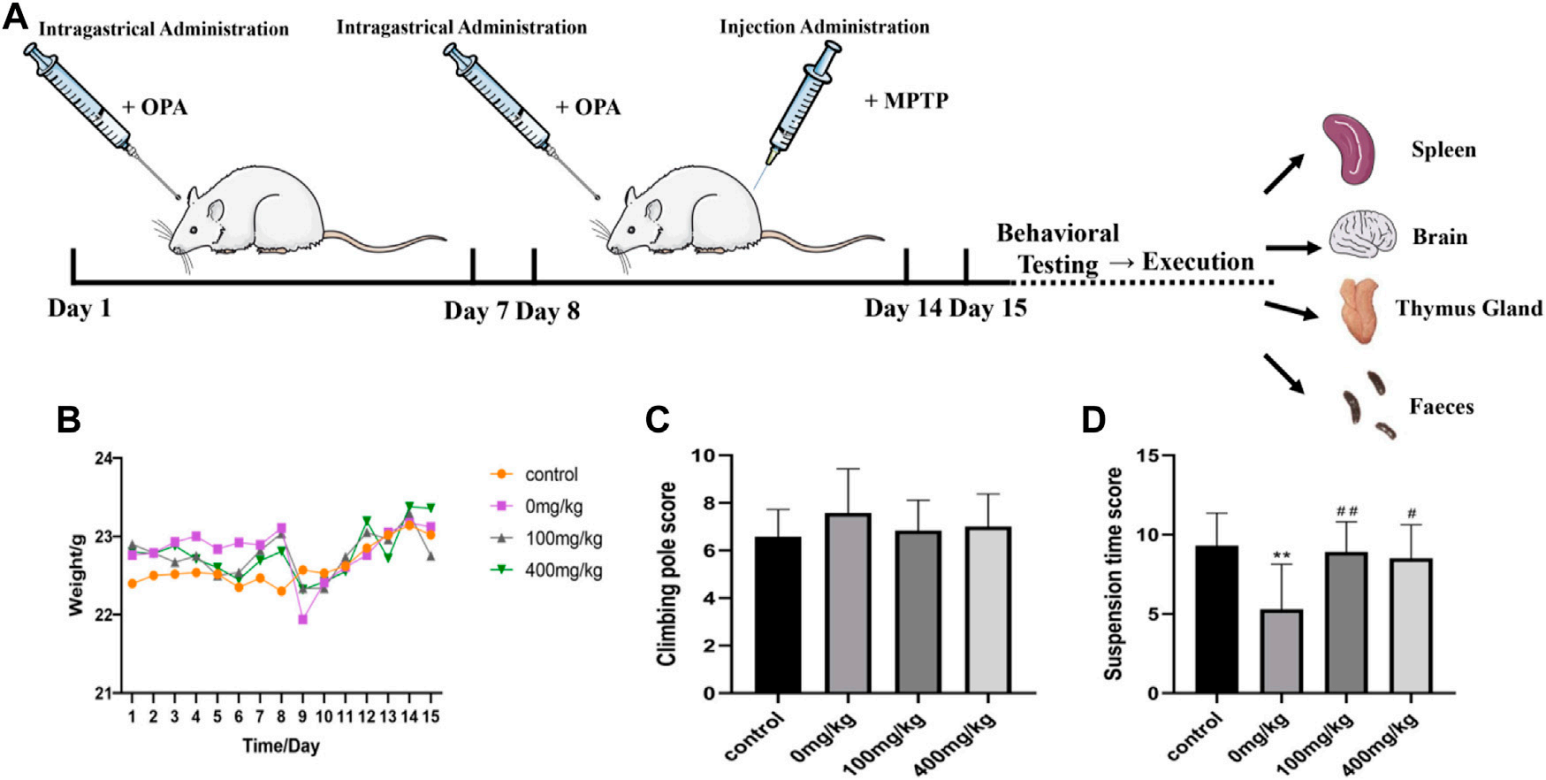
Effects of OPA on weight and behavior of MPTP-induced PD model mice. **(A)** the scheme of experimental procedures. **(B)** the change of body weight during 14 days (*n* = 10, mean ± SD); **(C)** The results of PD model mice in climbing rod test (*n* = 10, mean ± SD); **(D)** the difference of PD model mice in suspension test (*n* = 10, mean ± SD). **p < 0.01 compared with control mice injected with saline; ^#^ *p* < 0.05, ^##^ *p* < 0.01 compared with MPTP-treated mice.

Following the administration of MPTP, general conditions, such as the living status, body weight, immune organs, and motor ability of mice, had been reported to change, indicating that the animals responded to MPTP administration. In our experiment, we chose 8 weeks of healthy wild type C57BL/6 male mice, hypodermic MPTP (30 mg/kg/day), 7 days, to prepare subacute PD model mice (Figure 3A). Consistent with the phenomenon observed in previous studies, the mice developed restlessness symptoms after 5 min of MPTP injection, such as erect hair and tremor. After 30 min, the mice were excitedly running and climbing in the cage. After 2 h, the symptoms gradually disappeared, activity decreased, and exercise slowed down. Then, the mice gathered together (Supplementary Figure S1). According to the record of mouse weight, treatment with 30 mg/kg MPTP decreased sharply the weight of PD model mice (1.17 g) compared to the control group with an average rise of 0.27 g, which indicated that weight loss may be related to gastrointestinal dysfunction caused by MPTP. Also, pretreatment with low-dose (100 mg/kg) and high-dose (400 mg/kg) OPA prior to MPTP reduced the weight to 0.7 and 0.49 g, respectively (Figure 3B). The results showed that OPA could alleviate the effects of dietary intake or food digestion of mice induced by MPTP. After 7 consecutive days of intraperitoneal injection with MPTP, rod climbing behavior was tested. The results showed that compared with the control group (6.57 ± 1.16 s), the pole climbing time of mice in the model group was prolonged to varying degrees. However, the time of mice pretreated with low-dose and high-dose OPA was 6.83 ± 1.28 s and 7.00 ± 1.37 s, respectively, which were slightly lower than that of MPTP model group (7.57 ± 1.87 s) (Figure 3C). In the suspension test, C57BL/6 mice were scored according to the scoring criteria. The higher the score, the firmer the mice grasped the wire. It indicated that with better coordination of limb movements in mice, and vice versa, the situation of tremor and muscle stiffness in mice was serious. As displayed in Figure 3D, the scores of suspension time in MPTP model group (5.3 points) were less than those in control group, while compared with the control group (9.3 points), no significant difference was found in the scores of mice pretreated with OPA. Among them, the scores of mice pretreated with low-dose and high-dose OPA were 8.9 points and 8.5 points, respectively, which were higher than that of MPTP-treated group. These results further suggested that OPA could improve the motor dysfunction of PD model mice induced by MPTP.

### 3.4 OPA Alleviated the Loss of TH-Positive Cells and Apoptosis of Substantia Nigra in PD Model Mice

**FIGURE 4:**
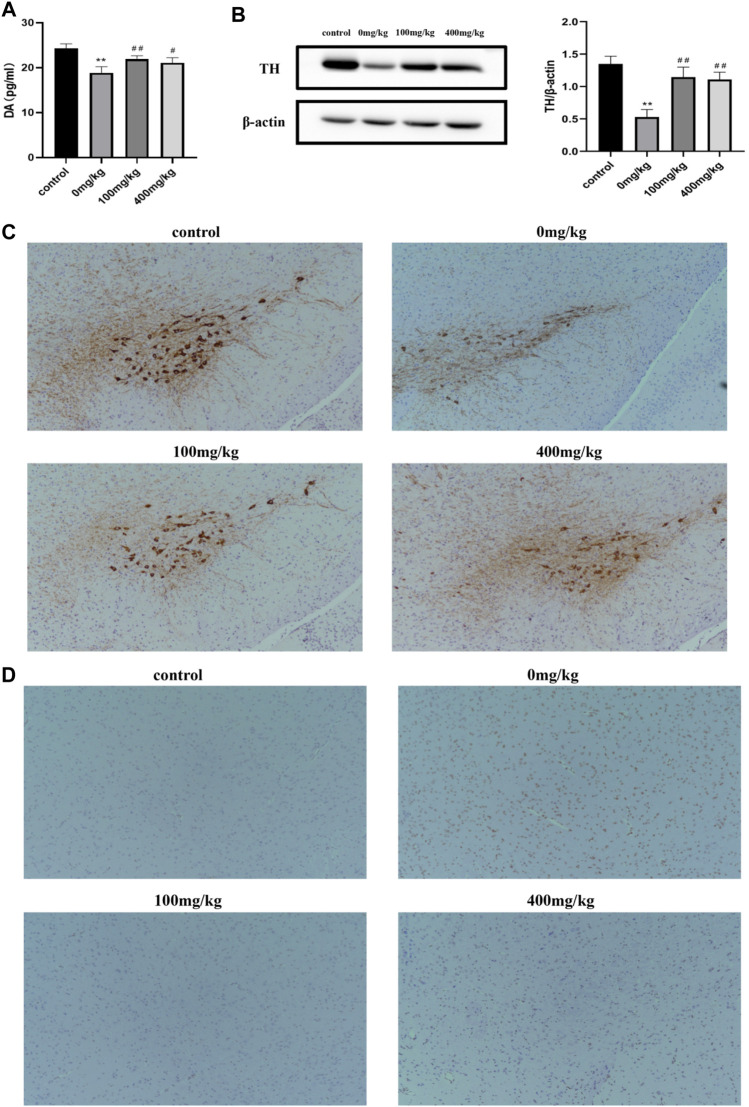
Effects of OPA on striatum and substantia nigra of MPTP-induced PD model mice. **(A)** the contents of striatal DA in PD model mice were quantified by ELISA (*n* = 6, mean ± SD); **(B)** the protein levels of striatal TH were measured by western blot. The density of the bands was normalized with that of β-actin (*n* = 6, mean ± SD); **(C)** the number of TH-positive cells in the SN was quantitatively analyzed by immunohistochemistry (*n* = 4, mean ± SD); **(D)** the levels of apoptotic neurons were detected by TUNEL assay. Brown indicated the apoptotic cells (*n* = 4, mean ± SD). **p < 0.01 compared with control mice injected saline; ^#^ *p* < 0.05, ^##^ *p* < 0.01 compared with MPTP-treated mice.

**FIGURE 5:**
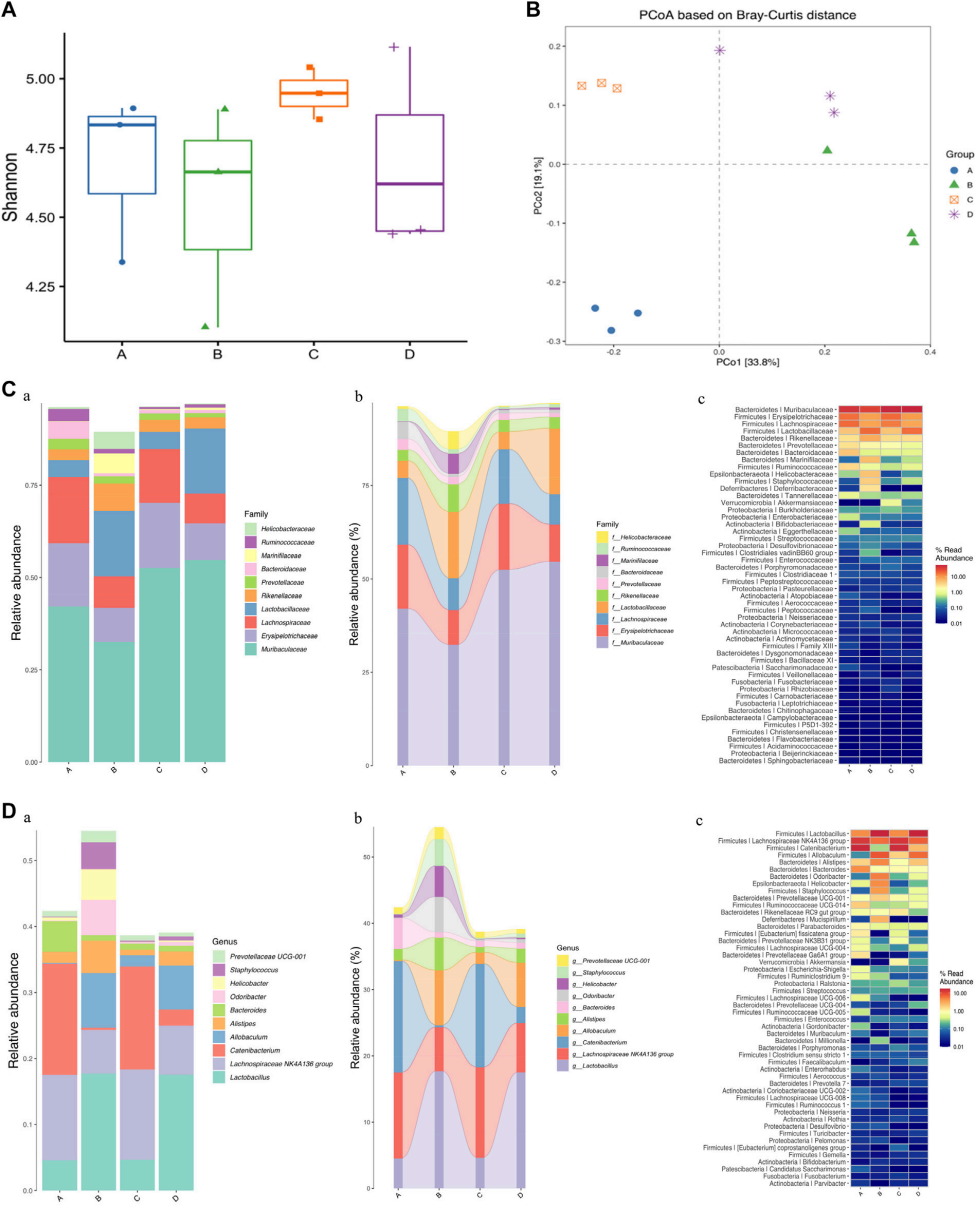
Effects of OPA on gut microbiota in MPTP-induced PD model mice. **(A)** analysis of alpha diversity based on 16S rRNA sequencing-predicted gut microbiota abundance by the Shnnon index; **(B)** PCoA based on Bray-Curtis of gut microbiota where samples of mice from different groups were highlighted with different colors. Principal components (PCs) 1 and 2 explained 33.8% and 19.1% of the variance, respectively. The position and distance of data points indicated the degree of similarity in terms of both the presence and relative abundance of bacterial taxonomies; Analyses of relative abundance in gut microbiota at the **(C)** family and **(D)** genus level among different groups, based on 16S rRNA sequencing (*n* = 3, mean ± SD). (a) Barplot; (b) Sankeyplot; (c) Heatmap; in above statistic graphs, A represented the control group injected with saline; B represented 0 mg/kg group injected with MPTP; C represented low-dose group injected with 100 mg/kg OPA; D represented high-dose group injected with 400 mg/kg OPA.

Substantia nigra (SN)-striatal transport pathway has been reported to take effect in PD pathogenesis. The concentration of DA indicates the ability of DAergic neurons to compound and also reflects the degree of damage to DAergic neurons and the potential neuroprotective effects of OPA on brain function. As measured by ELISA in our experiments, the striatal DA levels in MPTP-treated mice were remarkably decreased by 22.27% (*p* < 0.01) compared with those in control mice injected with saline. However, a significant rise occurred in mice pretreated with low-dose and high-dose OPA, in which the DA levels increased by 15.98% and 11.81%, respectively (Figure 4A). These results suggested that OPA exerted influence on the metabolism of brain neurotransmitters by restraining the MPTP-induced decrease of striatal DA. Moreover, TH, the marker of DAergic neurons and rate-limiting enzyme in the procession of DA synthesis, not only exists in the perinucleus of DAergic neurons but also in the DAergic nerve fibers of the striatum. To detect the activity of TH expression, we firstly measured the protein level of striatal TH by western blot. As shown in Figure 5B, compared with control mice, a prominent decrease occurred in striatal TH expression of mice injected with MPTP. However, OPA could significantly inhibit the reduction of striatal TH expression induced by MPTP (Figure 4B). As the brain region with abundant TH, SN was analyzed by immunohistochemistry assay to detect the levels of TH expression. As displayed in Figure 4C, it presented the same result that compared with the control group, a significant loss of TH-positive DAergic neurons in MPTP-treated mice also occurred. When pretreated with OPA, the levels of TH expression in DAergic neurons demonstrated an obviously rising effect than those in MPTP-treated group. These results demonstrated that the neuroprotective effects of OPA involved the inhibition of TH-positive DAergic neurons loss in MPTP-induced PD model mice. The appearance of PD patients’ symptoms is inseparable from the apoptosis of DAergic neurons. To prove that OPA influenced the survival of DAergic neurons in SN, we tested the apoptotic cells in the SN by TUNEL staining. The results demonstrated that compared with the control mice, the number of apoptotic neurons was increased in MPTP-induced PD model mice, while in OPA-pretreated PD mice, the MPTP-induced apoptosis of DAergic neurons was markedly inhibited. Moreover, pretreatment with low-dose OPA could more effectively protect SN cells against apoptosis induced by MPTP than the high-dose OPA-pretreated group (Figure 4D). All results confirmed that OPA demonstrated neuroprotective effects on MPTP-induced subacute model mice by increasing the levels of DA, enhancing the activity of TH expression in both striatum and SN cells, and inhibiting neurons apoptosis.

### 3.5 OPA Improved the Dysbiosis of Gut Microbiota in MPTP-Induced PD Model Mice

At present, researchers confirmed that the gut microbiota may play a crucial part in progression of PD. In order to verify whether OPA could further benefit the gut microbial dysbiosis by affecting the microbiota composition in MPTP-induced PD model mice, relative analysis based on alpha and beta diversity measures was conducted, which provided a holistic view of gut microbiota and focus on abundance, diversity, and distribution of intestinal microorganism. Shannon index of alpha diversity was used to investigate community diversity, which the larger value represented the higher diversity. As displayed in Figure 5A, a decline in the Shannon diversity index occurred in MPTP-treated mice compared with that of control mice injected with saline. Also, the alpha diversity indices of Shannon tended to be higher in PD model mice pretreated with low-dose OPA. Beta diversity was further evaluated with PCoA, which determined the extent of similarity between microbial communities. Although individual differences were significant, the clusters of gut microbiota in PD model mice were clearly separated from those of mice pretreated with OPA (Figure 5B). To explore the abundance and distribution in gut microbiota and the potential bacterial groups concerned with microbial dysbiosis, we identified a few altered species at the family and genus level. The barplot, sankeyplot, and heatmap all revealed a significant difference in relative sample abundance across the groups at the family and genus level. A significant decrease was observed in the abundance of intestinal microorganism, such as *Muribaculaceae*, *Erysipelotrichaceae*, *Lachnospiraceae*, principally at the family level in MPTP-induced PD mice compared with control mice. An obvious increase occurred in those of OPA-pretreated PD mice in varying degrees. However, the abundance of *Lactobacillaceae*, *Rikenellaceae*, *Marinifilaceae* were strikingly higher in MPTP-treated mice than those of control mice, while no statistically significant difference was found in those of PD mice injected with low-dose OPA compared with control mice (Figure 5C; Supplementary Table S1.1). The significant difference in gut microbiota was also obviously displayed at both family and genus levels among those groups. The decreased abundance of *Lachnospiraceae* NK4A136 group, *Catenibacterium*, *Bacteroides*, and increased abundance of *Lactobacillus*, *Odoribacter*, *Prevotellaceae* UCG-001, were observed in MPTP-treated mice compared with control mice. Compared with MPTP-treated mice at the genus level, an opposite result was found in those of OPA-pretreated mice, in which the abundance of gut microbiota was mostly close to that of control mice, especially in low-dose OPA-pretreated PD mice (Figure 5D; Supplementary Table S1.2). Together, these results indicated that OPA could modulate the microbiota compositions to improve gut microbial dysbiosis induced by MPTP in PD model mice.

## 4 Discussion

Mounting evidence indicates that although different morphological types of cell death co-exist in the brain of PD patients, apoptosis is considered to be one of the most important types of cell death, which is essential for constructing effective neuronal networks in the brain (Perier et al., 2012). Apoptosis is a type of programmed cell death, which is characterized by morphological changes such as cell shrinkage and chromatin condensation. Also, this physiologic process is essential for development, organ morphogenesis, tissue homeostasis, and defense against infected or damaged cells. Generally, apoptosis can be divided into intrinsic (mitochondrial) and extrinsic (death receptor) pathways. Both of these rely on the activation of caspases, a family of cysteine proteases that specifically target aspartic acid residues (Taylor et al., 2008). In the intrinsic pathway, caspase9 and 3 are successively activated, whereas the extrinsic pathway is activated by caspase8 and 3. Besides, Bcl-2-associated X protein (BAX) and anti-apoptotic BCL-2 family members including BCL-2 play a vital role together in response to external apoptotic signals (Liu et al., 2019). However, excessive or abnormal re-activation of apoptosis is associated with neurodegenerative diseases, cancer, and autoimmune diseases (Comi et al., 2012; Ghavami et al., 2014; Lopez and Tait, 2015). With a high lipid solubility and a great accessibility into the brain, MPTP can be catalyzed by monoamine oxidase B located in the outer membrane of glial cells to produce the DA structural analogue, while MPP^+^ can trigger PD-like symptoms by inducing typical apoptosis in midbrain dopamine neurons and model cell lines (such as SH-SY5Y and PC12 cells), accompanied by reactive oxygen species (ROS) production, caspases activation, and DNA fragmentation. Thus, MPTP and MPP^+^ have been selected to establish PD models of vivo and vitro because of the typical neurotoxicity in a large number of research studies (Li et al., 2017; Jiang et al., 2018; Vivacqua et al., 2020). In the present, SH-SY5Y cells induced by MPP^+^ and subcutaneous injection of C57BL/6 mice with MPTP were used to establish PD models *in vitro* and *in vivo*, respectively.

In recent times, more and more researchers are concerned with the connection between poly/oligosaccharides and apoptosis in the pathological and behavioral changes of neurodegenerative diseases. For instance, *Lycium barbarum* polysaccharides (LBP), extracted from the fruits of *Lycium barbarum L.,* proved to be an effective antioxidant, and the neuroprotective properties that LBP prevented 6-OHDA-induced apoptosis in a dose-dependent manner in PC12 cells, through the ROS-NO pathway in the mechanism of slowing the accumulation of ROS, decreasing the level of protein-bound 3-nitrotyrosine (3-NT) and inhibiting the overexpression of nuclear factor κB (NF-κB) (Gao et al., 2014). This study aimed to explore the biological function and specific mechanism of OPA in regulating neuron apoptosis in PD. The present results showed that treating SH-SY5Y cells with MPP^+^ prominently reduced cell viability and induced apoptosis, elevated the level of Bax and Caspase3, but decreased the level of Bcl-2, which were in accordance with the previous reports (Wang and XU, 2005; Kim et al., 2016; Kim and Park, 2018; Liu et al., 2020b; Liu et al., 2020c; Yang et al., 2020; Zhou et al., 2021). However, according to the experimental results of Hoechst 33342 staining and flow cytometry, the apoptosis of SH-SY5Y cells was remarkably inhibited by the pretreatment of OPA in the PD cell model. Moreover, OPA could decrease the expression of pro-apoptosis-associated genes, such as caspase3 and Bax, and reduce the ratio of Bax/Bcl-2. The above results showed that a significant improvement was found on the apoptosis of SH-SY5Y cells in a dose-dependent manner after the pretreatment of OPA, which suggested that OPA demonstrated a cell-protective effect in MPP^+^-induced cell models of PD by restoring cell morphological changes, increasing cell survival rate, and regulating the expression of apoptosis-related genes. Among PD animal models, behavioral testing of mice is essential. An *in vivo* study using MPTP-conditioned animal models was necessary to illustrate the potential value of OPA for clinical application. Our results showed that OPA ameliorated the poor rod climbing behavior and suspension ability of MPTP-induced PD model mice. These findings indicated that OPA can improve MPTP-induced motor impairment in PD mice.

DA is a key neurotransmitter, which mainly regulates balance and movement, and the perturbations of DA signaling are involved in the etiopathogenesis or utilized in the treatment of PD (Sun et al., 2018). As a product of DAergic neurons in SN, DA demonstrates unstable properties and is particularly susceptible to be oxidized, in which the generated ROS can increase oxidative stress and lead to neurodegeneration (Sanders and Timothy Greenamyre, 2013). Even obstruction of DA’s storage and transport can cause cell damage (Uhl et al., 2000). In contrast, the utilization of medicinal L-3,4-dihydroxyphenylalanine (L-DOPA) contributes to improving PD symptom via the increase in DA content though accompanied by side effects such as dyskinesia, which is still the classic approach of PD treatment (Bartholini et al., 1989; Obeso et al., 2000). Moreover, as the rate-limiting enzyme of catecholamine synthesis, such as DA, noradrenaline, and adrenaline, TH catalyzes the hydroxylation of tyrosine to L-DOPA (Molinoff and Axelrod, 1971). The α-synuclein is a major component of Lewy bodies that may bind to TH and inhibit its activity, while loss of TH activity and reduced protein levels are thought to be responsible for decreased DA levels and the PD phenotype (Nagatsu, 1990; Blanchard-Fillion et al., 2001; Perez et al., 2002; Corti et al., 2011). In PD patients, because of the apoptosis of dopamine neurons in the SNpc, which is the brain region with the most TH-rich, the level of DA transported to the striatum is low (Daubner et al., 2011). As expected, the decrease of DA levels, loss of TH-positive cells, and apoptosis of SN cells were induced by MPTP, which were inhibited by OPA pretreatment.

The gut microbiome has been reported to play an important role in the deep interconnection between the gut and the brain, which is widely accepted as the microbiome-gut-brain axis. The process involves that microbiome in the gut modulates brain functionality and activity via its metabolic products and ability to stimulate the ENS by sending neuronal signals directly to the brain via the vagus nerve (Sun and Shen, 2018). A healthy and stable gut microbiota community is conducive to homeostatic balance of barrier integrity, function, metabolism, and immunity of the gut, as well as regulating the gut-brain axis (Jang et al., 2020). Studies showed that there are not only disorders of intestinal flora, but also an obvious difference in the abundance and composition of gut microbes in both PD patients and MPTP-induced PD mice (Sun et al., 2018)^,^ (Lai et al., 2018; Zhou et al., 2019; Travagli et al., 2020). Also, alterations in the composition and number of gut microbiota are found in PD patients. For example, gut microbial dysbiosis has been found to be related to the pathogenesis of PD, as reflected by a significant decrease in *Prevotellaceae*, which has been revealed to participate in regulating DAergic neuron function in SNpc and fight against neurodegeneration (Shen et al., 2021), from fecal samples of PD patients compared with that of healthy individuals (Sun and Shen, 2018). Our results of relative analysis based on alpha and beta diversity measures demonstrated that significant differences were found in the relative abundance and distribution of gut microbiota at the family and genus level across control mice, PD mice, and OPA-recipient PD mice. Moreover, we demonstrated that gut microbial dysbiosis in PD mice involved the decreased abundance of *Prevotellacea*, *Erysipelotrichaceae*, and *Lachnospiraceae*. Also, increased abundance of the beneficial bacteria including *Lactobacillaceae* and *Rikenellaceae* in family level was consistent with observations in human subjects with PD, while no statistically significant difference was found in those of PD mice injected with low-dose OPA. Previous research showed that the decrease of *Prevotellaceae* and the increase of *Lactobacillacea* could modulate the production of microbial short-chain fatty acids (Wang et al., 2021) and affect the secretion of growth hormone, thereby influencing the normal function of the dopamine system and participating in the occurrence and development of degenerative neurological diseases. In particular, it is known that the decrease in abundance of *Erysipelotrichaceae* and increased abundance of *Bacteroides* can be a consequence of gut inflammation, especially increased plasma levels of tumor necrosis factor-alpha (TNF-α) (Dey et al., 2013; Labbé et al., 2014; Metta et al., 2022). As seen in Figure 5D, compared with the PD model group, the levels of pathogenic bacterium were decreased by OPA pretreatment, such as *Odoribacter*, *Staphylococcus*, and *Prevotellaceae* UCG-001, which generated toxin and then triggered inflammation. In fact, gut dysbiosis (microbiota dysregulation) has been correlated with a series of neurodegenerative diseases, including AD, HD, and PD. For instance, GV-971, a sodium oligomannate, has been reported to possess significant potential to alleviate neuroinflammation by reconstituting gut microbiota, which accomplished the Phase 3 clinical trial for AD in China (Wang et al., 2019). In addition, transplants of gut microbiota from PD patients into mice could exacerbate motor deficits, underlining the interplay between gut microbiota and the progression of PD (Sampson et al., 2016). In PD patients with dysbiosis, a higher abundance of *Enterobacteriaceae* has also reported to correlate with severity of motor signs, including gait abnormalities and postural instability (Scheperjans et al., 2015). Meanwhile, our results suggested that gut microbial dysbiosis owing to specific microbes might be implicated in PD progression and clinical manifestation. Also, OPA could improve intestinal dysbiosis in MPTP-induced PD mice by regulating the composition of enteric microorganism that the abundance of beneficial bacteria was increased, while that of pathogenic bacteria was reduced in feces.

## 5 Conclusion

**FIGURE 6:**
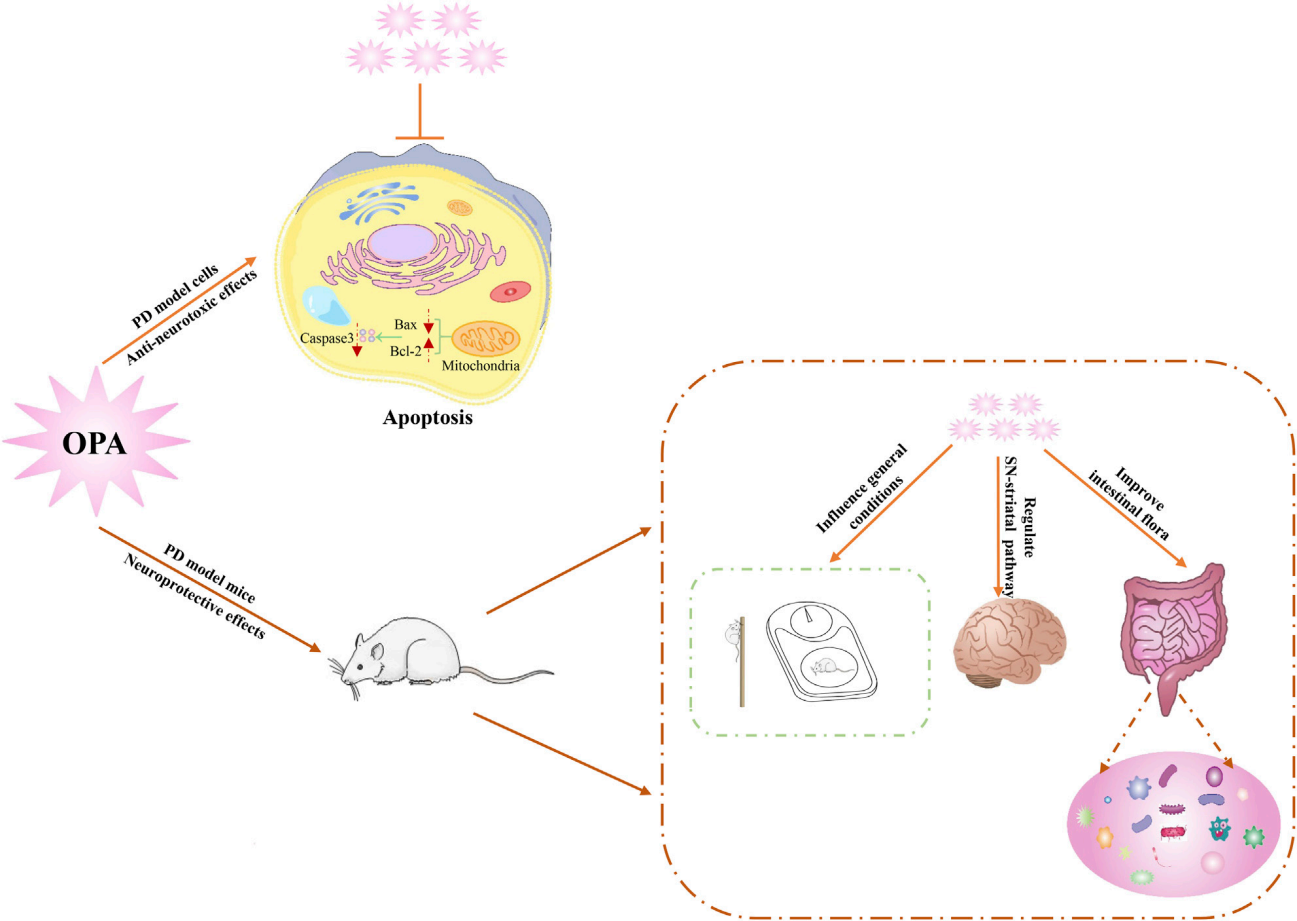
Schematic diagram of underlying mechanism for neuroprotective effects of OPA on PD models.

In summary, OPA exerted the neuroprotective effects by effectively inhibiting the toxicity of MPP^+^ on SH-SY5Y cells, improving behavioral deficits, attenuating the loss of DAergic neurons, and improving gut microbial dysbiosis in MPTP-exposed mice (Figure 6). Our findings uncovered a potent role of OPA-mediated apoptosis in the pathogenesis of PD and also provided an important clue for exploring potential targets for novel therapeutics of PD.

## Data Availability

The datasets presented in this study can be found in online repositories. The names of the repository/repositories and accession number(s) can be found in the article/Supplementary Material.

## References

[B1] Aubignat M. Tir M. Krystkowiak P. (2020). Non-motor Symptoms of Parkinson's Disease from Pathophysiology to Early Diagnosis. Rev. Med. Interne 42, 251–257. 10.1016/j.revmed.2020.06.019 32680717

[B2] Auger S. D. Kanavou S. Lawton M. Ben-Shlomo Y. Hu M. T. Schrag A. E. (2020). Testing Shortened Versions of Smell Tests to Screen for Hyposmia in Parkinson's Disease. Mov. Disord. Clin. Pract. 7, 394–398. 10.1002/mdc3.12928 32373655 PMC7197313

[B3] Bartholini G. Zivkovic B. Scatton B. (1989). “Dopaminergic Neurons: Basic Aspects,” in Catecholamines II (Springer), 277–318. 10.1007/978-3-642-73551-6_7

[B4] Blanchard-Fillion B. Souza J. M. Friel T. Jiang G. C. Vrana K. Sharov V. (2001). Nitration and Inactivation of Tyrosine Hydroxylase by Peroxynitrite. J. Biol. Chem. 276, 46017–46023. 10.1074/jbc.M105564200 11590168

[B5] Cai W. Xu J. Li G. Liu T. Guo X. Wang H. (2020). Ethanol Extract of Propolis Prevents High-Fat Diet-Induced Insulin Resistance and Obesity in Association with Modulation of Gut Microbiota in Mice. Food Res. Int. 130, 108939. 10.1016/j.foodres.2019.108939 32156386

[B6] Charlett A. Dobbs R. J. Dobbs S. M. Weller C. Brady P. Peterson D. W. (1999). Parkinsonism: Siblings Share *Helicobacter pylori* Seropositivity and Facets of Syndrome. Acta Neurol. Scand. 99, 26–35. 10.1111/j.1600-0404.1999.tb00654.x 9925235

[B7] Comi C. Fleetwood T. Dianzani U. (2012). The Role of T Cell Apoptosis in Nervous System Autoimmunity. Autoimmun. Rev. 12, 150–156. 10.1016/j.autrev.2011.08.011 22504460

[B8] Corti O. Lesage S. Brice A. (2011). What Genetics Tells Us about the Causes and Mechanisms of Parkinson's Disease. Physiol. Rev. 91, 1161–1218. 10.1152/physrev.00022.2010 22013209

[B9] Daubner S. C. Le T. Wang S. (2011). Tyrosine Hydroxylase and Regulation of Dopamine Synthesis. Arch. Biochem. Biophys. 508, 1–12. 10.1016/j.abb.2010.12.017 21176768 PMC3065393

[B10] Dey N. Soergel D. A. Repo S. Brenner S. E. (2013). Association of Gut Microbiota with Post-operative Clinical Course in Crohn's Disease. BMC Gastroenterol. 13, 131. 10.1186/1471-230X-13-131 23964800 PMC3848607

[B11] Gao K. Liu M. Cao J. Yao M. Lu Y. Li J. (2014). Protective Effects of Lycium Barbarum Polysaccharide on 6-OHDA-Induced Apoptosis in PC12 Cells through the ROS-NO Pathway. Molecules 20, 293–308. 10.3390/molecules20010293 25547727 PMC6272587

[B12] Ghavami S. Shojaei S. Yeganeh B. Ande S. R. Jangamreddy J. R. Mehrpour M. (2014). Autophagy and Apoptosis Dysfunction in Neurodegenerative Disorders. Prog. Neurobiol. 112, 24–49. 10.1016/j.pneurobio.2013.10.004 24211851

[B13] Hu X. Song Q. Li X. Li D. Zhang Q. Meng W. (2018). Neuroprotective Effects of Kukoamine A on Neurotoxin-Induced Parkinson's Model through Apoptosis Inhibition and Autophagy Enhancement. Neuropharmacology 117, 352–363. 10.1016/j.neuropharm.2017.02.022 28238714

[B14] Jang J. H. Yeom M. J. Ahn S. Oh J. Y. Ji S. Kim T. H. (2020). Acupuncture Inhibits Neuroinflammation and Gut Microbial Dysbiosis in a Mouse Model of Parkinson's Disease. Brain Behav. Immun. 89, 641–655. 10.1016/j.bbi.2020.08.015 32827699

[B15] Jiang Y. Liu H. Ji B. Wang Z. Wang C. Yang C. (2018). Apelin-13 Attenuates ER Stress-Associated Apoptosis Induced by MPP^+^ in SH-Sy5y Cells. Int. J. Mol. Med. 42, 1732–1740. 29901077 10.3892/ijmm.2018.3719

[B16] Keshavarzian A. Green S. J. Engen P. A. Voigt R. M. Naqib A. Forsyth C. B. (2015). Colonic Bacterial Composition in Parkinson's Disease. Mov. Disord. 30, 1351–1360. 10.1002/mds.26307 26179554

[B17] Kim C. Park S. (2018). IGF-1 Protects SH-Sy5y Cells against MPP+-induced Apoptosis via PI3K/PDK-1/Akt Pathway. Endocr. Connect. 7, 443–455. 10.1530/EC-17-0350 29459421 PMC5843822

[B18] Kim I. S. Ganesan P. Choi D. K. (2016). Cx43 Mediates Resistance against MPP⁺-Induced Apoptosis in SH-Sy5y Neuroblastoma Cells via Modulating the Mitochondrial Apoptosis Pathway. Int. J. Mol. Sci. 17, 1819. 10.3390/ijms17111819 PMC5133820 27809287

[B19] Kim M.-H. Namgoong H. Jung B.-D. Kwon M.-S. Choi Y.-S. Shin T. (2017). Fucoidan Attenuates 6-Hydroxydopamine-Induced Neurotoxicity by Exerting Anti-oxidative and Anti-apoptotic Actions in SH-Sy5y Cells. Korean J. Veterinary Res. 57, 1–7. 10.14405/kjvr.2017.57.1.1

[B20] Kong G. Cao K. L. Judd L. M. Li S. Renoir T. Hannan A. J. (2018). Microbiome Profiling Reveals Gut Dysbiosis in a Transgenic Mouse Model of Huntington's Disease. Neurobiol. Dis. 135, 104268. 10.1016/j.nbd.2018.09.001 30194046

[B21] Kuang L. Cao X. Lu Z. (2017). Baicalein Protects against Rotenone-Induced Neurotoxicity through Induction of Autophagy. Biol. Pharm. Bull. 40, 1537–1543. 10.1248/bpb.b17-00392 28659545

[B22] Kumaresan M. Khan S. (2021). Spectrum of Non-motor Symptoms in Parkinson's Disease. Cureus 13, e13275. 10.7759/cureus.13275 33728210 PMC7949722

[B23] Labbé A. Ganopolsky J. G. Martoni C. J. Prakash S. Jones M. L. (2014). Bacterial Bile Metabolising Gene Abundance in Crohn's, Ulcerative Colitis and Type 2 Diabetes Metagenomes. PloS One 9, e115175. 10.1371/journal.pone.0115175 25517115 PMC4269443

[B24] Lai F. Jiang R. Xie W. Liu X. Tang Y. Xiao H. (2018). Intestinal Pathology and Gut Microbiota Alterations in a Methyl-4-Phenyl-1,2,3,6-Tetrahydropyridine (MPTP) Mouse Model of Parkinson's Disease. Neurochem. Res. 43, 1986–1999. 10.1007/s11064-018-2620-x 30171422

[B25] Lee S. J. Bose S. Seo J. G. Chung W. S. Lim C. Y. Kim H. (2014). The Effects of Co-administration of Probiotics with Herbal Medicine on Obesity, Metabolic Endotoxemia and Dysbiosis: a Randomized Double-Blind Controlled Clinical Trial. Clin. Nutr. 33, 973–981. 10.1016/j.clnu.2013.12.006 24411490

[B26] Li H. Shi R. Ding F. Wang H. Han W. Ma F. (2016). Astragalus Polysaccharide Suppresses 6-Hydroxydopamine-Induced Neurotoxicity in *Caenorhabditis elegans* . Oxid. Med. Cell Longev. 2016, 4856761. 10.1155/2016/4856761 27885333 PMC5112302

[B27] Li J. Chen L. Qin Q. Wang D. Zhao J. Gao H. (2022). Upregulated Hexokinase 2 Expression Induces the Apoptosis of Dopaminergic Neurons by Promoting Lactate Production in Parkinson's Disease. Neurobiol. Dis. 163, 105605. 10.1016/j.nbd.2021.105605 34973450

[B28] Li X. M. Zhang X. J. Dong M. X. (2017). Isorhynchophylline Attenuates MPP+-Induced Apoptosis through Endoplasmic Reticulum Stress- and Mitochondria-dependent Pathways in PC12 Cells: Involvement of Antioxidant Activity. Neuromolecular Med. 19, 480–492. 10.1007/s12017-017-8462-x 28822073

[B29] Lim H. S. Kim J. S. Moon B. C. Ryu S. M. Lee J. Park G. (2019). Batryticatus Bombyx Protects Dopaminergic Neurons against MPTP-Induced Neurotoxicity by Inhibiting Oxidative Damage. Antioxidants (Basel) 8, 574. 10.3390/antiox8120574 PMC6943627 31766449

[B30] Liu H. Wang J. Zhang Q. Geng L. Yang Y. Wu N. (2020). Protective Effect of Fucoidan against MPP+-Induced SH-Sy5y Cells Apoptosis by Affecting the PI3K/Akt Pathway. Mar. Drugs 18, 333. 10.3390/md18060333 PMC7344518 32630523

[B31] Liu J. Liu W. Yang H. (2019). Balancing Apoptosis and Autophagy for Parkinson's Disease Therapy: Targeting BCL-2. ACS Chem. Neurosci. 10, 792–802. 10.1021/acschemneuro.8b00356 30400738

[B32] Liu L. Huh J. R. Shah K. (2022). Microbiota and the gut-brain-axis: Implications for New Therapeutic Design in the CNS. EBioMedicine 77, 103908. 10.1016/j.ebiom.2022.103908 35255456 PMC8897630

[B33] Liu L. Wang J. Wang H. (2020). Hydrogen Sulfide Alleviates Oxidative Stress Injury and Reduces Apoptosis Induced by MPP+ in Parkinson's Disease Cell Model. Mol. Cell Biochem. 472, 231–240. 10.1007/s11010-020-03801-y 32577946

[B34] Liu Y. Jin W. Deng Z. Wang J. Zhang Q. (2020). Preparation and Neuroprotective Activity of Glucuronomannan Oligosaccharides in an MPTP-Induced Parkinson's Model. Mar. Drugs 18, 438. 10.3390/md18090438 PMC7551172 32842556

[B35] Lopez J. Tait S. W. (2015). Mitochondrial Apoptosis: Killing Cancer Using the Enemy within. Br. J. Cancer 112, 957–962. 10.1038/bjc.2015.85 25742467 PMC4366906

[B36] Lu K. Zhou J. Deng J. Li Y. Wu C. Bao J. (2021). *Periplaneta americana* Oligosaccharides Exert Anti-inflammatory Activity through Immunoregulation and Modulation of Gut Microbiota in Acute Colitis Mice Model. Molecules 26, 1718. 10.3390/molecules26061718 33808686 PMC8003390

[B37] Ma X. Hu Y. Li X. Zheng X. Wang Y. Zhang J. (2018). *Periplaneta americana* Ameliorates Dextran Sulfate Sodium-Induced Ulcerative Colitis in Rats by Keap1/Nrf-2 Activation, Intestinal Barrier Function, and Gut Microbiota Regulation. Front. Pharmacol. 9, 944. 10.3389/fphar.2018.00944 30186174 PMC6113651

[B38] Metta V. Leta V. Mrudula K. R. Prashanth L. K. Goyal V. Borgohain R. (2022). Gastrointestinal Dysfunction in Parkinson's Disease: Molecular Pathology and Implications of Gut Microbiome, Probiotics, and Fecal Microbiota Transplantation. J. Neurol. 269, 1154–1163. 10.1007/s00415-021-10567-w 33881598

[B39] Molinoff P. B. Axelrod J. (1971). Biochemistry of Catecholamines. Annu. Rev. Biochem. 40, 465–500. 10.1146/annurev.bi.40.070171.002341 4399447

[B40] Mozaffari S. Nikfar S. Daniali M. Abdollahi M. (2020). The Pharmacological Management of Constipation in Patients with Parkinson's Disease: a Much-Needed Relief. Expert Opin. Pharmacother. 21, 701–707. 10.1080/14656566.2020.1726319 32037901

[B41] Nagatsu T. (1990). Change of Tyrosine Hydroxylase in the Parkinsonian Brain and in the Brain of MPTP-Treated Mice as Revealed by Homospecific Activity. Neurochem. Res. 15, 425–429. 10.1007/BF00969928 1975089

[B42] Nawaz H. Sargent L. Quilon H. Cloud L. J. Testa C. M. Snider J. D. (2022). Anticholinergic Medication Burden in Parkinson's Disease Outpatients. J. Park. Dis. 12, 599–606. 10.3233/JPD-212769 PMC9602053 34806617

[B43] Obeso J. A. Olanow C. W. Nutt J. G. (2000). Levodopa Motor Complications in Parkinson's Disease. Trends Neurosci. 23, S2–S7. 10.1016/s1471-1931(00)00031-8 11052214

[B44] Perez R. G. Waymire J. C. Lin E. Liu J. J. Guo F. Zigmond M. J. (2002). A Role for Alpha-Synuclein in the Regulation of Dopamine Biosynthesis. J. Neurosci. 22, 3090–3099. 10.1523/JNEUROSCI.22-08-03090.2002 11943812 PMC6757524

[B45] Perier C. Bové J. Vila M. (2012). Mitochondria and Programmed Cell Death in Parkinson's Disease: Apoptosis and beyond. Antioxid. Redox Signal 16, 883–895. 10.1089/ars.2011.4074 21619488

[B46] Roy M. Péré M. Damier P. Bruley des Varannes S. Derkinderen P. Rouaud T. (2021). STW5 (Iberogast®) for Constipation in Parkinson's Disease. Rev. Neurol. Paris. 177, 296–301. 10.1016/j.neurol.2020.06.005 32829914

[B47] Sampson T. R. Debelius J. W. Thron T. Janssen S. Shastri G. G. Ilhan Z. E. (2016). Gut Microbiota Regulate Motor Deficits and Neuroinflammation in a Model of Parkinson's Disease. Cell 167, 1469–e12. e12. 10.1016/j.cell.2016.11.018 27912057 PMC5718049

[B48] Sanders L. H. Timothy Greenamyre J. (2013). Oxidative Damage to Macromolecules in Human Parkinson Disease and the Rotenone Model. Free Radic. Biol. Med. 62, 111–120. 10.1016/j.freeradbiomed.2013.01.003 23328732 PMC3677955

[B49] Scheperjans F. Aho V. Pereira P. A. Koskinen K. Paulin L. Pekkonen E. (2015). Gut Microbiota Are Related to Parkinson's Disease and Clinical Phenotype. Mov. Disord. 30, 350–358. 10.1002/mds.26069 25476529

[B50] Shen T. Yue Y. He T. Huang C. Qu B. Lv W. (2021). The Association between the Gut Microbiota and Parkinson's Disease, a Meta-Analysis. Front. Aging Neurosci. 13, 636545. 10.3389/fnagi.2021.636545 33643026 PMC7907649

[B51] Sheu J. J. Tsai M. T. Erickson S. R. Wu C. H. (2019). Association between Anticholinergic Medication Use and Risk of Dementia Among Patients with Parkinson's Disease. Pharmacotherapy 39, 798–808. 10.1002/phar.2305 31251824

[B52] Souza R. B. Frota A. F. Sousa R. S. Cezario N. A. Santos T. B. Souza L. M. (2017). Neuroprotective Effects of Sulphated Agaran from Marine Alga Gracilaria Cornea in Rat 6-Hydroxydopamine Parkinson's Disease Model: Behavioural, Neurochemical and Transcriptional Alterations. Basic Clin. Pharmacol. Toxicol. 120, 159–170. 10.1111/bcpt.12669 27612165

[B53] Sun M. F. Shen Y. Q. (2018). Dysbiosis of Gut Microbiota and Microbial Metabolites in Parkinson's Disease. Ageing Res. Rev. 45, 53–61. 10.1016/j.arr.2018.04.004 29705121

[B54] Sun M. F. Zhu Y. L. Zhou Z. L. Jia X. B. Xu Y. D. Yang Q. (2018). Neuroprotective Effects of Fecal Microbiota Transplantation on MPTP-Induced Parkinson's Disease Mice: Gut Microbiota, Glial Reaction and TLR4/TNF-α Signaling Pathway. Brain Behav. Immun. 70, 48–60. 10.1016/j.bbi.2018.02.005 29471030

[B55] Taylor R. C. Cullen S. P. Martin S. J. (2008). Apoptosis: Controlled Demolition at the Cellular Level. Nat. Rev. Mol. Cell Biol. 9, 231–241. 10.1038/nrm2312 18073771

[B56] Travagli R. A. Browning K. N. Camilleri M. (2020). Parkinson Disease and the Gut: New Insights into Pathogenesis and Clinical Relevance. Nat. Rev. Gastroenterol. Hepatol. 17, 673–685. 10.1038/s41575-020-0339-z 32737460

[B57] Uhl G. R. Li S. Takahashi N. Itokawa K. Lin Z. Hazama M. (2000). The VMAT2 Gene in Mice and Humans: Amphetamine Responses, Locomotion, Cardiac Arrhythmias, Aging, and Vulnerability to Dopaminergic Toxins. FASEB J. 14, 2459–2465. 10.1096/fj.00-0205rev 11099463

[B58] Vivacqua G. Biagioni F. Busceti C. L. Ferrucci M. Madonna M. Ryskalin L. (2020). Motor Neurons Pathology after Chronic Exposure to MPTP in Mice. Neurotox. Res. 37, 298–313. 10.1007/s12640-019-00121-y 31721049

[B59] Vogt N. M. Kerby R. L. Dill-McFarland K. A. Harding S. J. Merluzzi A. P. Johnson S. C. (2017). Gut Microbiome Alterations in Alzheimer's Disease. Sci. Rep. 7, 11. 10.1038/s41598-017-13601-y 29051531 PMC5648830

[B60] Wang Q. Luo Y. Ray Chaudhuri K. Reynolds R. Tan E. K. Pettersson S. (2021). The Role of Gut Dysbiosis in Parkinson's Disease: Mechanistic Insights and Therapeutic Options. Brain 144, 2571–2593. 10.1093/brain/awab156 33856024

[B61] Wang T. Gu J. Wu P. F. Wang F. Xiong Z. Yang Y. J. (2009). Protection by Tetrahydroxystilbene Glucoside against Cerebral Ischemia: Involvement of JNK, SIRT1, and NF-kappaB Pathways and Inhibition of Intracellular ROS/RNS Generation. Free Radic. Biol. Med. 47, 229–240. 10.1016/j.freeradbiomed.2009.02.027 19272442

[B62] Wang X. Sun G. Feng T. Zhang J. Huang X. Wang T. (2019). Sodium Oligomannate Therapeutically Remodels Gut Microbiota and Suppresses Gut Bacterial Amino Acids-Shaped Neuroinflammation to Inhibit Alzheimer's Disease Progression. Cell Res. 29, 787–803. 10.1038/s41422-019-0216-x 31488882 PMC6796854

[B63] Wang X. J. Xu J. X. (2005). Salvianic Acid A Protects Human Neuroblastoma SH-Sy5y Cells against MPP+-Induced Cytotoxicity. Neurosci. Res. 51, 129–138. 10.1016/j.neures.2004.10.001 15681030

[B64] Wasser C. I. Mercieca E. C. Kong G. Hannan A. J. McKeown S. J. Glikmann-Johnston Y. (2020). Gut Dysbiosis in Huntington's Disease: Associations Among Gut Microbiota, Cognitive Performance and Clinical Outcomes. Brain Commun. 2, fcaa110. fcaa110. 10.1093/braincomms/fcaa110 33005892 PMC7519724

[B65] Yang X. Zhang M. Wei M. Wang A. Deng Y. Cao H. (2020). MicroRNA-216a Inhibits Neuronal Apoptosis in a Cellular Parkinson's Disease Model by Targeting Bax. Metab. Brain Dis. 35, 627–635. 10.1007/s11011-020-00546-x 32140823

[B66] Zeng B. Y. (2017). Effect and Mechanism of Chinese Herbal Medicine on Parkinson's Disease. Int. Rev. Neurobiol. 135, 57–76. 10.1016/bs.irn.2017.02.004 28807165

[B67] Zhou S. Zhang D. Guo J. Chen Z. Chen Y. Zhang J. (2021). Deficiency of NEAT1 Prevented MPP+-induced Inflammatory Response, Oxidative Stress and Apoptosis in Dopaminergic SK-N-SH Neuroblastoma Cells via miR-1277-5p/ARHGAP26 axis. Brain Res. 1750, 147156. 10.1016/j.brainres.2020.147156 33069733

[B68] Zhou Z. L. Jia X. B. Sun M. F. Zhu Y. L. Qiao C. M. Zhang B. P. (2019). Neuroprotection of Fasting Mimicking Diet on MPTP-Induced Parkinson's Disease Mice via Gut Microbiota and Metabolites. Neurotherapeutics 16, 741–760. 10.1007/s13311-019-00719-2 30815845 PMC6694382

[B69] Zhuang P. Zhang Y. Shou Q. Li H. Zhu Y. He L. (2020). Eicosapentaenoic and Docosahexaenoic Acids Differentially Alter Gut Microbiome and Reverse High-Fat Diet-Induced Insulin Resistance. Mol. Nutr. Food Res. 64, e1900946. 10.1002/mnfr.201900946 32298529

