# Supplementary material for: Neuroprotective Effects of Oligosaccharides From Periplaneta Americana on Parkinson’s Disease Models In Vitro and In Vivo
Source: Front Pharmacol. 2022 Jul 18;13:936818. doi: 10.3389/fphar.2022.936818 (PMC9340460; doi:10.3389/fphar.2022.936818)
Supplement: Supplementary file 1 [file Table1.DOCX]

Supplementary data

Figure S1:


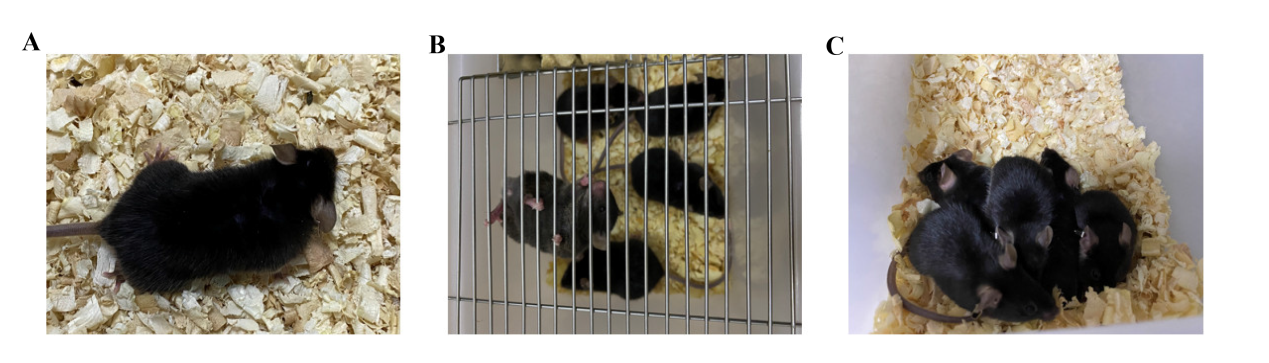


**Figure S1. General behavioral observations after injection of MPTP**

**A.Mice thriller; B.Mice climbing; C.Mice gathering**

Table S1.1:

**Table S1.1. The top ten abundance of gut microbiota at the family level**

| Flora | Group | | | |
| --- | --- | --- | --- | --- |
|  | Control | MPTP | Low-dose OPA | High-dose OPA |
| *Muribaculaceae* | 0.423±0.0377 | 0.3247±0.0033 | 0.5253±0.0297 | 0.5468±0.0340 |
| *Erysipelotrichaceae* | 0.1713±0.0854 | 0.0926±0.1570 | 0.1767±0.0400 | 0.0996±0.0710 |
| *Lachnospiraceae* | 0.1790±0.0456 | 0.0855±0.0089 | 0.1457±0.0022 | 0.0811±0.0207 |
| *Lactobacillaceae* | 0.0457±0.0081 | 0.1775±0.0467 | 0.0468±0.0115 | 0.1758±0.0984 |
| *Rikenellaceae* | 0.0292±0.0099 | 0.0739±0.0579 | 0.0320±0.0033 | 0.0306±0.0079 |
| *Prevotellaceae* | 0.0289±0.0183 | 0.0199±0.0265 | 0.0178±0.0003 | 0.0114±0.0027 |
| *Bacteroidaceae* | 0.0463±0.0147 | 0.0082±0.0034 | 0.0093±0.0009 | 0.0087±0.0027 |
| *Marinifilaceae* | 0.0012±0.0006 | 0.0539±0.0391 | 0.0022±0.0004 | 0.0059±0.0021 |
| *Ruminococcaceae* | 0.0335±0.0129 | 0.0116±0.0063 | 0.0062±0.0016 | 0.0090±0.0030 |
| *Helicobacteraceae* | 0.0048±0.0060 | 0.0467±0.0799 | 0.0006±0.0003 | 0.0022±0.0006 |

Table S1.2:

**Table S1.2. The top ten abundance of gut microbiota at the genus level**

| Flora | Group | | | |
| --- | --- | --- | --- | --- |
|  | Control | MPTP | Low-dose OPA | High-dose OPA |
| *Lactobacillus* | 0.0457±0.0081 | 0.1773±0.0478 | 0.0468±0.0115 | 0.1755±0.0983 |
| *Lachnospiraceae NK4A136 group* | 0.1296±0.0251 | 0.0662±0.0254 | 0.1365±0.0033 | 0.0743±0.0216 |
| *Catenibacterium* | 0.1683±0.0855 | 0.0031±0.0043 | 0.1560±0.0358 | 0.0244±0.0385 |
| *Allobaculum* | 0.0013±0.0007 | 0.0828±0.1424 | 0.0170±0.0035 | 0.0666±0.0570 |
| *Alistipes* | 0.0168±0.0168 | 0.0493±0.0402 | 0.0084±0.0024 | 0.0213±0.0044 |
| *Bacteroides* | 0.0463±0.0147 | 0.0082±0.0034 | 0.0093±0.0009 | 0.0087±0.0027 |
| *Odoribacter* | 0.0011±0.0006 | 0.0534±0.0749 | 0.0021±0.0003 | 0.0058±0.0022 |
| *Helicobacter* | 0.0048±0.0060 | 0.0467±0.0799 | 0.0006±0.0003 | 0.0022±0.0006 |
| *Staphylococcus* | 0.0008±0.0003 | 0.0406±0.0483 | 0.0018±0.0004 | 0.0060±0.0051 |
| *Prevotellaceae UGG-001* | 0.0089±0.0138 | 0.0175±0.0256 | 0.0081±0.0007 | 0.0060±0.0014 |
