# Supplementary material for: Neuroprotective Effects of Oligosaccharides From Periplaneta Americana on Parkinson’s Disease Models In Vitro and In Vivo
Source: Front Pharmacol. 2022 Jul 18;13:936818. doi: 10.3389/fphar.2022.936818 (PMC9340460; doi:10.3389/fphar.2022.936818)
Supplement: Supplementary file 2 [file DataSheet1.zip › 16S rRNA/Images/lefse_cladogram.pdf]

Cladogram

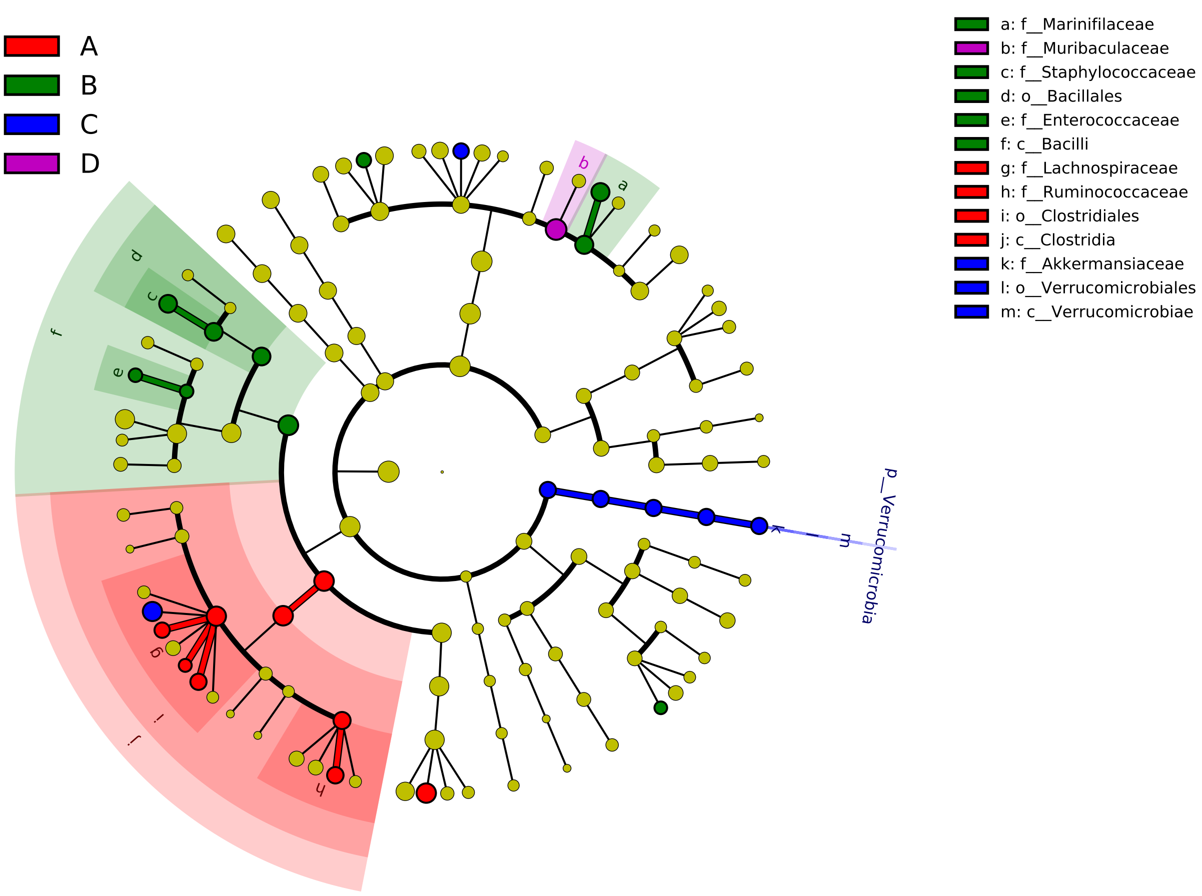

A
B
C
D
a: f__Marinifilaceae
b: f__Muribaculaceae
c: f__Staphylococcaceae
d: o__Bacillales
e: f__Enterococcaceae
f: c__Bacilli
g: f__Lachnospiraceae
h: f__Ruminococcaceae
i: o__Clostridiales
j: c__Clostridia
k: f__Akkermansiaceae
l: o__Verrucomicrobiales
m: c__Verrucomicrobiae
p__Verrucomicrobia
