# Supplementary material for: Neuroprotective Effects of Oligosaccharides From Periplaneta Americana on Parkinson’s Disease Models In Vitro and In Vivo
Source: Front Pharmacol. 2022 Jul 18;13:936818. doi: 10.3389/fphar.2022.936818 (PMC9340460; doi:10.3389/fphar.2022.936818)
Supplement: Supplementary file 2 [file DataSheet1.zip › 16S rRNA/Images/krona.html]

Javascript must be enabled to view this page.

magnitude
magnitudeUnassigned

C\_1
C\_2
C\_3
N\_1
N\_2
N\_3
L\_1
L\_2
L\_3
H\_1
H\_2
H\_3
H\_4

99.999999799999899.999999889999999.999999859999999.999999849999999.999999839999899.999999849999999.999999839999899.999999799999899.999999819999899.999999769999999.999999879999999.999999859999999.9999998499999

99.999999799999899.996498979999999.996498949999999.985996209999999.982495289999899.999999849999999.982495289999899.992997979999899.989497089999899.999999769999999.999999879999999.999999859999999.9999998499999

0.007001820.007001820.00350091

0.007001820.007001820.00350091

0.00700182

0.00700182

0.00700182

0.007001820.00350091

0.007001820.00350091

0.007001820.00350091

0.007001820.00350091

0.00350091

0.00350091

0.00350091

0.00350091

0.00350091

0.00350091

48.0850020557.9190589255.4964290335.7898053161.1538999749.1072678660.2191569355.4719226362.2846939458.4266908763.1249124464.7423329657.75801703

48.0850020557.9190589255.4964290335.7898053161.1538999749.1072678660.2191569355.4719226362.2846939458.4266908763.1249124464.7423329657.75801703

0.017504550.017504550.017504550.017504550.017504550.017504550.003500910.028007280.00350091

0.017504550.017504550.017504550.017504550.010502730.003500910.010502730.00350091
0.017504550.017504550.017504550.017504550.017504550.017504550.003500910.028007280.00350091

0.01750455

0.01750455

0.01050273

0.01050273

0.00700182

0.00700182

0.00700182

0.00700182

0.007001820.007001820.003500910.007001820.003500910.003500910.007001820.0350091
47.997479357.8980534655.4614199335.7793025861.1223917849.0967651360.1771460155.4404144462.2321802958.367175463.1249124464.7248284157.75451612

0.003500910.003500910.010502730.010502730.010502730.01050273
0.003500910.003500910.003500910.00350091

0.010502730.007001820.007001820.00700182

0.00350091

0.003500910.007001820.003500910.003500910.02800728

0.07001820.10152640.189049150.878728477.786024377.512953370.238061890.168043690.255566450.353591930.826214810.462120150.68967931
0.010502730.00350091

0.056014560.10152640.178546420.843719377.673995247.4884470.224058250.161041870.234560990.34308920.815712080.455118330.68967931

0.021005460.077020030.290575550.245063720.003500910.007001820.007001820.045511830.017504550.04201092

0.056014560.10152640.157540960.766699347.383419697.243383280.220557340.154040050.231060080.336087380.770200250.437613780.64766839

0.00350091

0.014003640.010502730.03500910.10152640.021005460.014003640.007001820.021005460.010502730.010502730.00700182

0.010502730.028007280.10152640.003500910.010502730.007001820.021005460.010502730.01050273

0.010502730.00700182

0.003500910.017504550.003500910.00700182

0.003500910.028007280.049012740.014003640.003500910.021005460.042010920.021005460.03500910.056014560.038510010.007001820.04201092

0.014003640.045511830.003500910.003500910.021005460.038510010.017504550.028007280.045511830.038510010.003500910.04201092
0.014003640.045511830.003500910.003500910.014003640.031508190.017504550.024506370.045511830.038510010.003500910.04201092

0.007001820.007001820.00350091

0.003500910.014003640.003500910.010502730.003500910.003500910.007001820.010502730.00350091

0.003500910.014003640.003500910.010502730.003500910.003500910.007001820.010502730.00350091

0.00350091

0.007001820.007001820.003500910.003500910.003500910.003500910.00700182
0.007001820.007001820.003500910.003500910.003500910.010502730.003500910.003500910.00700182

0.003500910.01050273

3.049292810.983755774.635205150.598655645.041310730.325584641.809970581.764458741.75045510.798207521.144797641.466881381.13779581
0.00350091

0.007001820.007001820.007001820.007001820.003500910.00350091

0.007001820.010502730.024506370.007001820.03500910.003500910.031508190.024506370.003500910.007001820.010502730.024506370.00700182

0.021005460.003500910.010502730.014003640.003500910.007001820.01750455

0.003500910.017504550.00700182

0.00350091

0.003500910.007001820.003500910.00700182

0.007001820.007001820.003500910.003500910.00350091

0.010502730.007001820.003500910.00700182

0.007001820.00700182

0.03500910.031508190.003500910.003500910.059515470.014003640.045511830.049012740.063016380.042010920.042010920.045511830.05601456
0.03500910.024506370.003500910.003500910.056014560.007001820.038510010.024506370.063016380.042010920.031508190.042010920.03851001

0.00700182

0.003500910.007001820.00700182

0.007001820.024506370.010502730.003500910.00700182

0.00350091

0.003500910.00700182

0.003500910.00700182

0.031508190.003500910.003500910.007001820.045511830.028007280.021005460.03500910.007001820.007001820.04551183

0.112029130.063016382.482145360.353591934.708724270.196050970.892732110.78770480.763198430.567147460.507631980.51463380.79820753

0.003500910.007001820.003500910.028007280.007001820.017504550.063016380.014003640.017504550.003500910.014003640.00700182

0.045511830.042010920.087522760.024506370.021005460.038510010.059515470.038510010.042010920.056014560.094524580.672174770.03851001

0.672174770.37459740.626662930.189049150.115530040.042010920.672174770.759697520.756196610.07001820.112029130.185548240.15754096

0.007001820.01050273

0.038510010.028007280.003500910.003500910.010502730.021005460.010502730.045511830.031508190.003500910.003500910.01400364

0.028007280.003500910.003500910.010502730.010502730.010502730.010502730.003500910.01400364

0.007001820.024506370.007001820.028007280.00350091

0.003500910.003500910.010502730.028007280.00350091

0.007001820.003500910.014003640.007001820.003500910.00350091

2.08654250.458619241.347850440.007001820.003500910.014003640.03500910.007001820.014003640.36759557

0.00350091

0.007001820.003500910.010502730.007001820.00350091

0.007001820.010502730.007001820.00350091

0.007001820.010502730.007001820.00350091

0.007001820.010502730.007001820.00350091

38.4539980446.1174905541.485786332.1033468732.7895252832.4709424553.1998319649.1772860955.0308080153.1648228556.805769557.7720207350.57064837
38.6710544746.1489987441.5593054132.1313541532.7930261932.4849460953.248844749.2648088555.0833216653.2313401456.9808150157.898053550.61966111

0.217056430.031508190.073519110.028007280.003500910.014003640.049012740.087522760.052513650.066517290.175045510.126032770.04901274

0.217056430.031508190.073519110.028007280.003500910.014003640.049012740.087522760.052513650.066517290.175045510.126032770.04901274

1.207814020.847220270.910236660.178546421.246324030.189049150.462120150.357092840.472622880.322083740.381599211.1307940.29057555

0.01400364

0.01400364

1.197311290.847220270.910236660.178546421.232320390.185548240.462120150.357092840.458619240.322083740.371096481.1307940.28357373

0.003500910.003500910.021005460.024506370.024506370.014003640.003500910.028007280.00350091

0.003500910.017504550.01050273

0.010502730.045511830.010502730.003500910.010502730.007001820.010502730.028007280.01050273

0.003500910.00700182

0.003500910.003500910.00350091

0.941744850.560145640.287074640.080520941.053773980.154040050.147038230.080520940.161041870.17154460.203052790.290575550.14003641

0.01050273

0.028007280.028007280.014003640.017504550.059515470.007001820.038510010.024506370.028007280.017504550.014003640.038510010.01750455

0.017504550.017504550.07001820.007001820.003500910.038510010.00350091

0.010502730.014003640.00700182

0.192550060.182047330.549642910.059515470.038510010.021005460.227559170.199551880.241562810.098025490.108528220.749194790.1015264

0.014003640.010502730.003500910.010502730.01050273

0.010502730.003500910.014003640.010502730.00700182

0.007001820.003500910.003500910.00350091

0.00350091

0.003500910.010502730.007001820.00350091

3.101806476.02506654.761237920.465621061.148298540.840218450.896233010.868225721.03626940.787704790.759697521.263828580.66867384

3.101806476.02506654.761237920.465621061.148298540.840218450.896233010.868225721.03626940.787704790.759697521.263828580.66867384

0.007001820.00350091

0.234560990.154040050.892732110.014003640.367595570.017504550.031508190.038510010.059515470.045511830.049012740.021005460.02800728

0.007001820.066517290.010502730.00350091

0.010502730.00350091

0.00350091

0.02100546

0.00350091

0.108528220.476123790.052513650.10152640.143537320.059515470.07001820.059515470.213555520.248564630.269570090.03851001

0.010502730.010502730.007001820.007001820.003500910.014003640.014003640.01050273

0.03500910.003500910.007001820.014003640.017504550.00350091

0.00700182

0.00700182

0.245063720.164542780.378098310.10152640.269570090.185548240.087522760.105027310.07001820.234560990.150539140.329085560.1715446

0.007001820.003500910.007001820.014003640.003500910.017504550.066517290.00700182

0.007001820.003500910.007001820.00350091

0.017504550.010502730.007001820.021005460.010502730.024506370.003500910.00350091

0.003500910.007001820.021005460.003500910.038510010.007001820.021005460.010502730.031508190.010502730.017504550.010502730.01750455

0.325584650.185548241.004761240.059515470.052513650.024506370.080520940.066517290.059515470.03500910.028007280.017504550.04901274

0.003500910.031508190.014003640.003500910.00700182

0.024506370.07001820.056014560.024506370.021005460.03500910.045511830.042010920.03500910.024506370.014003640.007001820.01050273

0.003500910.01400364

0.007001820.00350091

2.100546144.929281612.300098030.245063720.175045510.413107410.504131070.486626520.658171120.189049150.252065540.507631980.33258647

1.799467853.654950293.311861071.4703822813.047892457.652989773.322363812.821733643.455398392.730709972.888250942.415628054.20109228
0.500630160.570648370.549642910.455118332.079540681.550903231.274331330.903234841.169304020.679176590.588152920.199551881.14129674

0.010502730.021005460.259067360.556644730.007001820.003500910.021005460.028007280.021005460.028007280.01400364

0.010502730.021005460.259067360.556644730.007001820.003500910.021005460.028007280.021005460.028007280.01400364

0.007001820.00350091

0.003500910.00350091

0.003500910.00350091

0.00350091

0.220557341.50539140.360593750.192550061.865985160.402604681.158801291.323344071.207814030.311581010.147038230.262568270.3080801

1.078280351.568407792.401624410.801708438.843299255.142837130.871726640.5846521.053773971.711945092.132054331.925500622.7377118

0.084021850.014003640.059515470.03500910.010502730.014003640.007001820.014003640.010502730.010502730.007001820.00350091

0.112029130.098025490.115530040.168043691.662932361.393362270.20655370.154040050.259067360.360593750.906735750.161041871.06777762

0.00350091

0.007001820.01050273

0.03500910.150539140.056014560.028007280.017504550.003500910.049012740.007001820.045511830.038510010.003500910.01400364

0.049012740.031508190.014003640.003500910.003500910.024506370.007001820.01400364

0.010502730.003500910.021005460.056014560.10152640.014003640.038510010.024506370.031508190.007001820.010502730.049012740.01750455

0.00700182

0.332586471.267329511.102786720.511132897.022825933.724968490.5461420.367595570.665172941.211314941.200812211.666433271.6419269

0.455118330.003500911.032768520.021005460.007001820.007001820.024506370.010502730.066517290.014003640.00700182

0.049012740.101526390.024506370.031508190.024506370.07001820.122531850.136535490.10502730.066517290.077020020.038510010.03851001

0.049012740.101526390.024506370.031508190.024506370.07001820.122531850.136535490.10502730.066517290.077020020.038510010.03851001

0.007001820.014003640.003500910.007001820.021005460.010502730.042010920.017504550.010502730.003500910.02100546

0.028007280.049012740.021005460.031508190.010502730.03500910.063016380.063016380.03500910.038510010.042010920.021005460.01050273

0.01400364

0.014003640.038510010.003500910.010502730.028007280.038510010.049012740.028007280.010502730.024506370.014003640.00700182

0.021005460.003500910.021005460.00700182

0.007001820.007001820.003500910.003500910.007001820.003500910.01750455

0.017504550.007001820.00700182

0.010502730.00700182

0.010502730.00700182

0.010502730.00700182

0.00700182

0.00700182

0.00700182

0.00700182

0.007001820.00350091

0.038510010.003500910.017504550.003500910.007001820.007001820.010502730.017504550.007001820.01050273

0.031508190.003500910.017504550.003500910.007001820.003500910.00700182

0.010502730.003500910.017504550.007001820.00700182

0.00350091

0.00700182

0.00700182

0.003500910.017504550.00700182

0.021005460.003500910.00350091

0.01750455

0.00350091

0.00350091

0.00350091

0.00350091

0.01050273

0.007001820.007001820.010502730.01050273

0.014003640.010502730.010502730.031508190.024506370.00700182

0.014003640.010502730.010502730.031508190.024506370.00700182

0.014003640.010502730.010502730.031508190.017504550.00700182

0.010502730.010502730.01400364

0.00350091

0.007001820.00350091

0.010502730.003500910.021005460.00700182

0.00700182

0.00700182

0.014003640.010502730.003500910.00350091

0.014003640.010502730.003500910.00350091

0.010502730.003500910.00350091
0.014003640.003500910.00350091

0.00350091

0.01050273

0.01050273

0.01050273

0.01050273

0.003500910.010502730.045511830.980254871.452877750.721187510.199551880.007001820.150539140.32208374

0.003500910.010502730.045511830.980254871.452877750.721187510.199551880.007001820.150539140.32208374

0.003500910.010502730.045511830.976753961.452877750.721187510.199551880.007001820.150539140.32208374

0.003500910.010502730.045511830.973253051.452877750.710684780.199551880.007001820.150539140.32208374

0.003500910.010502730.045511830.973253051.452877750.710684780.199551880.007001820.150539140.32208374

0.003500910.010502730.045511830.973253051.452877750.710684780.199551880.007001820.150539140.32208374

0.003500910.01050273

0.00350091

0.00350091

0.00350091

0.031508190.003500910.017504550.003500910.007001820.003500910.00350091

0.031508190.003500910.017504550.003500910.007001820.003500910.00350091

0.031508190.003500910.017504550.003500910.007001820.003500910.00350091

0.031508190.003500910.017504550.003500910.007001820.003500910.00350091

0.031508190.003500910.017504550.003500910.007001820.003500910.00350091

1.092283990.273070980.777202072.391121680.283573720.325584630.339588270.234560970.28007280.301078260.17504550.234560970.23106006

0.00350091

0.182047320.14003640.084021842.219577080.185548240.150539130.164542770.129533670.157540950.133034580.098025480.129533670.09102366

0.010502730.003500910.00700182

0.010502730.003500910.00700182

0.010502730.003500910.00700182

0.010502730.003500910.00700182

0.038510010.03500910.03500910.021005460.031508190.014003640.038510010.028007280.052513650.049012740.010502730.003500910.0350091

0.038510010.03500910.03500910.021005460.031508190.014003640.038510010.028007280.052513650.049012740.010502730.003500910.0350091

0.028007280.03500910.024506370.021005460.031508190.014003640.03500910.017504550.031508190.024506370.010502730.003500910.02800728

0.024506370.017504550.010502730.003500910.007001820.010502730.003500910.003500910.00700182

0.017504550.003500910.007001820.007001820.003500910.01050273

0.003500910.021005460.014003640.014003640.003500910.007001820.01050273

0.021005460.010502730.00350091

0.007001820.01050273

0.003500910.007001820.003500910.007001820.01050273

0.003500910.00350091

0.00350091

0.010502730.010502730.003500910.010502730.021005460.024506370.00700182

0.045511830.042010920.028007280.021005460.119030950.052513650.028007280.017504550.024506370.021005460.014003640.045511830.01050273

0.045511830.042010920.028007280.021005460.112029130.052513650.028007280.007001820.024506370.017504550.014003640.045511830.01050273

0.00700182

0.00700182

0.00700182

0.010502730.00350091

0.010502730.00350091

0.010502730.00350091

0.00350091

0.00350091

0.00350091

0.00350091

0.028007280.038510010.014003642.139056150.042010920.042010920.042010920.03500910.042010920.021005460.045511830.04201092

0.007001820.010502730.010502732.069037950.007001820.007001820.024506370.010502730.02800728
0.028007280.038510010.014003642.139056150.042010920.042010920.042010920.03500910.042010920.021005460.045511830.04201092

0.021005460.028007280.003500910.066517290.03500910.03500910.017504550.028007280.042010920.021005460.03500910.01400364

0.021005460.028007280.003500910.066517290.03500910.03500910.017504550.028007280.042010920.021005460.03500910.01400364

0.00350091

0.00350091

0.00700182

0.00700182

0.00350091

0.00350091

0.00350091

0.00350091

0.059515470.021005460.007001820.038510010.03500910.042010920.056014560.038510010.045511830.010502730.052513650.03500910.00350091

0.059515470.021005460.007001820.038510010.028007280.042010920.056014560.038510010.045511830.010502730.052513650.03500910.00350091

0.059515470.021005460.007001820.024506370.028007280.042010920.056014560.038510010.045511830.010502730.052513650.03500910.00350091

0.007001820.003500910.01050273

0.031508190.014003640.007001820.021005460.014003640.024506370.010502730.024506370.010502730.0350091

0.003500910.003500910.007001820.00700182

0.010502730.007001820.003500910.014003640.028007280.024506370.017504550.010502730.003500910.021005460.00350091

0.017504550.010502730.007001820.01050273

0.01400364

0.01400364

0.00700182

0.00700182

0.00700182

0.906735760.133034580.693180230.17154460.094524570.17504550.17504550.10502730.122531850.168043680.077020020.10502730.1400364

0.010502730.003500910.007001820.003500910.021005460.007001820.01050273
0.906735760.133034580.693180230.17154460.094524570.17504550.17504550.10502730.122531850.168043680.077020020.10502730.1400364

0.175045510.098025490.10152640.007001820.010502730.017504550.010502730.021005460.003500910.056014560.02100546
0.01400364

0.161041870.098025490.098025490.007001820.010502730.014003640.003500910.017504550.003500910.056014560.02100546

0.003500910.003500910.007001820.00350091

0.003500910.003500910.007001820.00350091

0.003500910.010502730.003500910.00350091
0.717686610.129533670.584652010.063016380.084021840.136535490.14003640.091023660.101526390.143537310.063016380.049012740.11903094

0.080520940.021005460.007001820.007001820.010502730.028007280.024506370.031508190.031508190.014003640.021005460.0350091

0.014003640.007001820.007001820.003500910.003500910.007001820.021005460.003500910.00350091

0.161041870.042010920.133034590.017504550.063016380.052513650.017504550.021005460.017504550.031508190.042010920.007001820.02100546

0.13653550.024506370.129533680.014003640.052513650.017504550.021005460.017504550.031508190.042010920.007001820.02100546

0.010502730.017504550.003500910.003500910.06301638

0.01400364

0.37459740.042010920.395602860.024506370.028007280.07001820.024506370.031508190.03500910.007001820.03150819

0.37459740.042010920.395602860.024506370.028007280.07001820.024506370.031508190.03500910.007001820.03150819

0.021005460.014003640.017504550.003500910.003500910.007001820.00700182

0.014003640.00350091

0.007001820.014003640.017504550.003500910.007001820.00700182

0.091023670.003500910.031508190.007001820.007001820.021005460.007001820.014003640.010502730.024506370.007001820.003500910.01750455

0.01050273

0.003500910.003500910.003500910.003500910.007001820.010502730.003500910.02450637

0.003500910.003500910.003500910.003500910.007001820.010502730.003500910.02450637

0.003500910.003500910.003500910.003500910.007001820.010502730.003500910.02450637

0.00350091

0.00350091

0.007001820.140036410.007001828.353171820.007001820.010502730.017504550.01050273

0.007001820.140036410.007001828.353171820.007001820.010502730.017504550.01050273

0.007001820.140036410.007001828.353171820.007001820.010502730.017504550.01050273

0.007001820.140036410.007001828.353171820.007001820.010502730.017504550.01050273

0.007001820.140036410.007001828.353171820.007001820.010502730.017504550.01050273

0.007001820.003500910.07001820.007001820.010502730.01050273

0.140036410.003500918.283153620.007001820.01050273

0.20655370.003500910.049012740.014003640.017504550.014003640.017504550.014003640.00350091

0.20655370.003500910.049012740.014003640.017504550.014003640.017504550.014003640.00350091

0.20655370.003500910.049012740.014003640.017504550.014003640.017504550.014003640.00350091

0.007001820.00350091
0.20655370.003500910.049012740.014003640.017504550.014003640.017504550.014003640.00350091

0.20655370.003500910.049012740.014003640.017504550.007001820.014003640.014003640.00350091

0.052513650.038510010.010502730.010502730.087522750.031508190.059515470.066517290.03500910.056014560.03500910.017504550.01400364

0.052513650.038510010.010502730.010502730.087522750.031508190.059515470.066517290.03500910.056014560.03500910.017504550.01400364

0.052513650.038510010.010502730.010502730.087522750.031508190.059515470.066517290.03500910.056014560.03500910.017504550.01400364

0.017504550.017504550.007001820.045511830.010502730.021005460.021005460.010502730.017504550.010502730.01050273

0.017504550.017504550.007001820.045511830.010502730.021005460.021005460.010502730.017504550.010502730.01050273

0.00350091

0.003500910.003500910.010502730.003500910.00350091

0.003500910.00350091

0.00350091

0.01750455

0.007001820.003500910.003500910.00700182

0.010502730.017504550.003500910.045511830.003500910.003500910.007001820.017504550.00350091

0.03500910.021005460.003500910.010502730.042010920.021005460.038510010.045511830.024506370.038510010.024506370.007001820.01400364

0.03500910.021005460.003500910.010502730.042010920.021005460.038510010.045511830.024506370.038510010.024506370.007001820.01400364

0.03500910.021005460.003500910.010502730.042010920.021005460.038510010.045511830.024506370.038510010.024506370.007001820.01400364

0.01050273
48.9707322841.0201652140.824114261.1293935737.407225827.1810670137.4002240141.4857862235.9963589940.151939435.9858562733.9728328840.89763333

17.8406384922.6298837524.152779689.1933902611.4969891810.2996778915.4600195815.3829995515.277972269.844559546.8477803911.024366318.99033746
0.010502730.010502730.010502730.00700182

17.8406384922.6298837524.142276959.1828875311.4969891810.2996778915.4495168515.3829995515.270970449.837557726.8477803911.024366318.99033746

0.007001820.014003640.007001820.007001820.007001820.00700182

0.007001820.007001820.00700182

0.007001820.007001820.00700182

0.007001820.007001820.00700182

0.007001820.007001820.00700182

0.00700182

0.00700182

0.00700182

0.00700182

0.021005460.003500910.108528220.091023670.857723010.115530040.014003640.007001820.021005460.031508190.045511830.03500910.02450637

0.00350091

0.00350091

0.00350091

0.042010920.003500910.073519110.042010920.259067360.014003640.014003640.014003640.007001820.007001820.02450637

1.417868650.045511835.475423610.10152640.371096493.364374740.185548240.129533680.23806190.07001820.217056430.108528220.04901274
12.7853241619.3635345121.54810258.202632689.553984027.9050553114.605797514.7703402814.343229248.888811075.3213835510.208654258.01708443

0.024506370.014003640.003500910.02100546

0.01050273

0.014003640.00350091

0.010502730.003500910.02100546

0.010502730.010502730.01050273

0.010502730.010502730.01050273

0.007001820.010502730.03500910.003500910.014003640.04901274

0.024506370.007001820.021005460.003500910.014003640.003500910.01050273

0.339588291.144797650.010502730.294076460.080520940.007001820.028007280.010502730.038510010.00700182

0.03500910.003500910.007001820.028007280.014003640.007001820.003500910.010502730.003500910.03150819

0.059515470.038510010.038510010.00350091

0.175045512.926760960.406105590.014003640.024506370.493628340.504131070.556644730.066517290.007001820.234560990.02100546

0.010502730.00350091

0.010502730.00350091

0.003500910.00700182

0.014003640.024506370.007001820.00350091

0.007001820.00350091

0.024506370.010502730.028007280.007001820.007001820.00700182

0.024506370.010502730.028007280.007001820.007001820.00700182

0.03500910.028007280.017504550.010502730.017504550.038510010.042010920.017504550.017504550.031508190.014003640.00700182

0.217056431.316342250.437613780.150539140.336087380.23806190.129533680.080520940.17154460.315081920.574149280.367595570.36059375

0.014003640.017504550.014003640.03500910.07001820.010502730.028007280.007001820.014003640.063016380.003500910.01050273

0.017504550.00700182

0.00350091

0.00350091

0.059515470.010502730.00350091

0.010502730.010502730.014003640.00700182

10.1526396914.980394913.758577237.887550768.286654533.6969612113.7025626713.9511272913.296457088.335667274.414647819.452457647.53395883

0.010502730.003500910.010502730.028007280.003500910.007001820.00700182

0.129533680.003500910.119030950.024506370.03500910.098025490.007001820.003500910.010502730.014003640.007001820.028007280.00350091

0.007001820.168043690.003500910.00350091

0.007001820.00350091

0.00350091

0.00700182

0.00350091

0.00350091

0.021005460.007001820.01050273

0.021005460.007001820.01050273

0.021005460.007001820.01050273

0.007001820.00700182
0.101526390.003500910.063016380.003500910.045511830.007001820.028007280.028007280.028007280.010502730.021005460.017504550.01750455

0.007001820.01050273

0.014003640.021005460.014003640.00350091

0.038510010.059515470.007001820.010502730.007001820.007001820.01400364

0.03500910.007001820.010502730.007001820.010502730.00700182

0.007001820.003500910.003500910.003500910.010502730.003500910.010502730.010502730.007001820.007001820.00350091

0.329085560.052513650.542641090.13653550.185548240.51463380.056014560.014003640.024506370.140036410.052513650.031508190.06301638
4.747234253.098305552.202072520.801708430.798207521.893992420.651169290.44811650.75269570.745693871.344349520.693180210.80871026

0.021005460.010502730.03500910.003500910.01400364

0.007001820.024506370.00350091

0.007001820.003500910.059515470.017504550.007001820.003500910.007001820.0350091

0.063016380.056014560.003500910.059515470.010502730.028007280.00350091

0.063016380.056014560.003500910.059515470.010502730.028007280.00350091

0.049012740.003500910.021005460.010502730.073519110.007001820.007001820.007001820.003500910.017504550.02450637

0.00350091

0.17154460.007001820.063016380.003500910.038510010.133034590.007001820.031508190.017504550.00350091

0.952247580.049012740.679176590.031508190.283573730.605657470.056014560.045511830.077020030.045511830.13653550.07001820.0350091

0.007001820.007001820.010502730.003500910.007001820.014003640.00350091

0.024506371.088783080.038510010.038510010.014003640.024506370.03150819

0.00700182

0.00700182

0.010502730.073519110.014003640.031508190.087522760.010502730.003500910.003500910.017504550.007001820.01750455

0.01400364

0.028007280.010502730.007001820.049012740.014003640.007001820.042010920.007001820.007001820.02100546

0.066517290.003500910.010502730.014003640.042010920.007001820.003500910.010502730.03150819

0.003500910.007001820.07001820.010502730.003500910.084021850.066517290.091023670.017504550.00350091

0.007001820.003500910.007001820.010502730.007001820.010502730.010502730.003500910.003500910.01400364

3.014283711.872986980.661672030.51463380.112029130.297577370.367595570.266069180.399103770.462120151.039770340.476123790.67217477

0.059515470.03500910.07001820.042010920.045511830.031508190.052513650.028007280.052513650.07001820.028007280.045511830.0350091
0.052513650.028007280.052513650.038510010.03500910.028007280.03500910.028007280.038510010.056014560.021005460.042010920.0350091

0.003500910.003500910.003500910.003500910.014003640.007001820.00350091

0.003500910.003500910.003500910.003500910.014003640.007001820.00350091

0.007001820.003500910.003500910.010502730.014003640.01050273

0.007001820.003500910.003500910.010502730.014003640.01050273

0.007001820.01050273

0.007001820.01050273

0.059515470.094524580.07001820.03500910.108528220.084021840.084021850.07001820.045511830.056014560.073519110.017504550.05251365

0.059515470.094524580.07001820.03500910.105027310.084021840.084021850.07001820.045511830.052513650.066517290.017504550.05251365
0.059515470.080520940.066517290.03500910.094524580.07001820.084021850.07001820.045511830.052513650.049012740.017504550.05251365

0.014003640.003500910.010502730.014003640.01750455

0.00350091

0.00700182

0.00350091

0.00350091

0.017504550.007001820.007001820.024506370.003500910.007001820.010502730.014003640.01050273

0.017504550.007001820.007001820.024506370.003500910.007001820.010502730.014003640.01050273

0.00700182

4.257106824.712225175.7695000524.5168743525.6511692816.59781545.394902663.959529446.3471502115.5300377728.780983057.0648368322.95196749

0.10502730.133034590.066517299.634504961.841478780.847220270.266069180.17504550.252065520.717686590.290575551.319843160.23456098

0.01050273

0.01050273

0.01050273

0.024506370.028007280.017504550.010502730.017504550.024506370.03500910.024506370.03500910.014003640.038510010.031508190.05251365

0.024506370.028007280.017504550.010502730.017504550.024506370.03500910.024506370.03500910.014003640.038510010.031508190.05251365

0.003500910.010502730.003500910.007001820.01400364
0.003500910.00350091

0.003500910.010502730.003500910.01400364

0.00350091

0.01400364

0.01050273

0.00350091

0.080520930.10152640.049012749.624002231.823974230.812211170.227559170.150539130.199551870.689679310.252065541.288334970.18204733
0.007001820.007001820.010502730.007001820.003500910.00350091

0.017504550.028007280.038510010.00700182

0.00700182

0.017504550.028007280.03851001

0.080520930.10152640.049012749.606497681.788965130.773701160.213555530.14003640.199551870.682677490.248564631.284834060.18204733

0.003500910.003500910.007001820.03500910.0350091

0.003500910.007001820.010502730.00350091

0.140036410.13653550.007001820.003500910.003500910.007001820.010502730.007001820.021005460.00350091

0.052513650.003500910.014003640.00350091

0.03500910.007001820.017504550.413107410.049012740.056014560.024506370.028007280.052513650.03500910.028007280.161041870.00700182

0.003500910.059515470.073519110.003500910.01050273

0.017504550.084021850.024506377.064836860.37459740.094524580.091023670.063016380.07001820.276571910.119030950.68267750.08402185

0.010502730.007001820.017504550.00700182

0.021005460.00350091

0.007001820.00350091

0.00700182

0.028007280.007001820.007001821.837977871.141296740.609158380.080520940.031508190.059515470.290575550.091023670.399103770.05251365

4.152079524.579190585.7029827614.8823693923.809690515.750595135.128833483.784483946.0950846914.8123511828.49040755.7449936722.71740651

0.084021840.217056430.13653550.094524570.301078270.133034580.126032760.199551870.161041860.196050970.182047330.189049140.21355551

0.024506370.010502730.007001820.007001820.003500910.003500910.01050273

0.024506370.010502730.007001820.007001820.003500910.003500910.01050273

0.084021840.192550060.126032770.094524570.294076450.133034580.119030940.196050960.161041860.192550060.182047330.189049140.20305278

0.024506370.017504550.014003640.007001820.049012740.014003640.021005460.042010920.03500910.014003640.03500910.017504550.05251365

0.00350091

0.007001820.007001820.017504550.007001820.007001820.003500910.003500910.021005460.01050273

0.010502730.007001820.017504550.010502730.056014560.007001820.056014560.010502730.07001820.003500910.042010920.02800728

0.010502730.010502730.01050273

0.010502730.003500910.007001820.010502730.014003640.007001820.014003640.014003640.01400364

0.007001820.063016380.003500910.003500910.00350091

0.01050273

0.024506370.007001820.007001820.003500910.007001820.010502730.014003640.003500910.007001820.042010920.02450637

0.042010920.126032770.084021850.049012740.10152640.049012740.038510010.07001820.052513650.10152640.087522760.063016380.07351911

0.003500910.028007280.010502730.00350091

0.007001820.028007280.01400364

0.003500910.007001820.00700182

0.056014560.038510010.021005460.063016380.080520930.028007280.028007280.017504550.031508190.038510010.021005460.049012740.02800728

0.056014560.038510010.021005460.063016380.080520930.028007280.028007280.017504550.031508190.038510010.021005460.049012740.02800728

0.007001820.003500910.007001820.007001820.01050273

0.024506370.003500910.014003640.014003640.003500910.003500910.014003640.00350091

0.031508190.031508190.014003640.049012740.066517290.024506370.021005460.014003640.024506370.024506370.021005460.03500910.02800728

0.01050273
0.031508190.017504550.010502730.021005460.028007280.031508190.014003640.017504550.017504550.010502730.003500910.01050273

0.031508190.017504550.010502730.021005460.028007280.031508190.014003640.017504550.007001820.010502730.003500910.01050273

0.031508190.014003640.010502730.003500910.003500910.031508190.003500910.010502730.007001820.003500910.00350091

0.003500910.017504550.024506370.010502730.007001820.010502730.00700182

0.014003640.056014560.045511830.10152640.168043690.115530040.133034590.080520930.119030950.087522750.140036410.052513650.14353732

0.014003640.056014560.045511830.10152640.168043690.115530040.133034590.080520930.119030950.087522750.140036410.052513650.14353732

0.003500910.052513650.03500910.094524580.143537320.087522760.115530040.059515470.098025490.052513650.084021850.052513650.11202913

0.007001820.003500910.01050273

0.01400364

0.003500910.010502730.00700182

0.007001820.014003640.017504550.003500910.01750455

0.003500910.003500910.007001820.007001820.028007280.031508190.038510010.02100546

0.007001820.00350091

0.00700182

0.00700182

0.00700182

0.014003640.03500910.010502730.007001820.021005460.01750455
0.007001820.007001820.007001820.021005460.01750455

0.007001820.028007280.003500910.00700182

0.007001820.028007280.003500910.00700182

3.966531294.250105035.4894272414.5812911223.2250385115.435513224.810250663.472902955.7659991414.4657610828.115810115.4334126822.32180367

0.007001820.028007280.014003640.003500910.017504550.010502730.007001820.08402185

0.024506370.00350091

0.007001820.003500910.014003640.017504550.010502730.007001820.08402185

3.966531294.250105035.4824254214.5532838423.2250385115.421509584.810250663.472902955.7624982314.4482565328.105307385.4264108622.23778182

0.03500910.017504550.024506370.03500910.073519110.017504550.049012740.031508190.045511830.031508190.042010920.017504550.05251365

0.00700182

0.00700182

0.23806190.210054610.420109231.323344071.256826770.847220280.283573730.325584650.2730710.304579190.836717550.262568270.31158101

0.00700182

0.371096490.388601040.37459741.627923263.640946651.071278530.23806190.108528220.332586470.276571910.934743030.388601040.39210195

0.003500910.084021850.03500910.119030950.16804369

0.014003640.010502730.038510010.010502730.00350091

0.007001820.00350091

0.01050273

0.010502730.003500910.003500910.010502730.007001820.007001820.007001820.010502730.014003640.007001820.0350091

0.010502730.024506370.007001820.042010922.261588011.897493352.5766699310.947346310.007001820.02450637

0.01050273

0.220557340.168043690.350091020.318582830.13653552.023526120.157540960.122531860.255566450.175045511.23232040.413107412.51715446

0.038510010.028007280.066517290.2065537

0.01400364

0.03150819

0.01400364

0.010502730.010502730.007001820.010502730.02100546

0.010502730.01400364

0.00700182

0.010502730.01400364

0.03500910.024506370.017504550.003500910.052513650.007001820.010502730.02100546

0.003500910.003500910.01050273

0.007001820.003500910.021005460.028007280.381599220.003500910.010502730.105027310.010502730.03851001

1.830976052.18806892.230079827.558465211.791065686.676235821.015263970.539140181.242823131.5228959516.520795412.9897773413.42599076

0.038510010.031508190.052513650.04901274

0.969752141.12729311.344349533.276851985.723988243.070298280.423610140.231060080.5461420.44461567.330906041.085282173.94902675

0.164542780.098025490.34308920.290575550.077020031.519395040.133034590.066517290.129533680.119030950.871726650.220557341.27083042

0.003500910.00350091

0.017504550.010502730.003500910.03500910.00700182

0.00350091

0.017504550.024506370.245063720.014003640.056014560.017504550.021005460.07001820.084021850.091023670.014003640.08752276

0.00350091

0.07001820.021005460.056014560.031508190.056014560.038510010.021005460.017504550.003500910.063016380.007001820.04901274

26.8659851513.6640526510.8738271927.387620770.178546410.2275591616.5277972222.1257526814.3572328814.728329350.3360873715.852121558.90631564

26.8659851513.6640526510.8738271927.387620770.178546410.2275591616.5277972222.1257526814.3572328814.728329350.3360873715.852121558.90631564

0.073519110.112029130.185548241.781963310.063016380.014003640.241562810.329085560.231060080.588152920.003500911.123792191.05377398
26.8659851513.6640526510.8738271927.387620770.178546410.2275591616.5277972222.1257526814.3572328814.728329350.3360873715.852121558.90631564

0.014003640.003500910.00700182

0.007001820.010502730.031508190.049012740.052513650.07001820.056014560.07001820.003500910.115530040.26256827

0.007001820.010502730.031508190.049012740.052513650.07001820.056014560.07001820.003500910.115530040.26256827

0.00350091

0.00350091

0.031508190.014003640.038510010.021005460.031508190.014003640.017504550.042010920.017504550.038510010.052513650.024506370.04551183

0.031508190.014003640.038510010.021005460.031508190.014003640.017504550.042010920.017504550.038510010.052513650.024506370.04551183

0.00350091

0.00350091

0.00350091

0.00700182

0.021005460.003500910.010502730.00350091

0.021005460.003500910.010502730.00350091

0.00700182

0.00700182

0.164542780.182047330.0525136524.730429910.056014560.059515471.648928722.072538861.386360455.822013720.0630163813.961630026.81277132

0.164542780.182047330.0525136524.730429910.056014560.059515471.648928722.072538861.386360455.822013720.0630163813.961630026.81277132

26.5684077913.3174625410.593754380.812211170.028007280.0910236714.5637865819.5910936812.655790518.202632680.213555520.626662930.73169024

0.00350091

0.007001820.00700182

0.007001820.014003640.028007280.031508190.080520930.056014560.017504550.017504550.014003640.038510010.021005460.031508190.04901274

0.007001820.014003640.028007280.031508190.080520930.056014560.017504550.017504550.014003640.038510010.021005460.031508190.04901274

0.014003640.028007280.021005460.056014560.038510010.014003640.017504550.010502730.03500910.021005460.031508190.03851001

0.017504550.010502730.003500910.00700182

0.017504550.010502730.003500910.00700182

0.010502730.00350091

0.010502730.00350091

0.014003640.010502730.010502730.045511830.028007280.007001820.017504550.010502730.03500910.017504550.021005460.03150819

0.014003640.010502730.010502730.038510010.028007280.007001820.017504550.010502730.031508190.010502730.021005460.03150819

0.007001820.003500910.00700182

0.003500910.00700182
0.010502730.007001820.003500910.00350091

0.007001820.003500910.00350091

0.007001820.010502730.024506370.017504550.003500910.003500910.003500910.01050273

0.007001820.010502730.024506370.017504550.003500910.003500910.003500910.01050273

0.007001820.010502730.024506370.017504550.003500910.003500910.003500910.01050273

0.01400364

0.01400364

0.01400364

0.01400364

0.01050273

0.01050273

0.01050273

0.028007280.010502730.010502730.038510010.014003640.007001820.014003640.01400364

0.007001820.007001820.003500910.00350091

0.007001820.007001820.00350091

0.00350091

0.00350091

0.00350091

0.00700182

0.007001820.00700182

0.010502730.003500910.017504550.003500910.007001820.01050273

0.017504550.00700182
0.010502730.017504550.007001820.01050273

0.01050273

0.01050273

0.01050273

0.01050273

0.01050273

0.003500910.00350091

0.003500910.00350091

0.003500910.00350091

0.01750455

0.003500910.010502730.003500910.00700182

0.077020030.014003640.017504550.017504550.003500910.150539140.066517290.077020030.028007280.007001820.007001820.007001820.02800728

0.077020030.014003640.017504550.017504550.003500910.150539140.066517290.077020030.028007280.007001820.007001820.007001820.02800728

0.077020030.014003640.017504550.017504550.003500910.150539140.066517290.077020030.028007280.007001820.007001820.007001820.02800728

0.252065530.052513651.179806740.056014560.0735191113.905615460.119030940.052513650.052513650.245063710.24156280.283573720.12953368

0.252065530.052513651.179806740.056014560.0735191113.905615460.119030940.052513650.052513650.245063710.24156280.283573720.12953368

0.252065530.052513651.179806740.056014560.0735191113.905615460.119030940.052513650.052513650.245063710.24156280.283573720.12953368

0.017504550.014003640.010502730.010502730.024506370.010502730.003500910.014003640.003500910.00700182

0.017504550.014003640.010502730.010502730.024506370.010502730.003500910.014003640.003500910.00700182

0.007001820.007001820.003500910.014003640.010502730.00700182

0.017504550.007001820.003500910.007001820.010502730.003500910.014003640.00350091

0.234560980.052513651.16580310.045511830.0735191113.895112730.094524570.042010920.049012740.231060070.238061890.27657190.12953368

0.234560980.052513651.16580310.045511830.0735191113.895112730.094524570.042010920.049012740.231060070.238061890.27657190.12953368

0.003500910.003500910.210054610.045511830.028007289.68001680.024506370.010502730.007001820.189049150.115530040.168043690.1015264

0.017504550.003500910.063016380.031508190.224058260.024506370.003500910.024506370.03500910.07001820.049012740.01050273

0.003500910.20305279

0.01400364

0.199551880.045511830.88923120.014003643.787984880.045511830.028007280.017504550.007001820.052513650.059515470.01750455

1.179806730.672174751.512393210.469121960.903234830.910236640.756196591.13779580.54964290.602156540.409606490.553143810.60215654

1.085282160.500630151.277832230.437613770.658171120.500630150.598655630.980254850.416608310.532138340.297577370.469121970.54264107

0.255566440.325584640.255566440.290575540.37809830.245063710.378098290.784203880.178546420.34659010.154040050.210054610.33258646

0.00350091

0.010502730.00350091

0.010502730.00350091

0.010502730.00350091

0.073519110.028007280.017504550.014003640.031508190.042010920.017504550.073519110.014003640.063016380.024506370.028007280.0700182

0.017504550.014003640.010502730.01400364

0.017504550.014003640.010502730.01400364

0.049012740.028007280.017504550.014003640.017504550.042010920.017504550.07001820.014003640.049012740.024506370.028007280.05601456

0.021005460.010502730.003500910.007001820.028007280.021005460.007001820.02450637

0.003500910.00350091

0.01750455

0.007001820.007001820.007001820.003500910.00350091

0.014003640.007001820.014003640.017504550.014003640.007001820.038510010.010502730.021005460.024506370.017504550.02800728

0.028007280.007001820.00350091

0.007001820.003500910.00350091

0.007001820.003500910.00350091

0.007001820.007001820.010502730.017504550.003500910.00350091

0.01050273

0.007001820.007001820.010502730.00350091

0.007001820.00350091

0.00700182

0.017504550.007001820.007001820.003500910.00350091
0.182047330.290575540.238061890.269570080.336087380.203052790.322083730.703682950.161041870.283573720.129533680.182047330.26256826

0.010502730.014003640.007001820.007001820.028007280.028007280.00700182

0.01400364

0.007001820.014003640.007001820.003500910.00350091

0.003500910.003500910.007001820.028007280.00350091

0.00700182

0.031508190.038510010.038510010.031508190.077020030.042010920.007001820.017504550.007001820.017504550.017504550.038510010.02450637

0.031508190.038510010.038510010.031508190.077020030.042010920.007001820.017504550.007001820.017504550.017504550.038510010.02450637

0.014003640.007001820.010502730.00700182

0.014003640.007001820.010502730.00700182

0.003500910.010502730.014003640.00700182

0.003500910.010502730.014003640.00700182

0.01400364

0.00350091

0.13653550.234560980.182047330.217056430.227559160.150539140.231060070.588152920.126032770.192550060.10152640.126032770.21005461

0.003500910.014003640.01050273

0.13653550.203052790.154040050.178546420.203052790.122531860.217056430.535639270.108528220.157540960.077020030.108528220.18204733

0.017504550.017504550.028007280.007001820.028007280.024506370.007001820.014003640.02100546

0.00700182

0.003500910.010502730.010502730.003500910.007001820.007001820.010502730.021005460.00700182

0.010502730.010502730.007001820.007001820.003500910.003500910.010502730.00700182

0.003500910.003500910.003500910.0350091

0.003500910.003500910.003500910.0350091

0.010502730.010502730.00350091

0.010502730.010502730.00350091

0.003500910.010502730.010502730.021005460.049012740.03500910.007001820.01400364

0.003500910.010502730.010502730.021005460.049012740.03500910.007001820.01400364

0.024506370.024506370.03500910.021005460.115530040.017504550.03500910.049012740.03500910.03500910.024506370.143537320.0350091

0.024506370.024506370.03500910.021005460.115530040.017504550.03500910.049012740.03500910.03500910.024506370.143537320.0350091

0.024506370.017504550.021005460.003500910.003500910.014003640.003500910.010502730.007001820.024506370.028007280.02450637

0.024506370.010502730.014003640.003500910.003500910.003500910.003500910.014003640.003500910.02450637

0.007001820.007001820.010502730.003500910.007001820.007001820.010502730.02450637

0.098025490.007001820.007001820.03500910.007001820.10502731

0.098025490.007001820.007001820.03500910.007001820.10502731

0.007001820.014003640.017504550.014003640.003500910.024506370.031508190.021005460.010502730.01050273

0.007001820.014003640.017504550.014003640.003500910.024506370.031508190.021005460.010502730.01050273

0.014003640.014003640.00350091

0.014003640.00700182

0.01400364

0.01400364

0.00700182

0.00700182

0.007001820.00350091

0.00700182

0.00700182

0.00350091

0.00350091

0.003500910.00350091

0.003500910.00350091

0.003500910.00350091

0.00350091

0.00350091

0.00350091

0.00350091

0.003500910.014003640.003500910.003500910.007001820.003500910.003500910.007001820.00350091

0.003500910.014003640.003500910.003500910.003500910.003500910.00700182

0.003500910.014003640.003500910.003500910.003500910.003500910.00700182

0.003500910.00700182

0.003500910.00350091

0.014003640.003500910.00350091

0.007001820.00350091

0.00700182

0.00700182

0.00350091

0.00350091

0.003500910.01050273

0.003500910.01050273

0.805209350.150539140.987256690.122531860.13653550.234560980.164542780.133034590.189049150.147038230.115530040.108528220.16804369

0.805209350.150539140.987256690.122531860.13653550.234560980.164542780.133034590.189049150.147038230.115530040.108528220.16804369

0.007001820.003500910.003500910.024506370.010502730.014003640.014003640.007001820.017504550.01750455

0.003500910.007001820.003500910.007001820.01750455

0.007001820.003500910.017504550.007001820.007001820.010502730.007001820.01750455

0.00350091

0.017504550.010502730.00700182

0.010502730.00700182

0.01750455

0.805209350.126032770.980254870.098025490.129533680.203052790.140036410.112029130.168043690.119030950.105027310.080520940.14353732

0.003500910.007001820.01050273

0.003500910.007001820.01050273

0.007001820.007001820.01400364

0.007001820.007001820.01400364

0.003500910.021005460.007001820.010502730.00700182

0.003500910.021005460.007001820.010502730.00700182

0.028007280.147038230.021005460.007001820.045511830.03500910.042010920.094524570.024506370.021005460.031508190.021005460.01050273

0.00700182

0.00700182

0.00700182

0.00700182

0.129533680.017504550.003500910.010502730.00350091

0.01050273

0.01050273

0.00350091
0.01050273

0.00700182

0.003500910.00700182

0.003500910.00700182
0.00700182

0.00350091

0.00350091

0.024506370.017504550.021005460.007001820.003500910.03500910.042010920.091023660.024506370.021005460.021005460.021005460.00700182

0.00350091
0.010502730.007001820.010502730.007001820.00350091

0.010502730.007001820.010502730.00700182

0.010502730.00350091

0.00700182

0.007001820.00700182

0.010502730.017504550.003500910.007001820.007001820.010502730.003500910.017504550.00350091
0.007001820.003500910.010502730.003500910.017504550.00350091

0.010502730.00700182

0.01050273

0.00700182

0.010502730.00700182

0.010502730.00700182

0.003500910.021005460.007001820.028007280.03500910.073519110.024506370.003500910.017504550.00350091

0.01400364

0.01400364

0.007001820.007001820.003500910.017504550.021005460.003500910.00350091

0.007001820.01400364

0.007001820.007001820.003500910.010502730.007001820.003500910.00350091

0.014003640.024506370.017504550.038510010.021005460.017504550.00350091

0.01750455

0.014003640.007001820.017504550.038510010.021005460.017504550.00350091

0.00350091

0.00350091

0.066517290.024506370.213555520.024506370.199551880.374597390.115530040.063016380.108528220.049012740.080520930.063016380.04901274

0.007001820.010502730.00350091

0.00700182

0.01050273

0.01050273

0.01050273

0.00350091

0.00350091

0.00350091

0.00350091

0.00350091

0.059515470.024506370.213555520.024506370.185548240.374597390.112029130.063016380.108528220.049012740.080520930.063016380.04901274

0.014003640.003500910.038510010.024506370.185548240.175045510.080520940.056014560.094524580.042010920.063016380.059515470.04901274
0.059515470.024506370.213555520.024506370.185548240.374597390.112029130.063016380.108528220.049012740.080520930.063016380.04901274

0.045511830.010502730.098025480.196050970.028007280.014003640.007001820.017504550.00350091

0.007001820.066517290.147038230.007001820.007001820.007001820.00350091

0.003500910.010502730.01050273

0.045511830.003500910.031508190.045511830.010502730.003500910.01050273

0.010502730.077020030.003500910.003500910.00700182

0.003500910.003500910.014003640.017504550.017504550.007001820.01050273

0.003500910.003500910.014003640.017504550.017504550.007001820.01050273

0.003500910.003500910.01400364

0.003500910.003500910.01400364

0.00350091
0.003500910.01400364

0.01400364

0.01400364

0.00350091

0.00350091

0.014003640.003500910.017504550.007001820.01050273

0.014003640.003500910.017504550.007001820.01050273

0.014003640.003500910.017504550.007001820.01050273

0.003500910.00350091

0.003500910.00350091

0.014003640.003500910.014003640.007001820.00700182
