# Supplementary material for: Neuroprotective Effects of Oligosaccharides From Periplaneta Americana on Parkinson’s Disease Models In Vitro and In Vivo
Source: Front Pharmacol. 2022 Jul 18;13:936818. doi: 10.3389/fphar.2022.936818 (PMC9340460; doi:10.3389/fphar.2022.936818)
Supplement: Supplementary file 2 [file DataSheet1.zip › 16S rRNA/06.FunctionPrediction/Images/KEGG_level2_Genetic Information Processing.pdf]

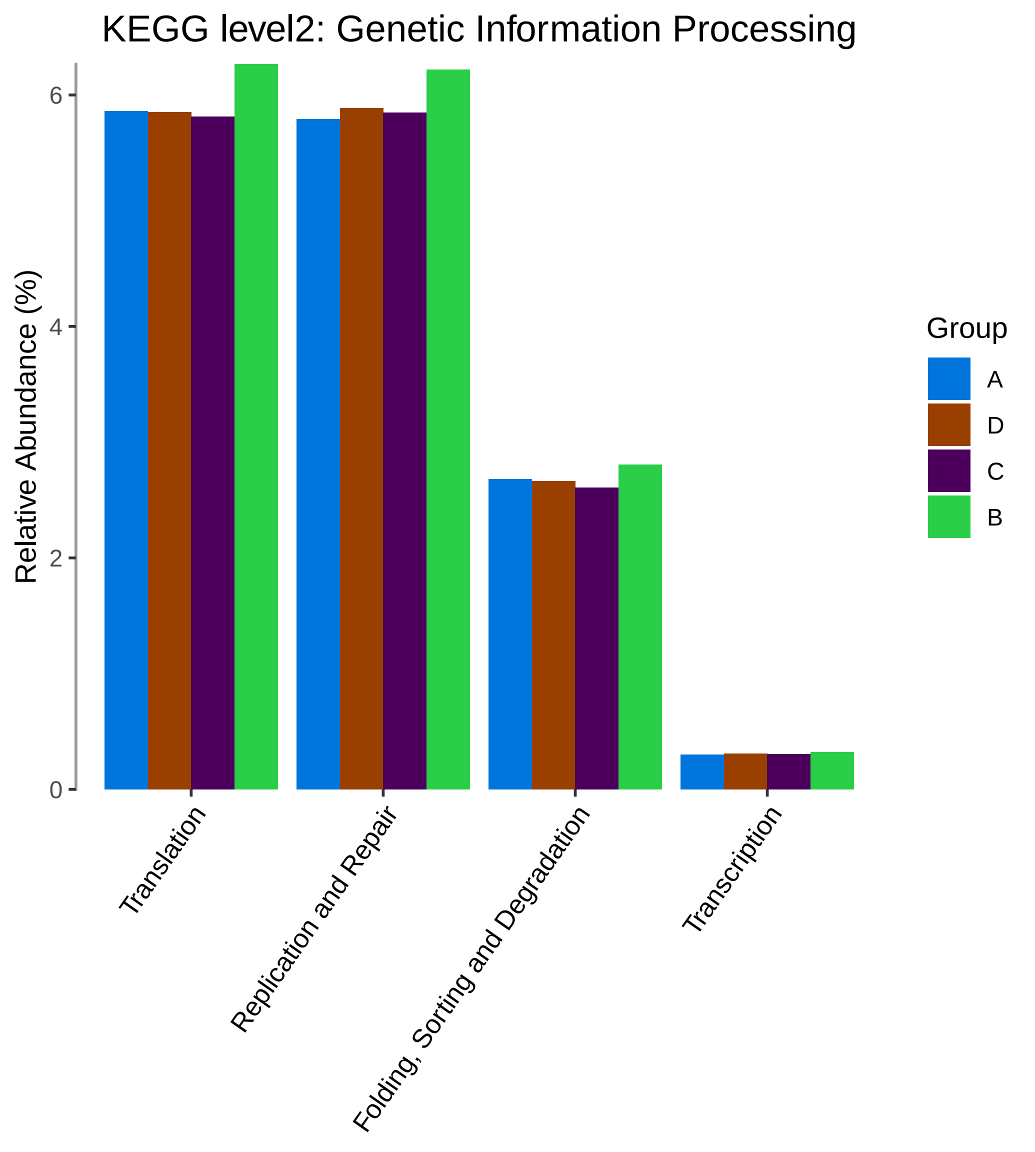
KEGG level2: Genetic Information Processing
Relative Abundance (%)
0
2
4
6
Translation
Replication and Repair
Folding, Sorting and Degradation
Transcription
Group
A
D
C
B
