# Supplementary material for: Neuroprotective Effects of Oligosaccharides From Periplaneta Americana on Parkinson’s Disease Models In Vitro and In Vivo
Source: Front Pharmacol. 2022 Jul 18;13:936818. doi: 10.3389/fphar.2022.936818 (PMC9340460; doi:10.3389/fphar.2022.936818)
Supplement: Supplementary file 2 [file DataSheet1.zip › 16S rRNA/06.FunctionPrediction/Images/KEGG_total_wilcox-test.pdf]

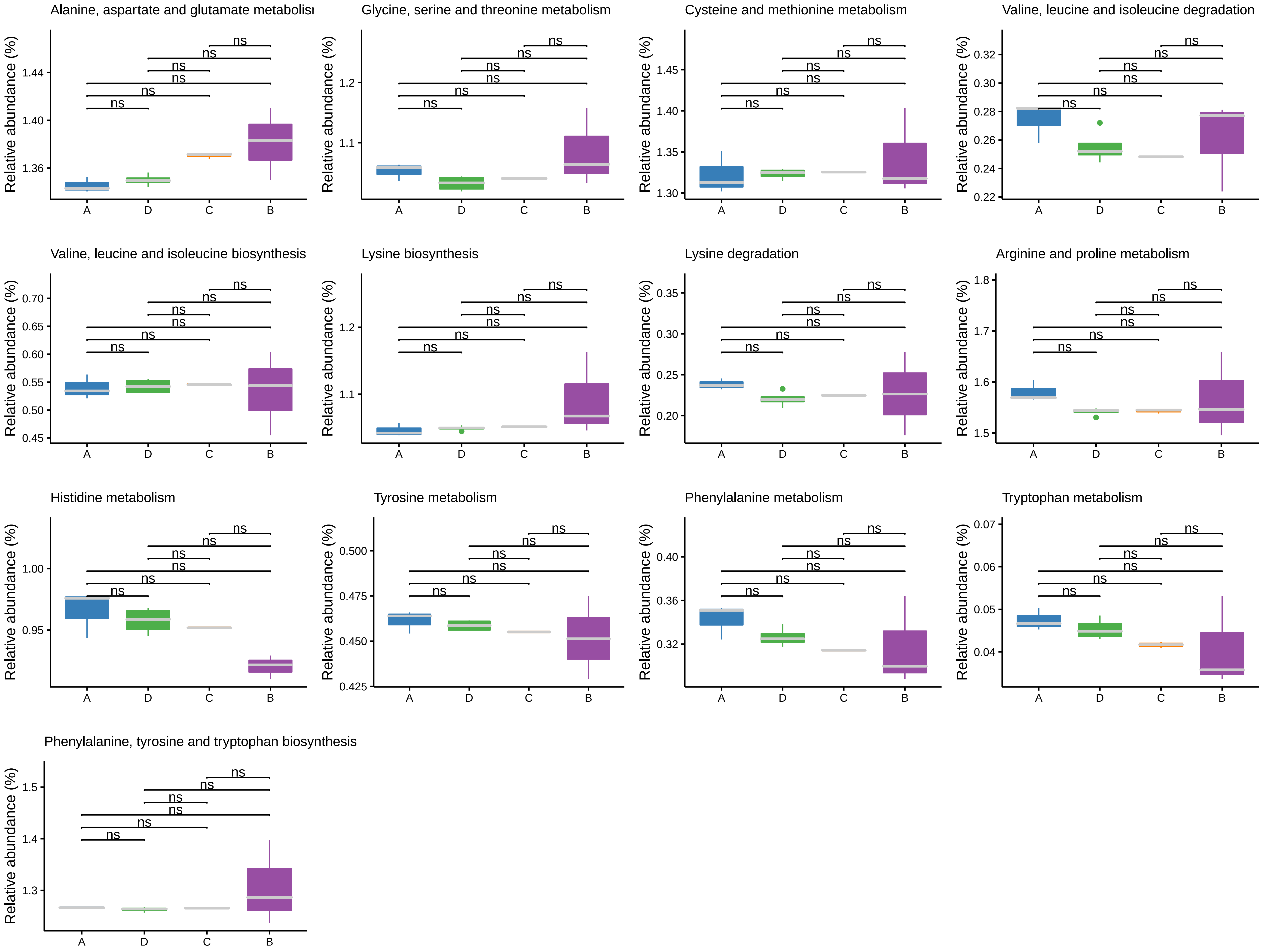
Alanine, aspartate and glutamate metabolisı
Relative abundance (%)
1.36 1.40 1.44
A D C B
ns
Glycine, serine and threonine metabolism
Relative abundance (%)
1.1 1.2
A D C B
ns
Cysteine and methionine metabolism
Relative abundance (%)
1.30 1.35 1.40 1.45
A D C B
ns
Valine, leucine and isoleucine degradation
Relative abundance (%)
0.22 0.24 0.26 0.28 0.30 0.32
A D C B
ns
Valine, leucine and isoleucine biosynthesis
Relative abundance (%)
0.45 0.50 0.55 0.60 0.65 0.70
A D C B
ns
Lysine biosynthesis
Relative abundance (%)
1.1 1.2
A D C B
ns
Lysine degradation
Relative abundance (%)
0.20 0.25 0.30 0.35
A D C B
ns
Arginine and proline metabolism
Relative abundance (%)
1.5 1.6 1.7 1.8
A D C B
ns
Histidine metabolism
Relative abundance (%)
0.95 1.00
A D C B
ns
Tyrosine metabolism
Relative abundance (%)
0.425 0.450 0.475 0.500
A D C B
ns
Phenylalanine metabolism
Relative abundance (%)
0.32 0.36 0.40
A D C B
ns
Tryptophan metabolism
Relative abundance (%)
0.04 0.05 0.06 0.07
A D C B
ns
Phenylalanine, tyrosine and tryptophan biosynthesis
Relative abundance (%)
1.3 1.4 1.5
A D C B
ns
