# Supplementary figures and images for: Neuroprotective Effects of Oligosaccharides From Periplaneta Americana on Parkinson’s Disease Models In Vitro and In Vivo (part 2 of 2)
Source: Front Pharmacol. 2022 Jul 18;13:936818. doi: 10.3389/fphar.2022.936818 (PMC9340460; doi:10.3389/fphar.2022.936818)

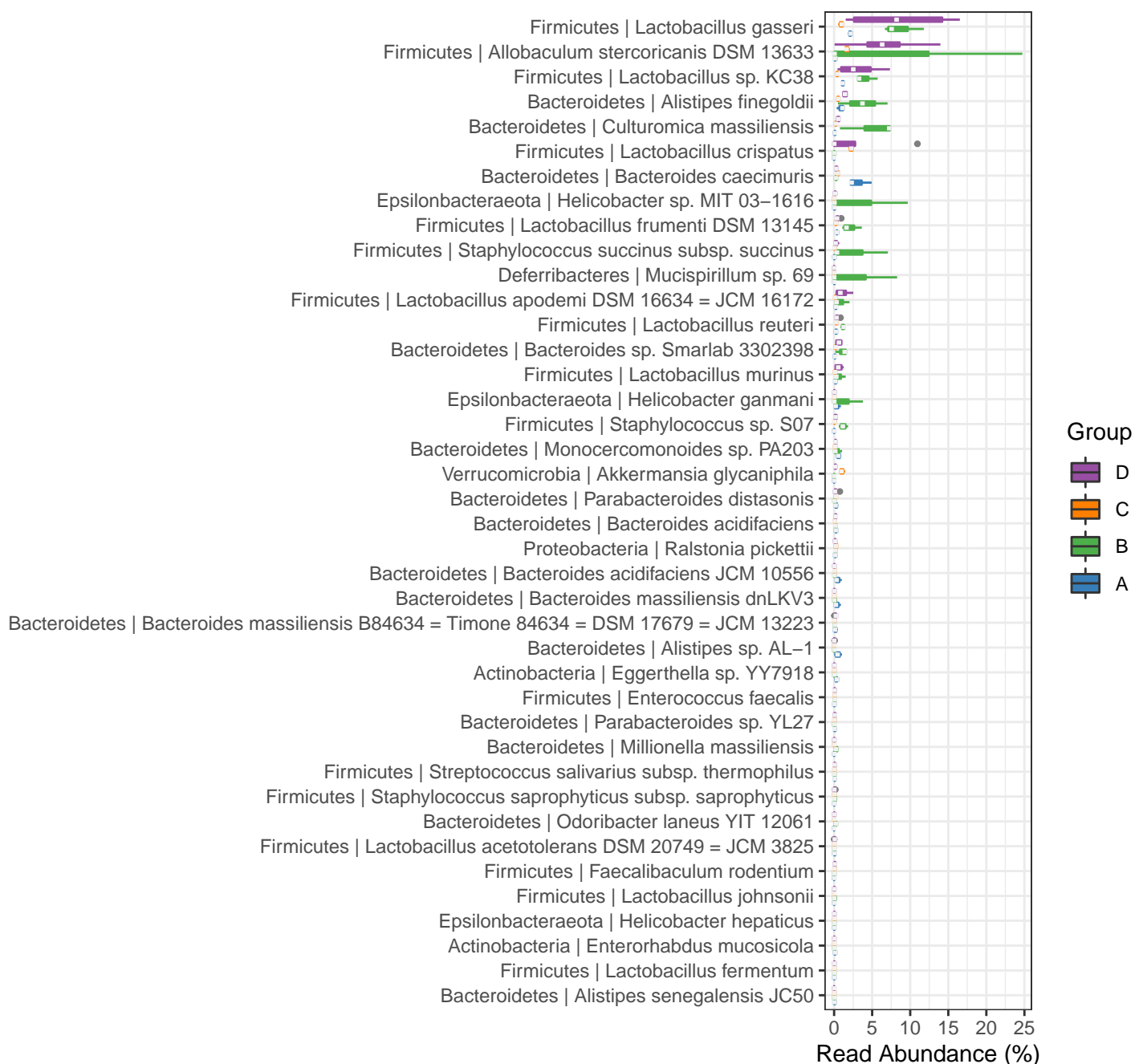

Supplement: Supplementary file 2 [file DataSheet1.zip › 16S rRNA/Images/Boxplot_Species.pdf]

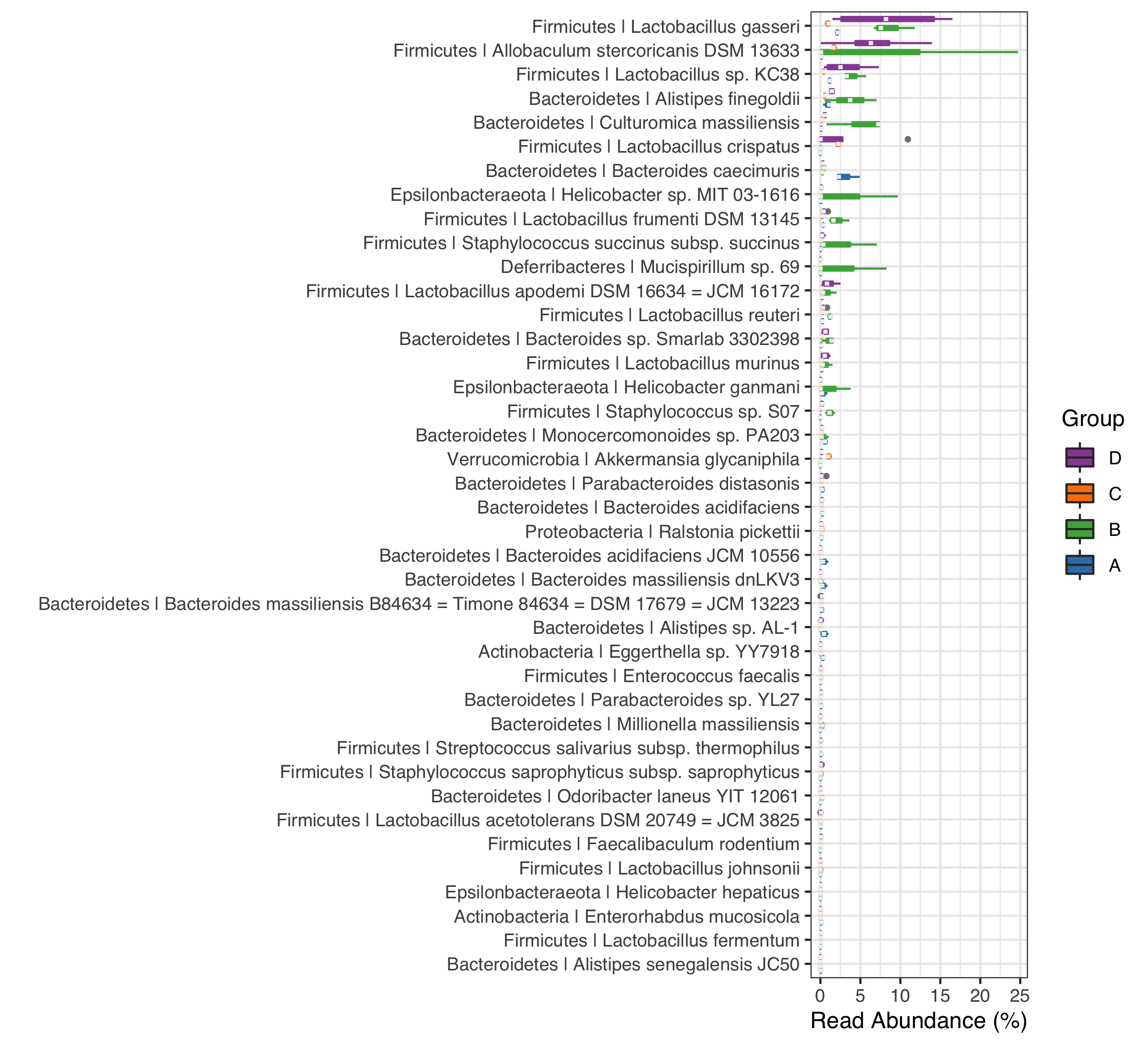

Supplement: Supplementary file 2 [file DataSheet1.zip › 16S rRNA/Images/Boxplot_Species.png]

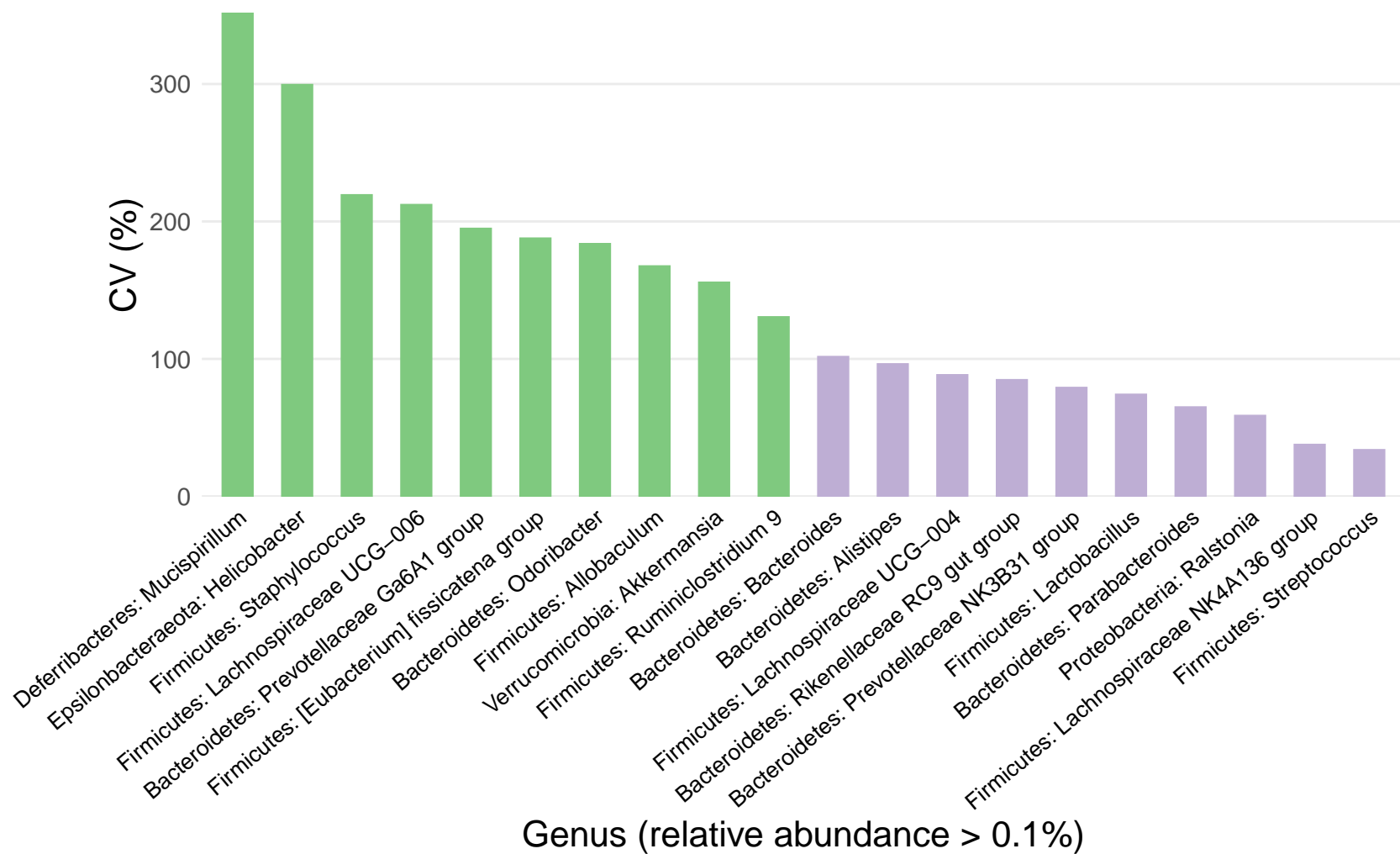

Supplement: Supplementary file 2 [file DataSheet1.zip › 16S rRNA/Images/CV_table_genus.pdf]

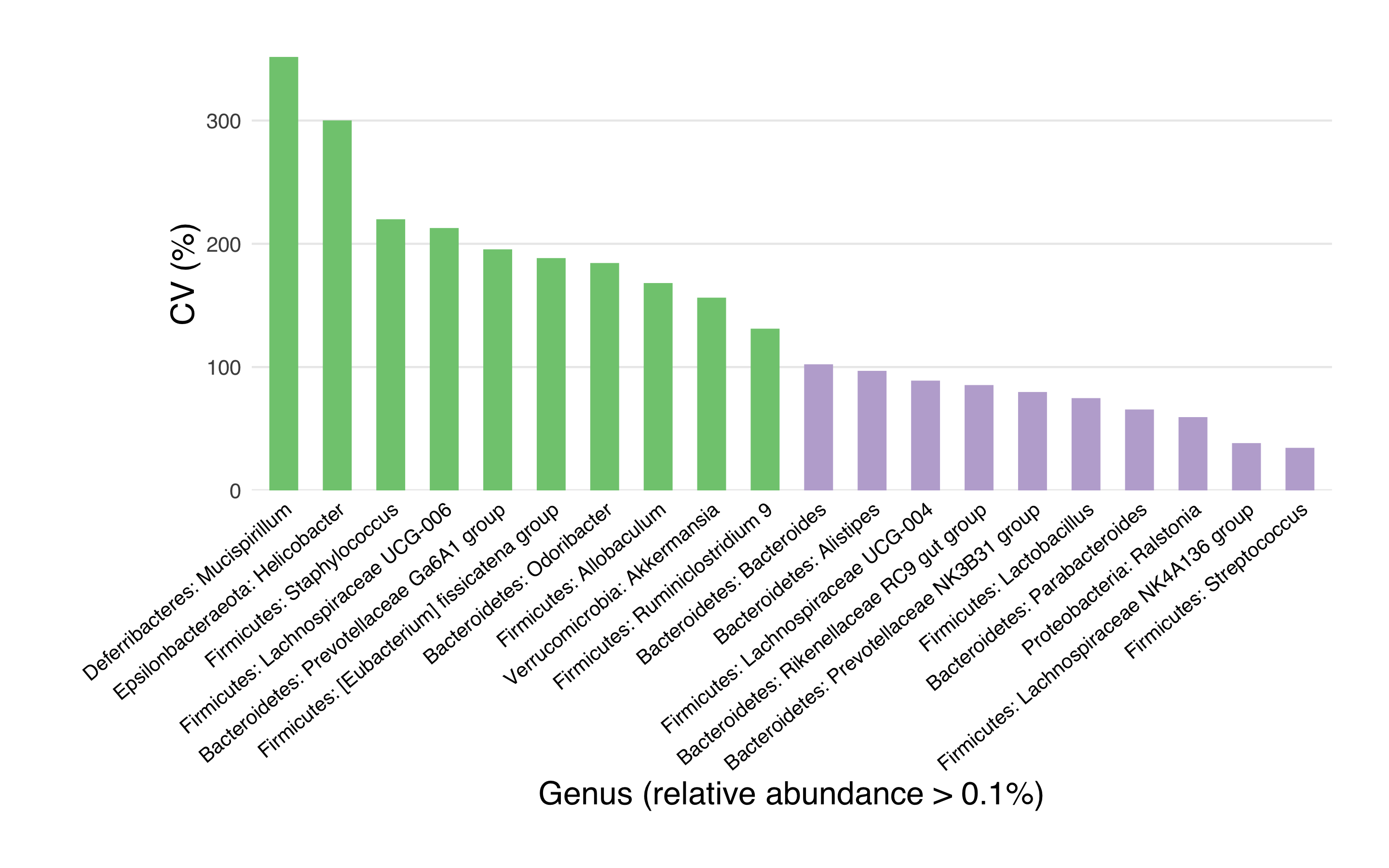

Supplement: Supplementary file 2 [file DataSheet1.zip › 16S rRNA/Images/CV_table_genus.png]

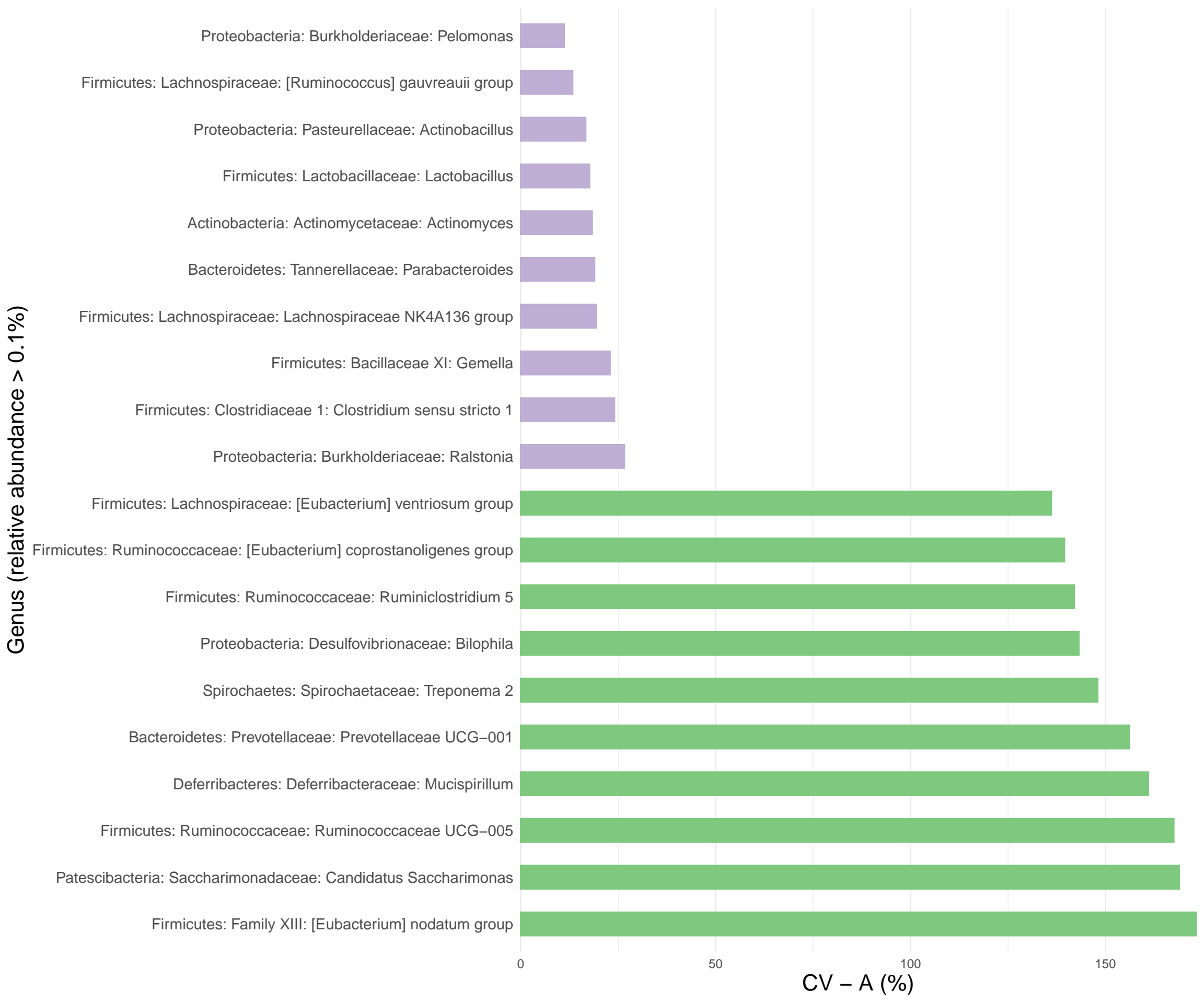

Supplement: Supplementary file 2 [file DataSheet1.zip › 16S rRNA/Images/CV_table_genus_A.pdf]
