# Supplementary figures and images for: Neuroprotective Effects of Oligosaccharides From Periplaneta Americana on Parkinson’s Disease Models In Vitro and In Vivo (part 1 of 2)
Source: Front Pharmacol. 2022 Jul 18;13:936818. doi: 10.3389/fphar.2022.936818 (PMC9340460; doi:10.3389/fphar.2022.936818)

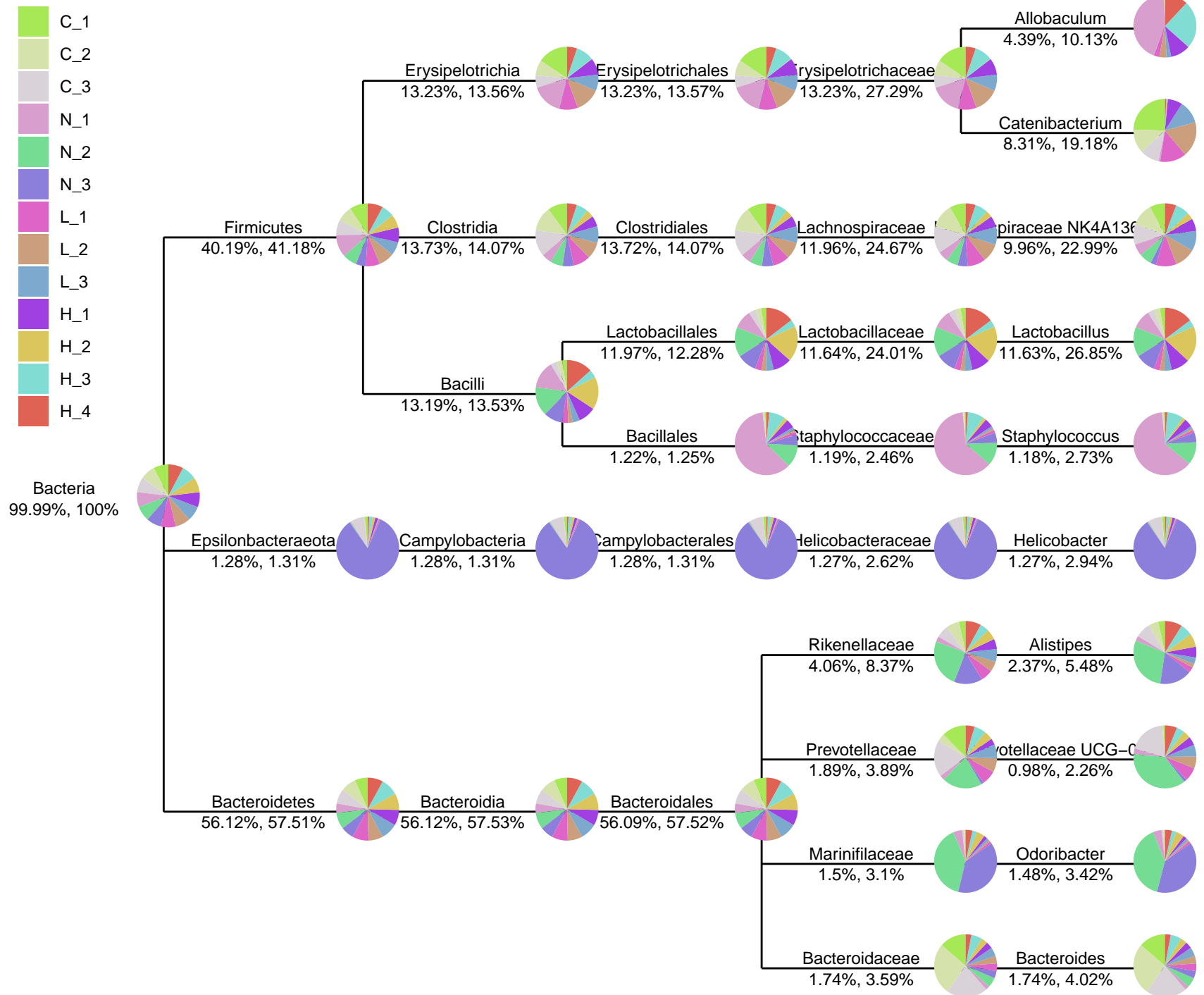

Supplement: Supplementary file 2 [file DataSheet1.zip › 16S rRNA/Images/specific_taxonomic_tree.pdf]

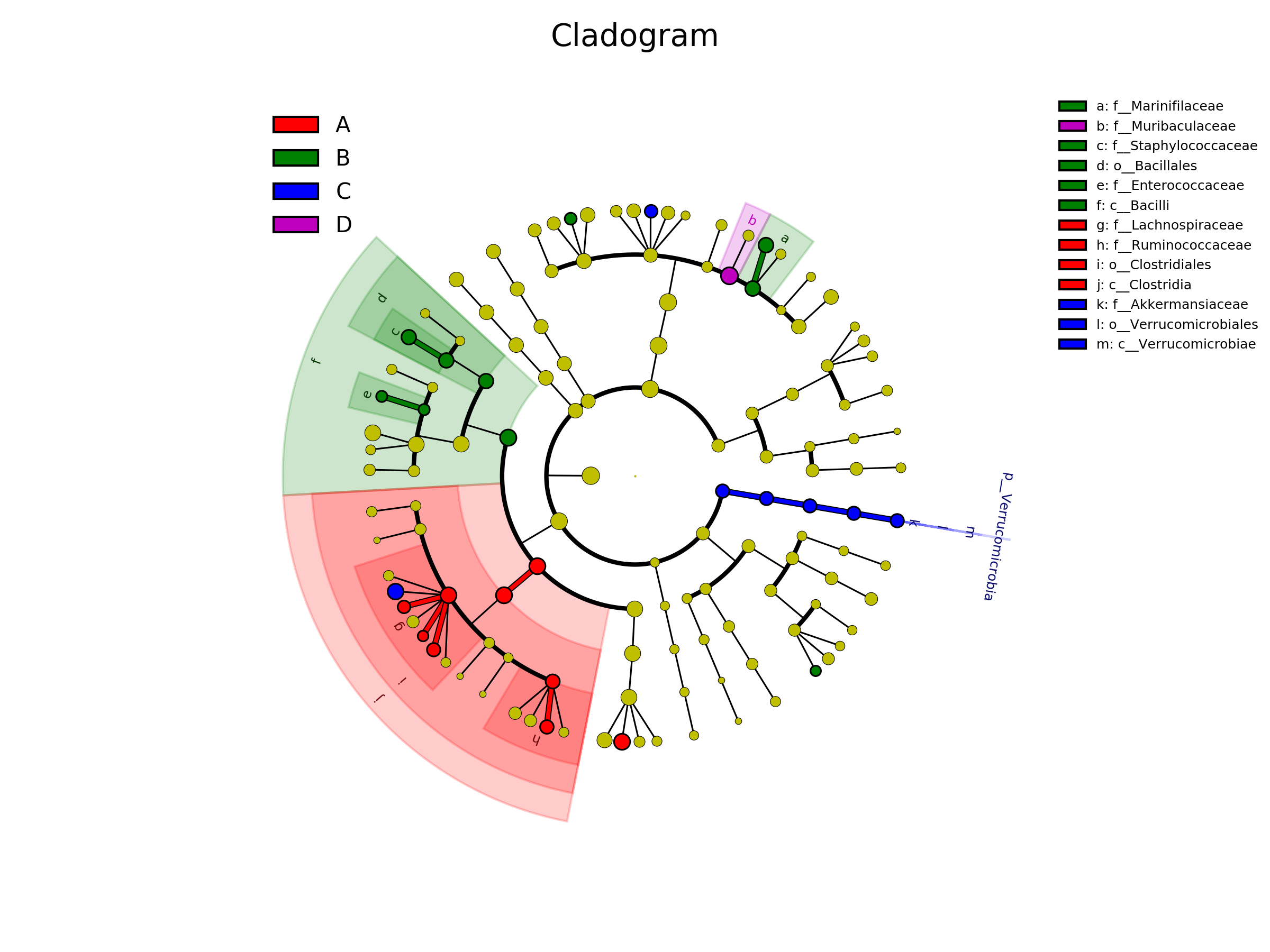

Supplement: Supplementary file 2 [file DataSheet1.zip › 16S rRNA/Images/lefse_cladogram.png]

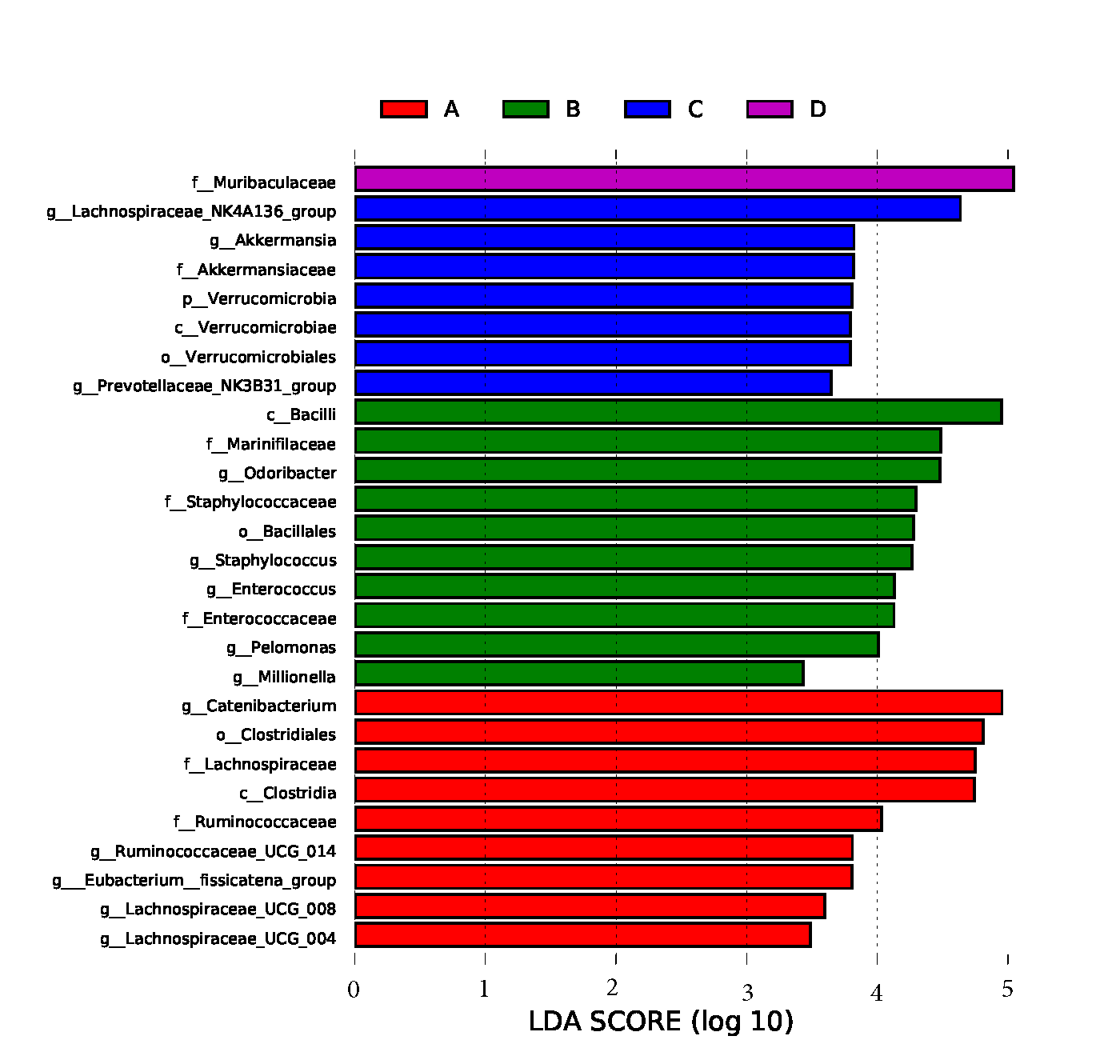

Supplement: Supplementary file 2 [file DataSheet1.zip › 16S rRNA/Images/lefse.png]

A B C D

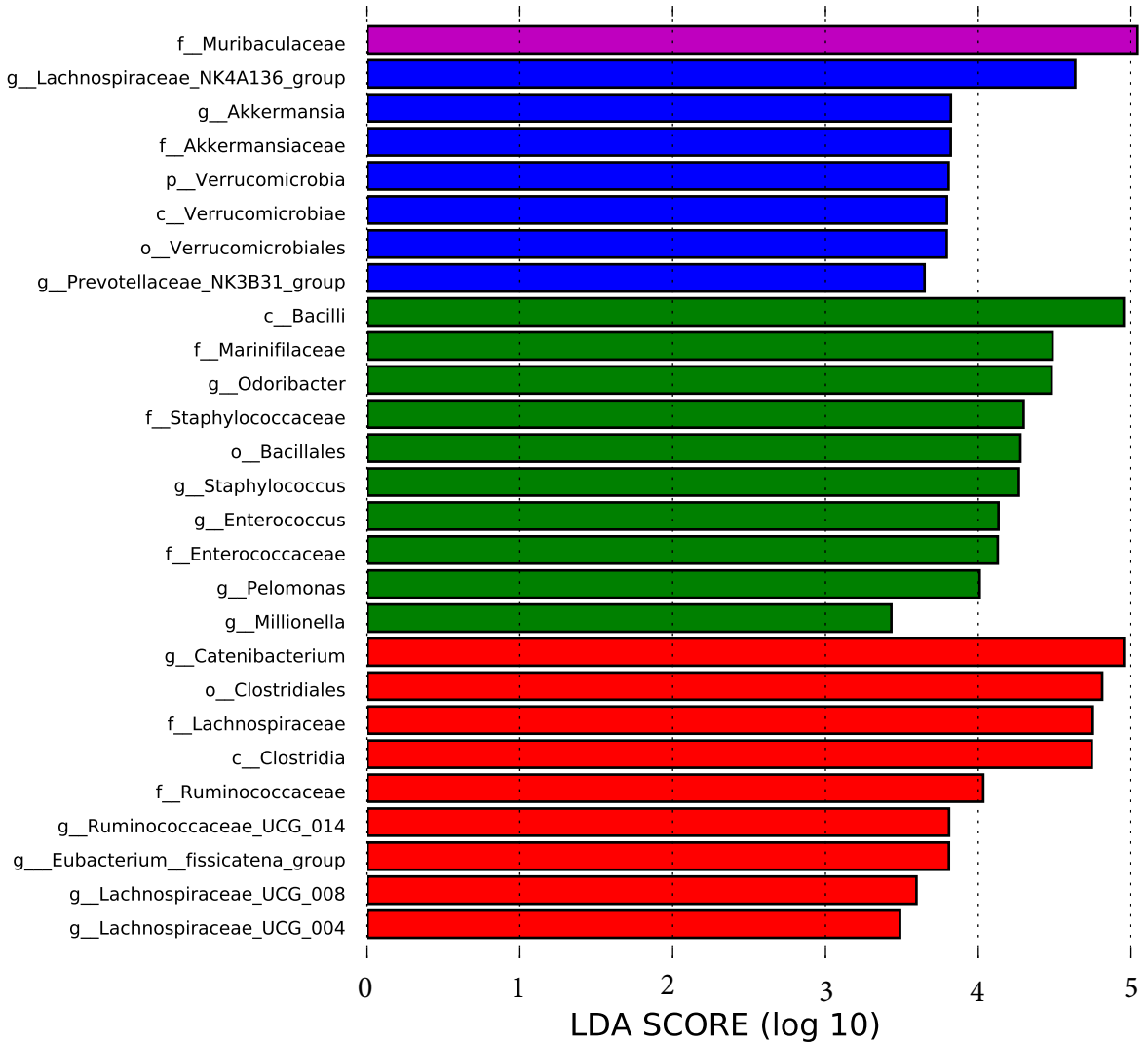

Supplement: Supplementary file 2 [file DataSheet1.zip › 16S rRNA/Images/lefse.pdf]

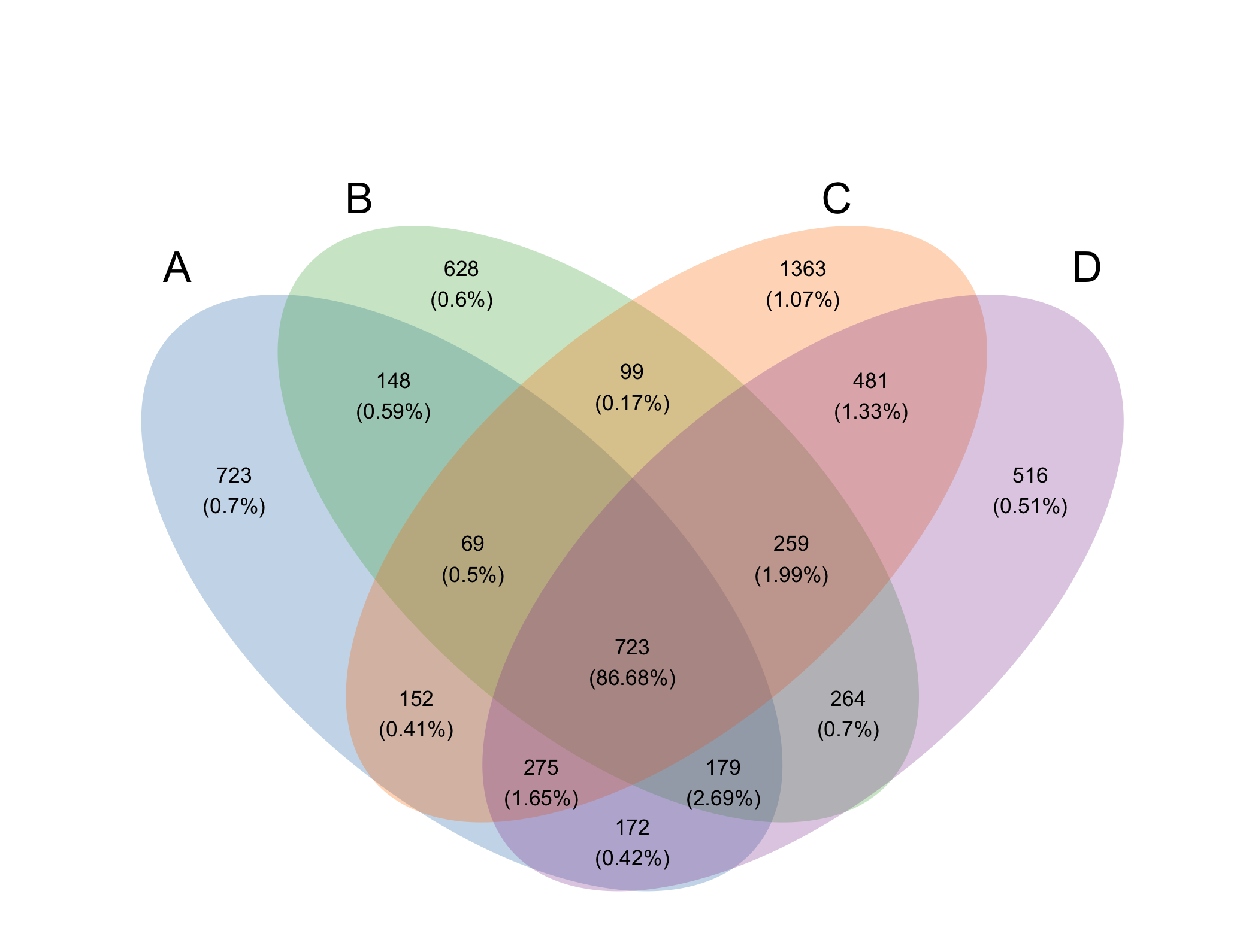

Supplement: Supplementary file 2 [file DataSheet1.zip › 16S rRNA/Images/Venn_Diagram/Venn_plot.png]

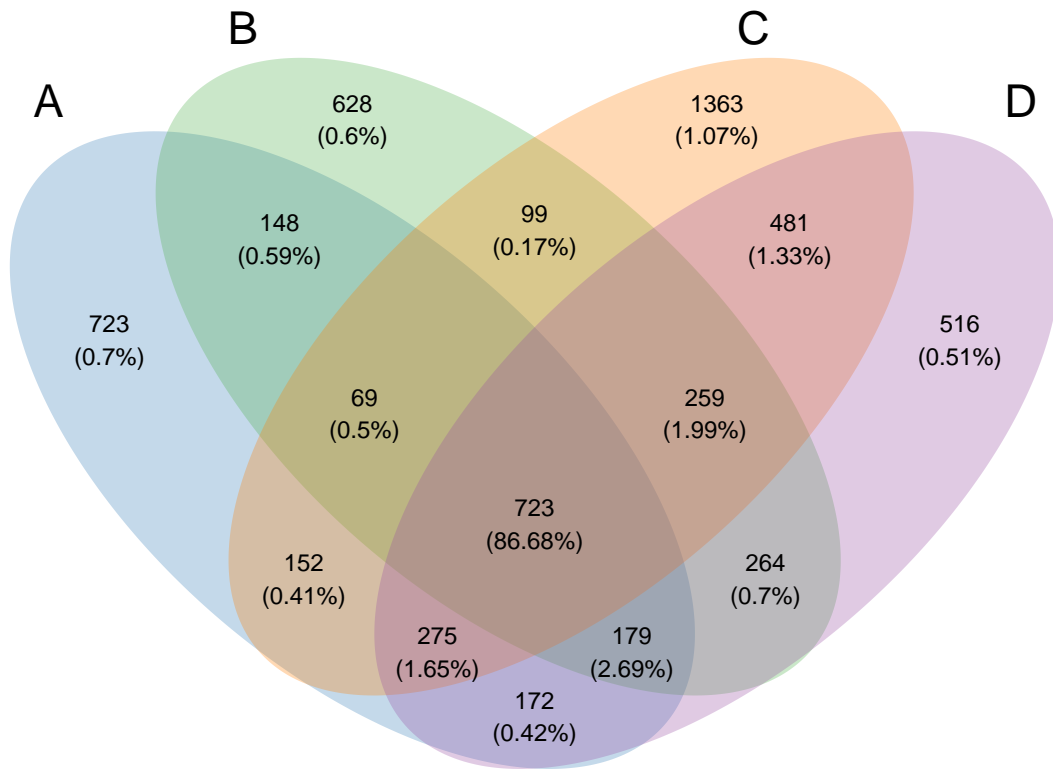

Supplement: Supplementary file 2 [file DataSheet1.zip › 16S rRNA/Images/Venn_Diagram/Venn_plot.pdf]

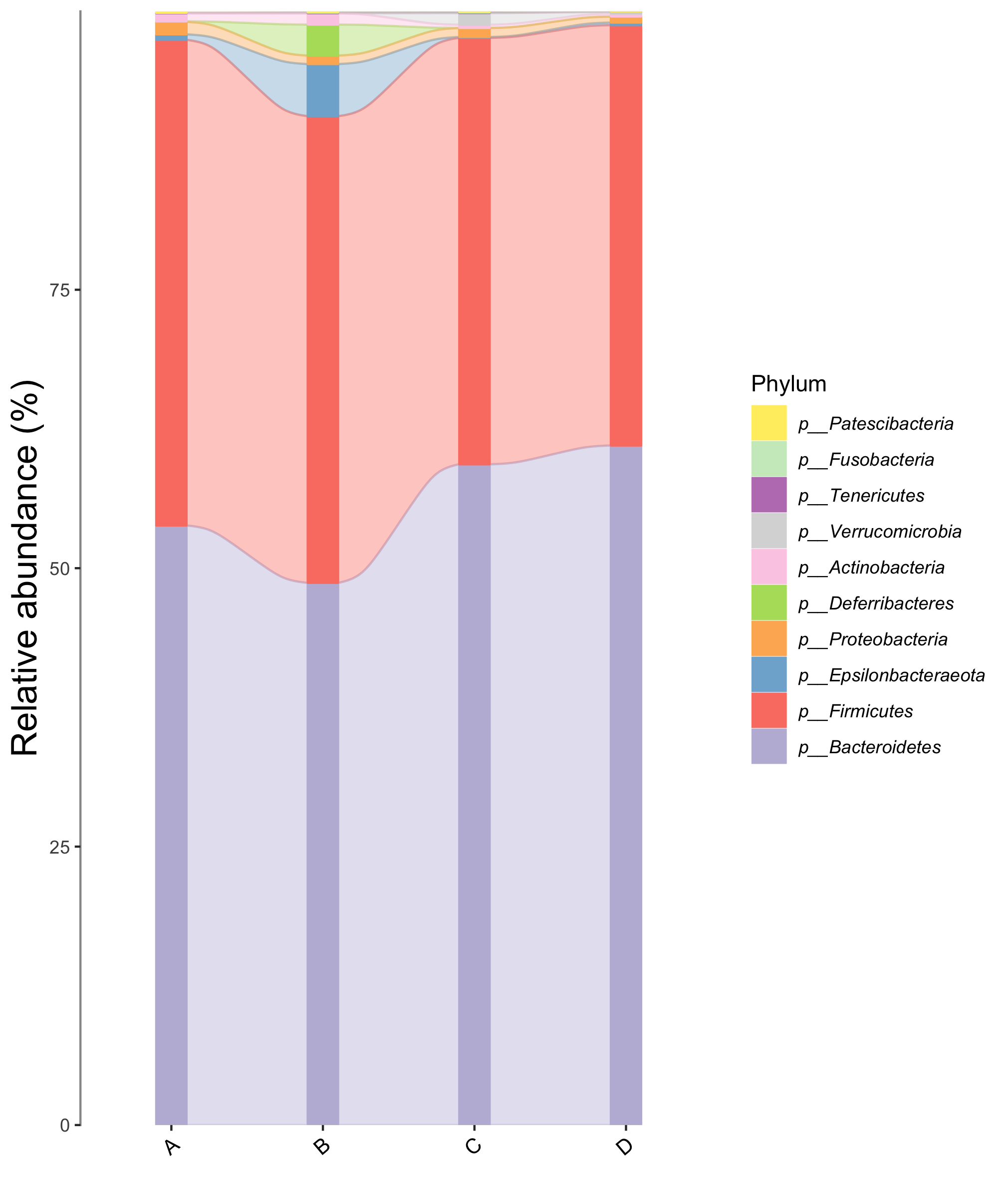

Supplement: Supplementary file 2 [file DataSheet1.zip › 16S rRNA/Images/Sankeyplot_Phylum_mean.png]

Relative abundance (%)

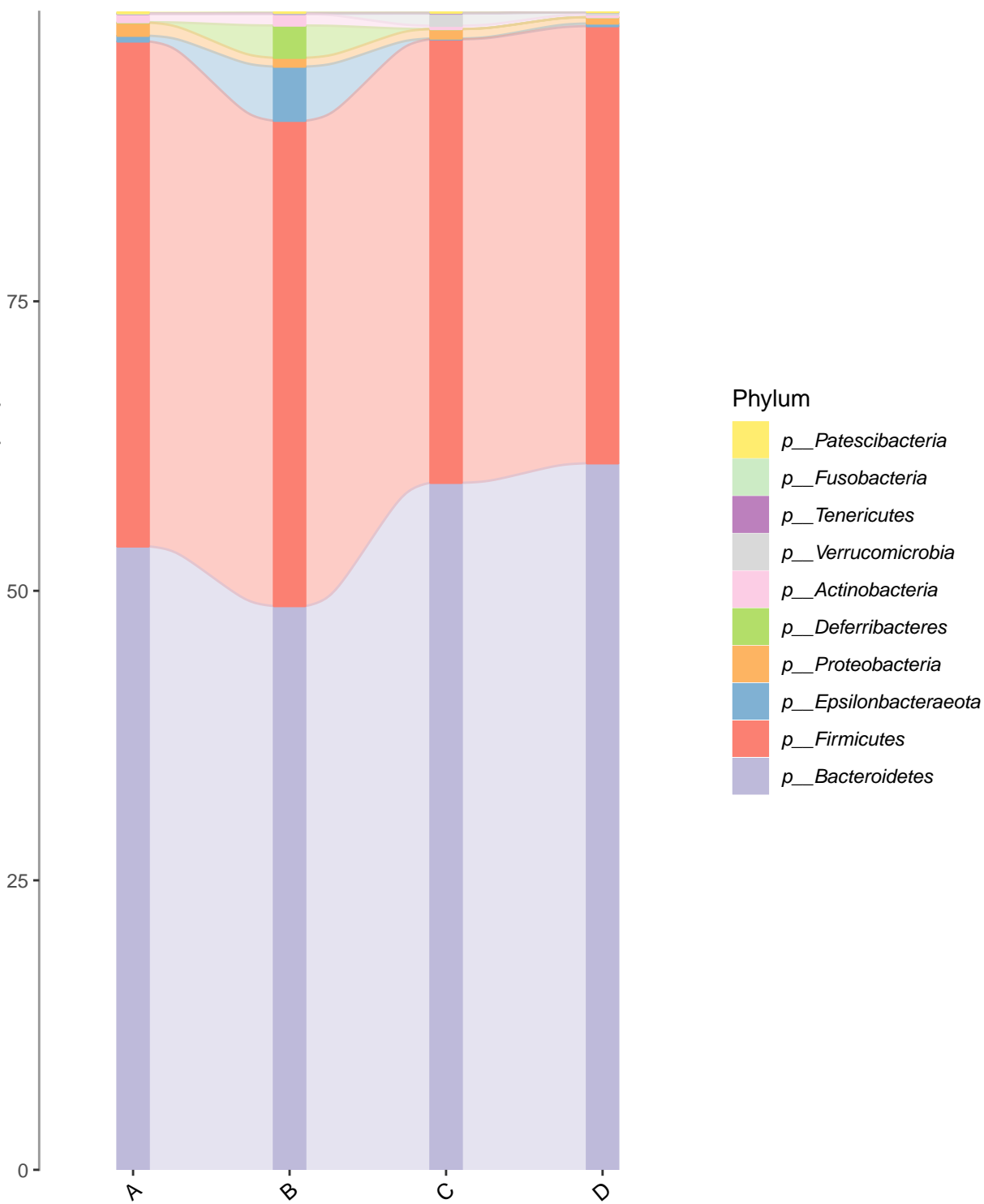

Supplement: Supplementary file 2 [file DataSheet1.zip › 16S rRNA/Images/Sankeyplot_Phylum_mean.pdf]

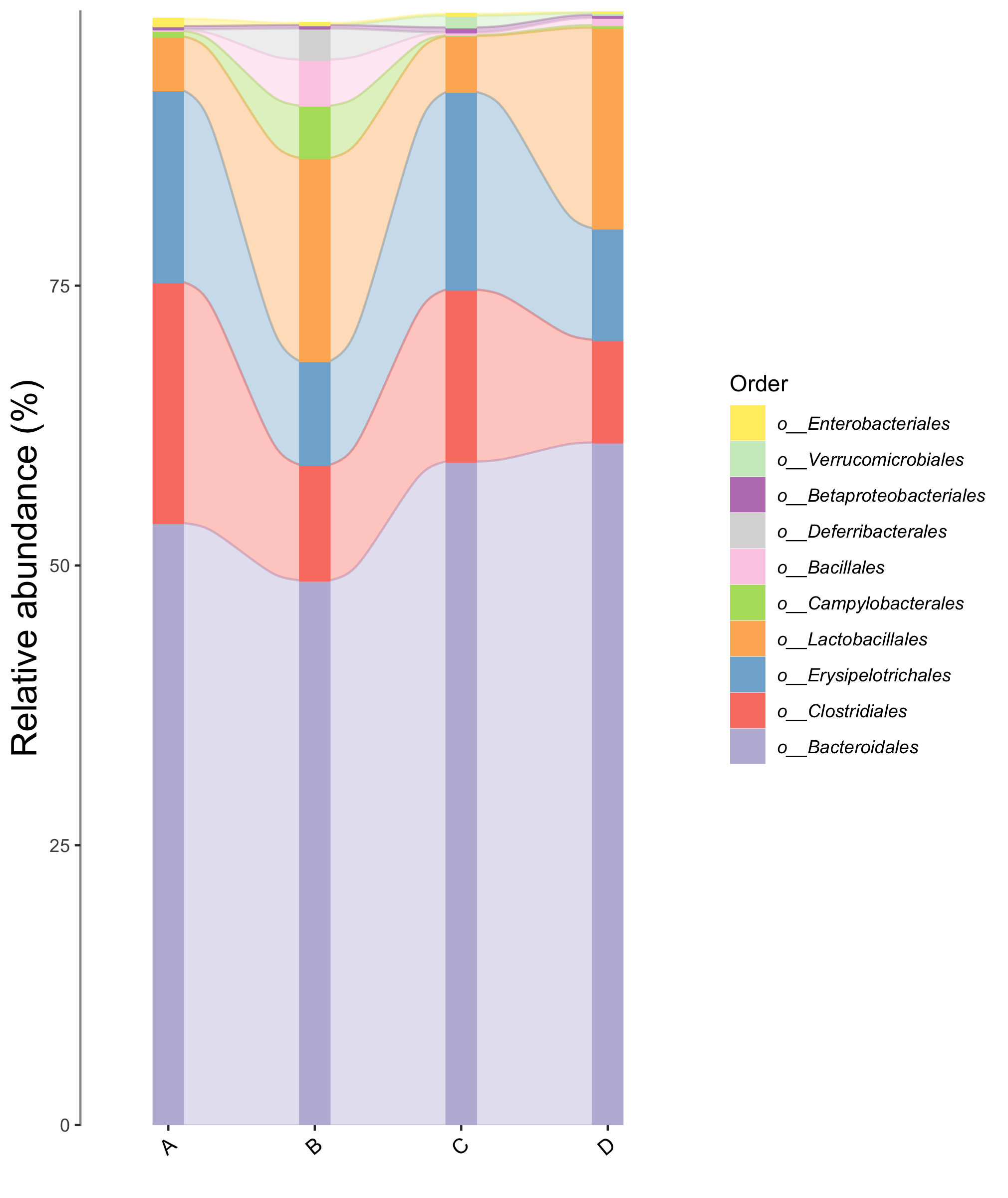

Supplement: Supplementary file 2 [file DataSheet1.zip › 16S rRNA/Images/Sankeyplot_Order_mean.png]

Relative abundance (%)

0

25

50

75

A

B

C

D

Order

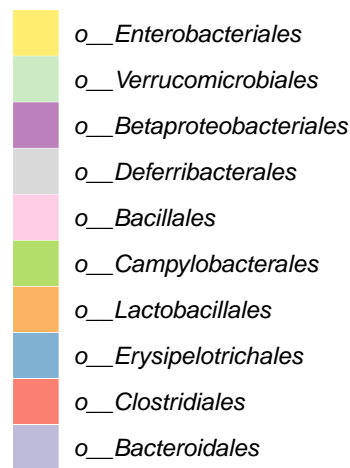

Supplement: Supplementary file 2 [file DataSheet1.zip › 16S rRNA/Images/Sankeyplot_Order_mean.pdf]

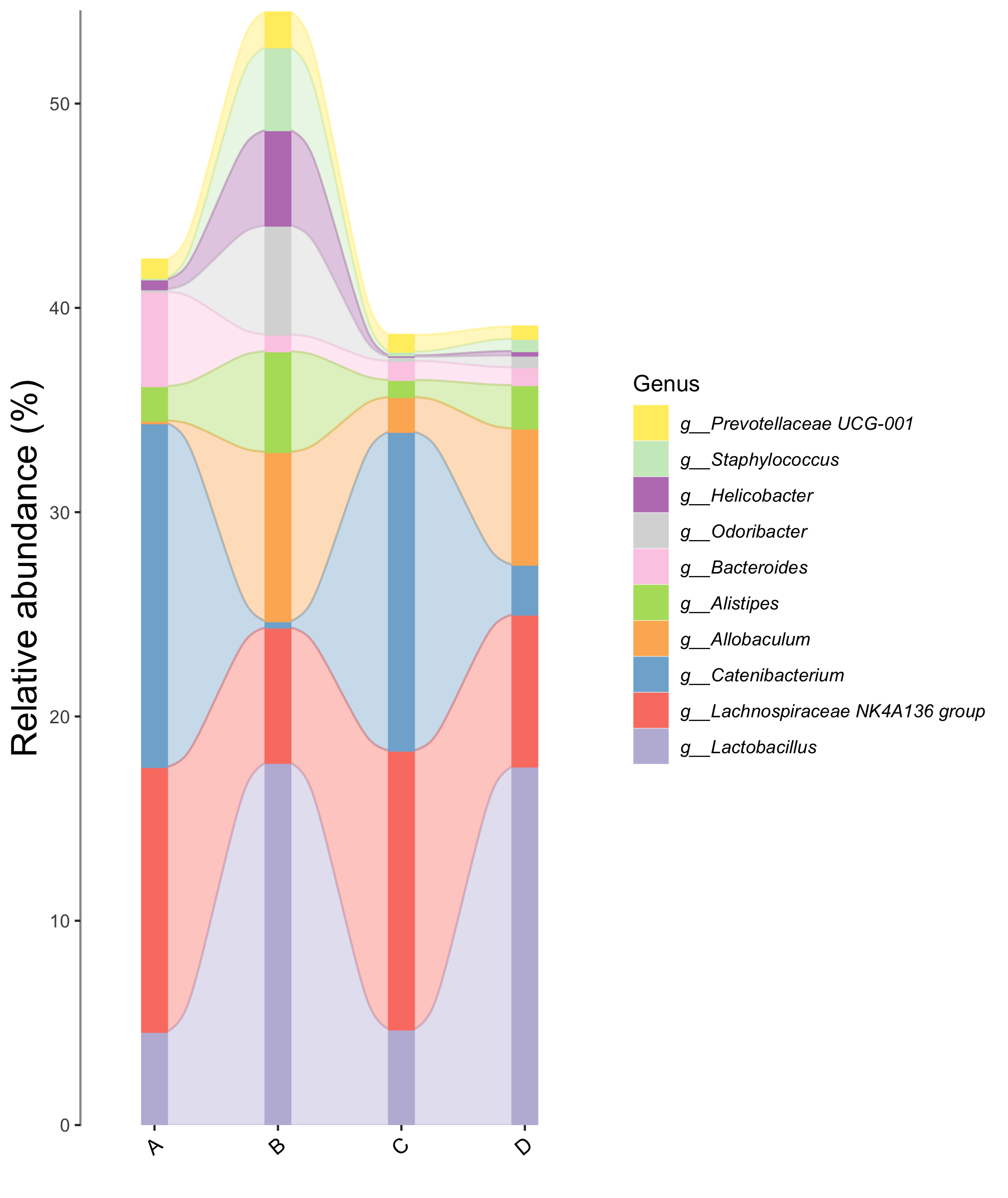

Supplement: Supplementary file 2 [file DataSheet1.zip › 16S rRNA/Images/Sankeyplot_Genus_mean.png]

Relative abundance (%)

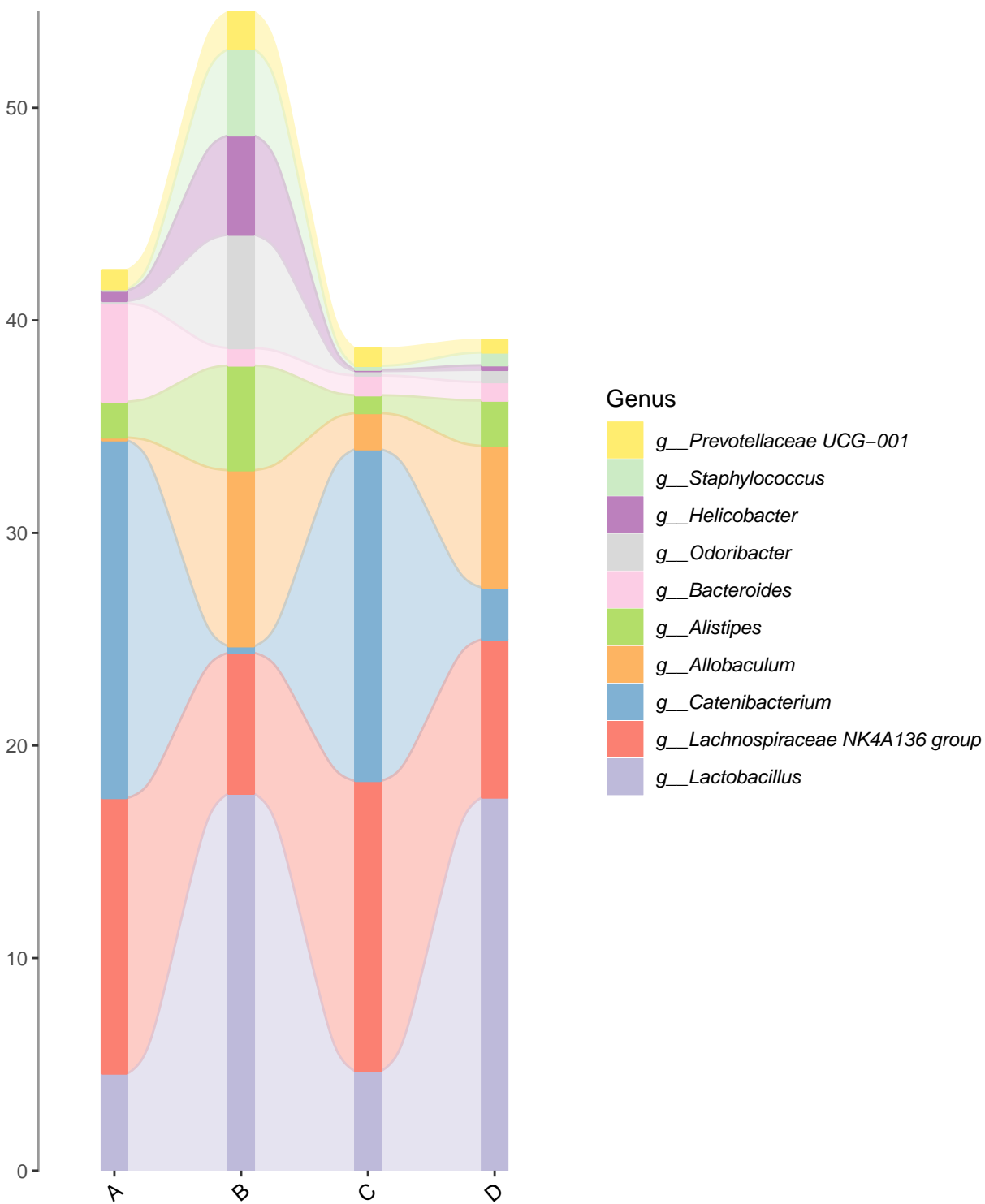

Supplement: Supplementary file 2 [file DataSheet1.zip › 16S rRNA/Images/Sankeyplot_Genus_mean.pdf]

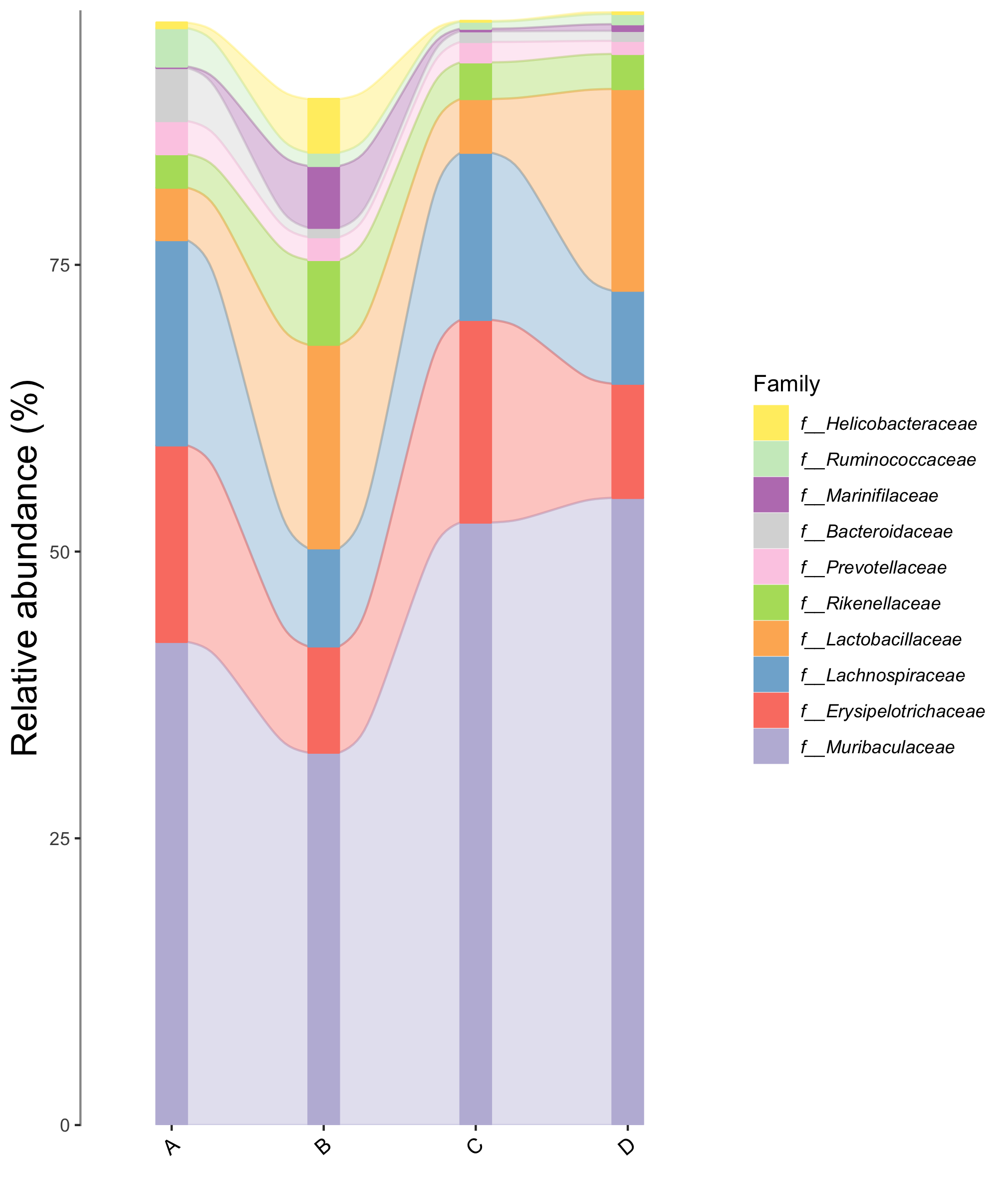

Supplement: Supplementary file 2 [file DataSheet1.zip › 16S rRNA/Images/Sankeyplot_Family_mean.png]

Relative abundance (%)

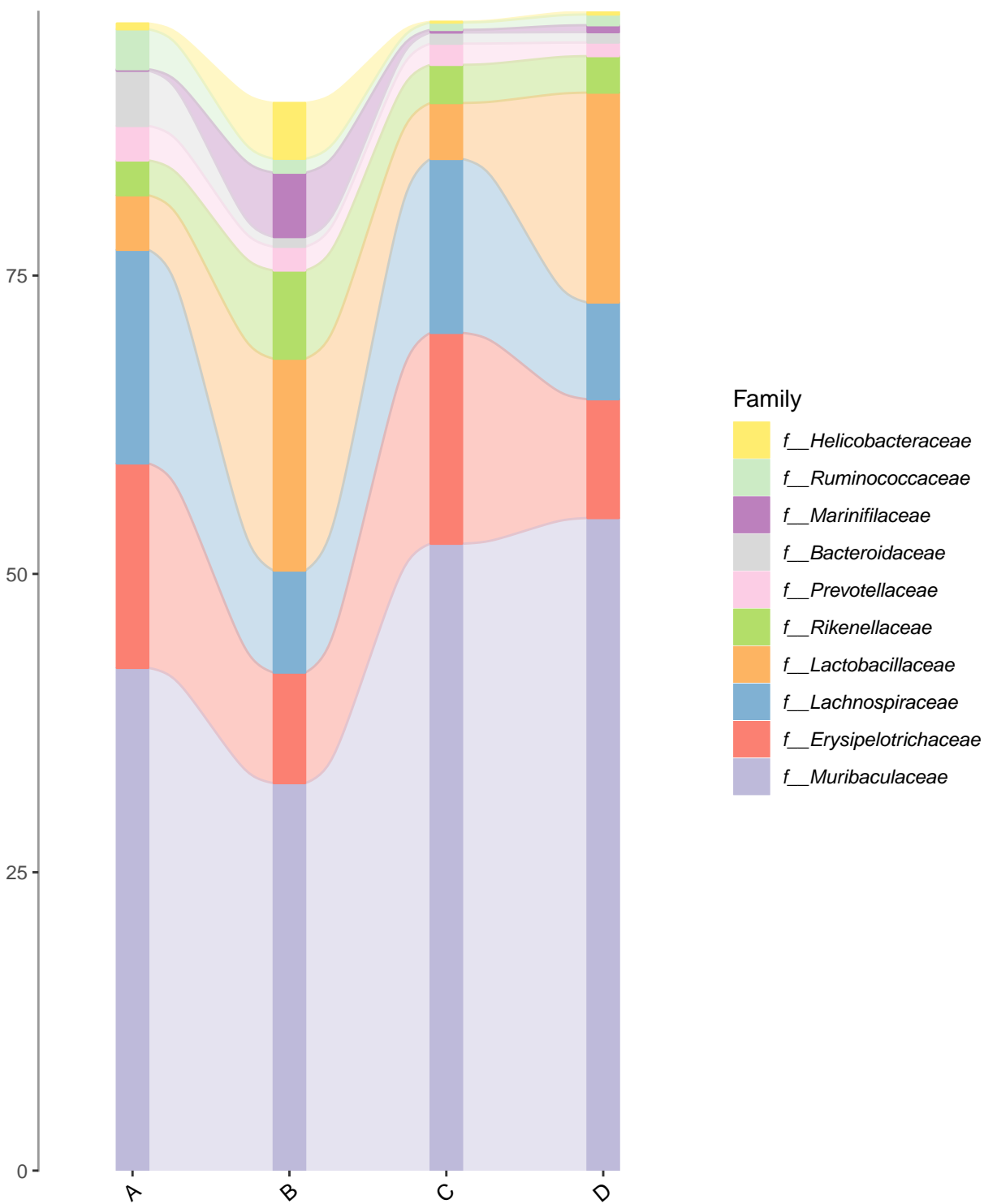

Supplement: Supplementary file 2 [file DataSheet1.zip › 16S rRNA/Images/Sankeyplot_Family_mean.pdf]

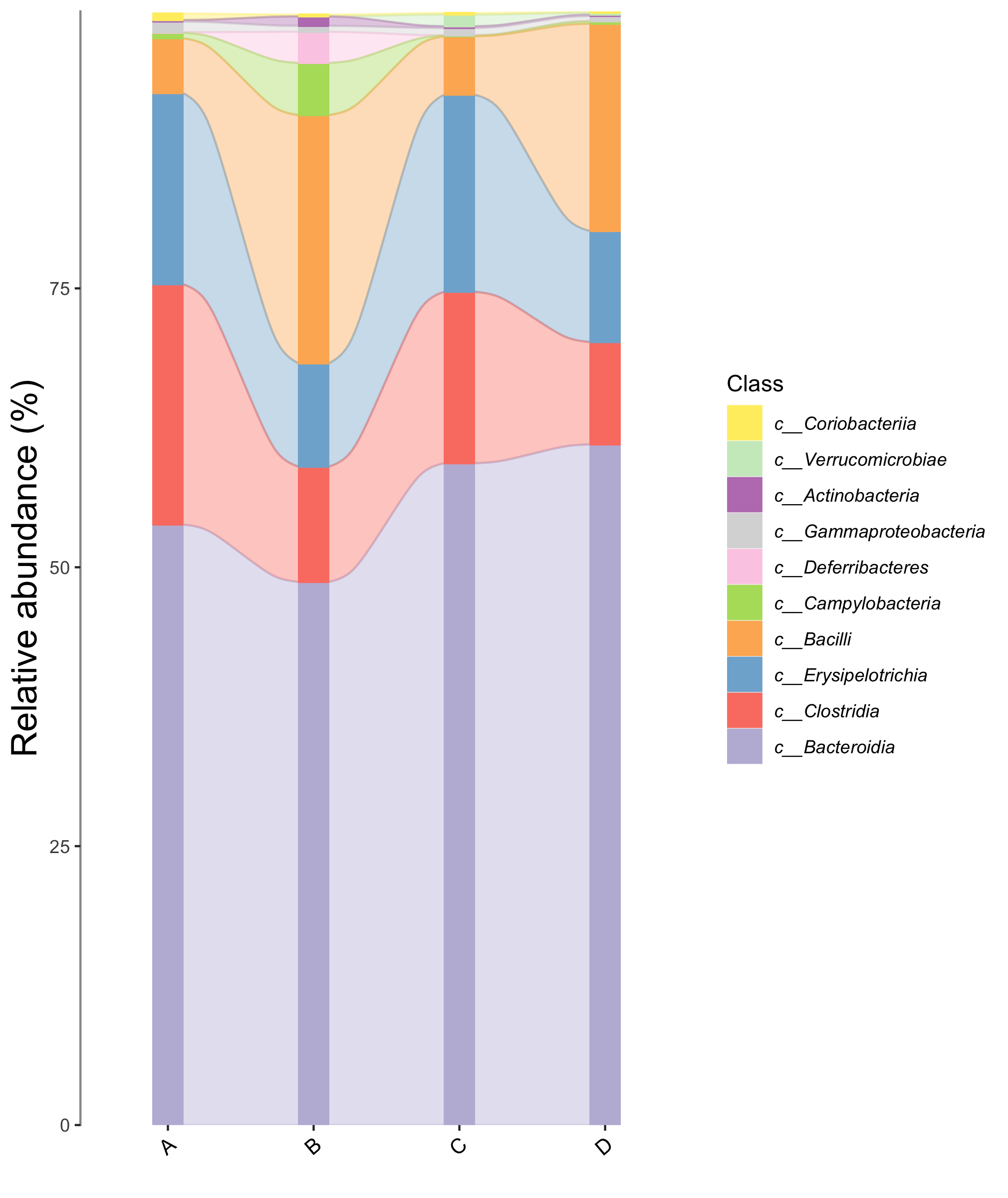

Supplement: Supplementary file 2 [file DataSheet1.zip › 16S rRNA/Images/Sankeyplot_Class_mean.png]

Relative abundance (%)

0

25

50

75

A

B

C

D

Class

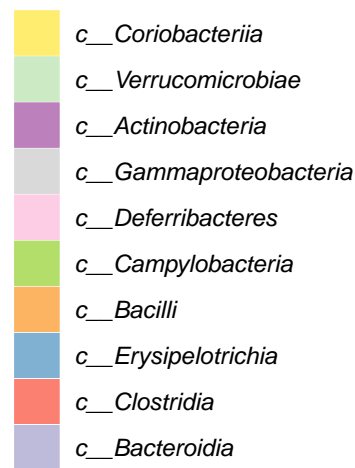

Supplement: Supplementary file 2 [file DataSheet1.zip › 16S rRNA/Images/Sankeyplot_Class_mean.pdf]

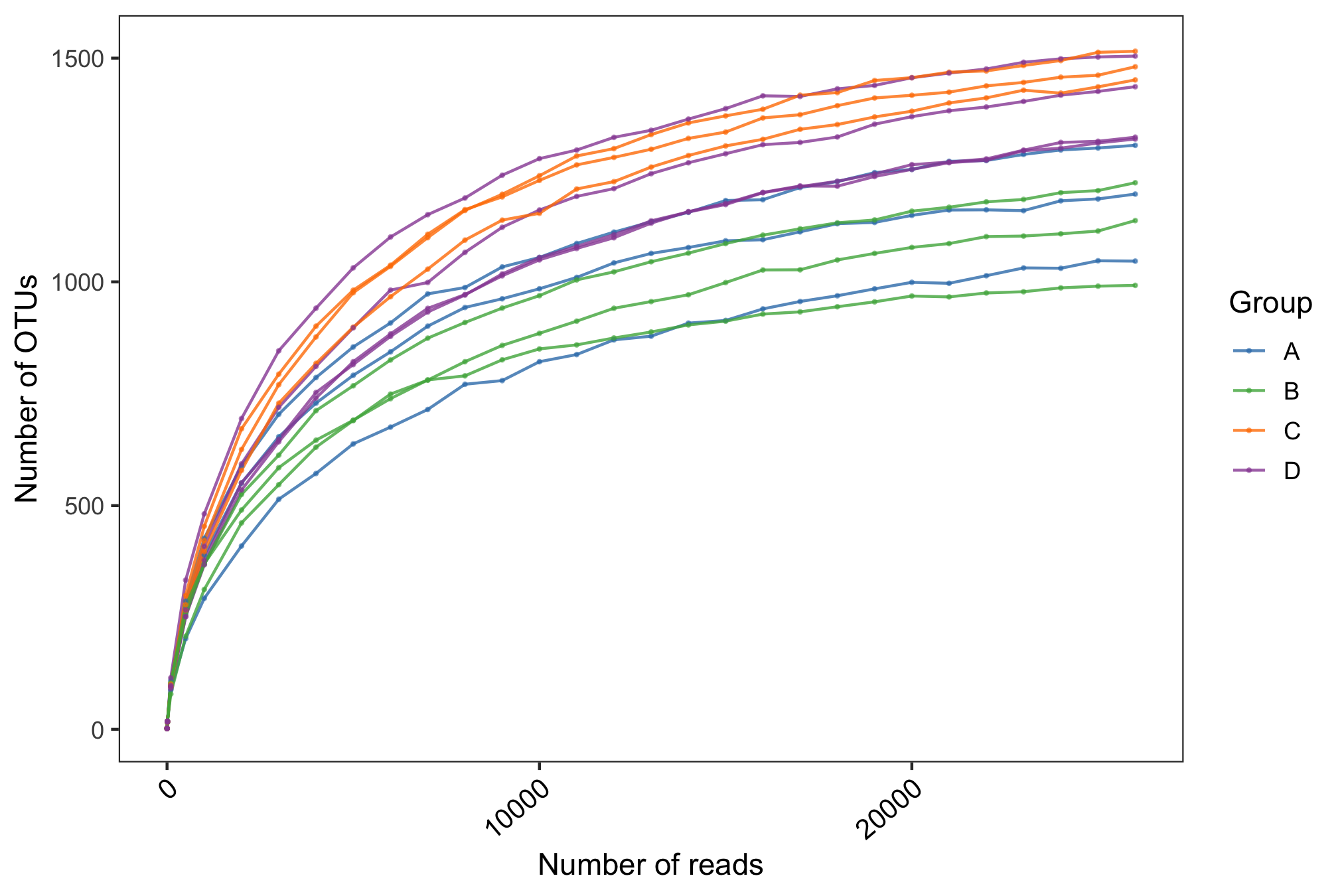

Supplement: Supplementary file 2 [file DataSheet1.zip › 16S rRNA/Images/Rarefaction_curve.png]

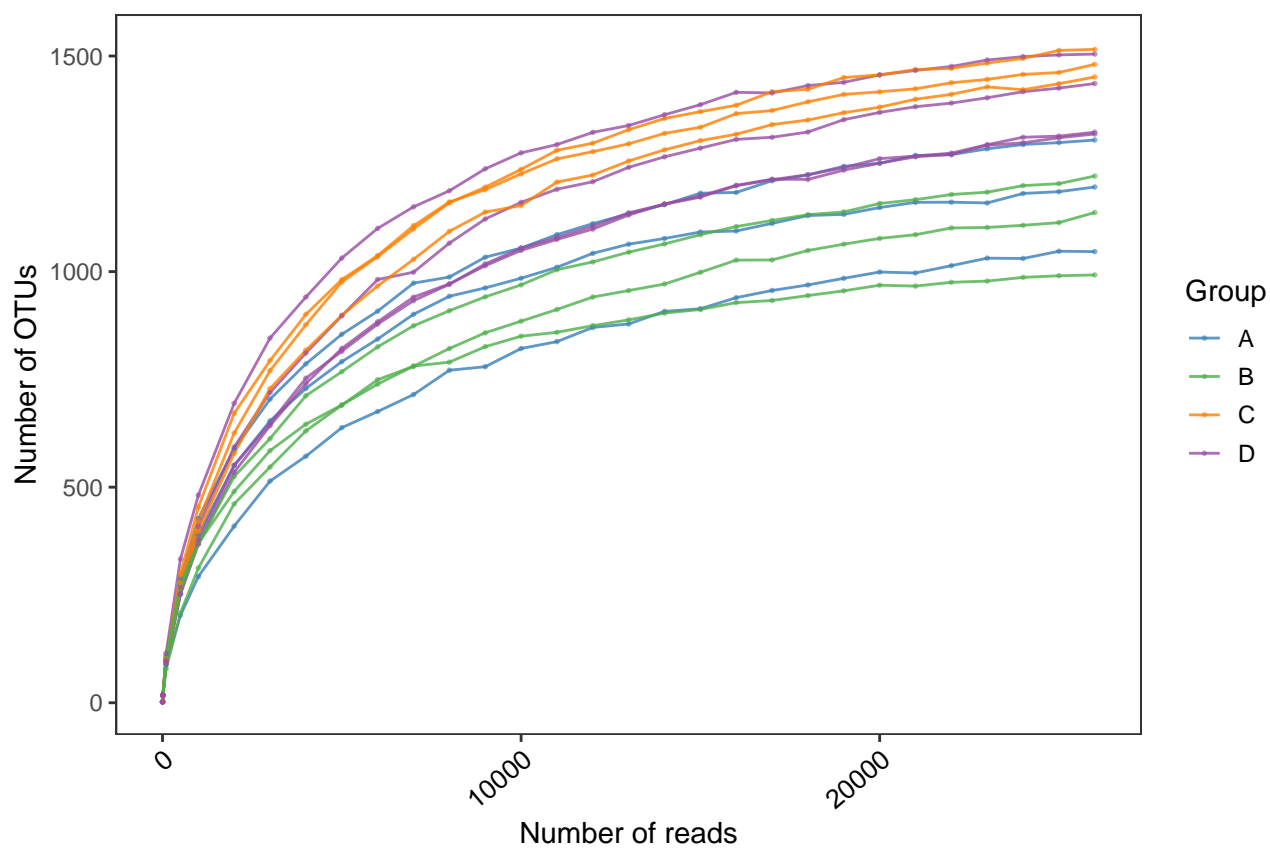

Supplement: Supplementary file 2 [file DataSheet1.zip › 16S rRNA/Images/Rarefaction_curve.pdf]

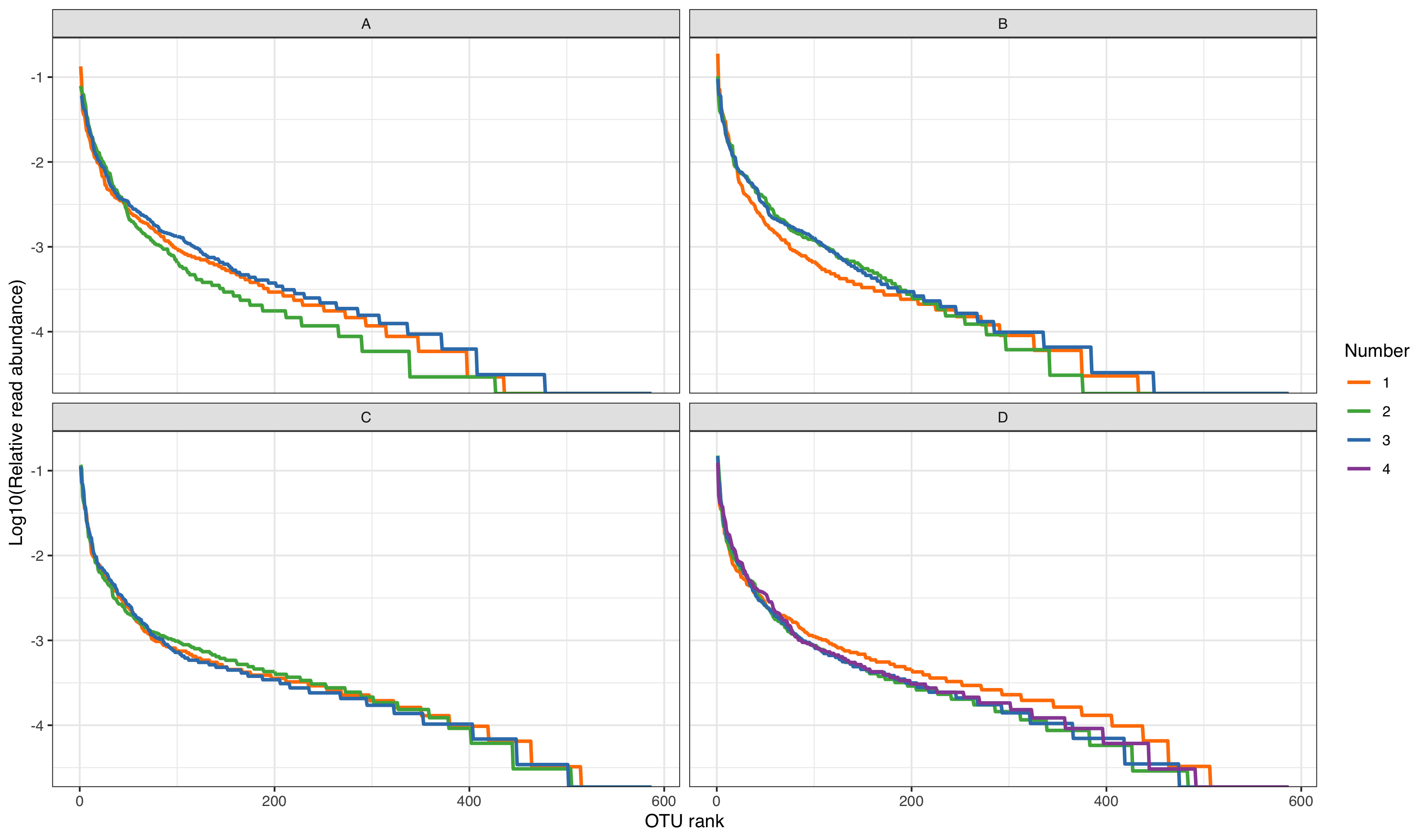

Supplement: Supplementary file 2 [file DataSheet1.zip › 16S rRNA/Images/Rank_abundance_curve_Group.png]

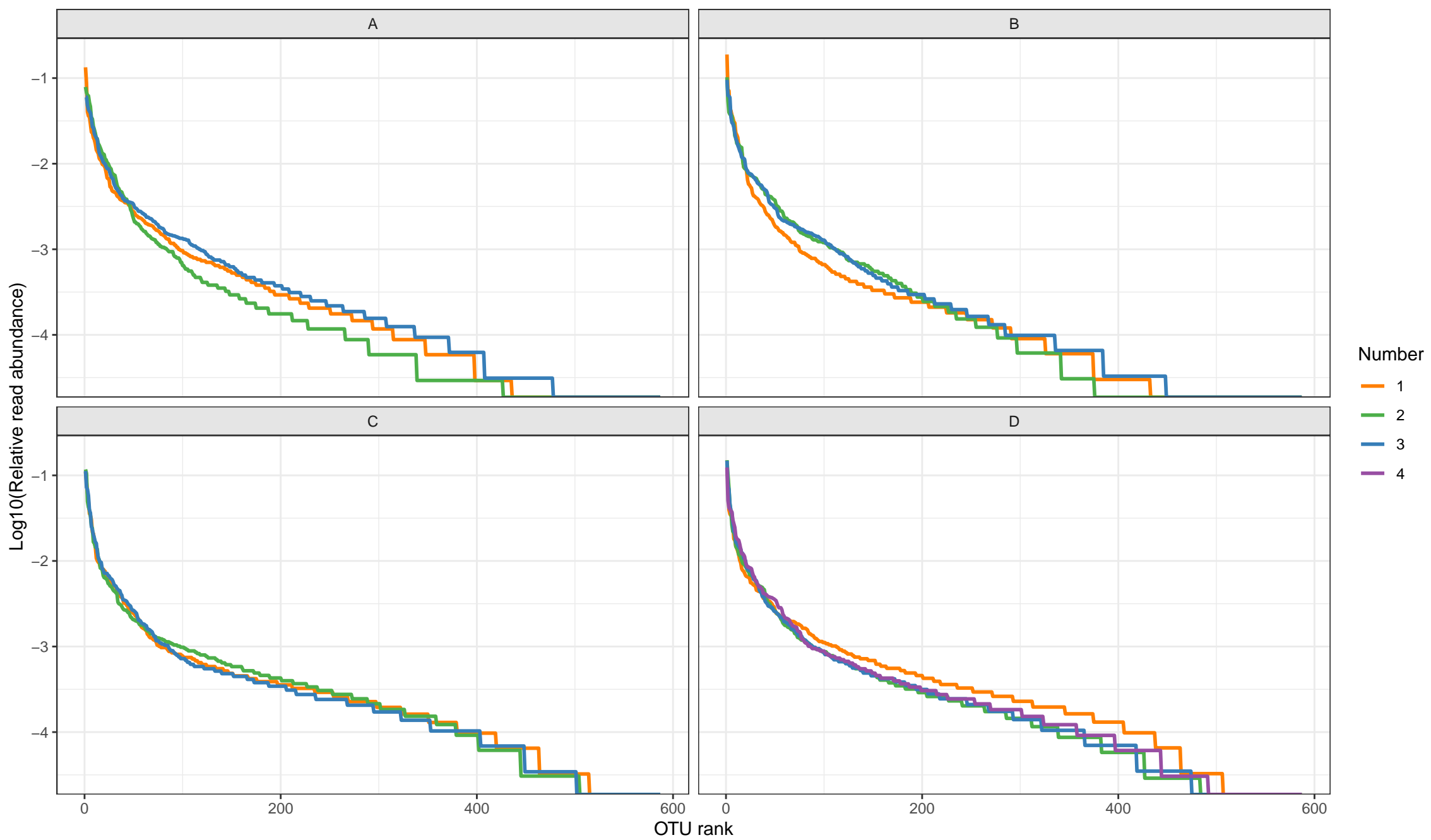

Supplement: Supplementary file 2 [file DataSheet1.zip › 16S rRNA/Images/Rank_abundance_curve_Group.pdf]

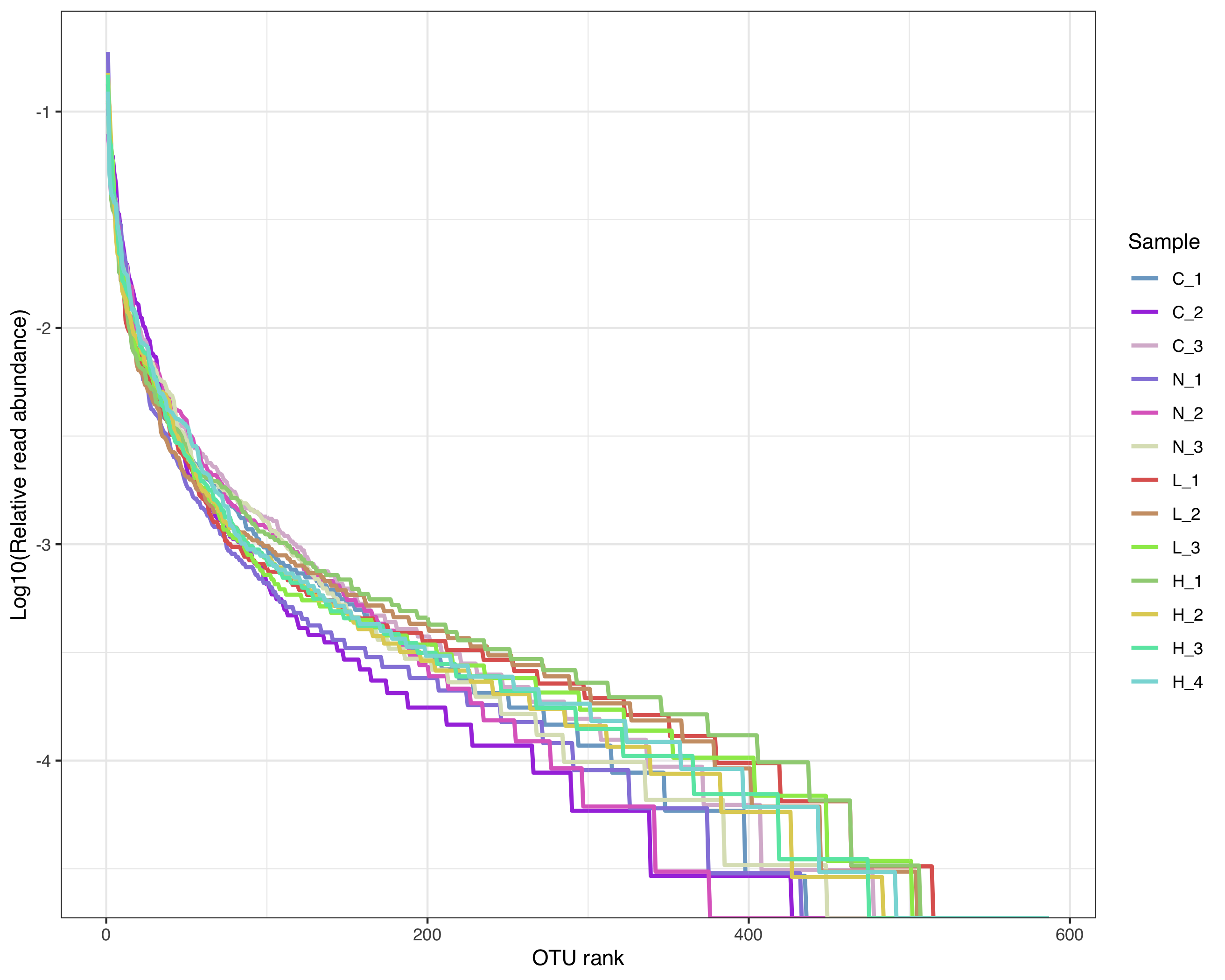

Supplement: Supplementary file 2 [file DataSheet1.zip › 16S rRNA/Images/Rank_abundance_curve.png]

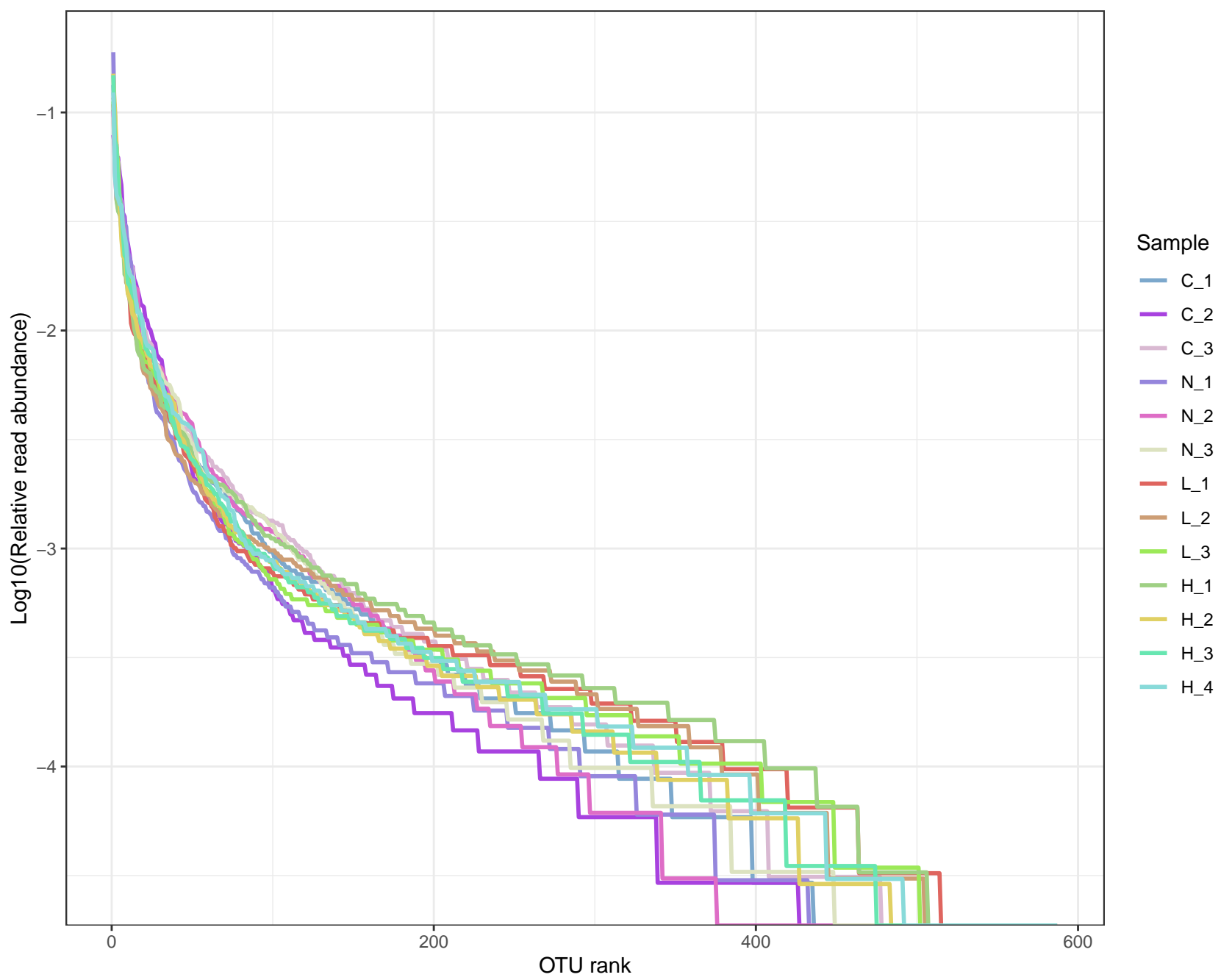

Supplement: Supplementary file 2 [file DataSheet1.zip › 16S rRNA/Images/Rank_abundance_curve.pdf]

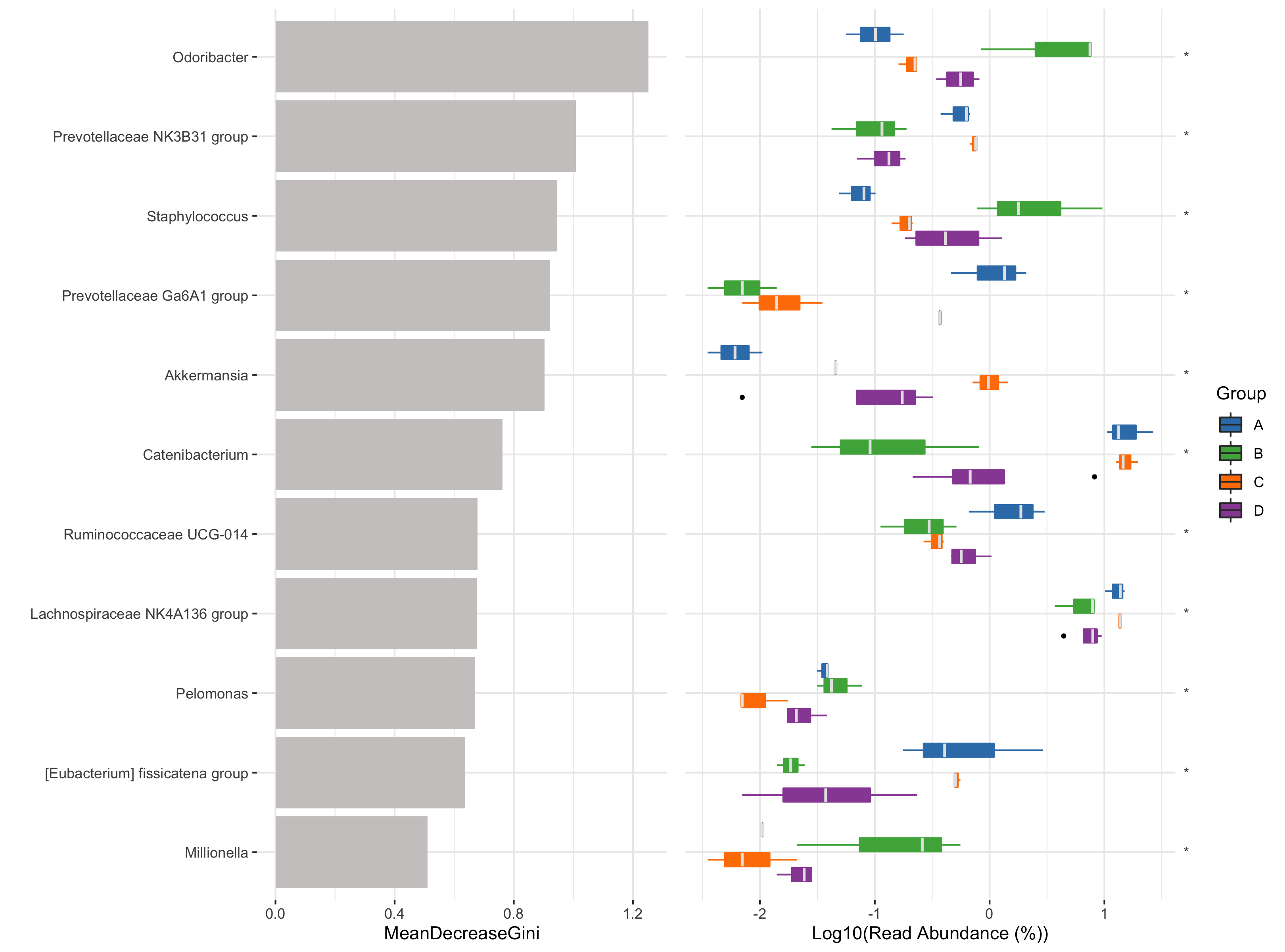

Supplement: Supplementary file 2 [file DataSheet1.zip › 16S rRNA/Images/RandomForest_Gini.png]

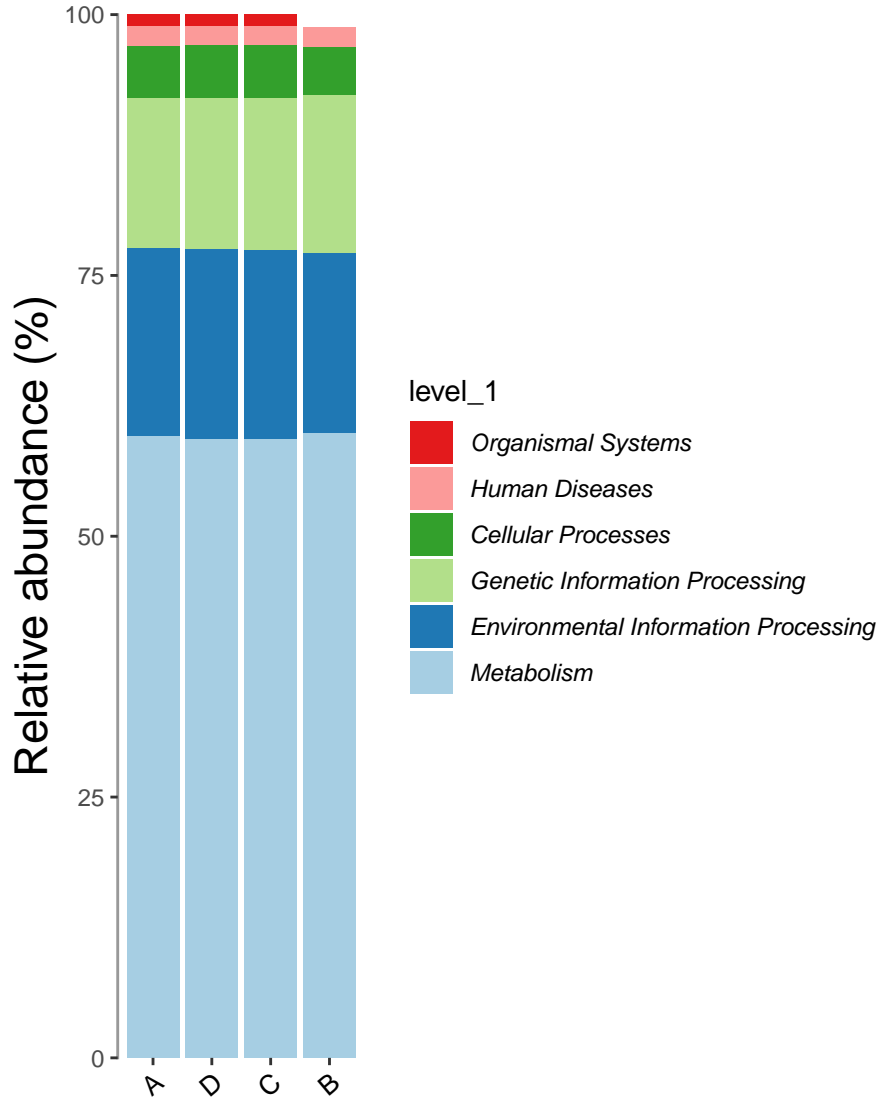

Supplement: Supplementary file 2 [file DataSheet1.zip › 16S rRNA/06.FunctionPrediction/Images/Barplot_level_1_mean.pdf]

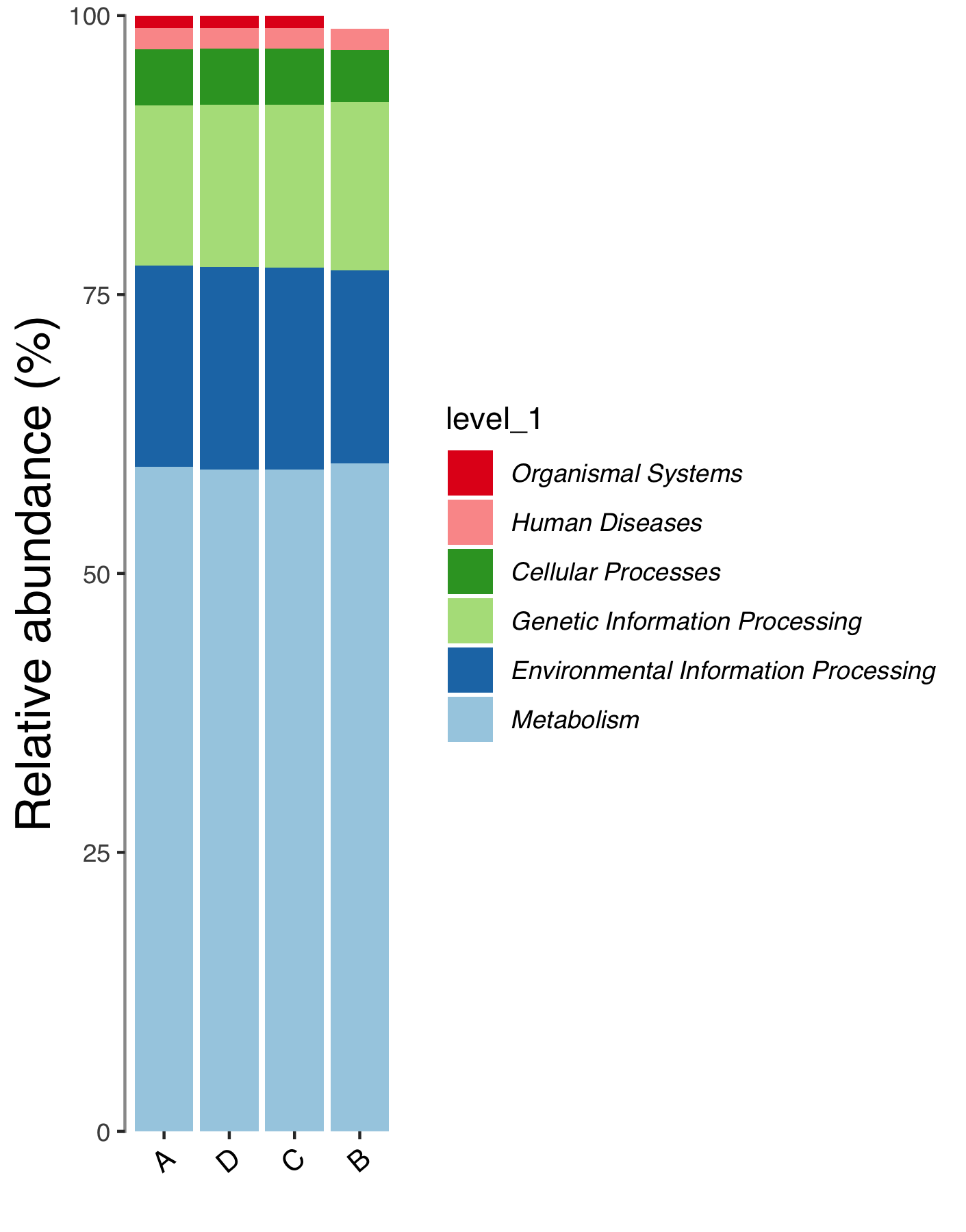

Supplement: Supplementary file 2 [file DataSheet1.zip › 16S rRNA/06.FunctionPrediction/Images/Barplot_level_1_mean.png]

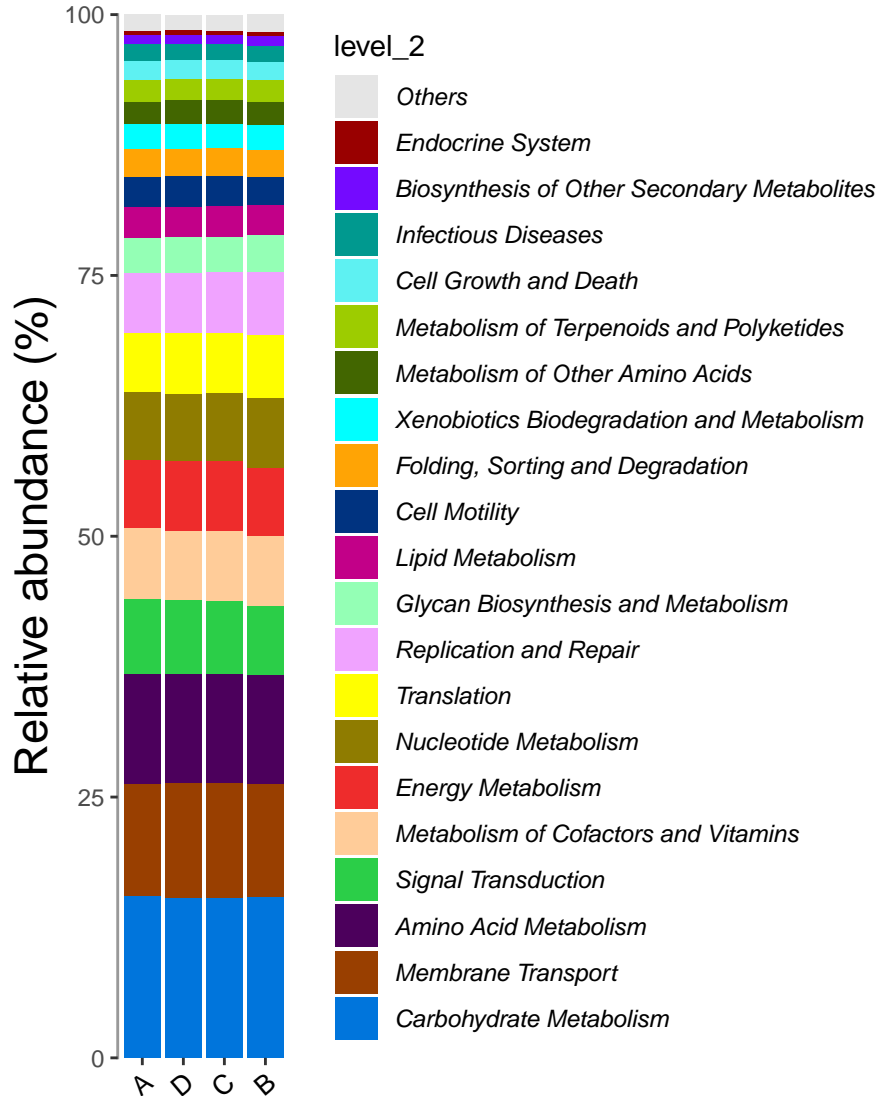

Supplement: Supplementary file 2 [file DataSheet1.zip › 16S rRNA/06.FunctionPrediction/Images/Barplot_level_2_mean.pdf]

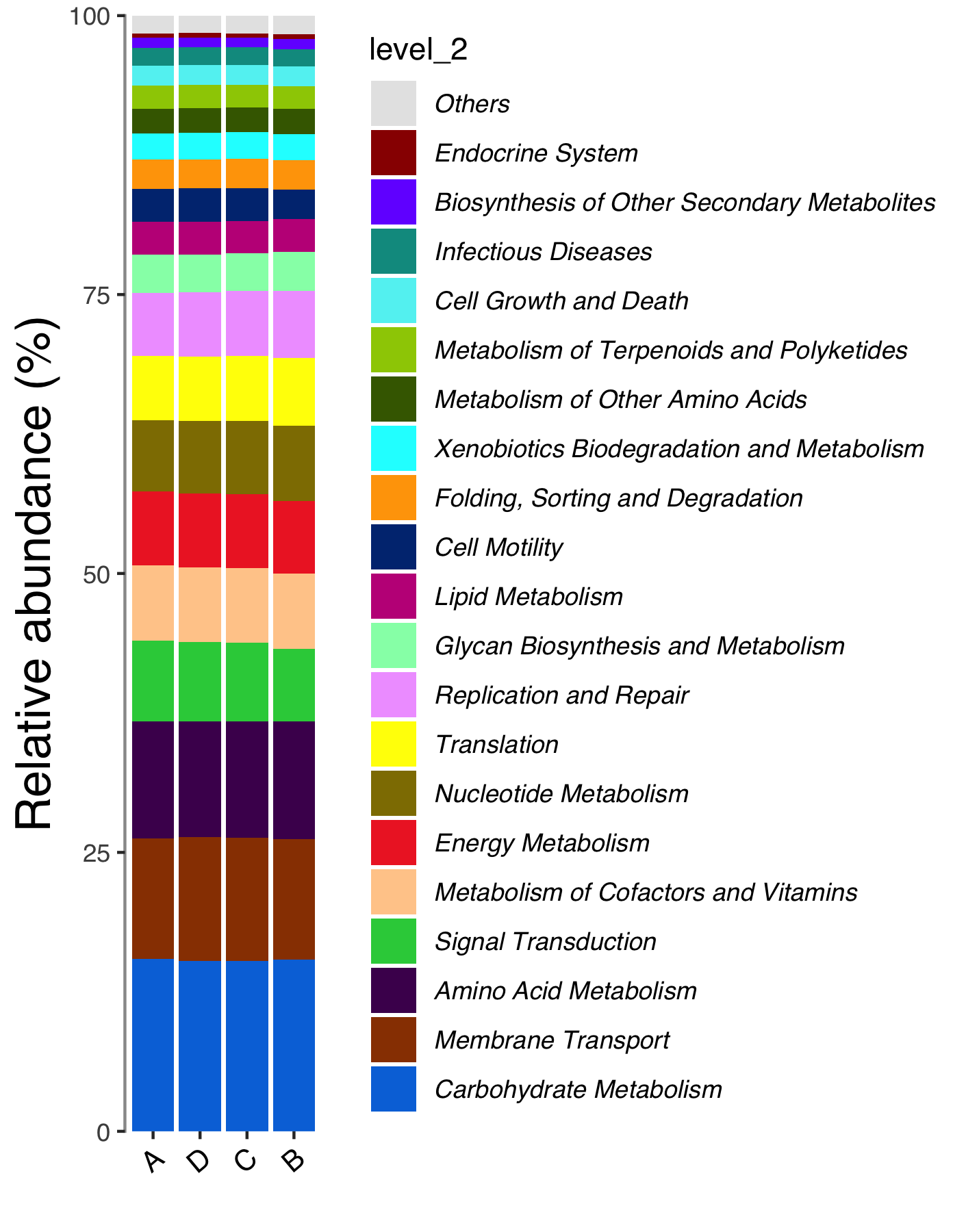

Supplement: Supplementary file 2 [file DataSheet1.zip › 16S rRNA/06.FunctionPrediction/Images/Barplot_level_2_mean.png]

Relative abundance (%)

50  
40  
30  
20  
10  
0

A D C B

level\_3

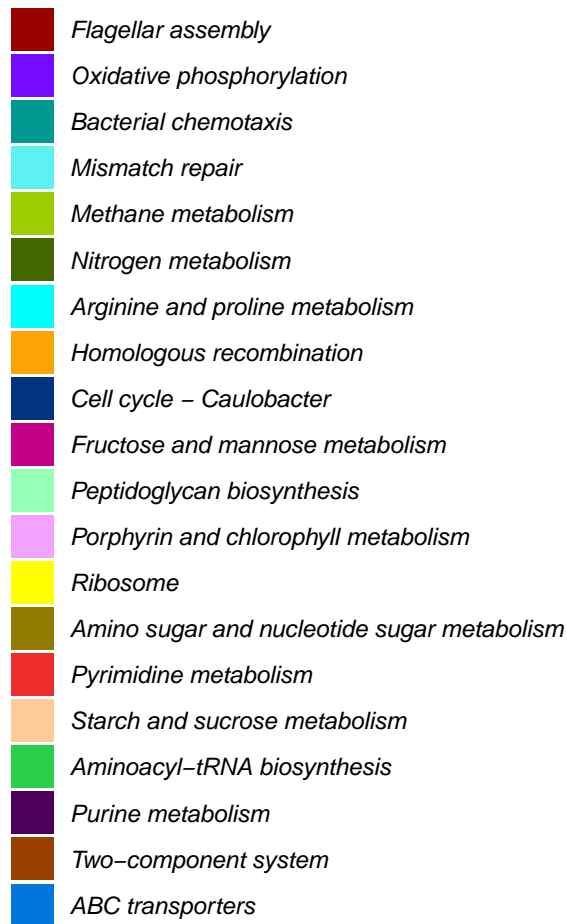

Supplement: Supplementary file 2 [file DataSheet1.zip › 16S rRNA/06.FunctionPrediction/Images/Barplot_level_3_mean.pdf]

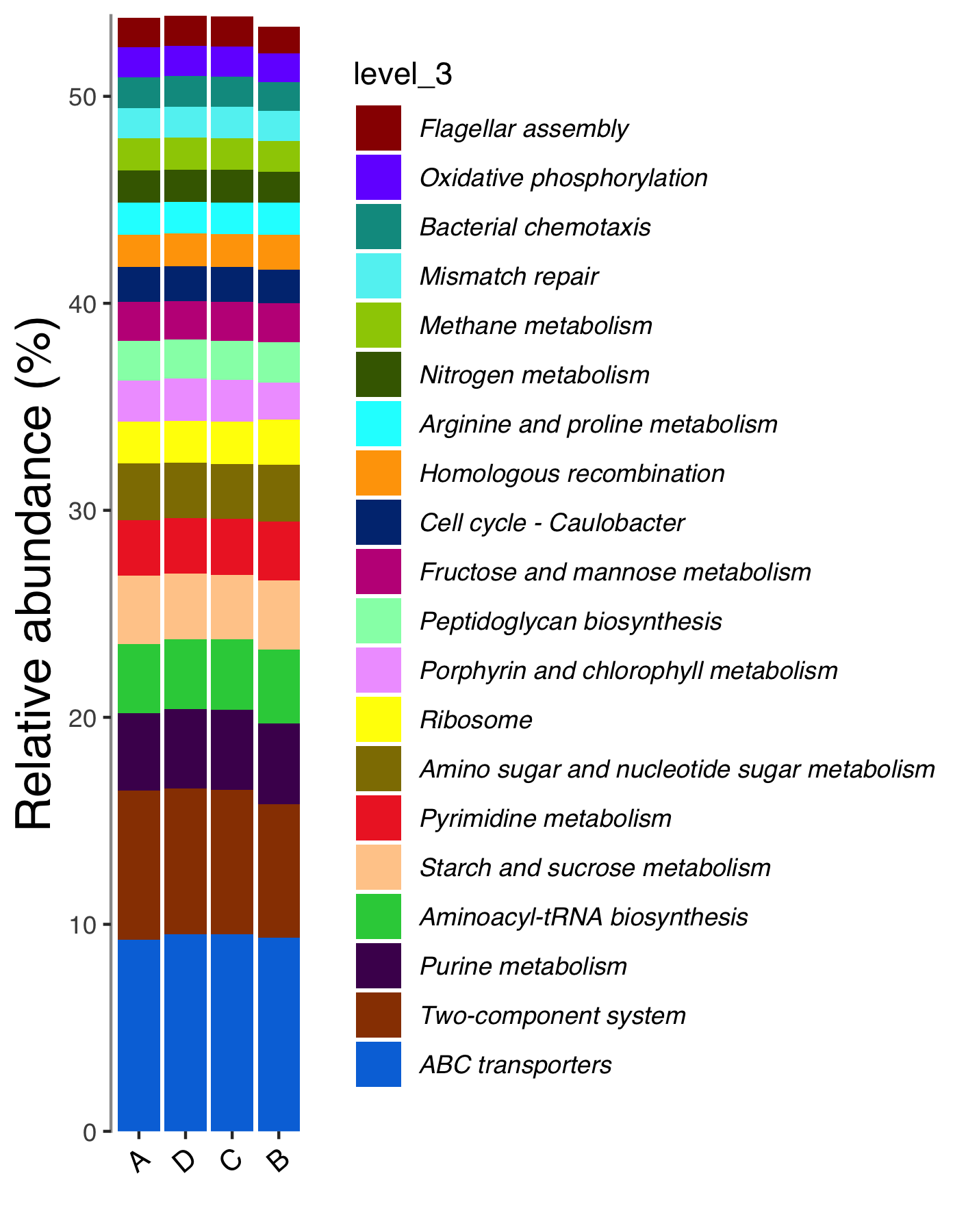

Supplement: Supplementary file 2 [file DataSheet1.zip › 16S rRNA/06.FunctionPrediction/Images/Barplot_level_3_mean.png]

## KEGG level2: Cellular Processes

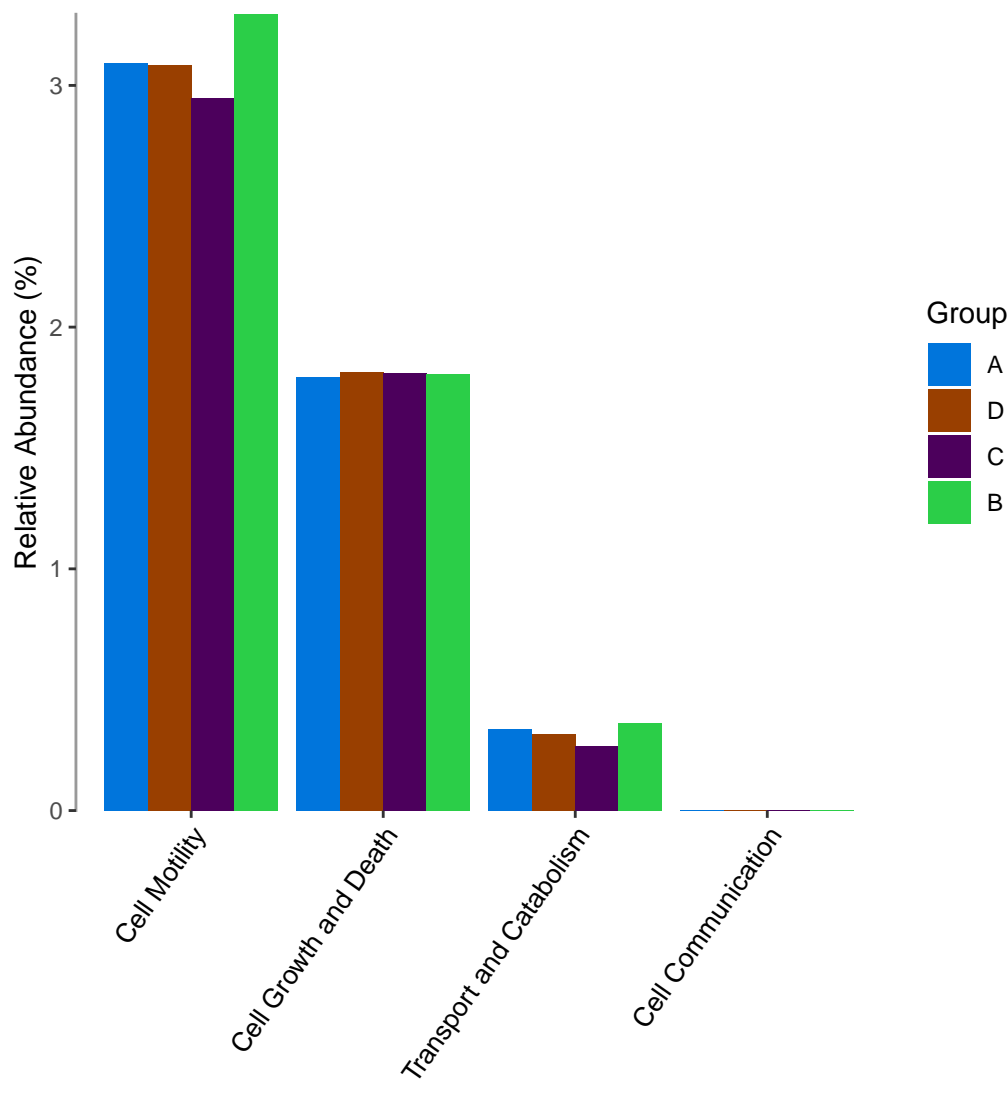

Supplement: Supplementary file 2 [file DataSheet1.zip › 16S rRNA/06.FunctionPrediction/Images/KEGG_level2_Cellular Processes.pdf]

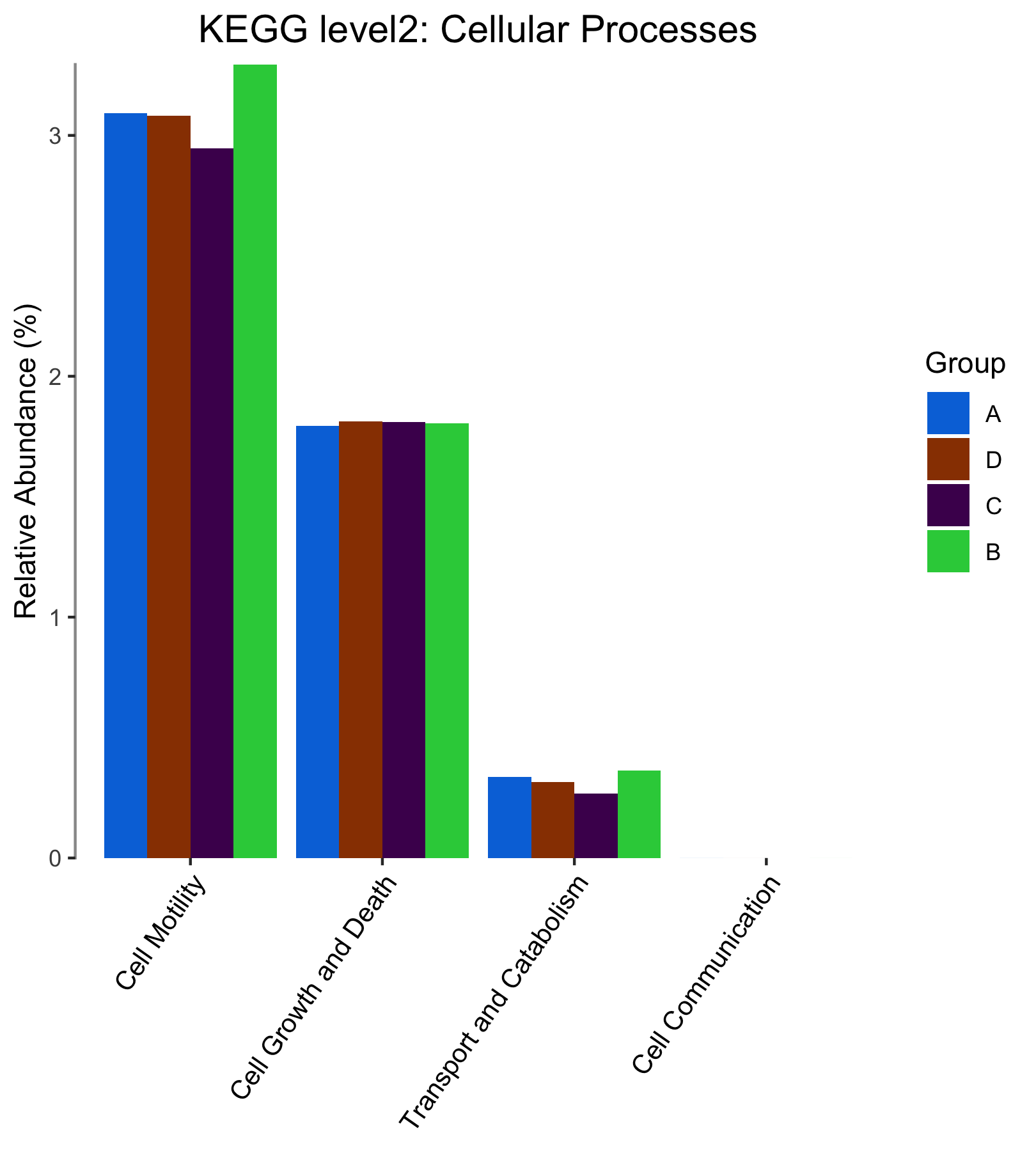

Supplement: Supplementary file 2 [file DataSheet1.zip › 16S rRNA/06.FunctionPrediction/Images/KEGG_level2_Cellular Processes.png]

# KEGG level2: Environmental Information Processing

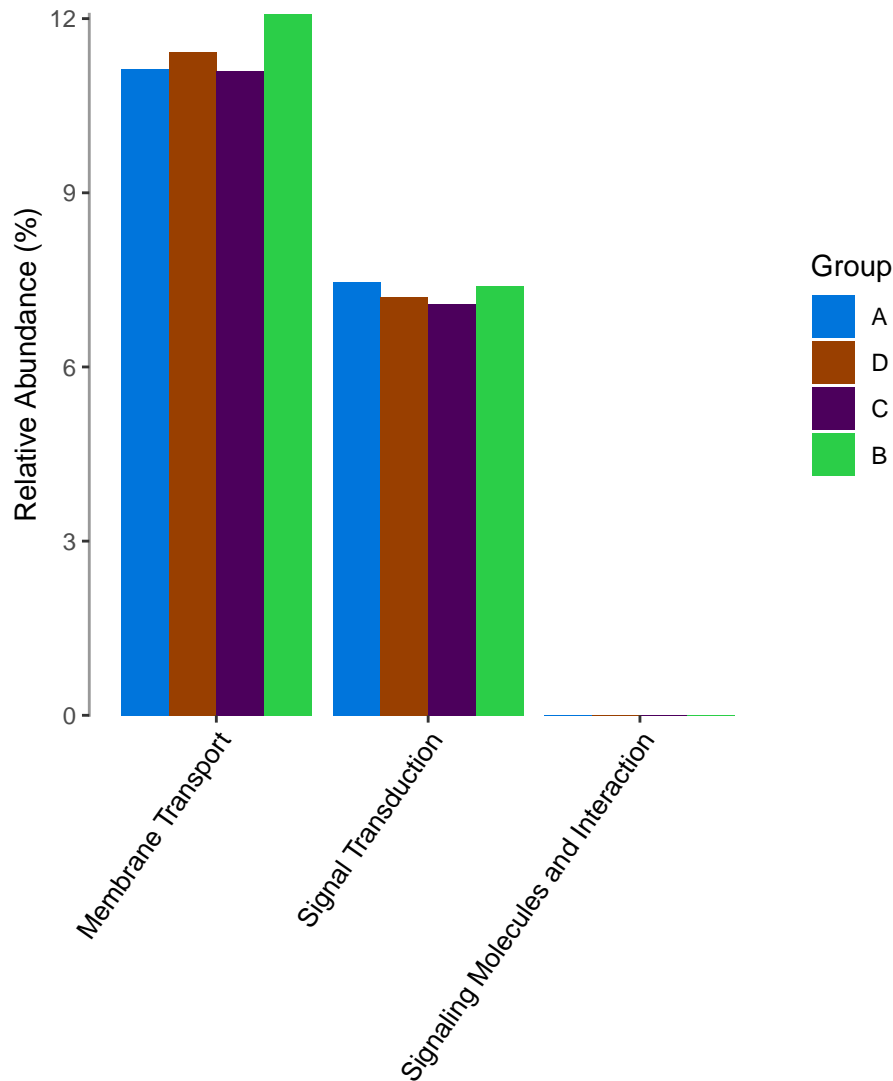

Supplement: Supplementary file 2 [file DataSheet1.zip › 16S rRNA/06.FunctionPrediction/Images/KEGG_level2_Environmental Information Processing.pdf]

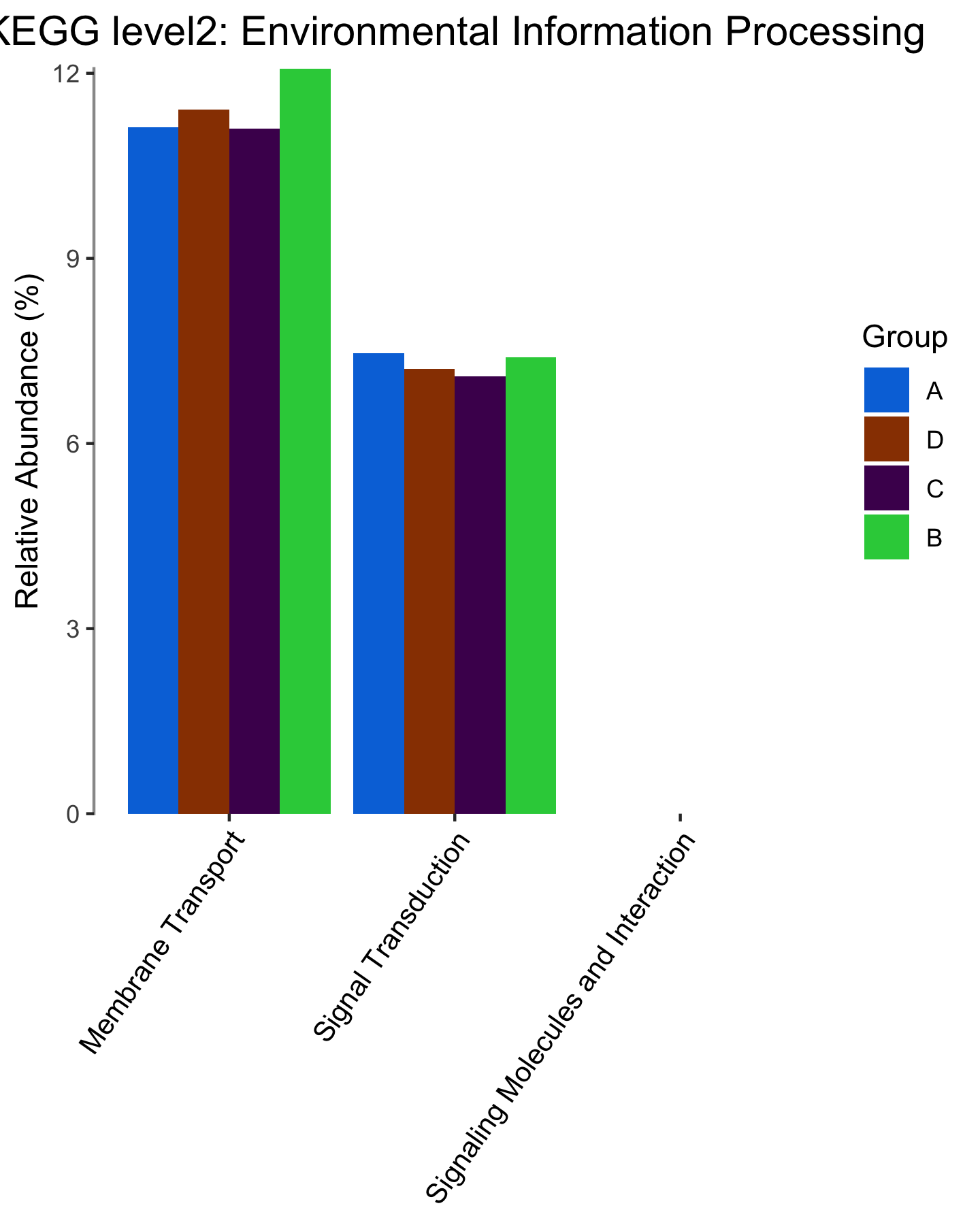

Supplement: Supplementary file 2 [file DataSheet1.zip › 16S rRNA/06.FunctionPrediction/Images/KEGG_level2_Environmental Information Processing.png]

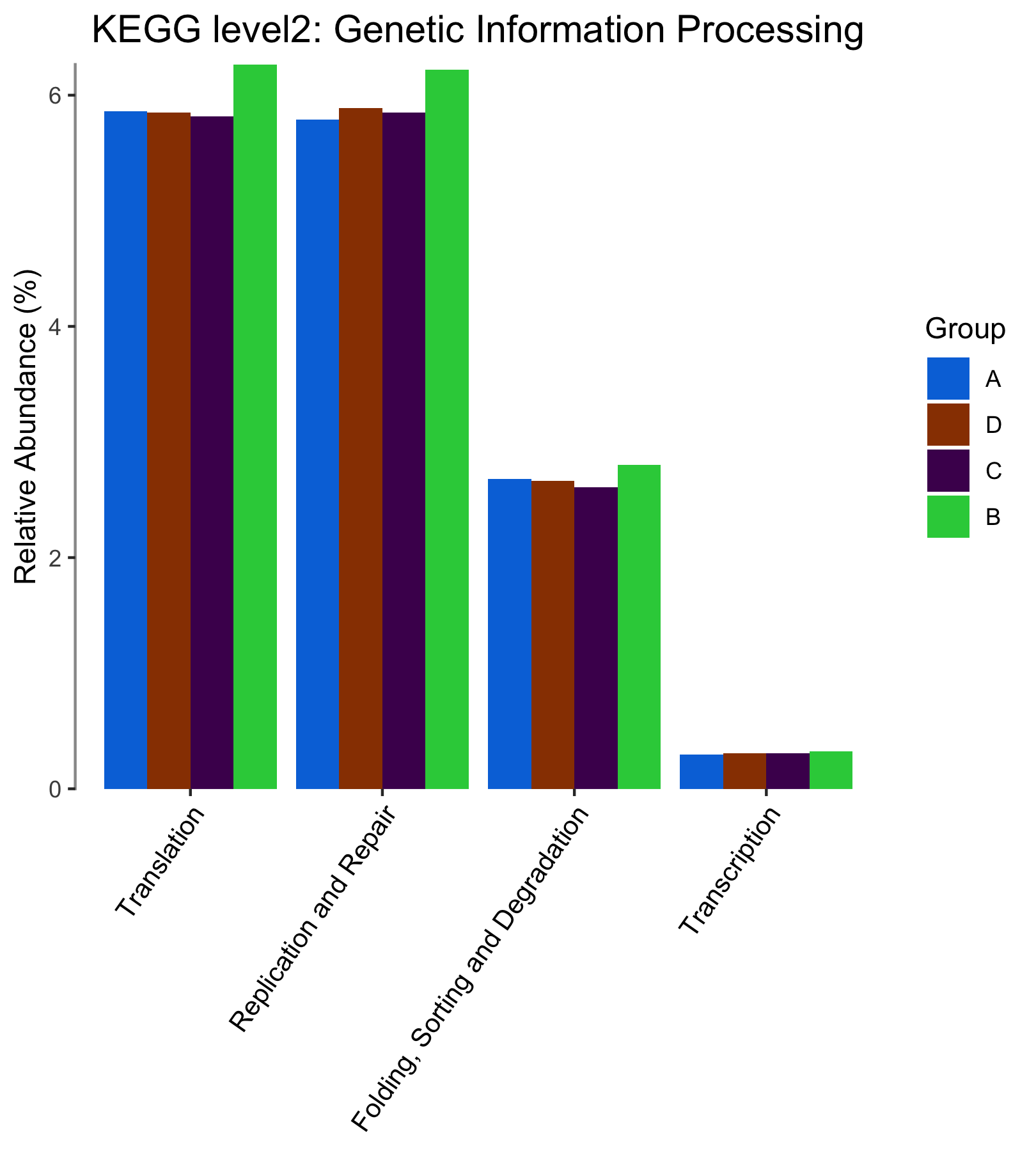

Supplement: Supplementary file 2 [file DataSheet1.zip › 16S rRNA/06.FunctionPrediction/Images/KEGG_level2_Genetic Information Processing.png]

## KEGG level2: Human Diseases

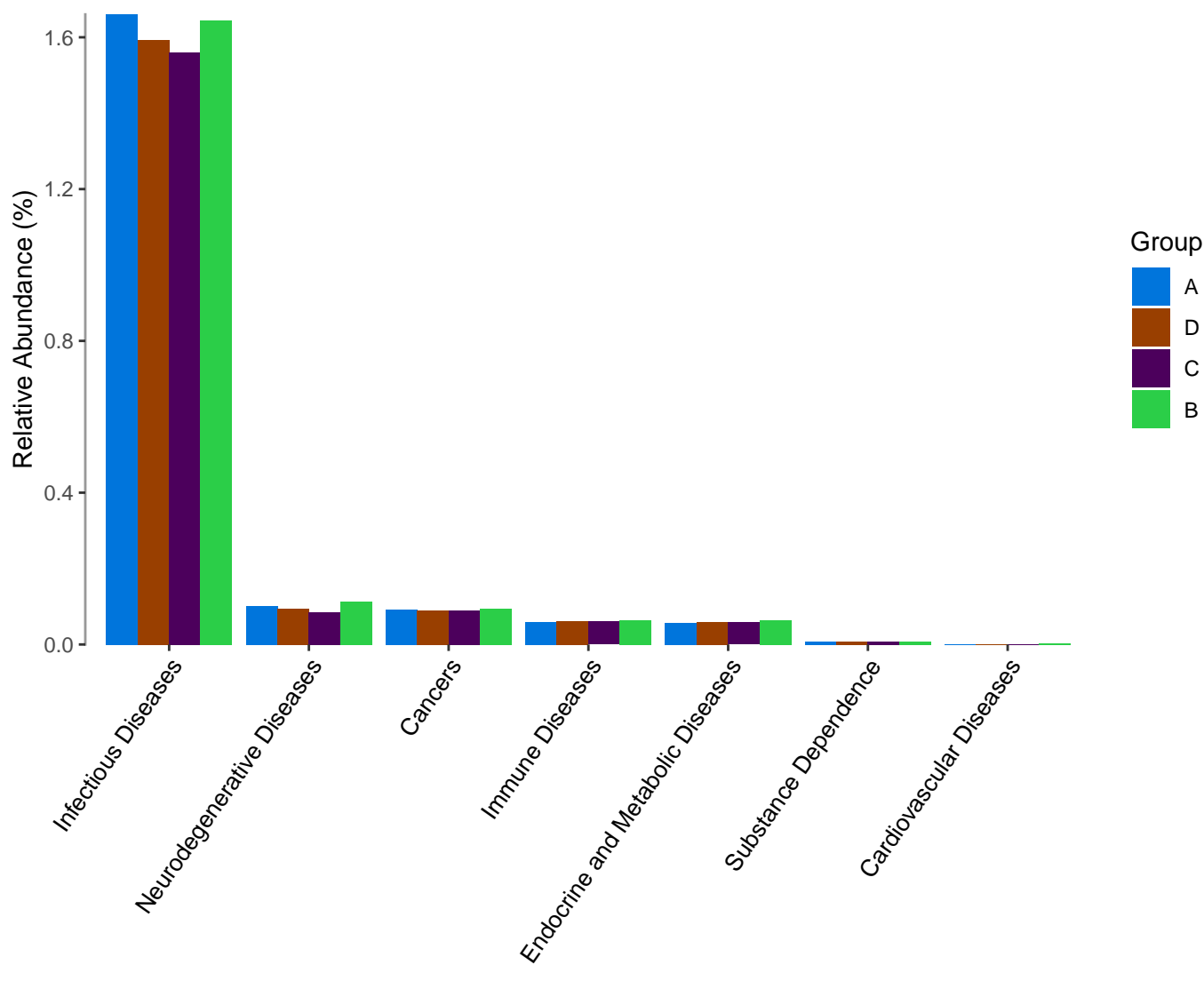

Supplement: Supplementary file 2 [file DataSheet1.zip › 16S rRNA/06.FunctionPrediction/Images/KEGG_level2_Human Diseases.pdf]

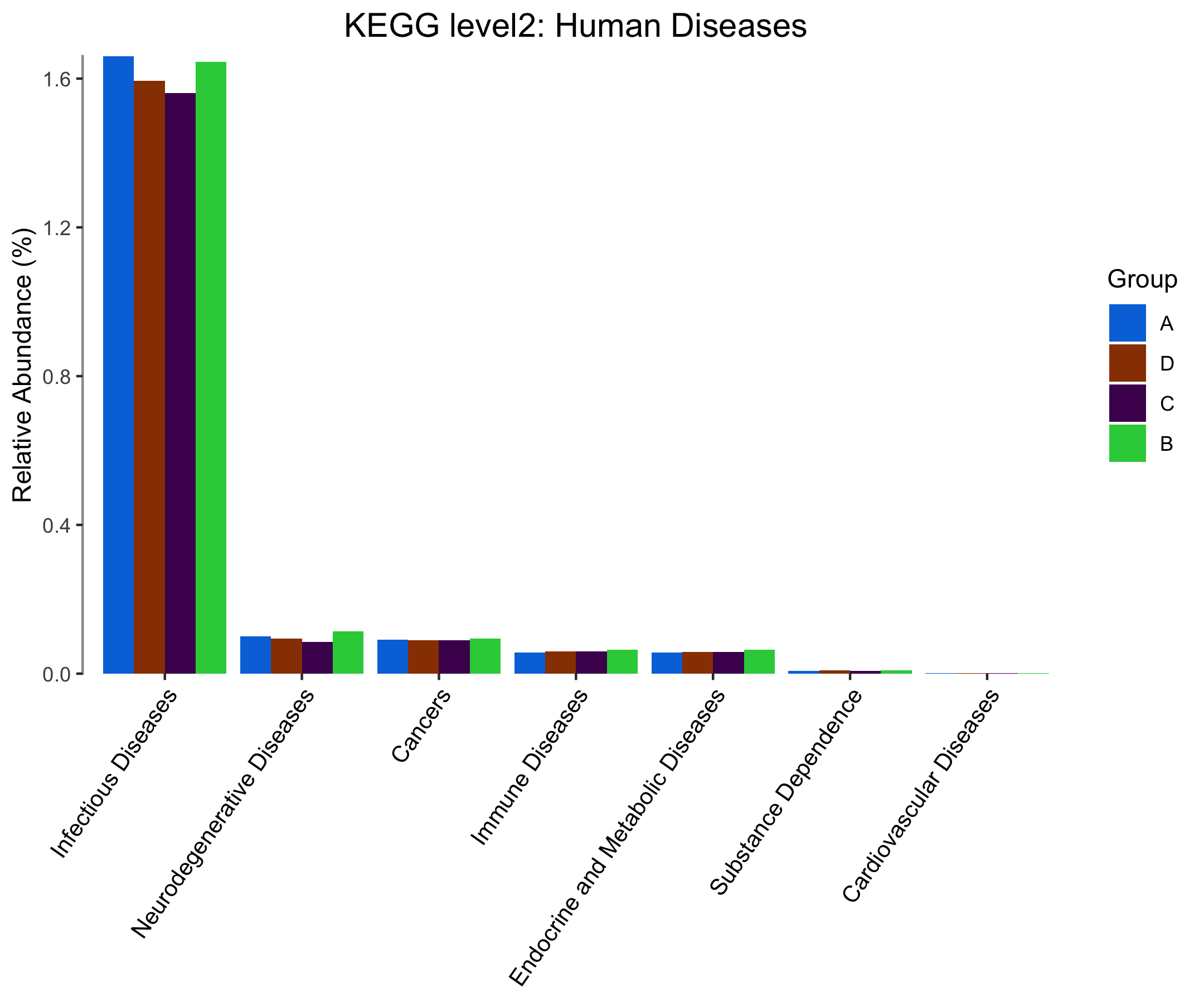

Supplement: Supplementary file 2 [file DataSheet1.zip › 16S rRNA/06.FunctionPrediction/Images/KEGG_level2_Human Diseases.png]

# KEGG level2: Metabolism

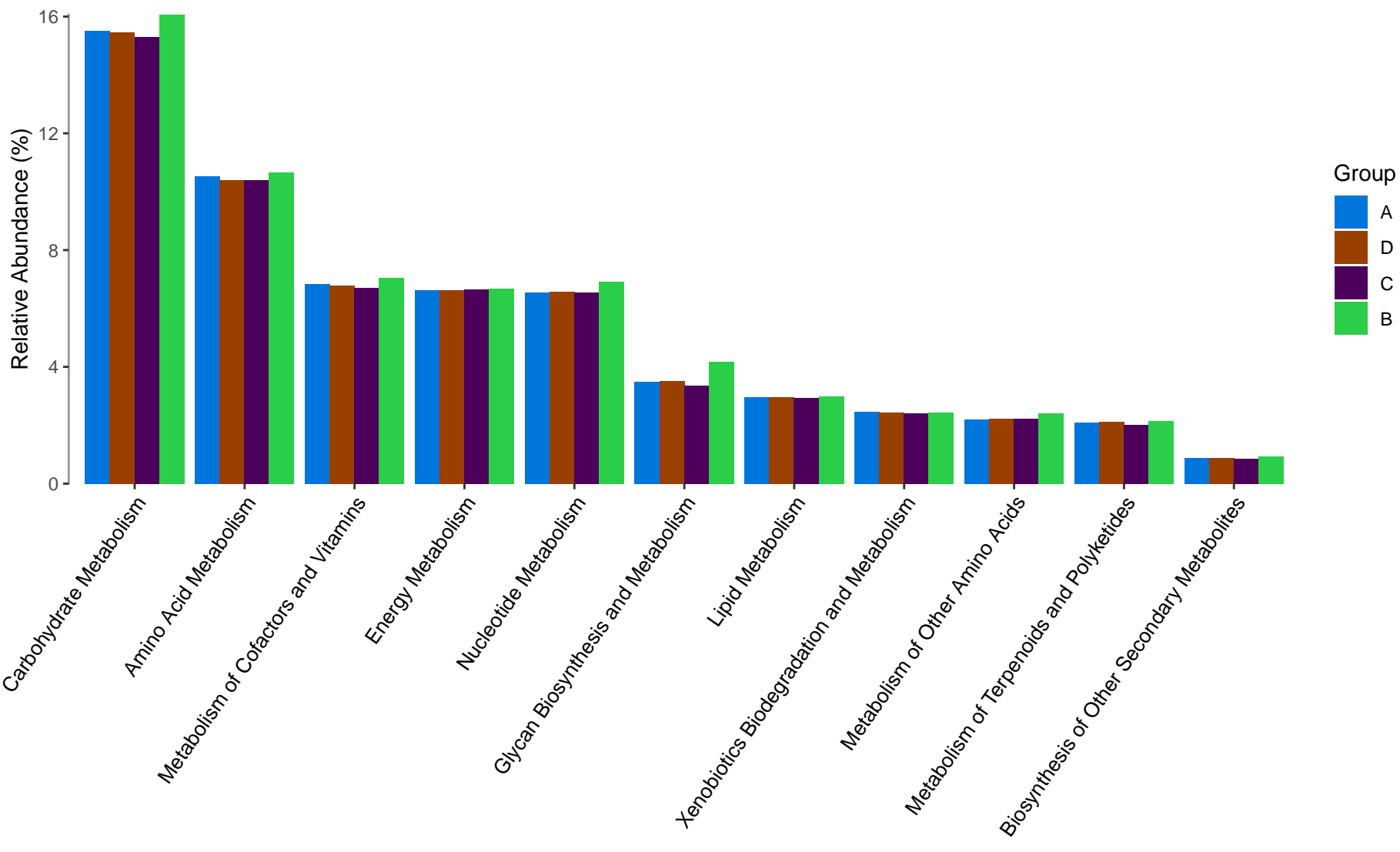

Supplement: Supplementary file 2 [file DataSheet1.zip › 16S rRNA/06.FunctionPrediction/Images/KEGG_level2_Metabolism.pdf]

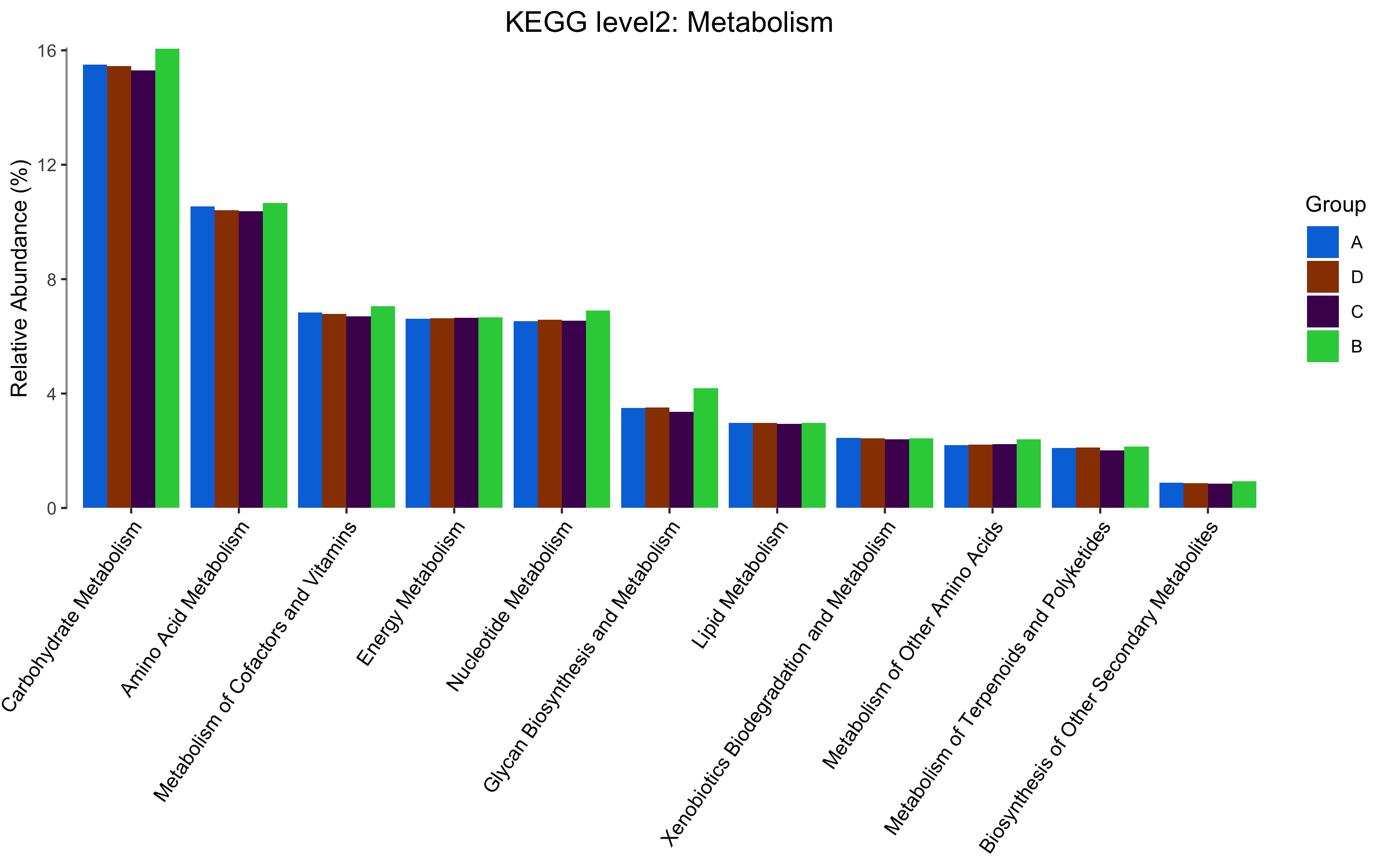

Supplement: Supplementary file 2 [file DataSheet1.zip › 16S rRNA/06.FunctionPrediction/Images/KEGG_level2_Metabolism.png]

## KEGG level2: Organismal Systems

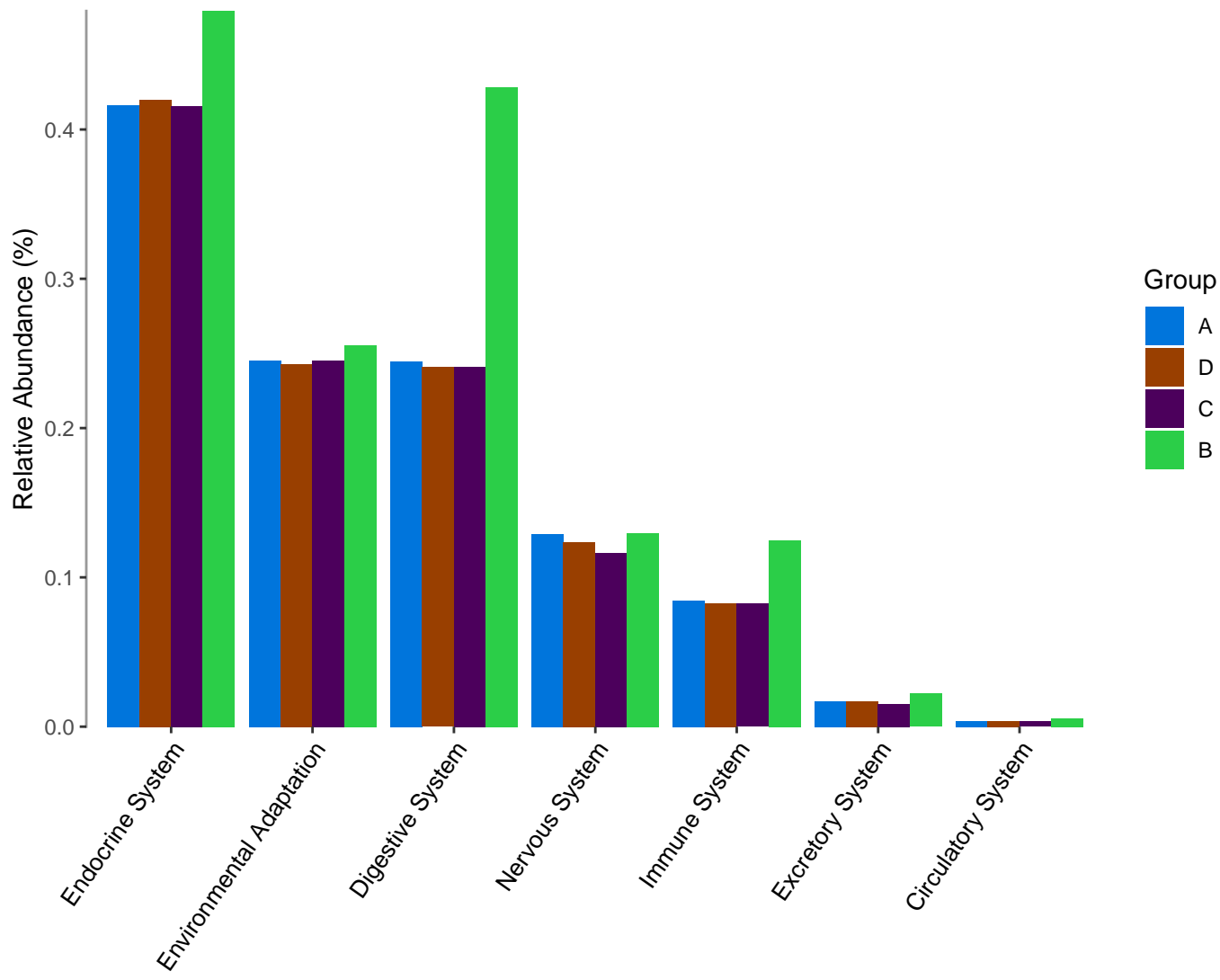

Supplement: Supplementary file 2 [file DataSheet1.zip › 16S rRNA/06.FunctionPrediction/Images/KEGG_level2_Organismal Systems.pdf]

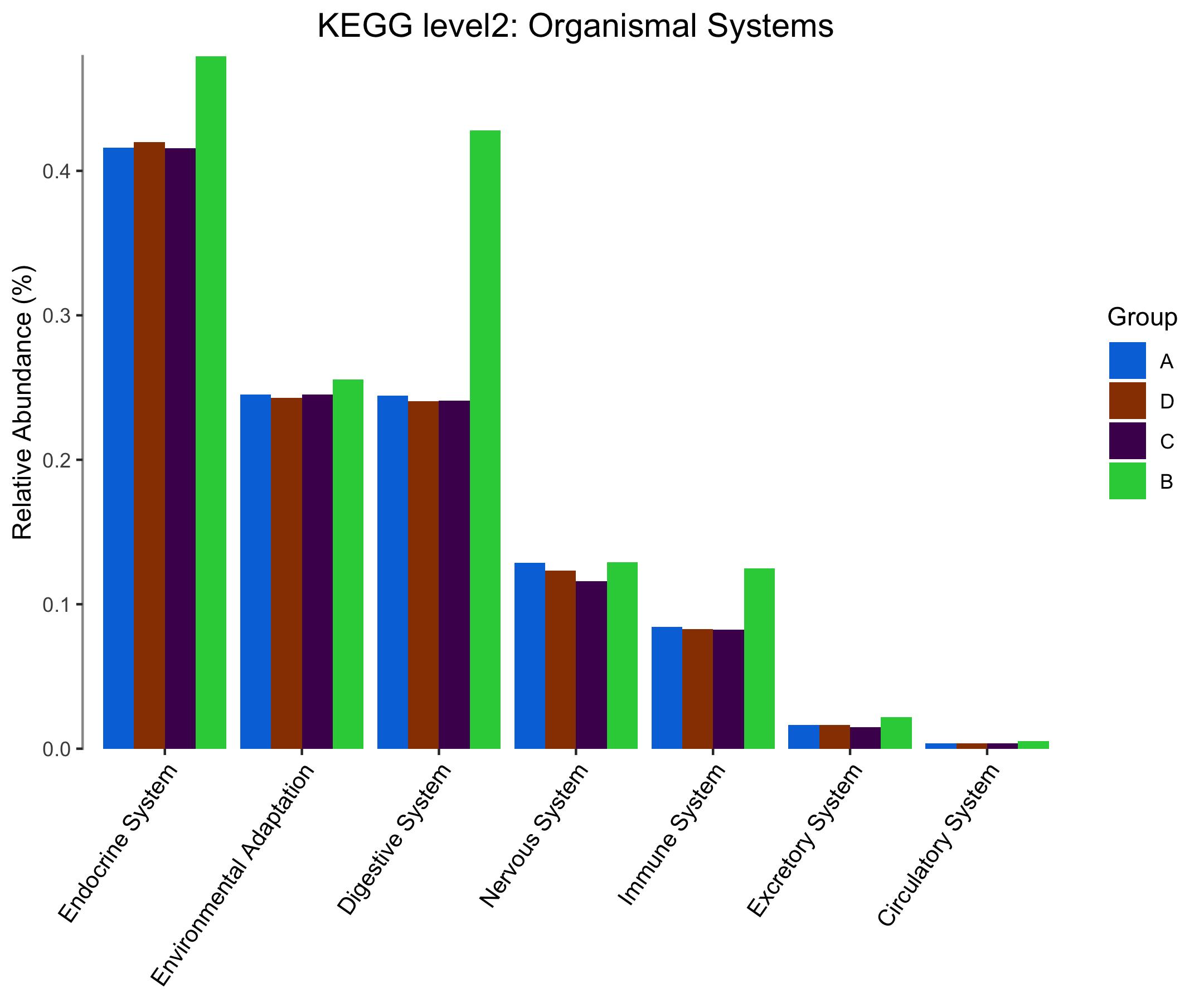

Supplement: Supplementary file 2 [file DataSheet1.zip › 16S rRNA/06.FunctionPrediction/Images/KEGG_level2_Organismal Systems.png]

# Alanine, aspartate and glutamate metabolism

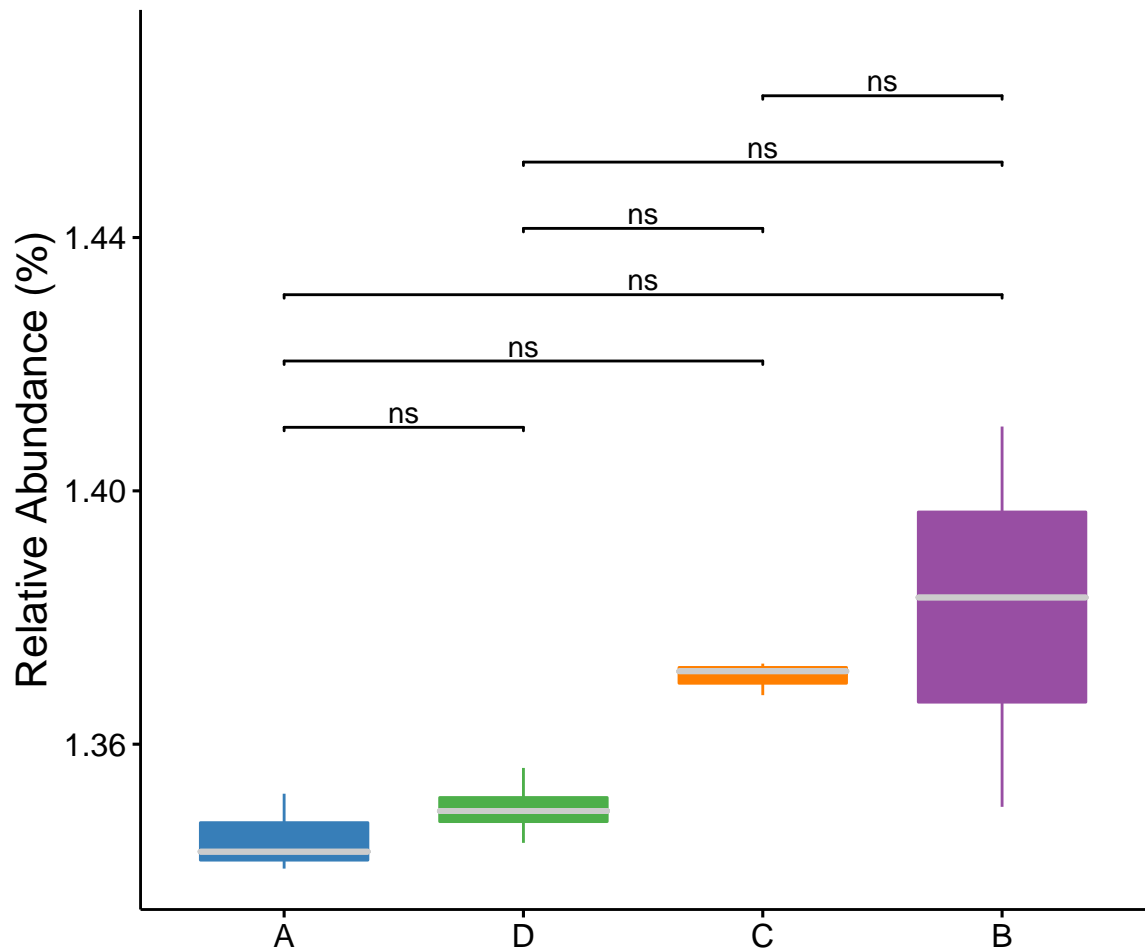

Supplement: Supplementary file 2 [file DataSheet1.zip › 16S rRNA/06.FunctionPrediction/Images/KEGG_level3_Alanine, aspartate and glutamate metabolism.pdf]

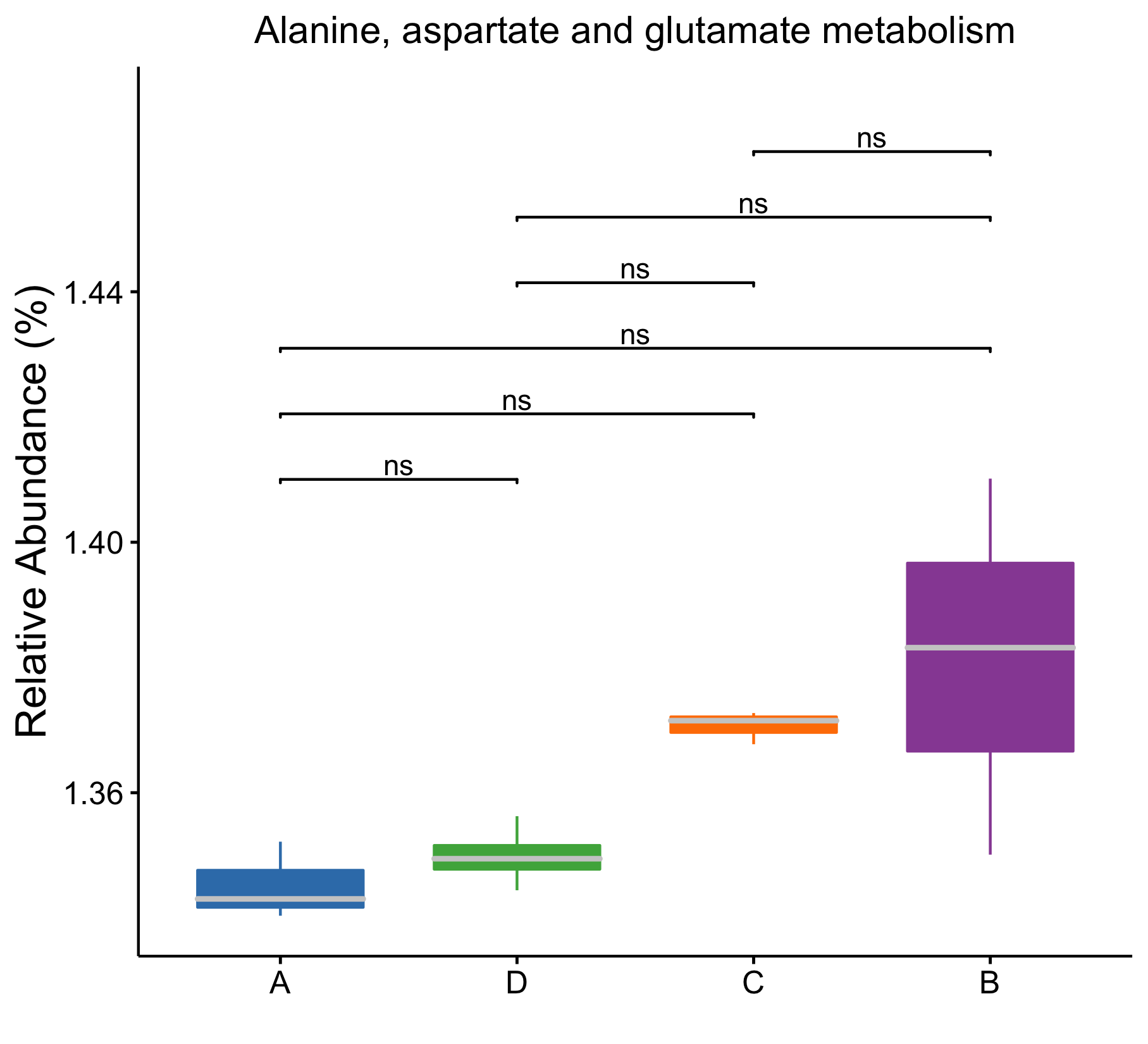

Supplement: Supplementary file 2 [file DataSheet1.zip › 16S rRNA/06.FunctionPrediction/Images/KEGG_level3_Alanine, aspartate and glutamate metabolism.png]

# Arginine and proline metabolism

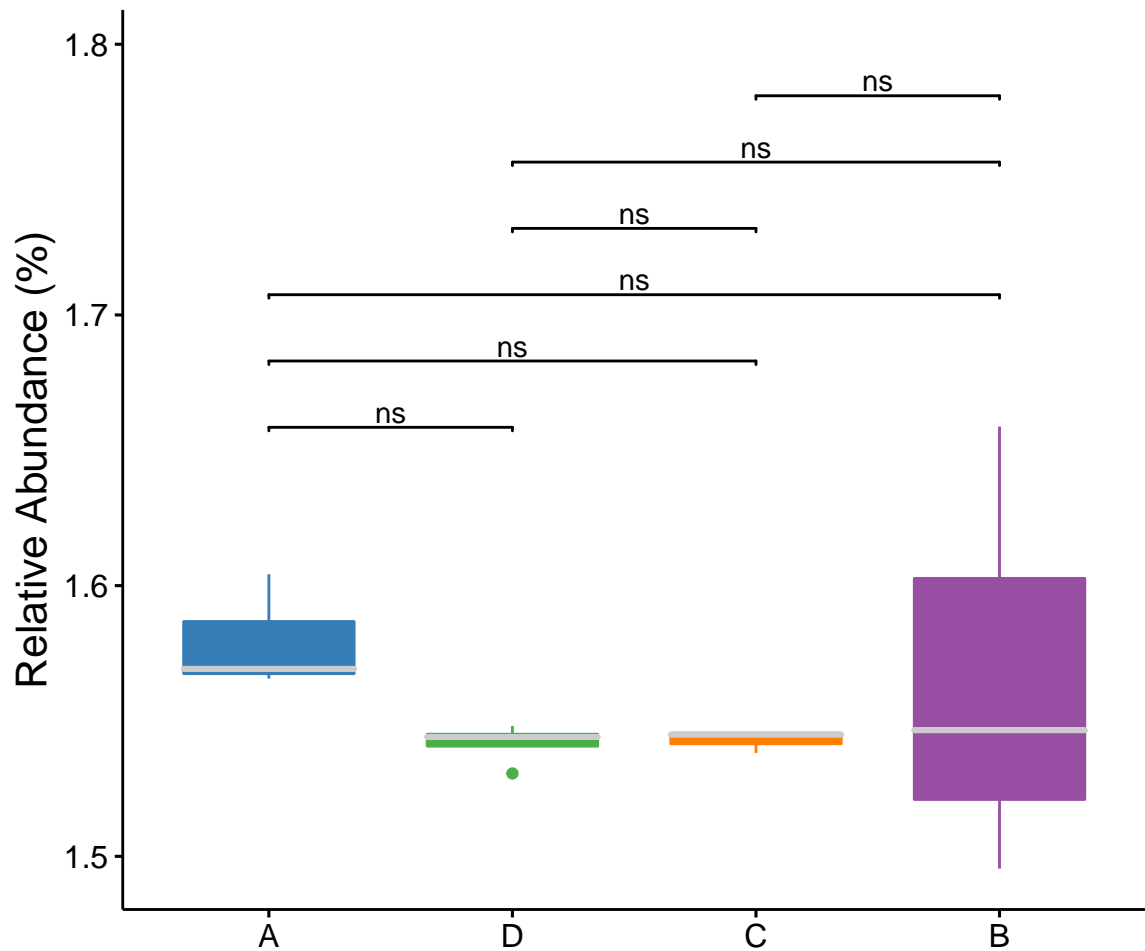

Supplement: Supplementary file 2 [file DataSheet1.zip › 16S rRNA/06.FunctionPrediction/Images/KEGG_level3_Arginine and proline metabolism.pdf]

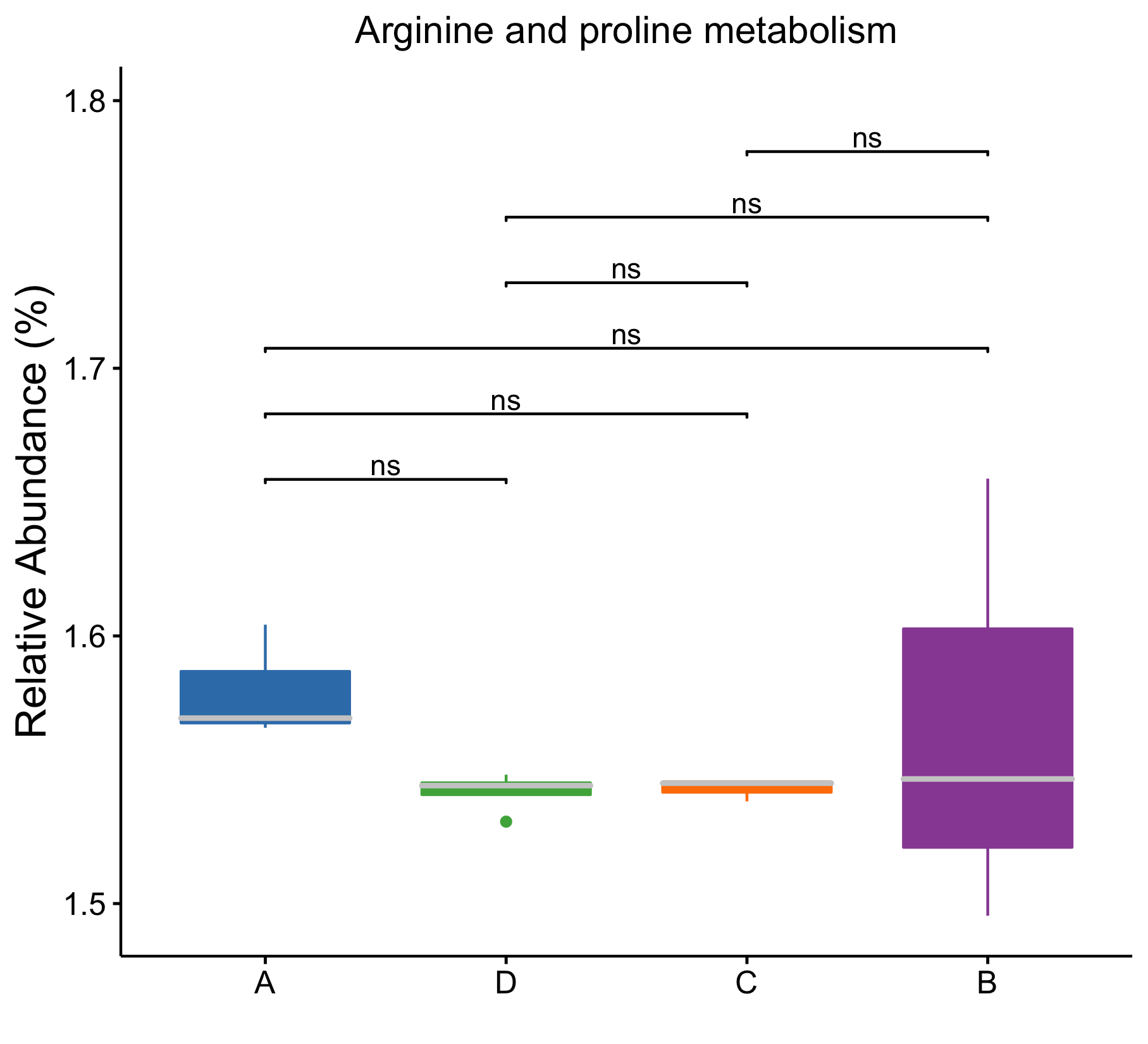

Supplement: Supplementary file 2 [file DataSheet1.zip › 16S rRNA/06.FunctionPrediction/Images/KEGG_level3_Arginine and proline metabolism.png]

# Cysteine and methionine metabolism

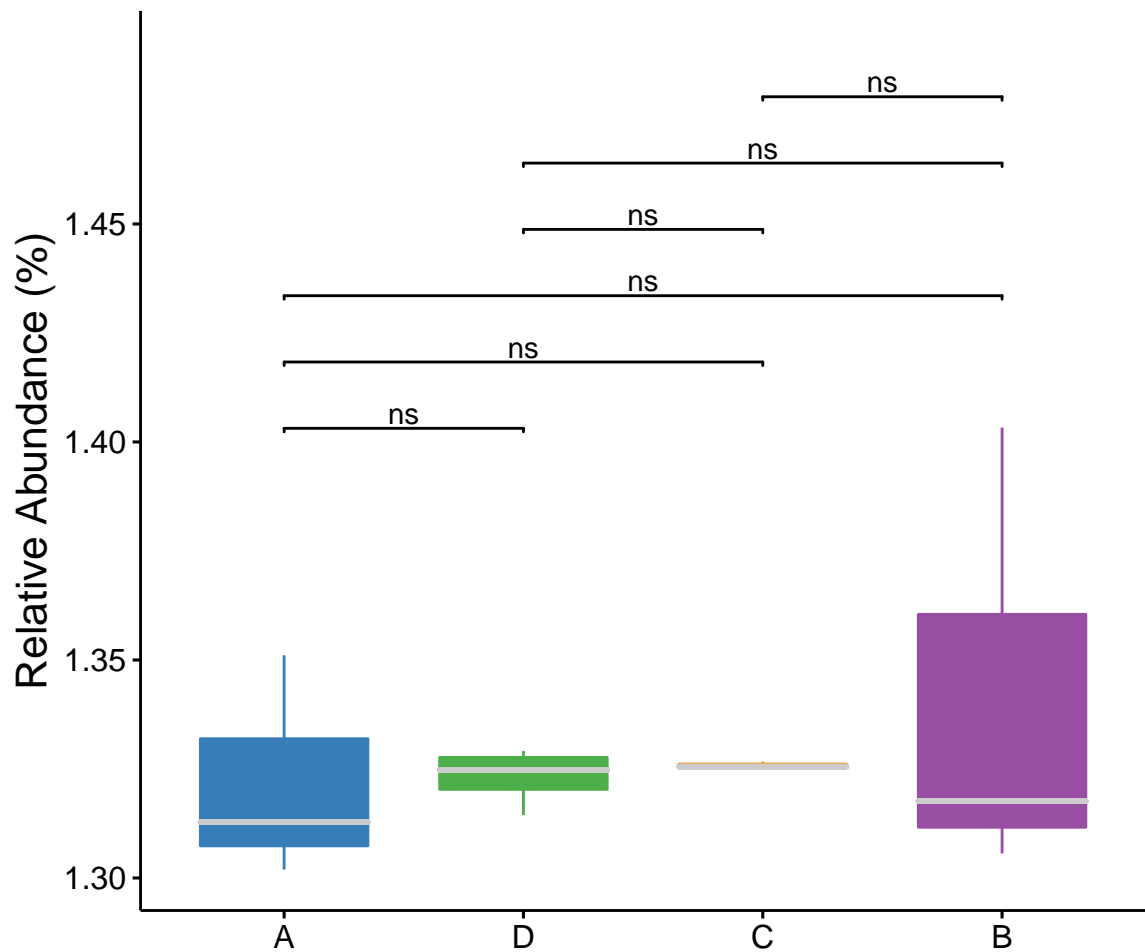

Supplement: Supplementary file 2 [file DataSheet1.zip › 16S rRNA/06.FunctionPrediction/Images/KEGG_level3_Cysteine and methionine metabolism.pdf]

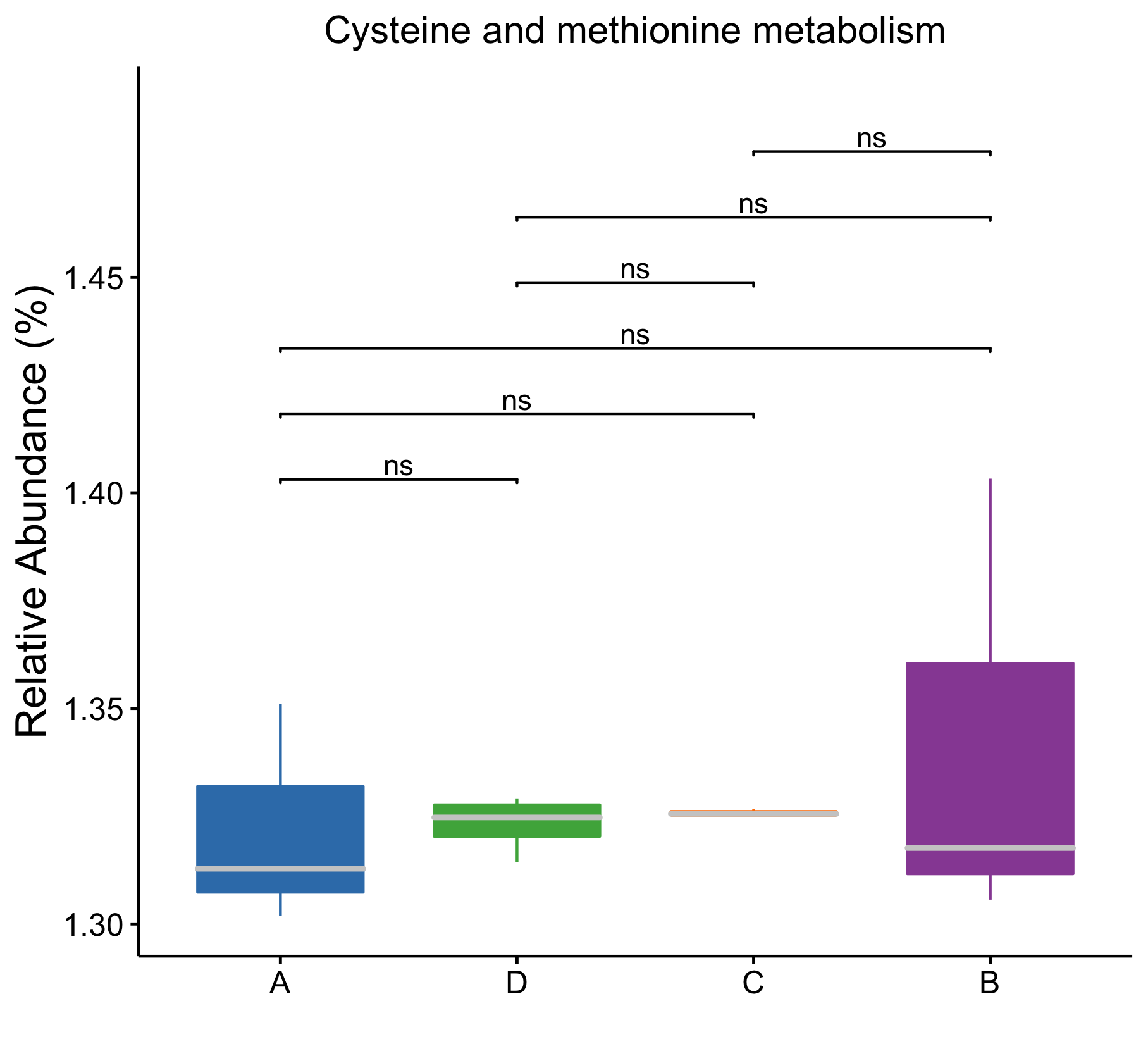

Supplement: Supplementary file 2 [file DataSheet1.zip › 16S rRNA/06.FunctionPrediction/Images/KEGG_level3_Cysteine and methionine metabolism.png]

# Glycine, serine and threonine metabolism

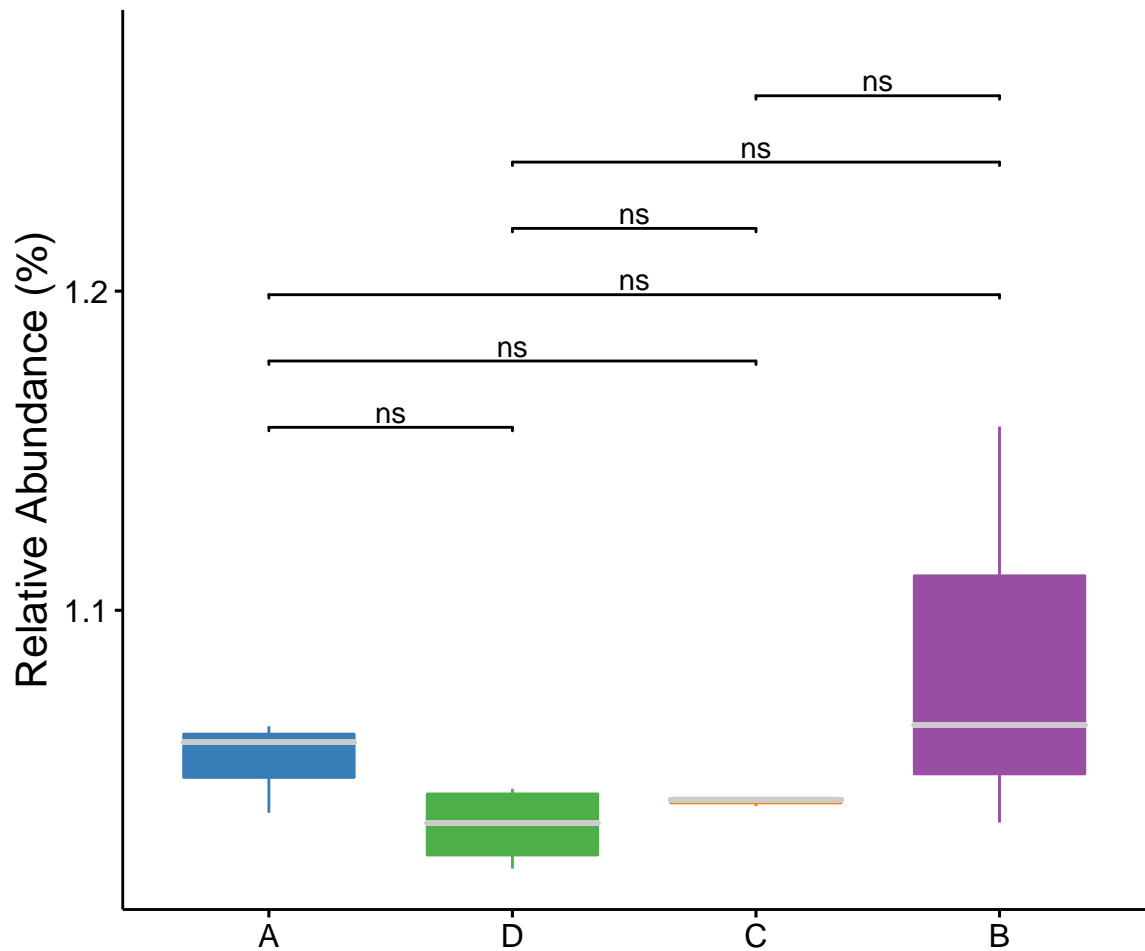

Supplement: Supplementary file 2 [file DataSheet1.zip › 16S rRNA/06.FunctionPrediction/Images/KEGG_level3_Glycine, serine and threonine metabolism.pdf]

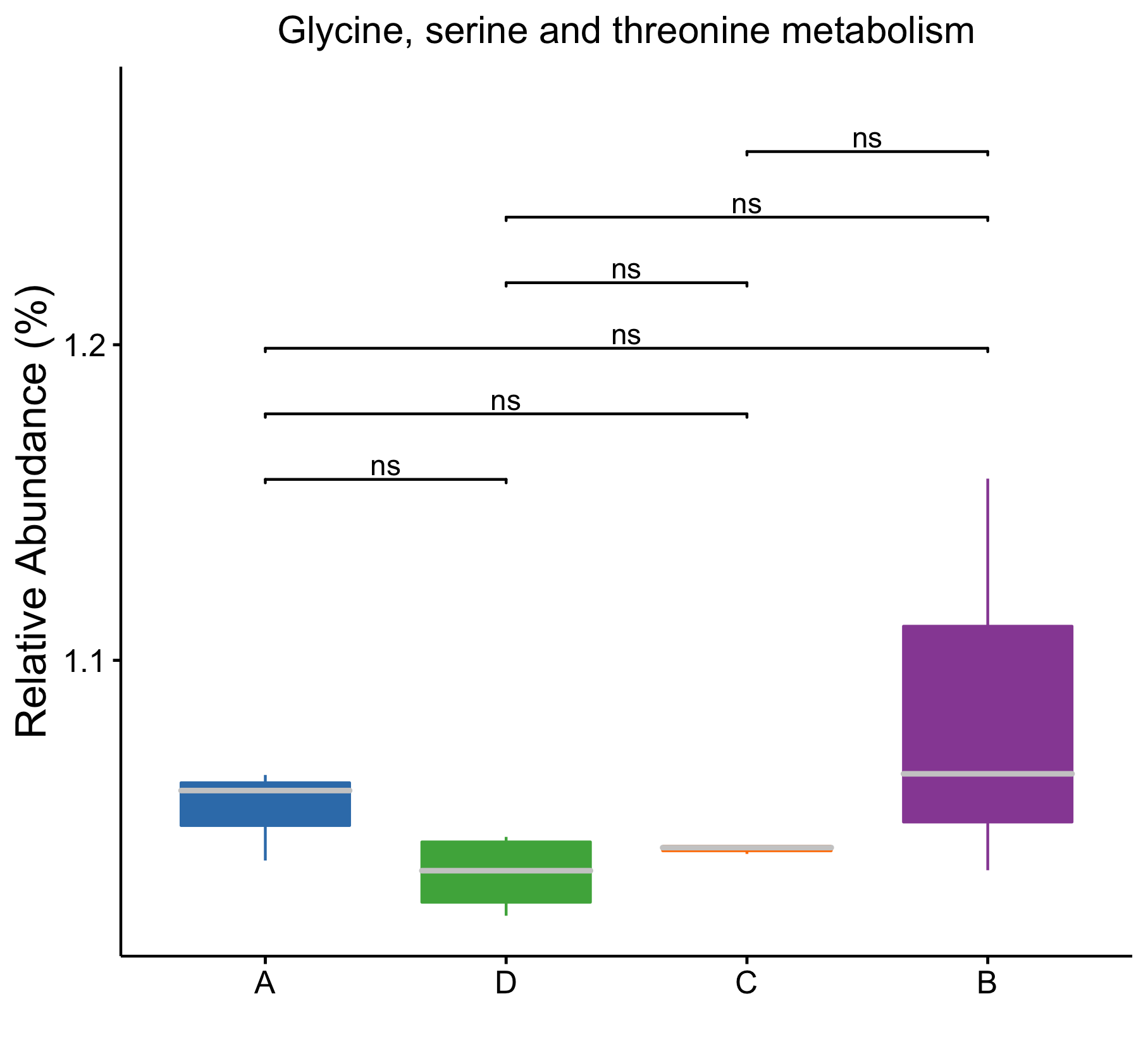

Supplement: Supplementary file 2 [file DataSheet1.zip › 16S rRNA/06.FunctionPrediction/Images/KEGG_level3_Glycine, serine and threonine metabolism.png]

# Histidine metabolism

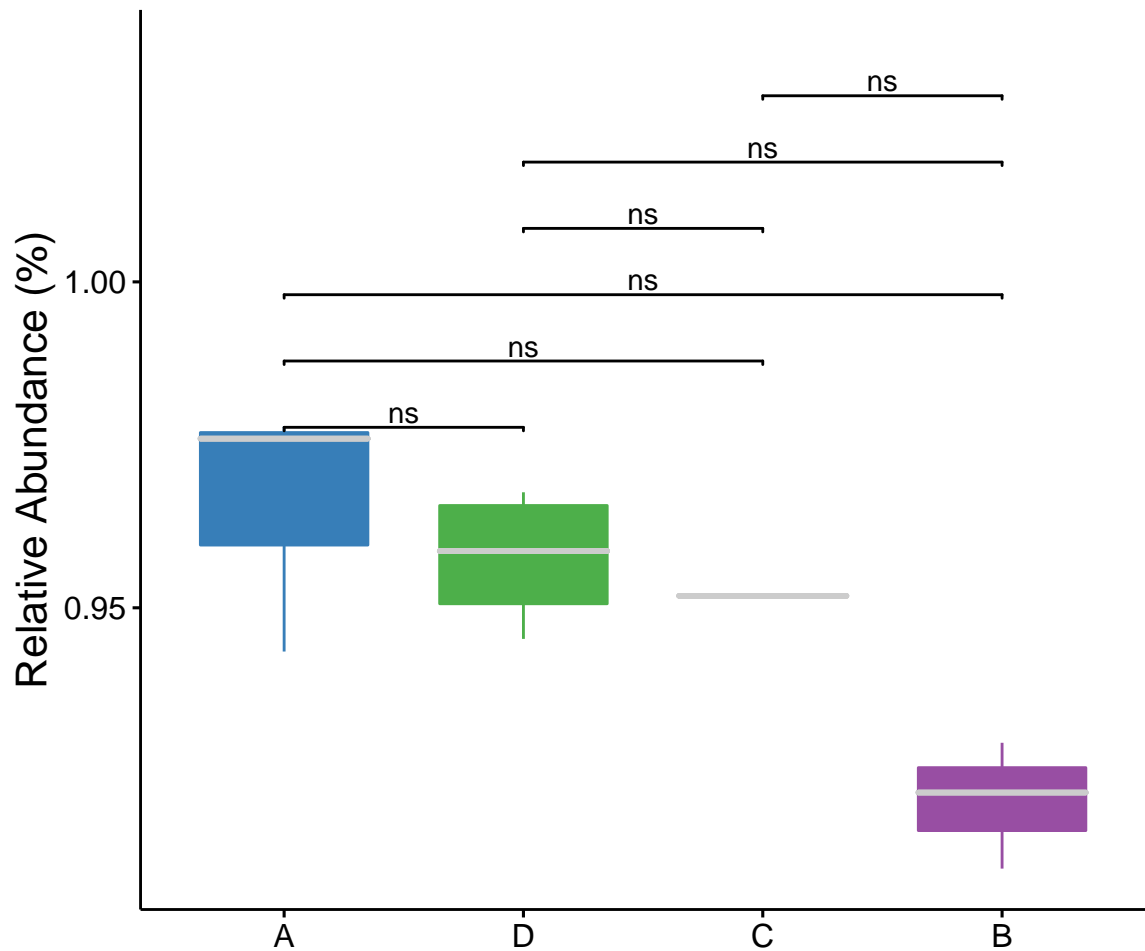

Supplement: Supplementary file 2 [file DataSheet1.zip › 16S rRNA/06.FunctionPrediction/Images/KEGG_level3_Histidine metabolism.pdf]

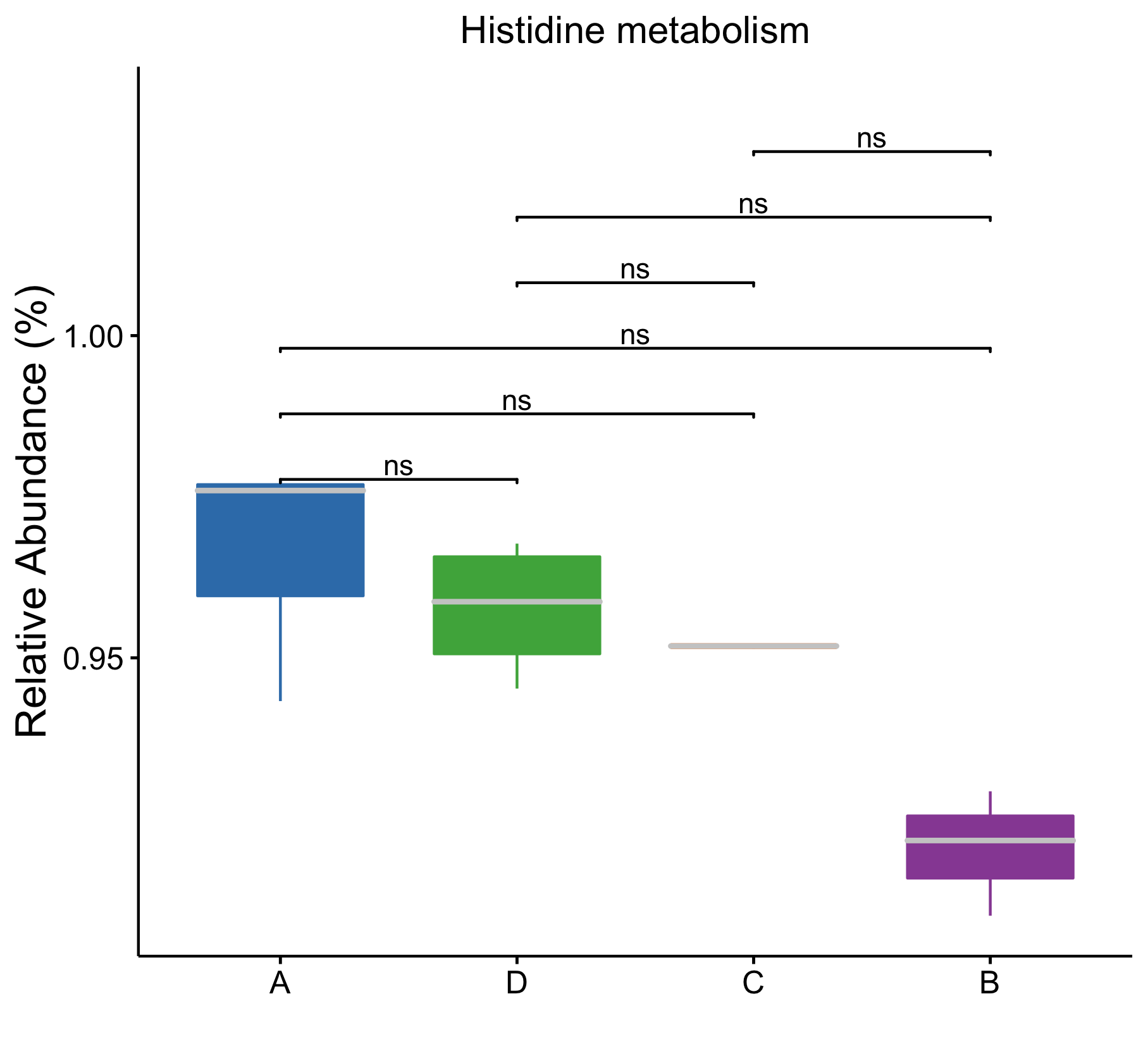

Supplement: Supplementary file 2 [file DataSheet1.zip › 16S rRNA/06.FunctionPrediction/Images/KEGG_level3_Histidine metabolism.png]

# Lysine biosynthesis

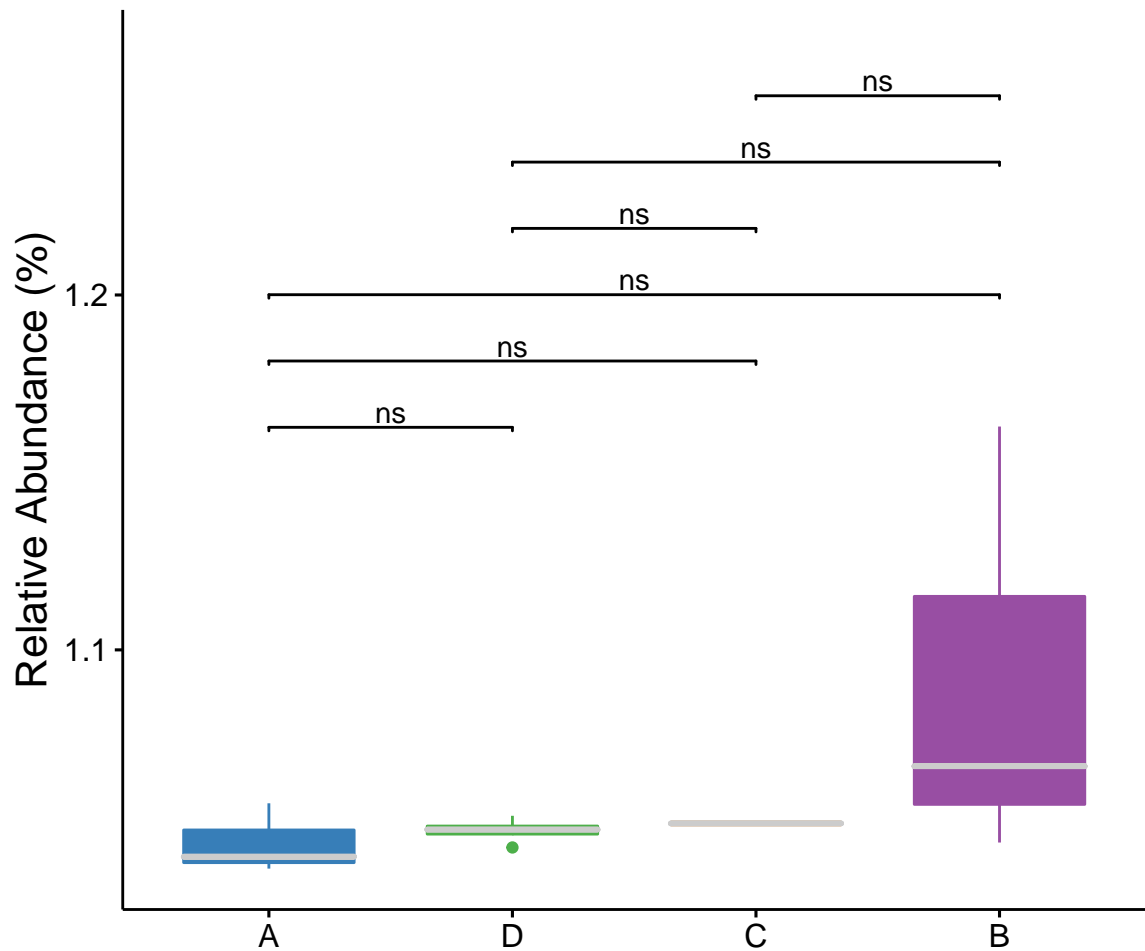

Supplement: Supplementary file 2 [file DataSheet1.zip › 16S rRNA/06.FunctionPrediction/Images/KEGG_level3_Lysine biosynthesis.pdf]

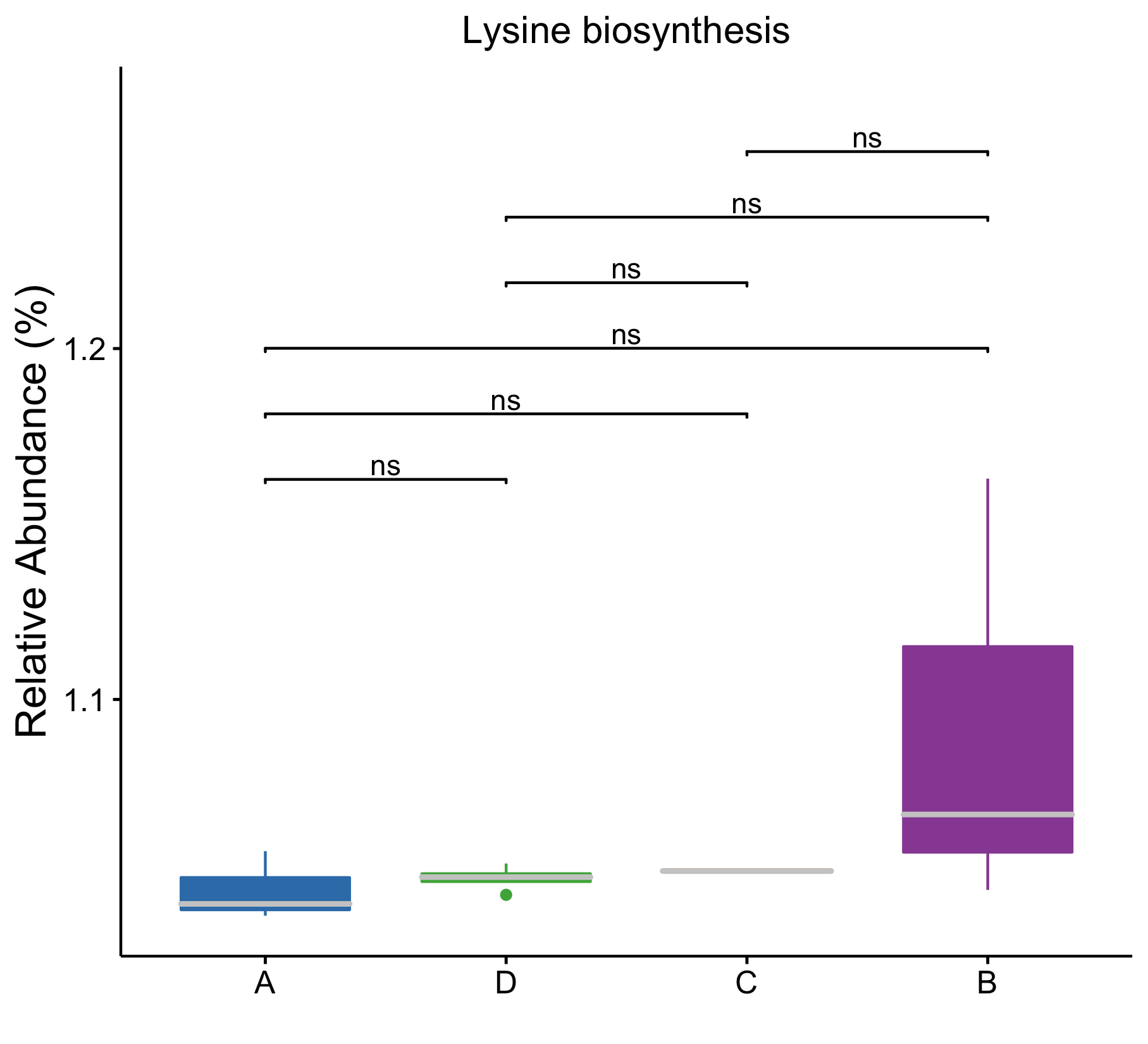

Supplement: Supplementary file 2 [file DataSheet1.zip › 16S rRNA/06.FunctionPrediction/Images/KEGG_level3_Lysine biosynthesis.png]

# Lysine degradation

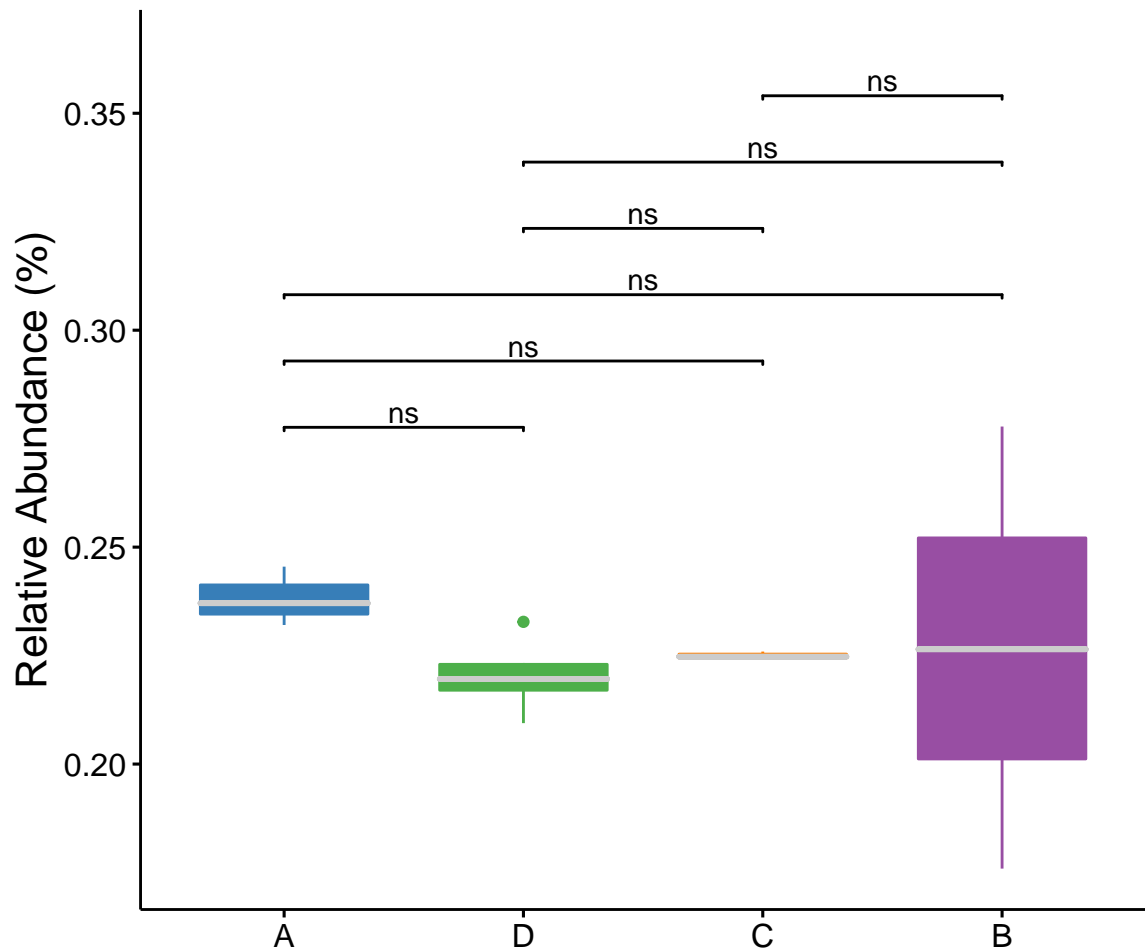

Supplement: Supplementary file 2 [file DataSheet1.zip › 16S rRNA/06.FunctionPrediction/Images/KEGG_level3_Lysine degradation.pdf]

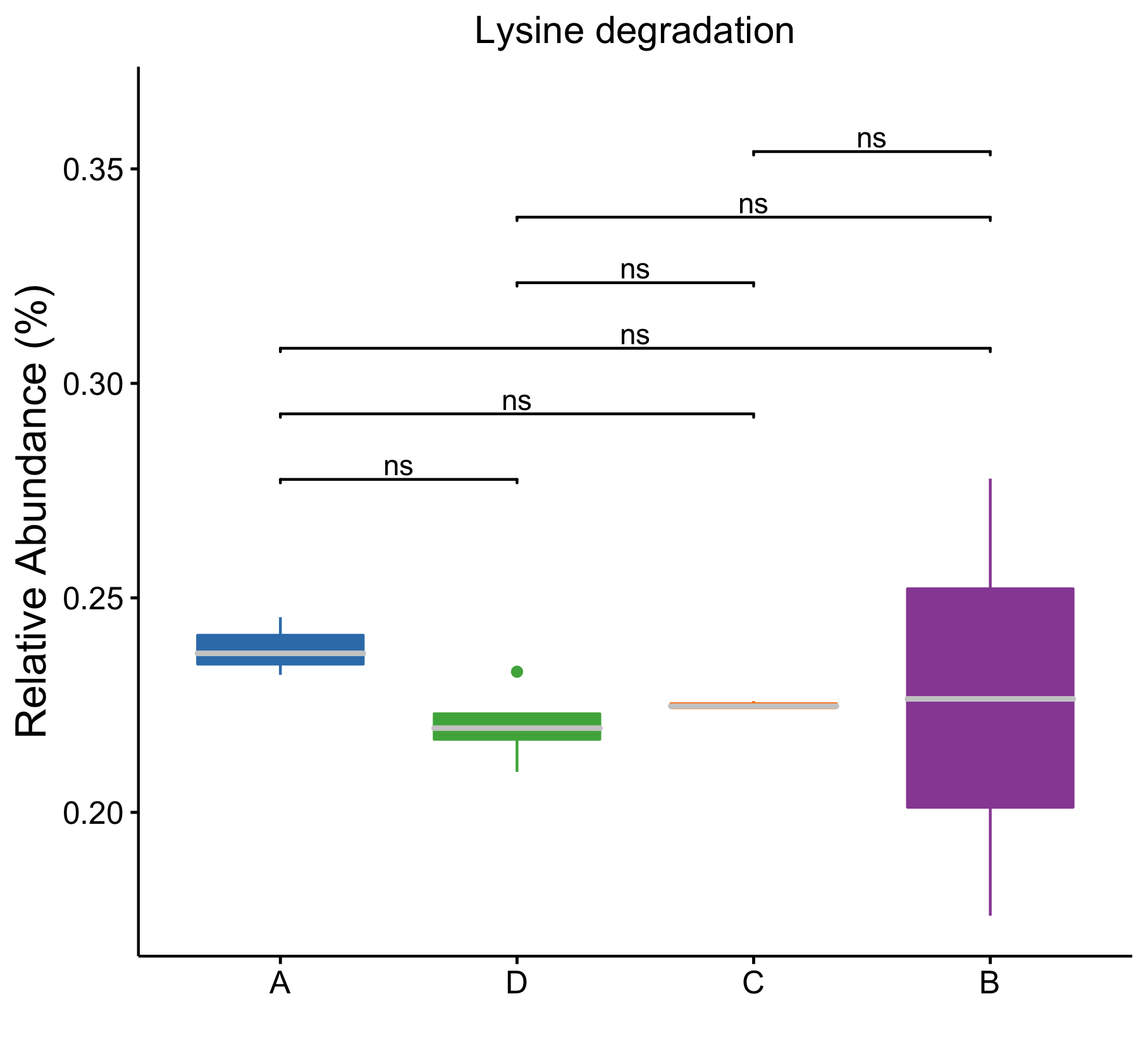

Supplement: Supplementary file 2 [file DataSheet1.zip › 16S rRNA/06.FunctionPrediction/Images/KEGG_level3_Lysine degradation.png]

# Phenylalanine metabolism

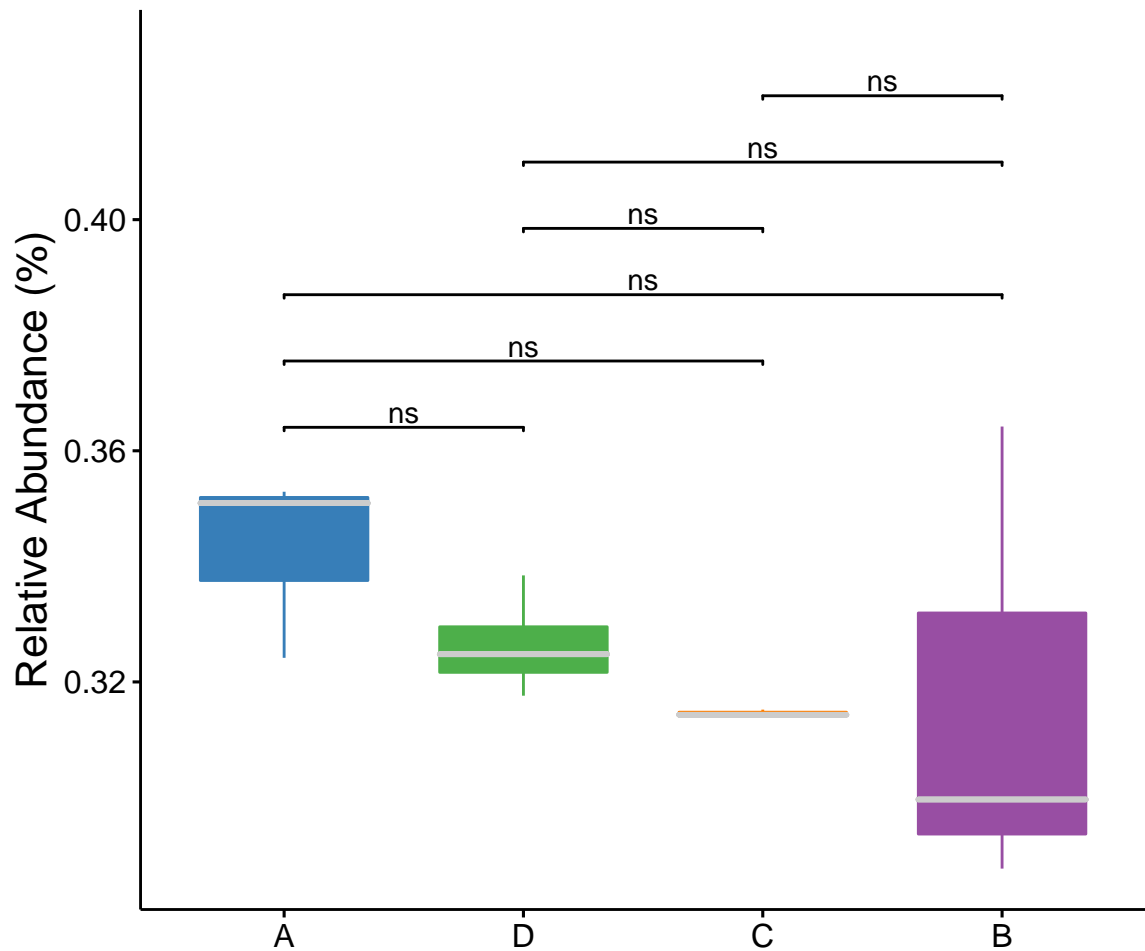

Supplement: Supplementary file 2 [file DataSheet1.zip › 16S rRNA/06.FunctionPrediction/Images/KEGG_level3_Phenylalanine metabolism.pdf]

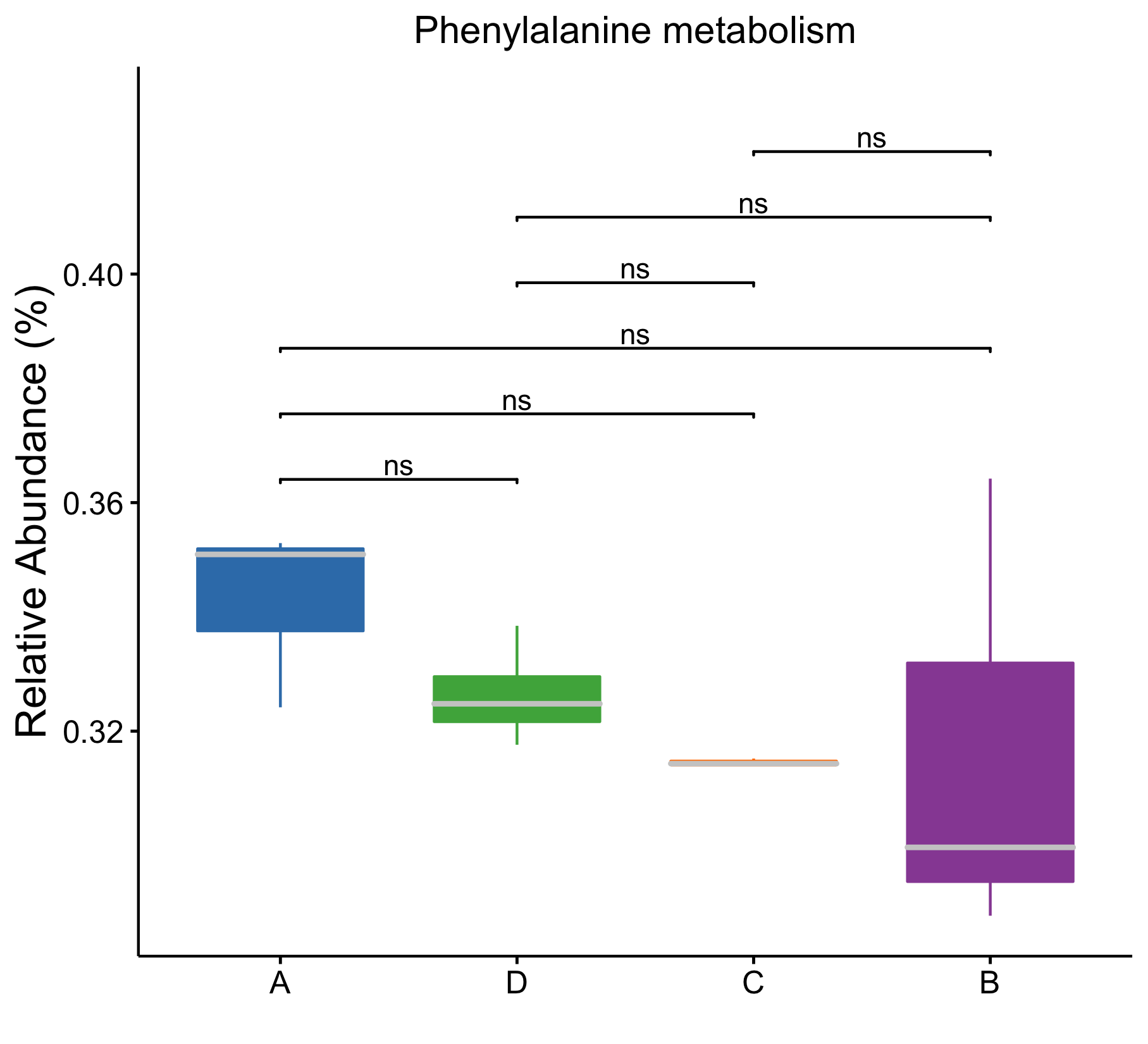

Supplement: Supplementary file 2 [file DataSheet1.zip › 16S rRNA/06.FunctionPrediction/Images/KEGG_level3_Phenylalanine metabolism.png]

# Phenylalanine, tyrosine and tryptophan biosynthesis

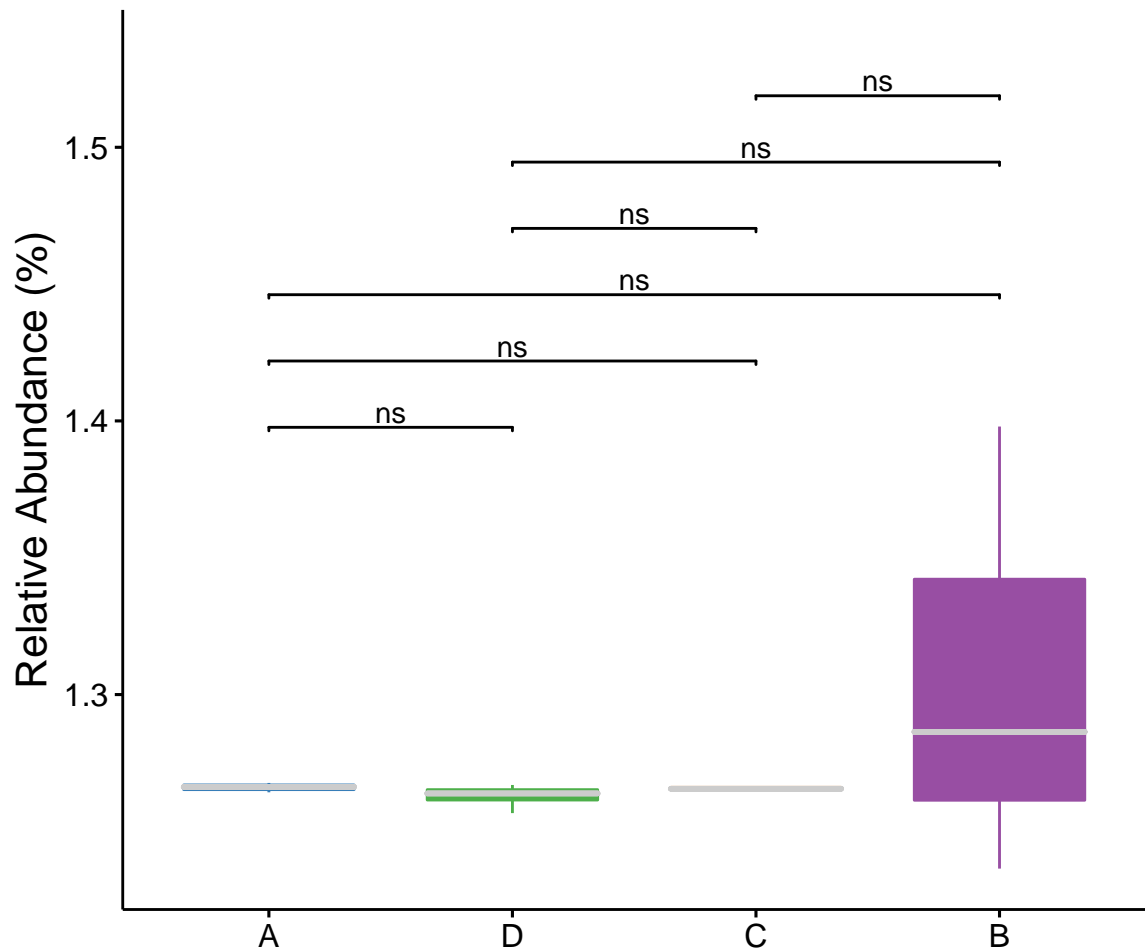

Supplement: Supplementary file 2 [file DataSheet1.zip › 16S rRNA/06.FunctionPrediction/Images/KEGG_level3_Phenylalanine, tyrosine and tryptophan biosynthesis.pdf]

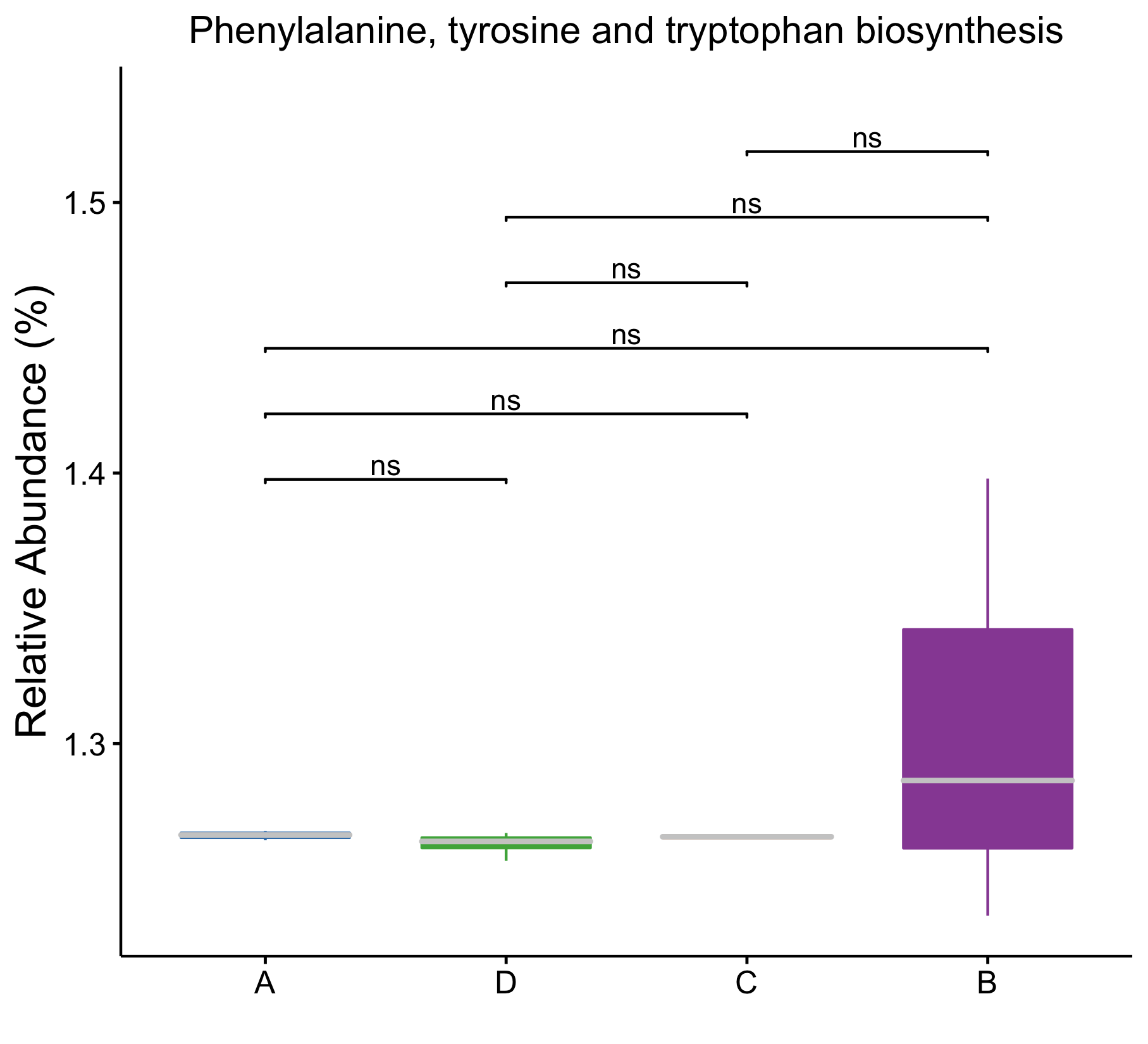

Supplement: Supplementary file 2 [file DataSheet1.zip › 16S rRNA/06.FunctionPrediction/Images/KEGG_level3_Phenylalanine, tyrosine and tryptophan biosynthesis.png]

# Tryptophan metabolism

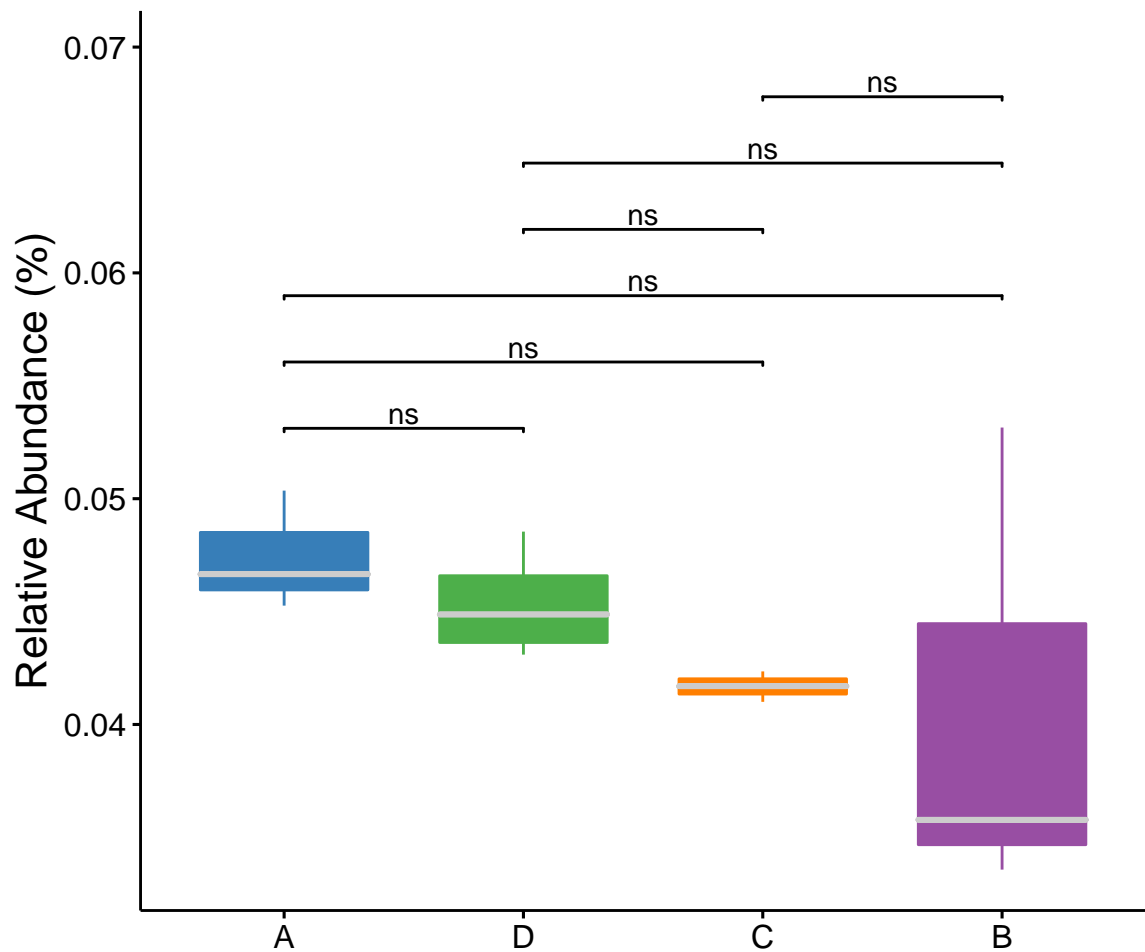

Supplement: Supplementary file 2 [file DataSheet1.zip › 16S rRNA/06.FunctionPrediction/Images/KEGG_level3_Tryptophan metabolism.pdf]

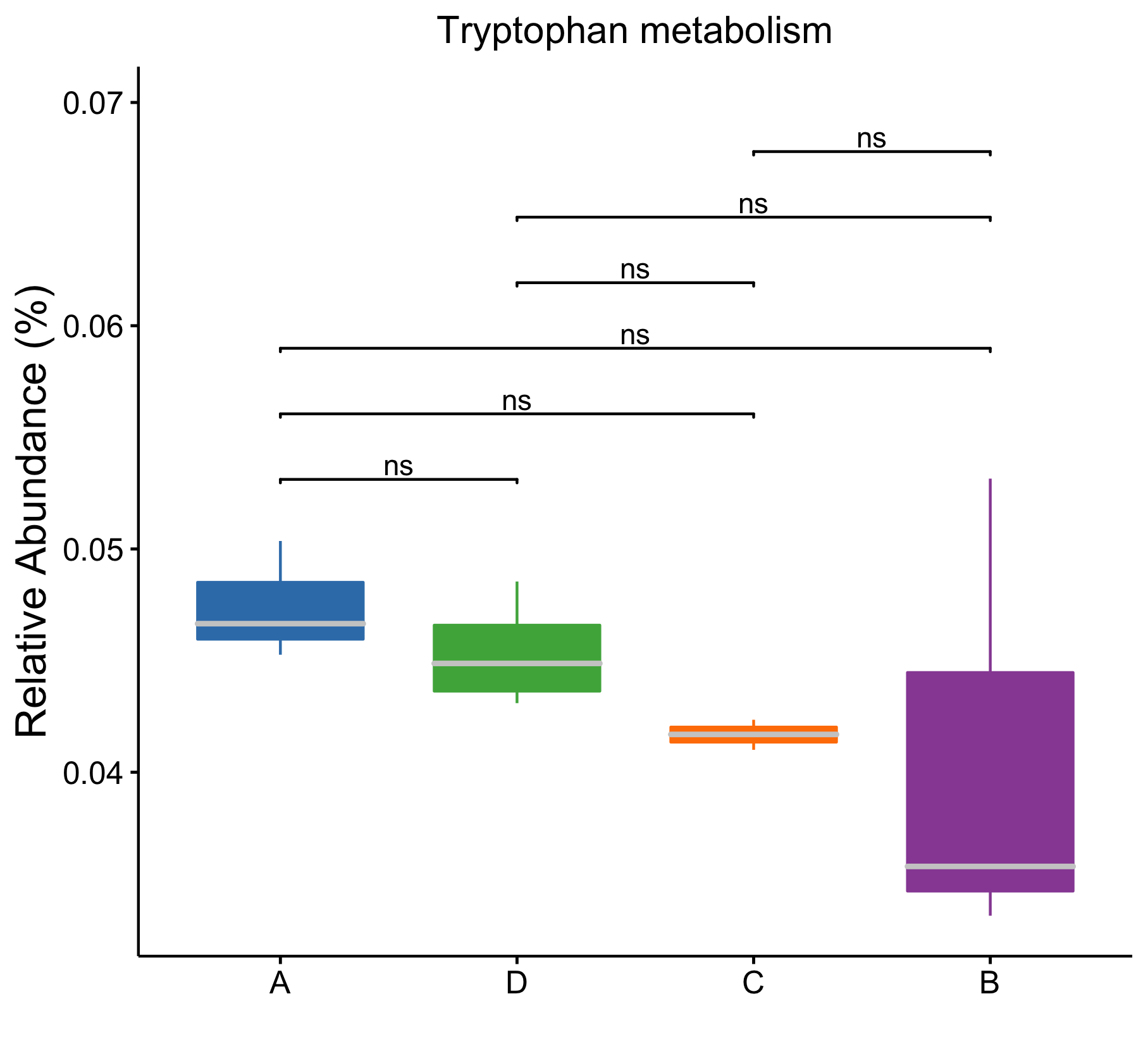

Supplement: Supplementary file 2 [file DataSheet1.zip › 16S rRNA/06.FunctionPrediction/Images/KEGG_level3_Tryptophan metabolism.png]

# Tyrosine metabolism

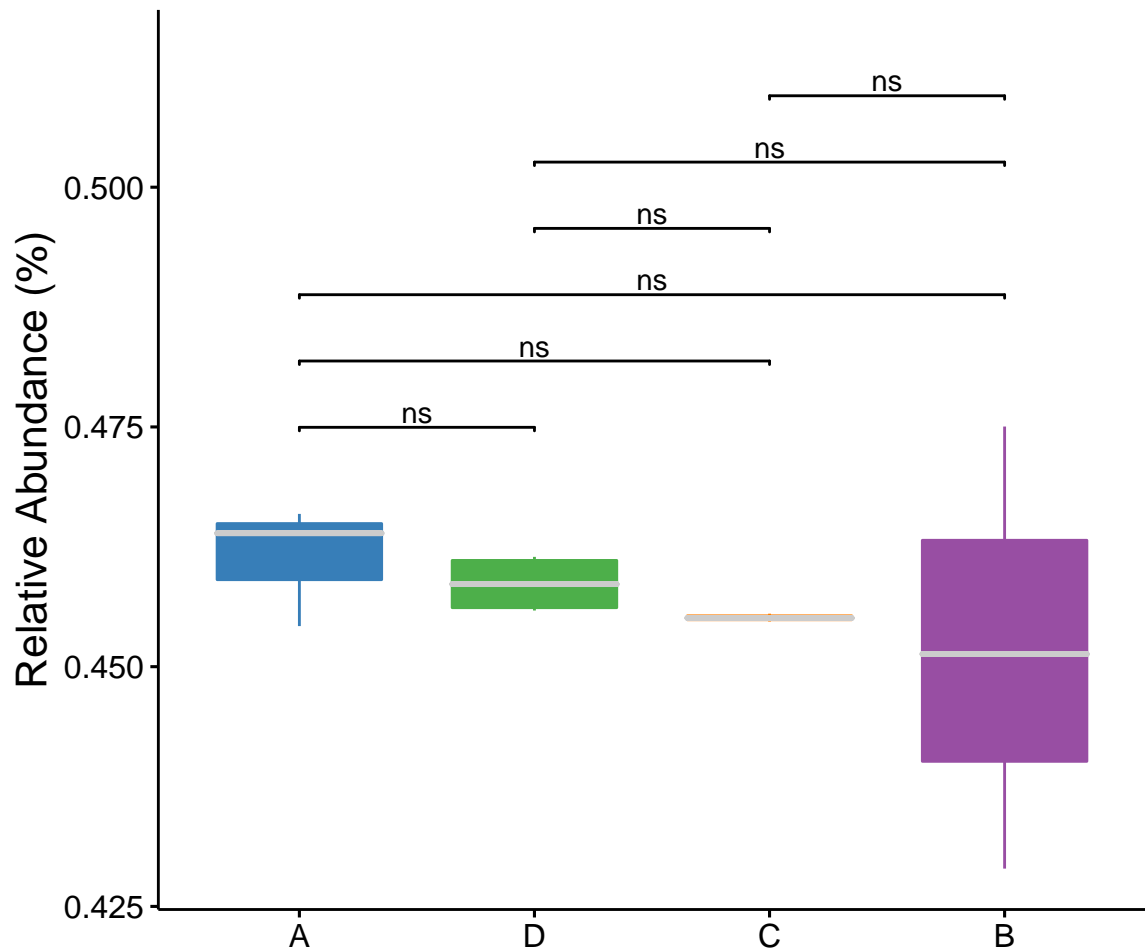

Supplement: Supplementary file 2 [file DataSheet1.zip › 16S rRNA/06.FunctionPrediction/Images/KEGG_level3_Tyrosine metabolism.pdf]

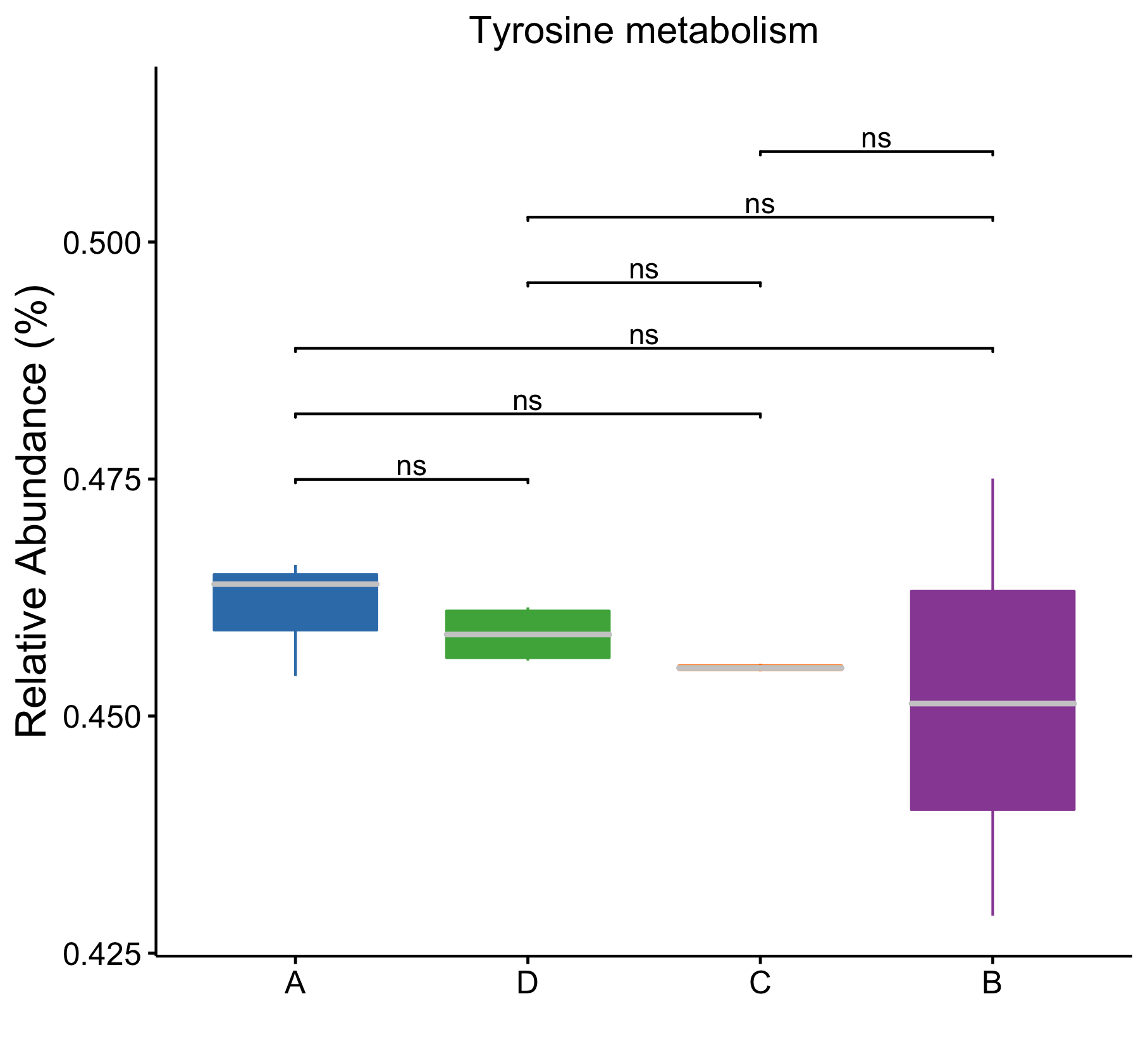

Supplement: Supplementary file 2 [file DataSheet1.zip › 16S rRNA/06.FunctionPrediction/Images/KEGG_level3_Tyrosine metabolism.png]

# Valine, leucine and isoleucine biosynthesis

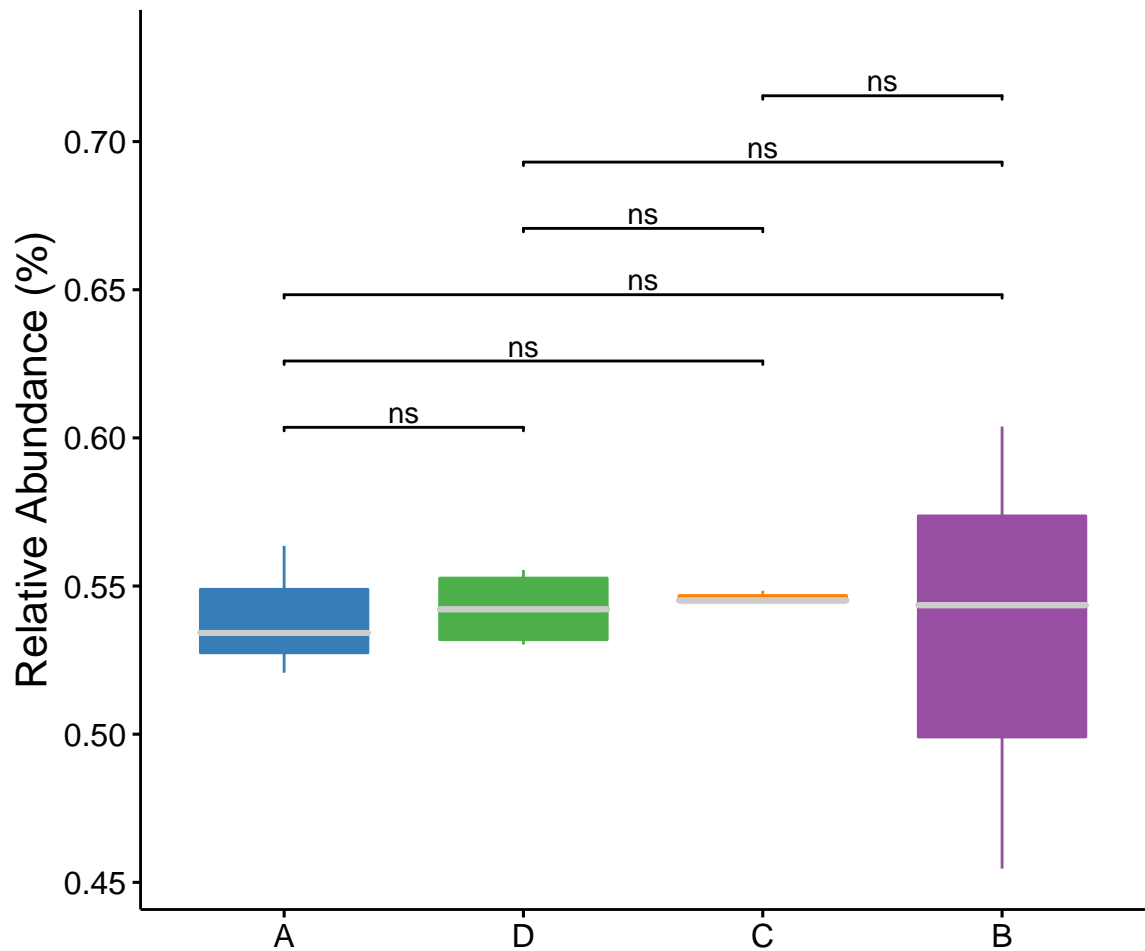

Supplement: Supplementary file 2 [file DataSheet1.zip › 16S rRNA/06.FunctionPrediction/Images/KEGG_level3_Valine, leucine and isoleucine biosynthesis.pdf]

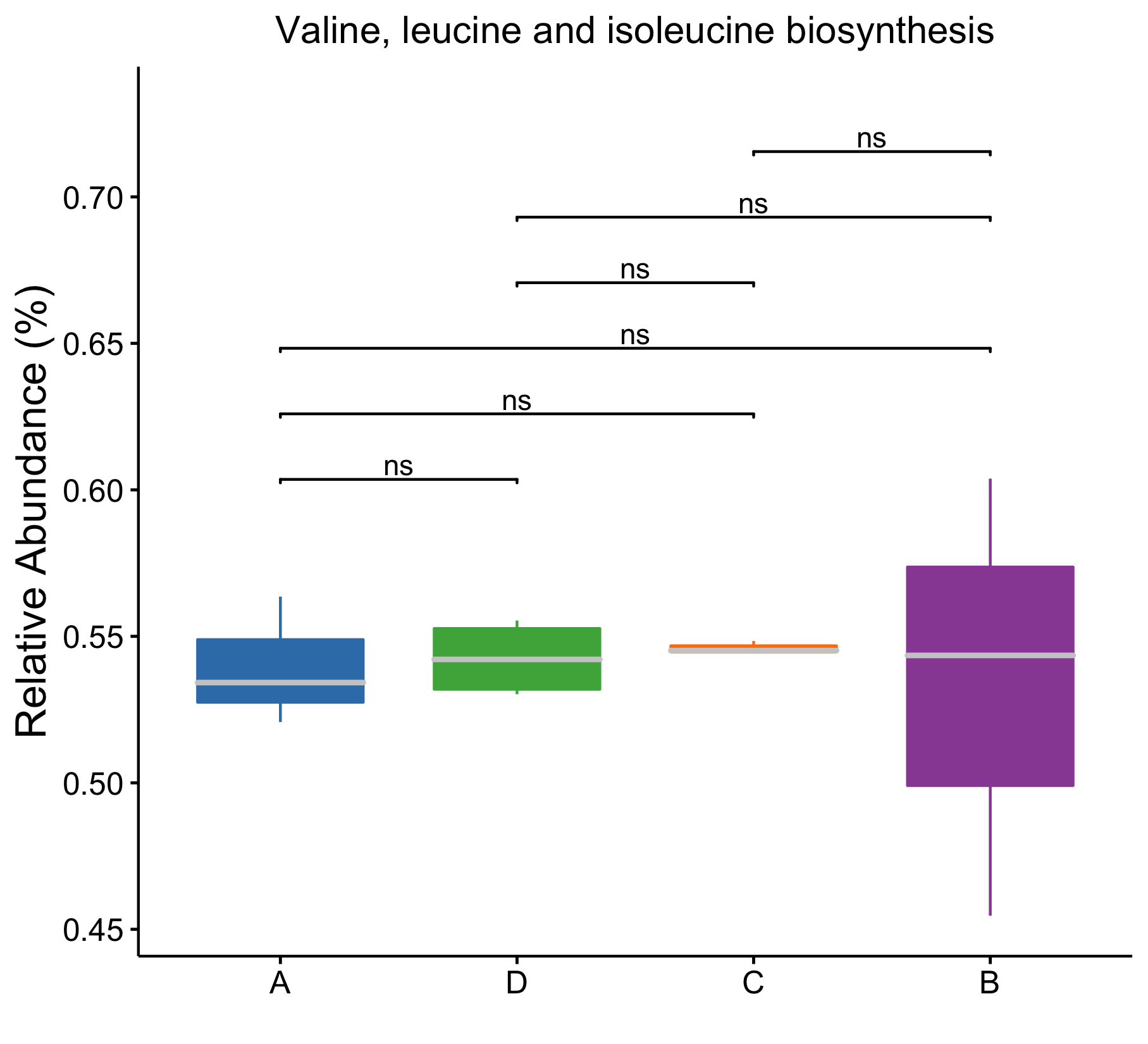

Supplement: Supplementary file 2 [file DataSheet1.zip › 16S rRNA/06.FunctionPrediction/Images/KEGG_level3_Valine, leucine and isoleucine biosynthesis.png]

# Valine, leucine and isoleucine degradation

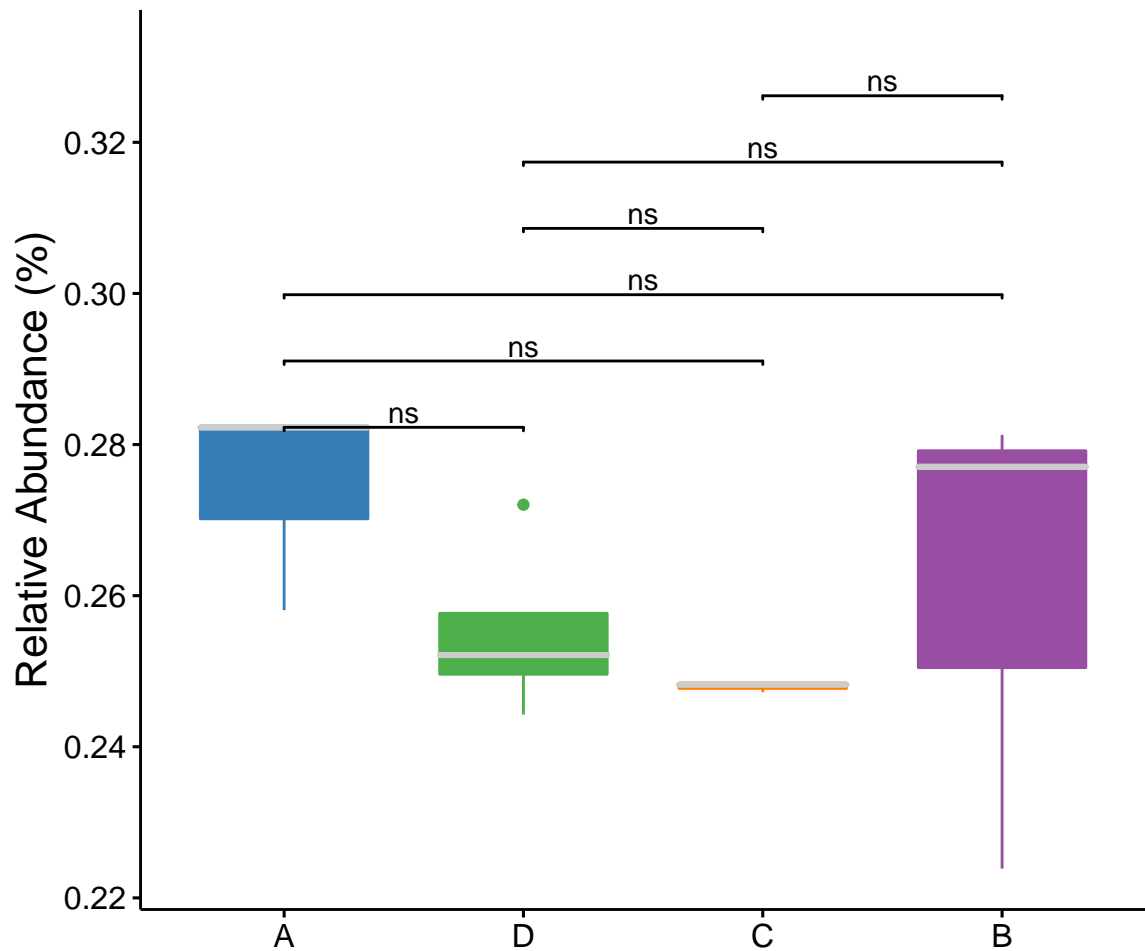

Supplement: Supplementary file 2 [file DataSheet1.zip › 16S rRNA/06.FunctionPrediction/Images/KEGG_level3_Valine, leucine and isoleucine degradation.pdf]

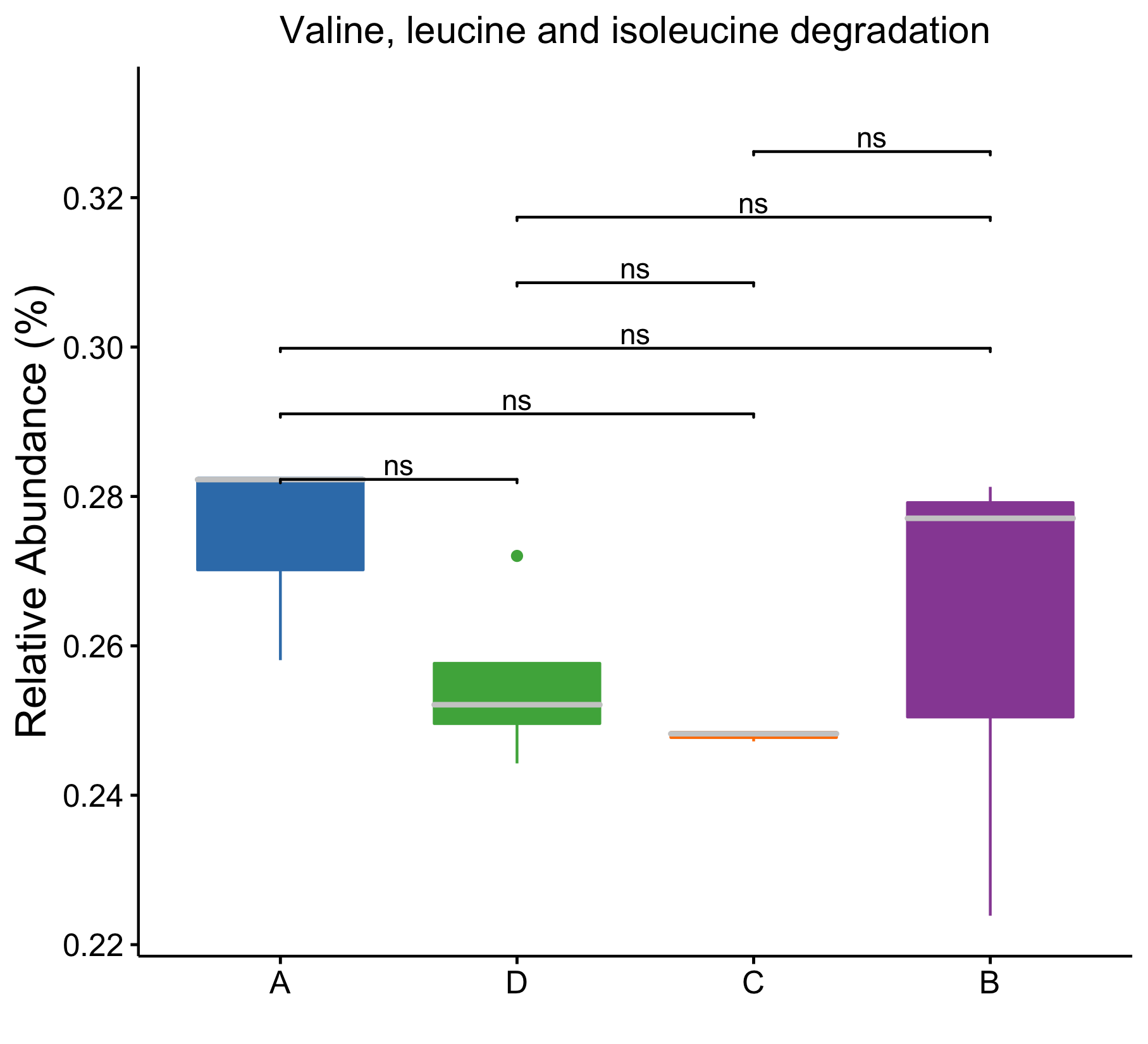

Supplement: Supplementary file 2 [file DataSheet1.zip › 16S rRNA/06.FunctionPrediction/Images/KEGG_level3_Valine, leucine and isoleucine degradation.png]

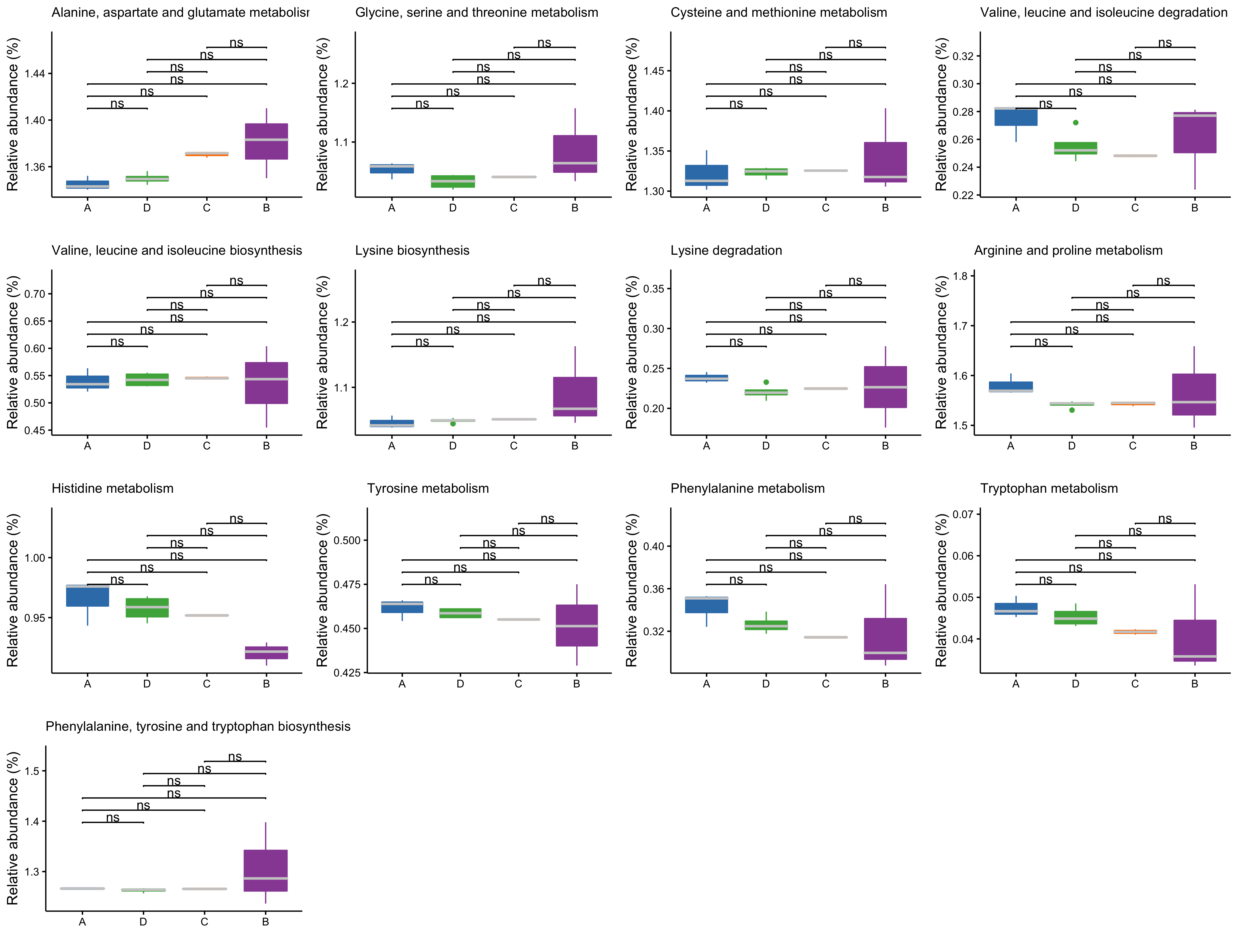

Supplement: Supplementary file 2 [file DataSheet1.zip › 16S rRNA/06.FunctionPrediction/Images/KEGG_total_wilcox-test.png]

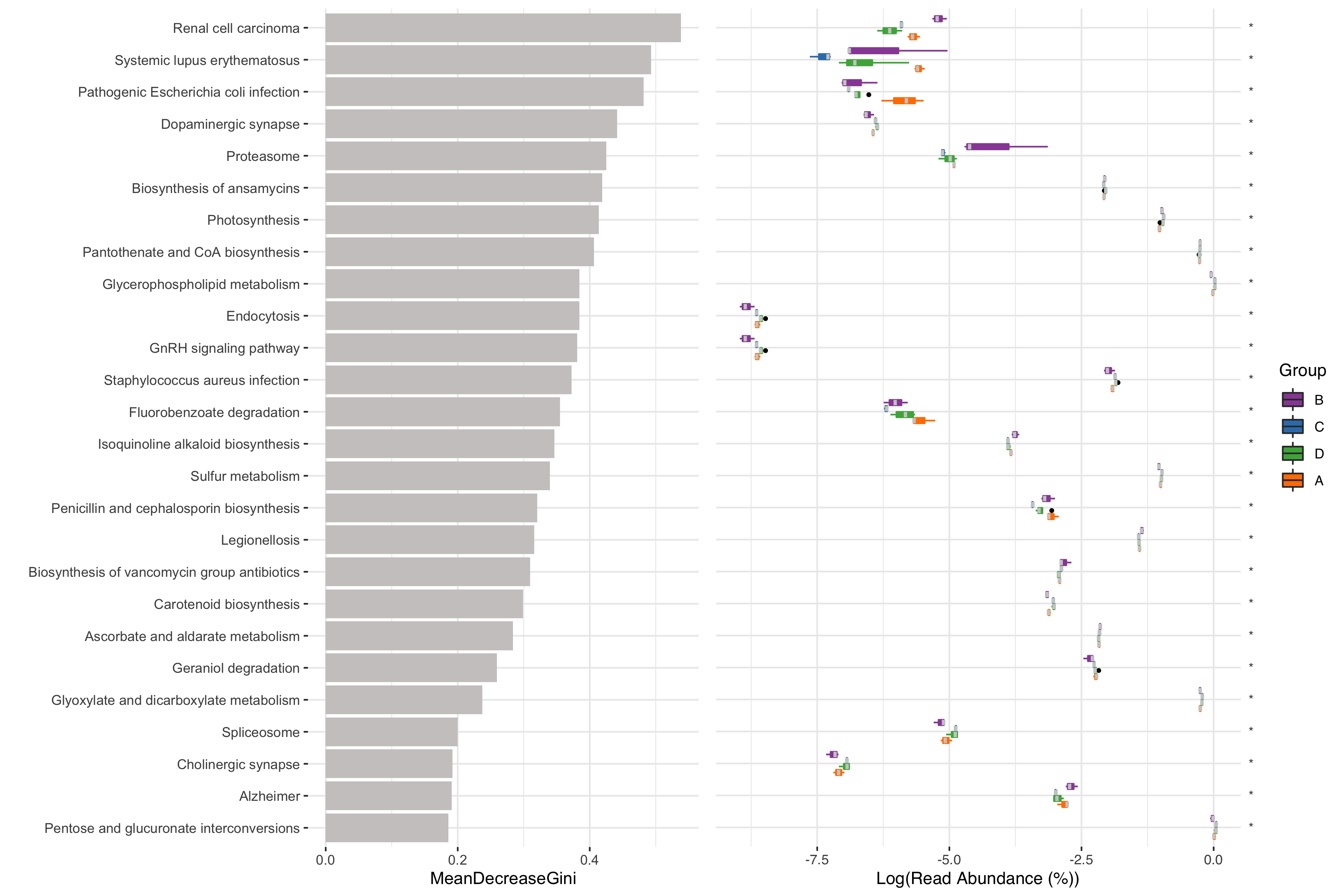

Supplement: Supplementary file 2 [file DataSheet1.zip › 16S rRNA/06.FunctionPrediction/Images/RandomForest_Gini_function.png]

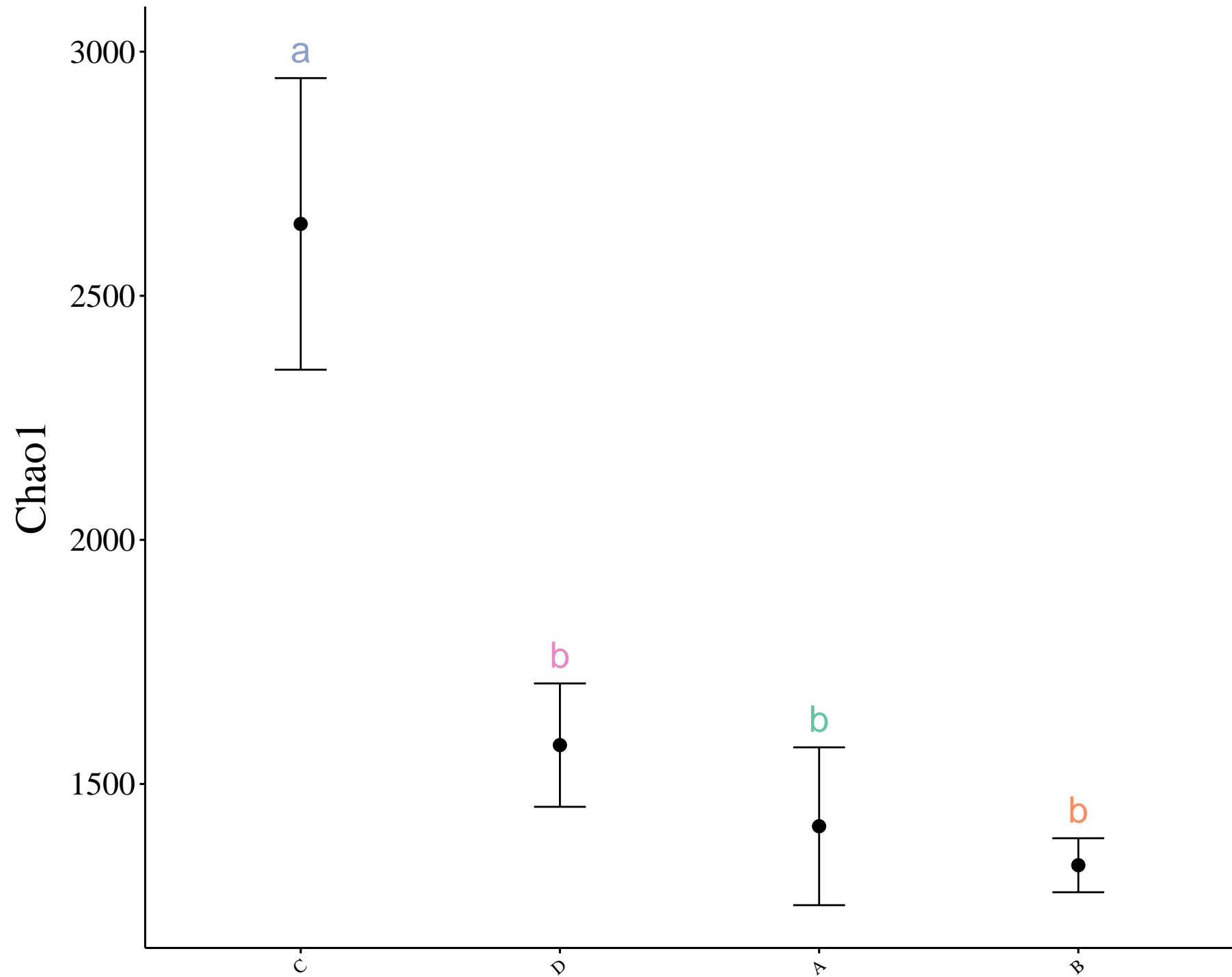

Supplement: Supplementary file 2 [file DataSheet1.zip › 16S rRNA/Images/AlphaDiversity_Chao1_duncun.pdf]

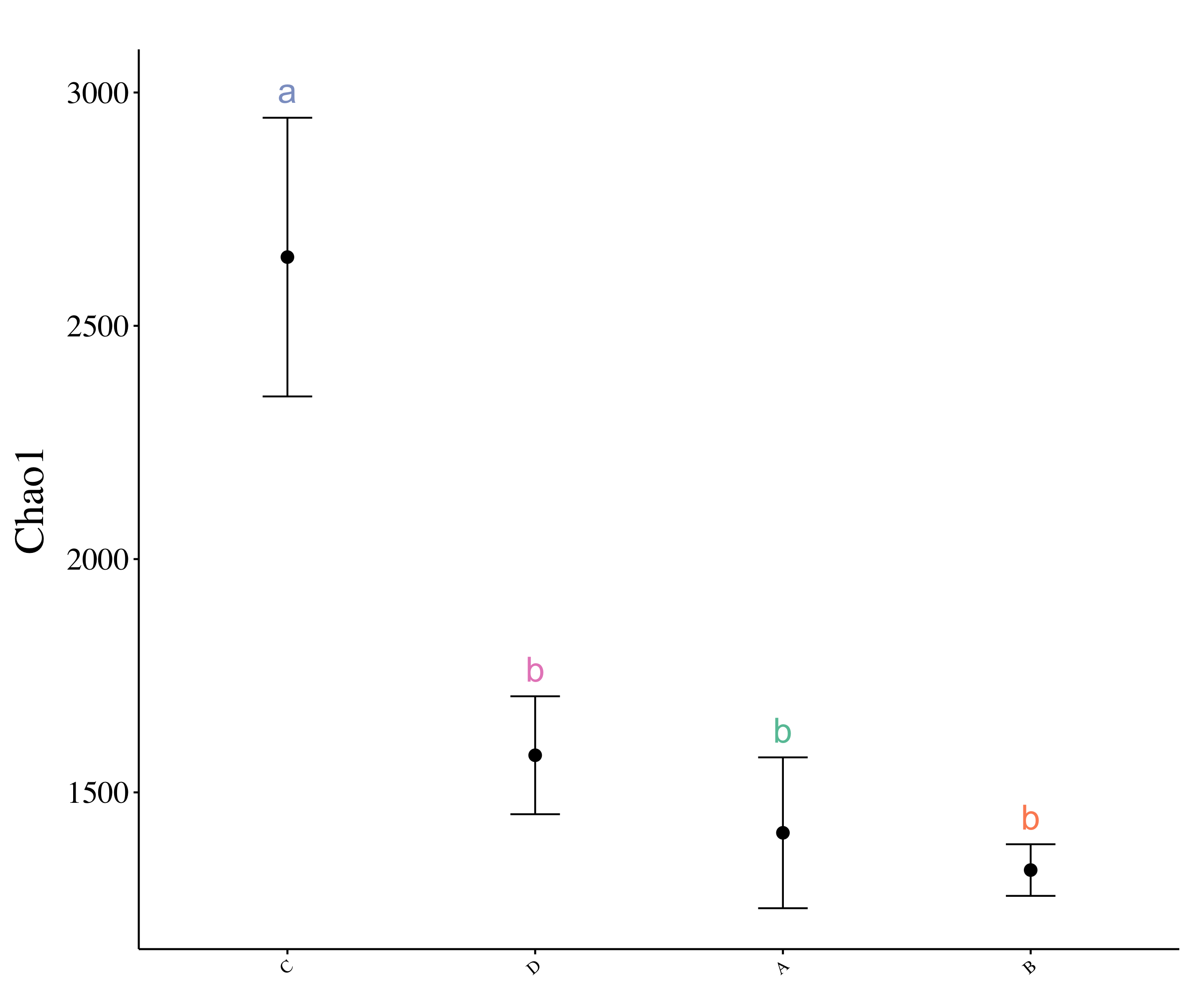

Supplement: Supplementary file 2 [file DataSheet1.zip › 16S rRNA/Images/AlphaDiversity_Chao1_duncun.png]

PD

130  
120  
110  
100  
90  
80

a

b

b

b

C

A

D

B

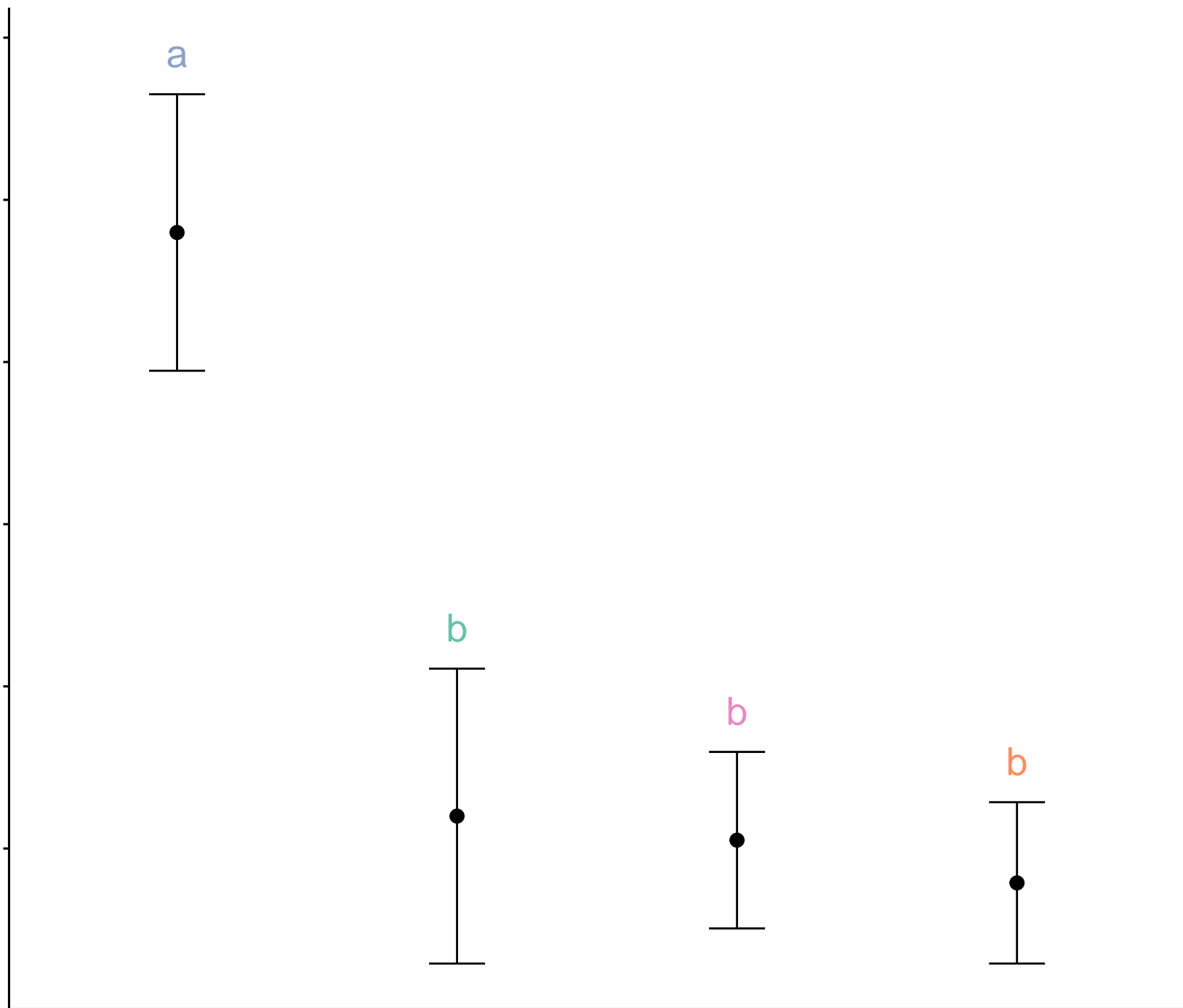

Supplement: Supplementary file 2 [file DataSheet1.zip › 16S rRNA/Images/AlphaDiversity_PD_duncun.pdf]

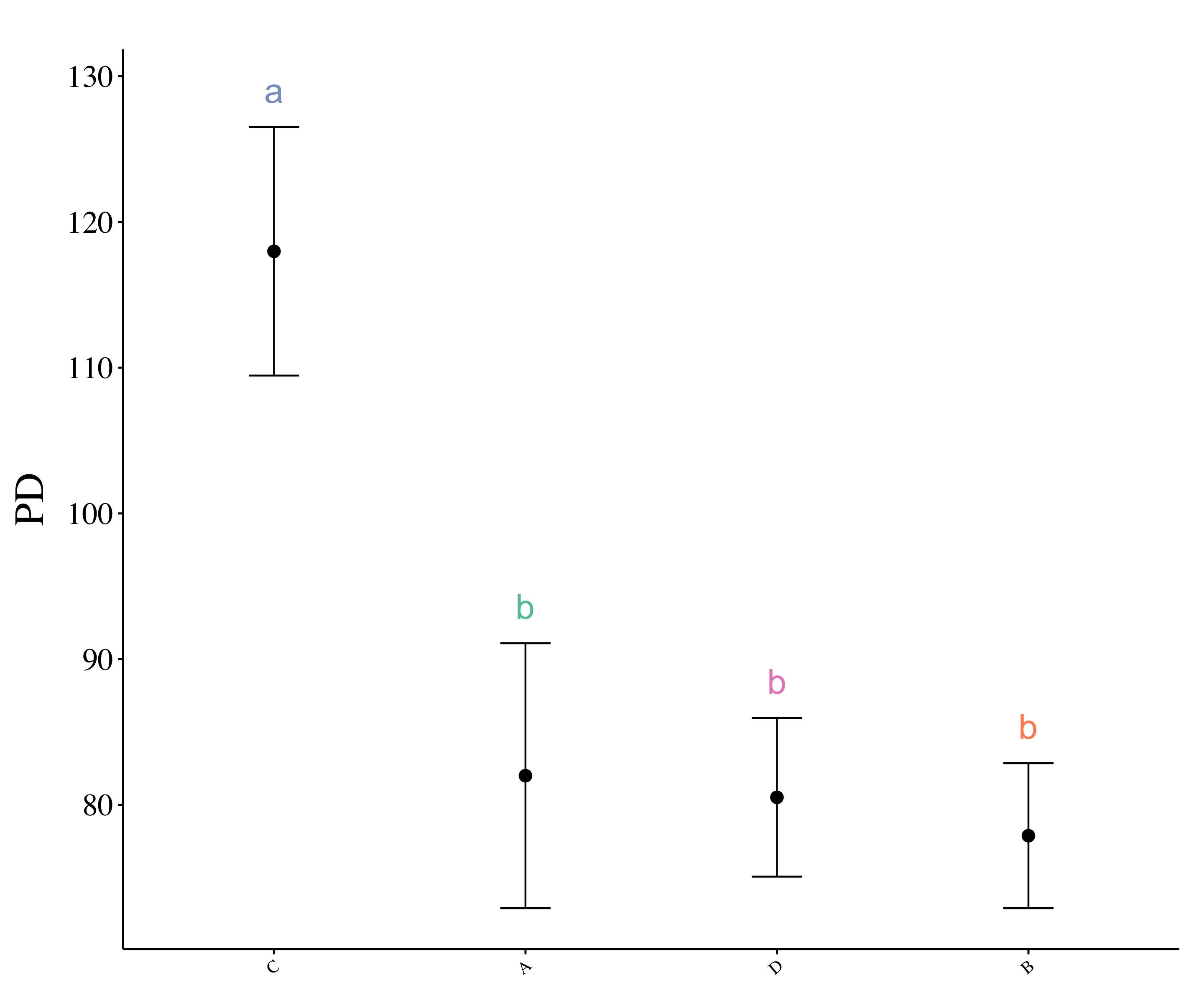

Supplement: Supplementary file 2 [file DataSheet1.zip › 16S rRNA/Images/AlphaDiversity_PD_duncun.png]

Shannon

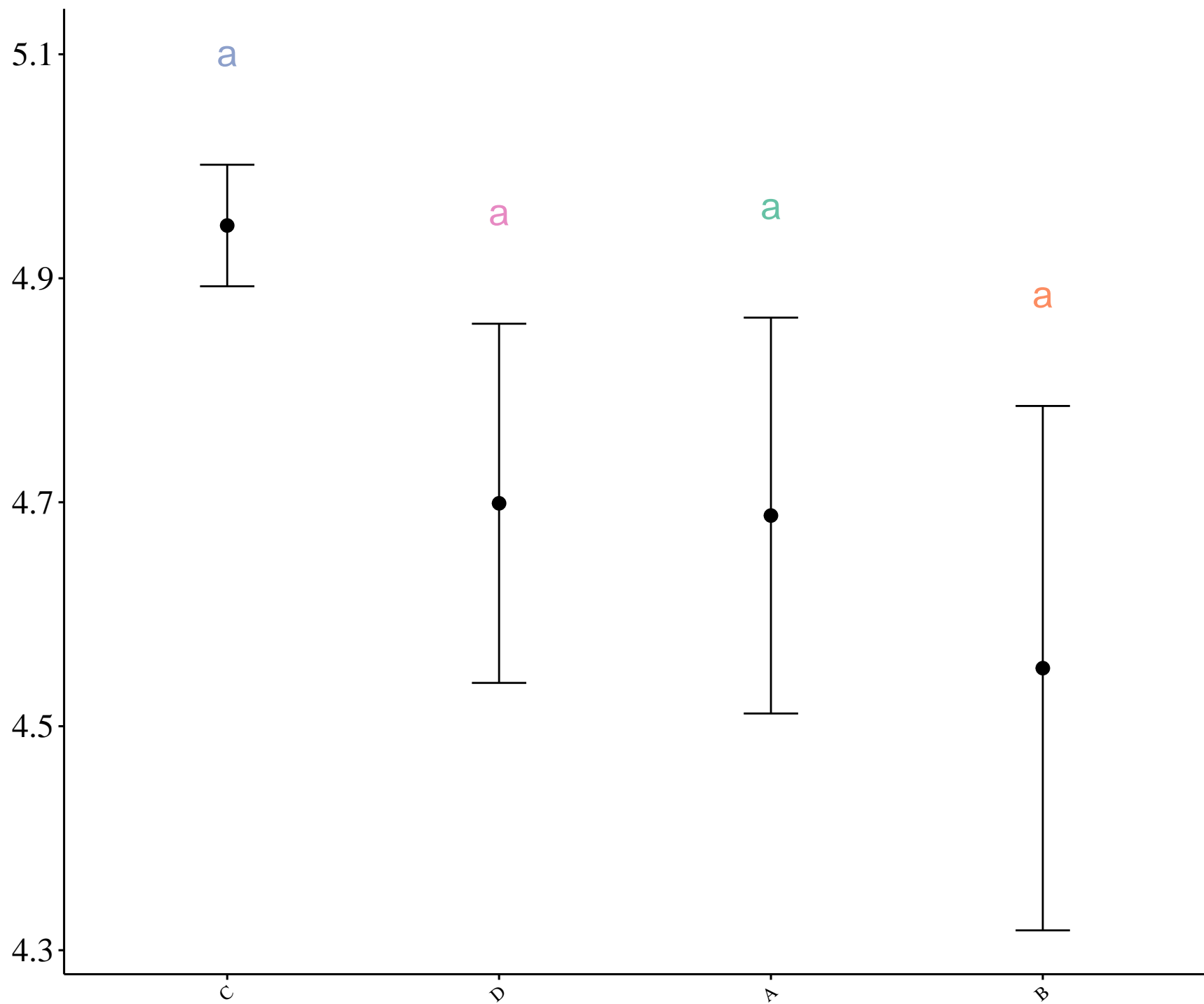

Supplement: Supplementary file 2 [file DataSheet1.zip › 16S rRNA/Images/AlphaDiversity_Shannon_duncun.pdf]

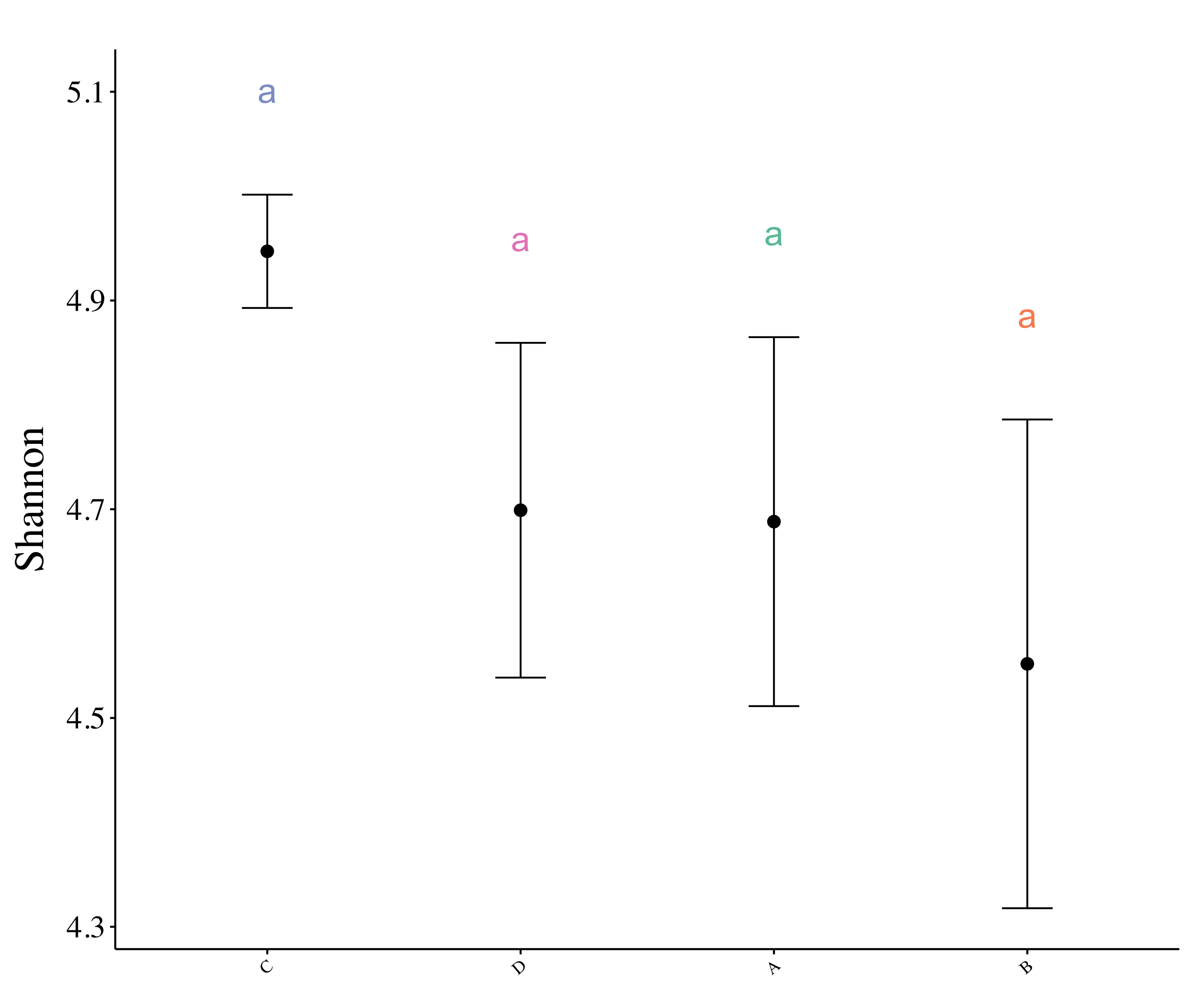

Supplement: Supplementary file 2 [file DataSheet1.zip › 16S rRNA/Images/AlphaDiversity_Shannon_duncun.png]

Simpson

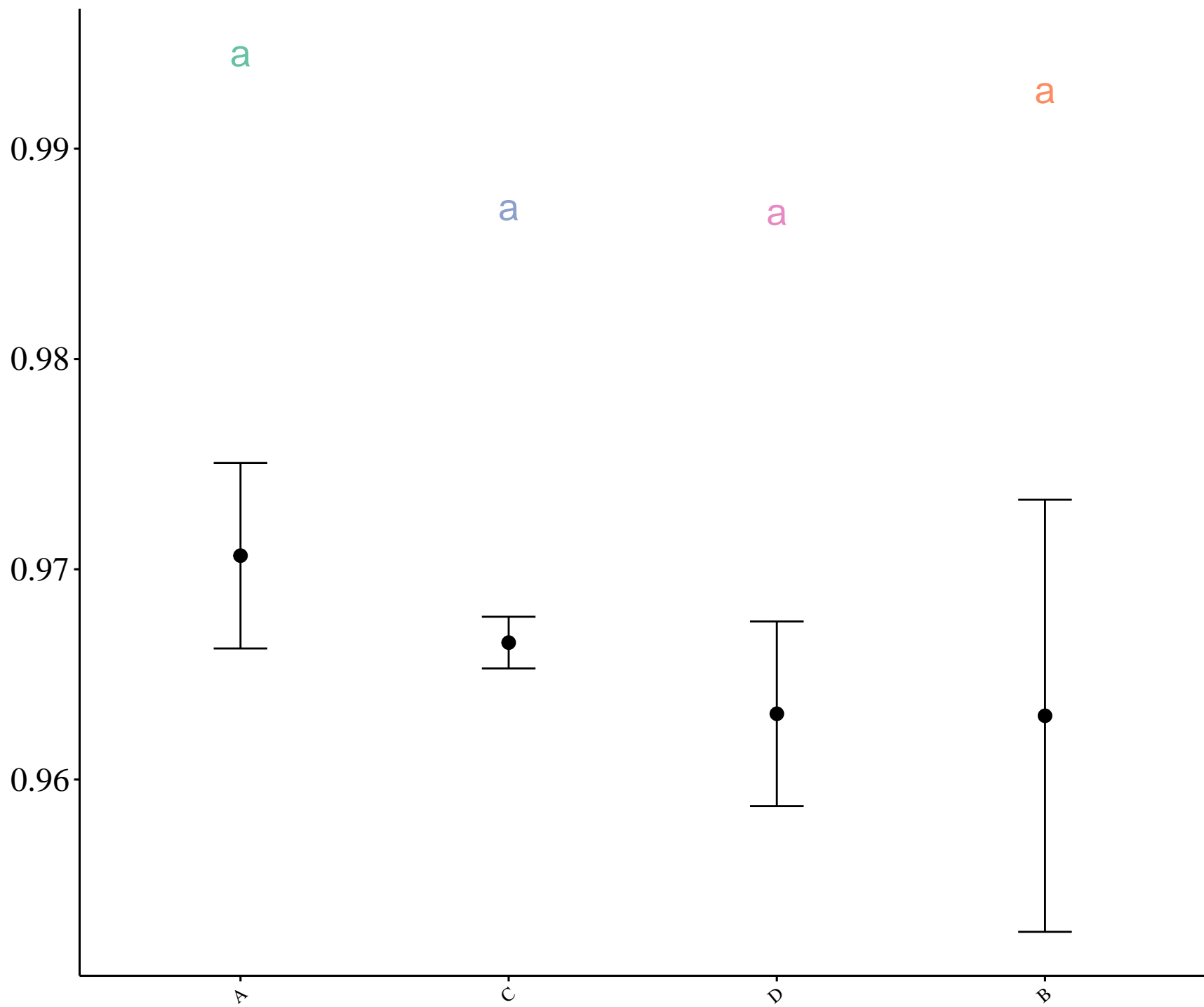

Supplement: Supplementary file 2 [file DataSheet1.zip › 16S rRNA/Images/AlphaDiversity_Simpson_duncun.pdf]

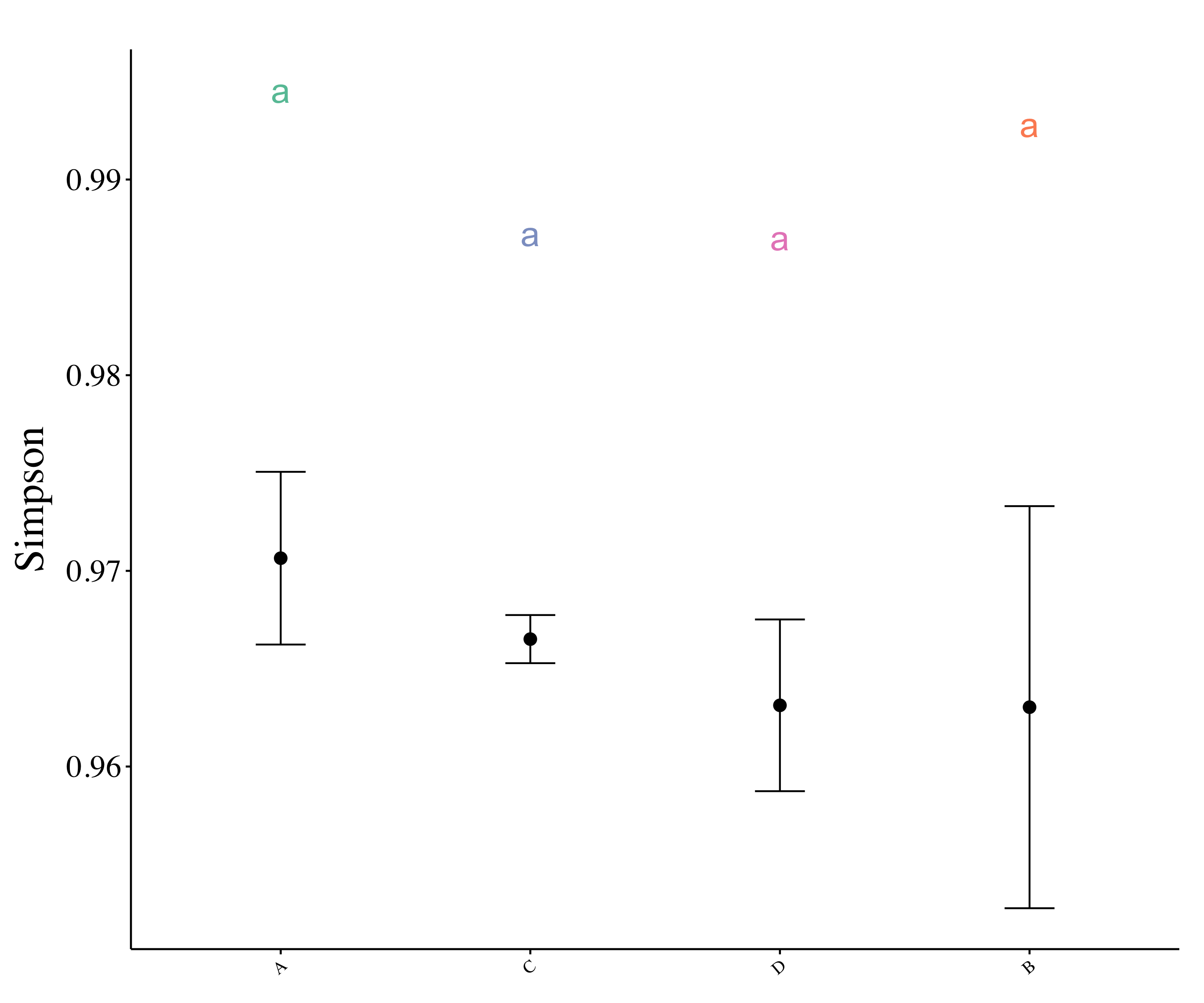

Supplement: Supplementary file 2 [file DataSheet1.zip › 16S rRNA/Images/AlphaDiversity_Simpson_duncun.png]

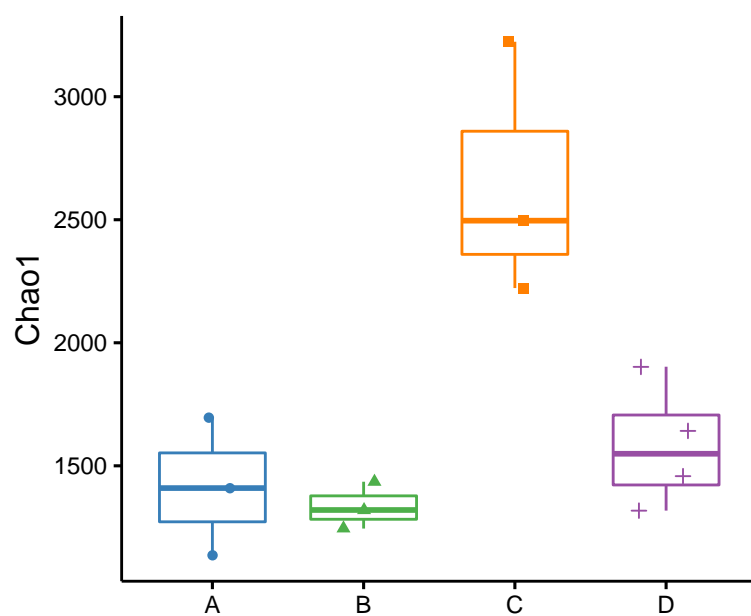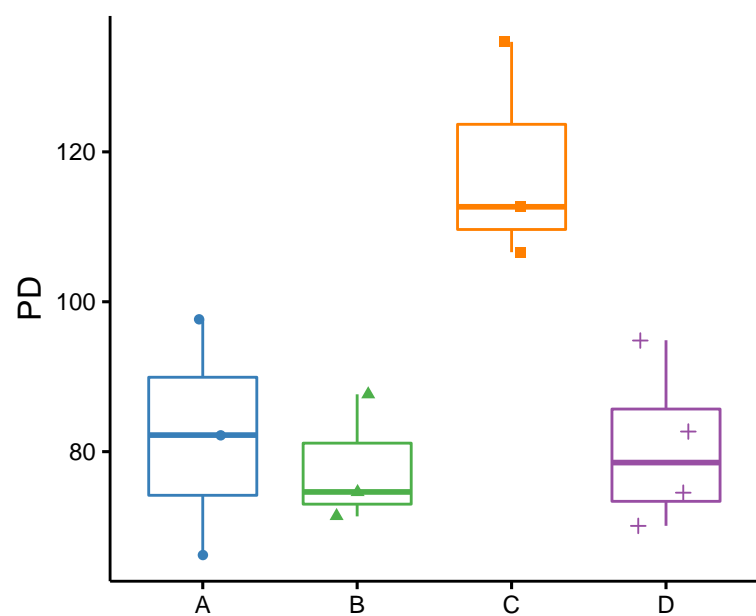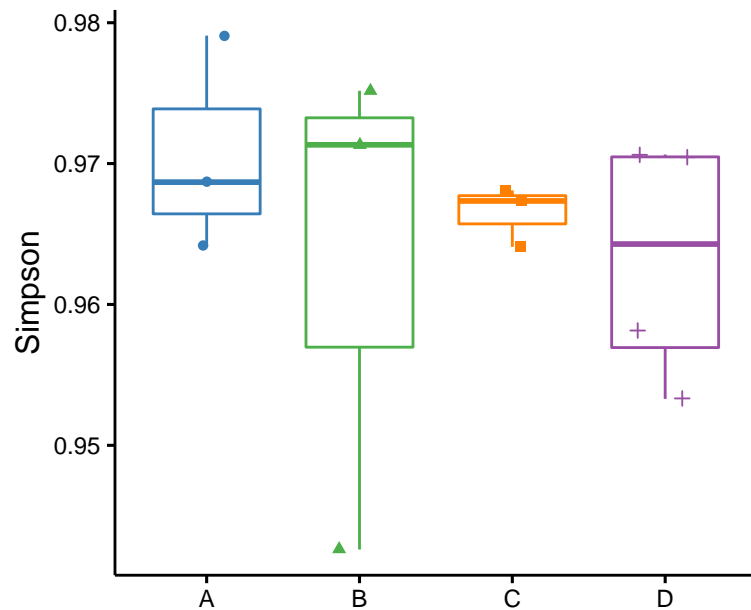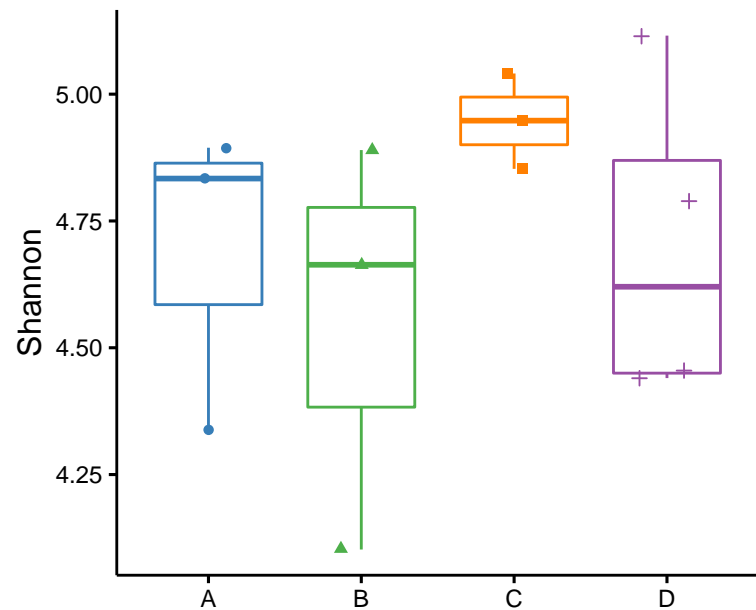

Supplement: Supplementary file 2 [file DataSheet1.zip › 16S rRNA/Images/Alpha_diversity_boxplot.pdf]

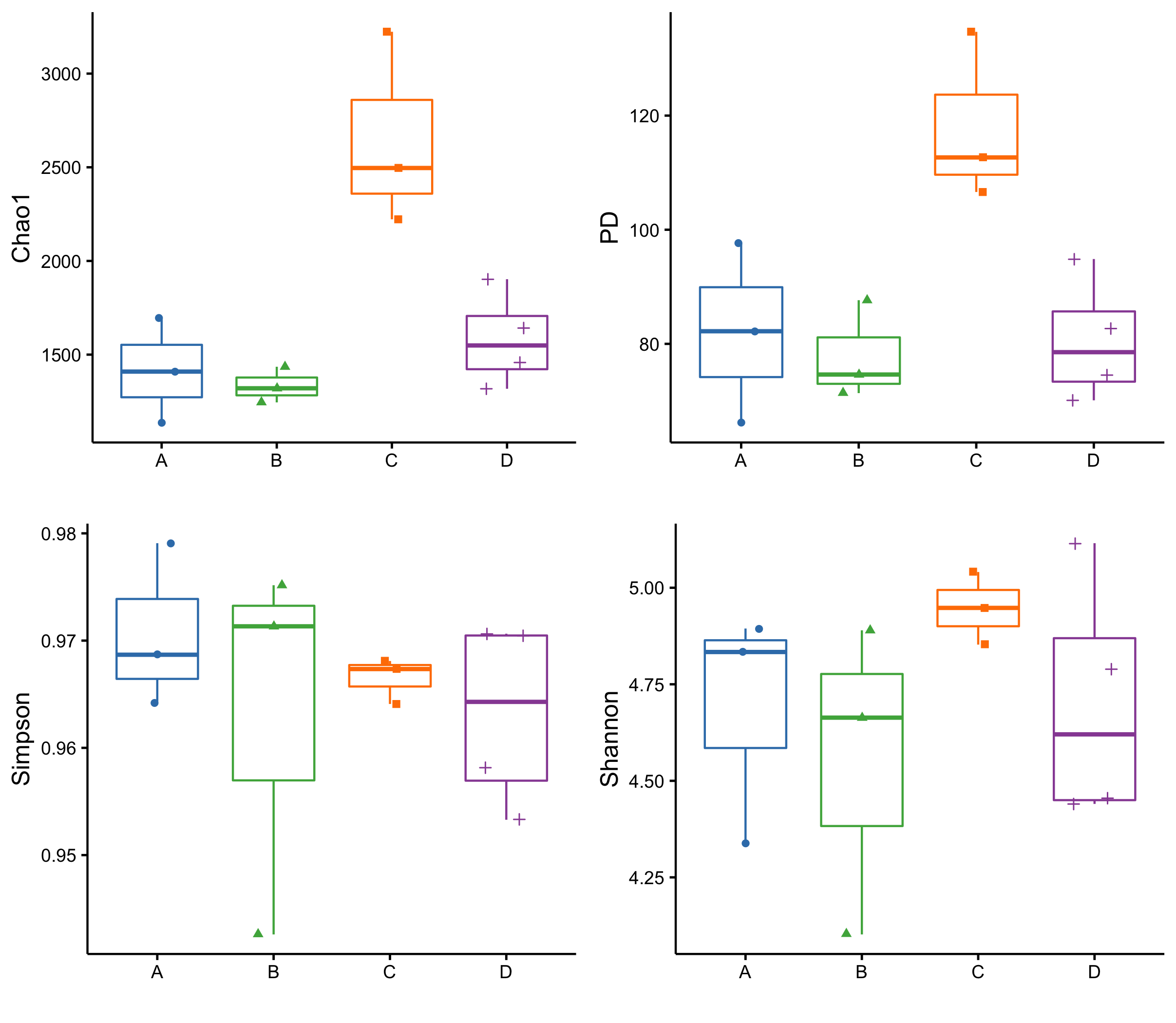

Supplement: Supplementary file 2 [file DataSheet1.zip › 16S rRNA/Images/Alpha_diversity_boxplot.png]

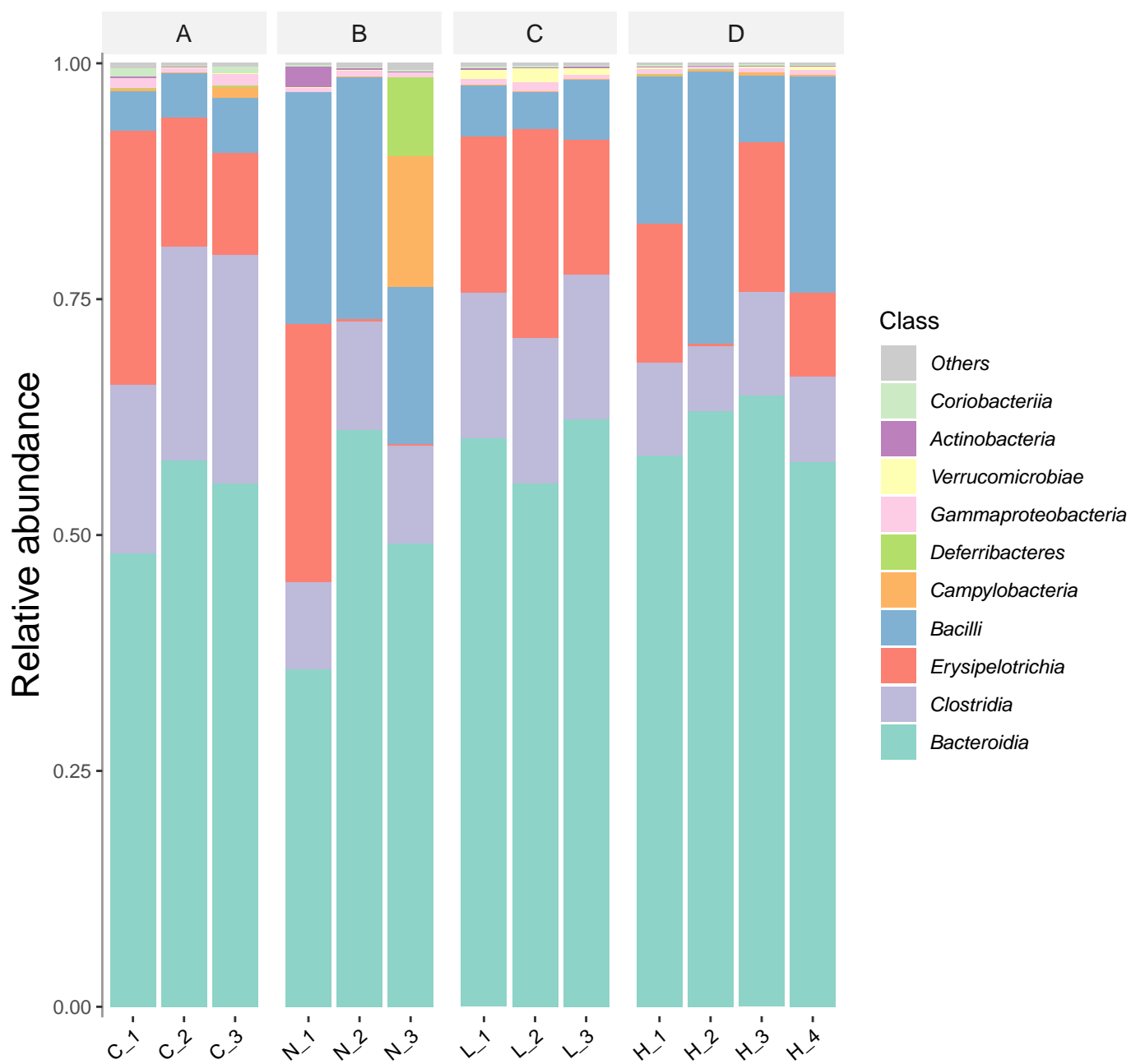

Supplement: Supplementary file 2 [file DataSheet1.zip › 16S rRNA/Images/Barplot_Class.pdf]

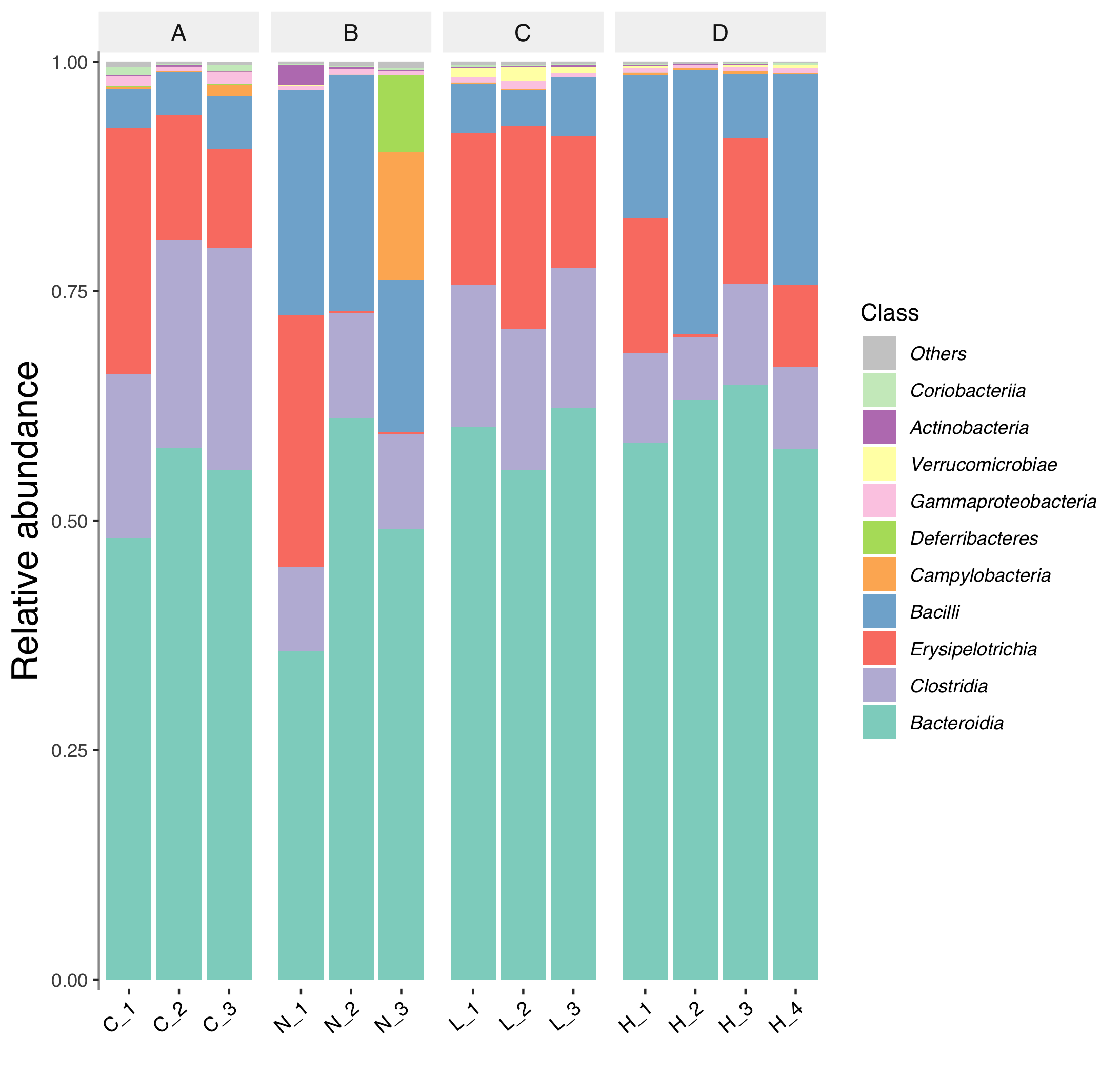

Supplement: Supplementary file 2 [file DataSheet1.zip › 16S rRNA/Images/Barplot_Class.png]

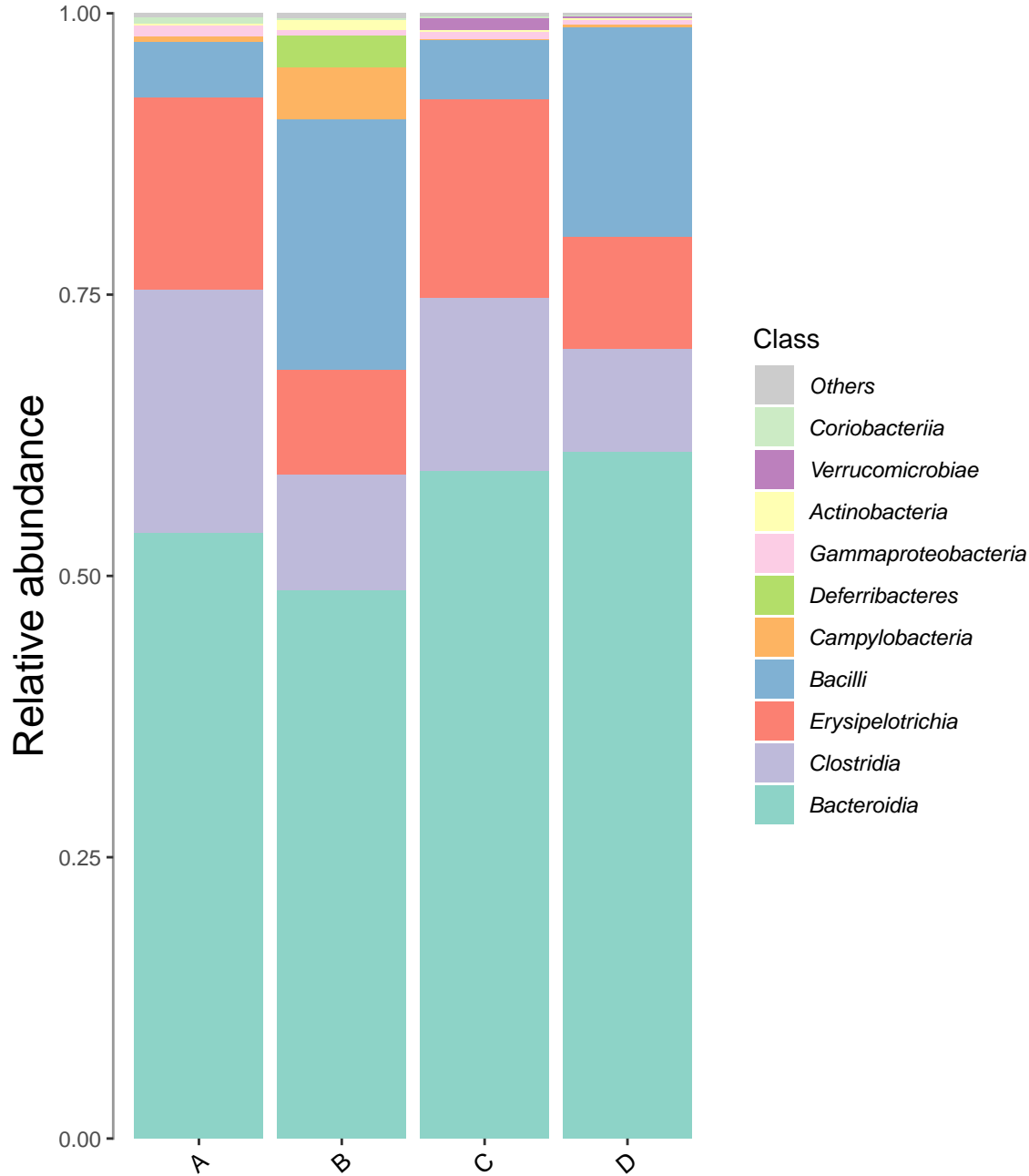

Supplement: Supplementary file 2 [file DataSheet1.zip › 16S rRNA/Images/Barplot_Class_mean.pdf]

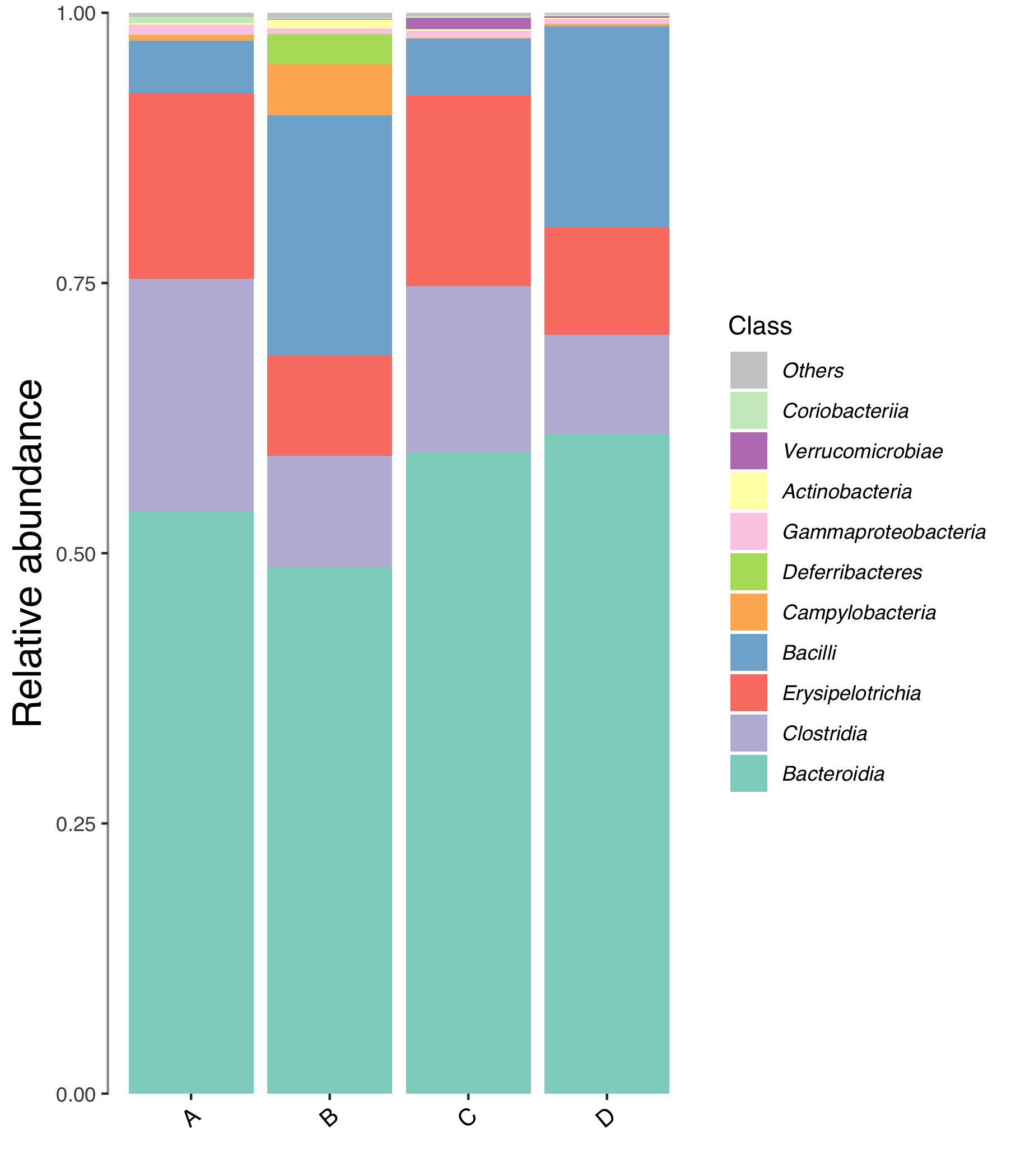

Supplement: Supplementary file 2 [file DataSheet1.zip › 16S rRNA/Images/Barplot_Class_mean.png]

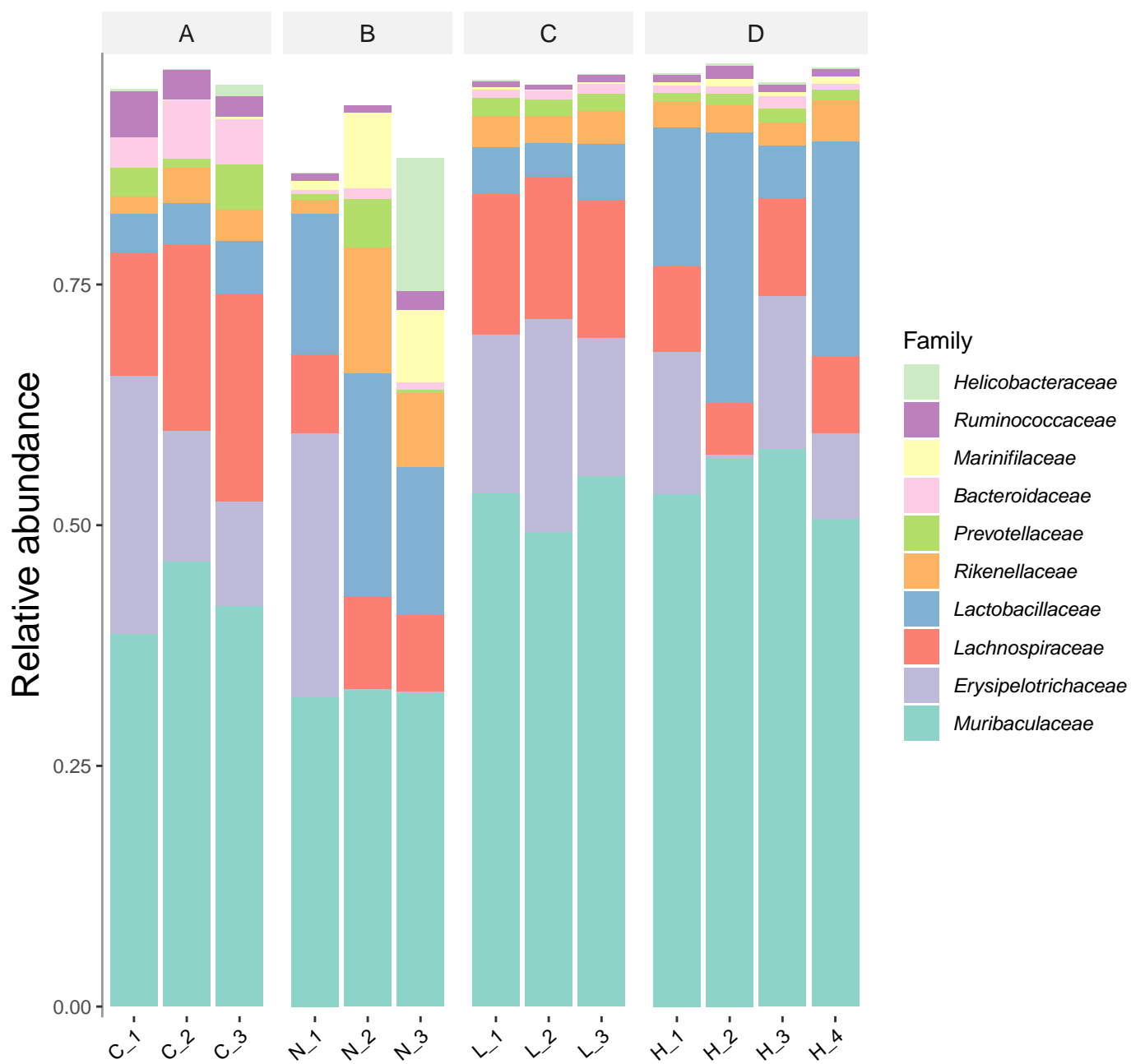

Supplement: Supplementary file 2 [file DataSheet1.zip › 16S rRNA/Images/Barplot_Family.pdf]

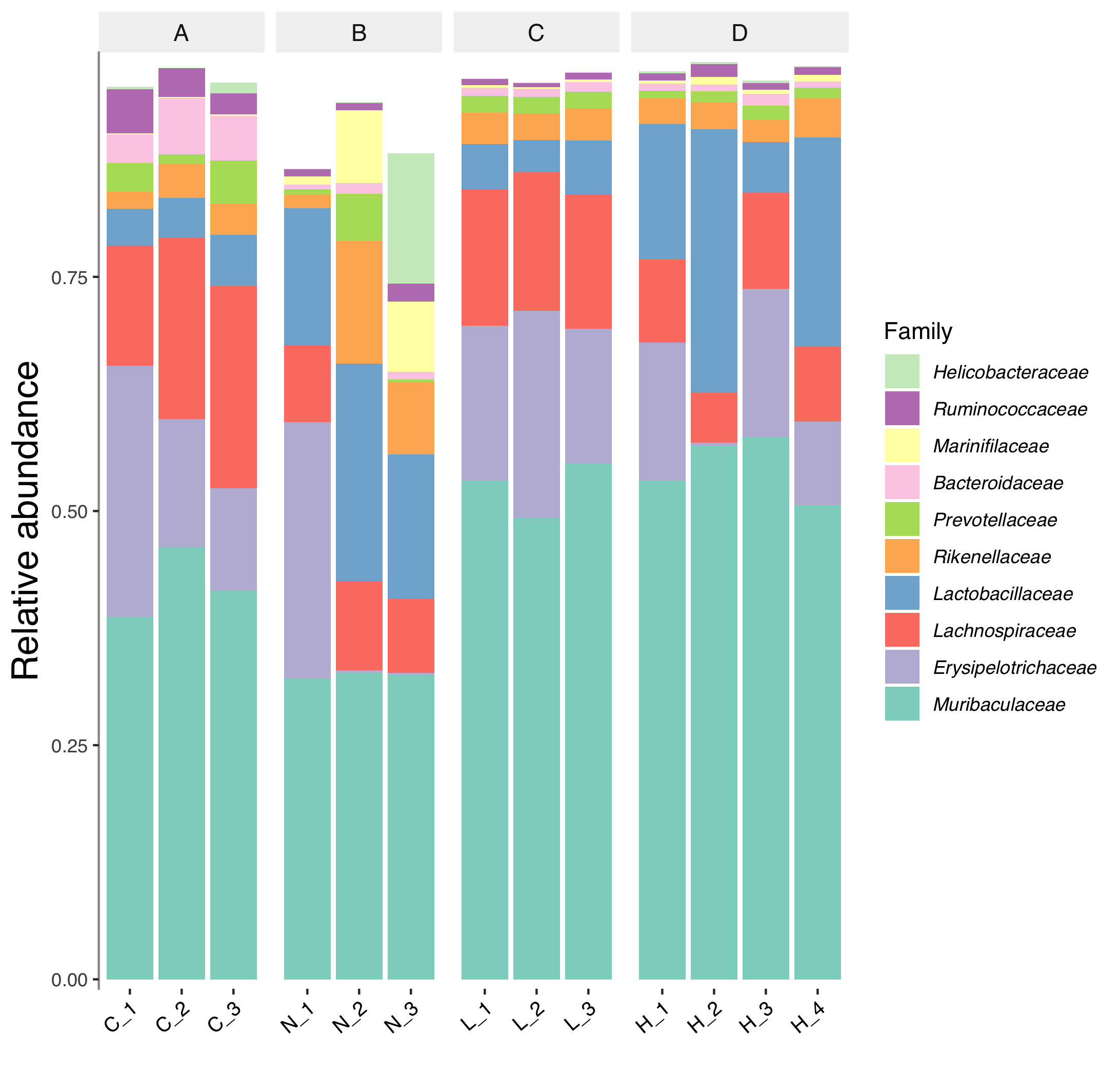

Supplement: Supplementary file 2 [file DataSheet1.zip › 16S rRNA/Images/Barplot_Family.png]

Relative abundance

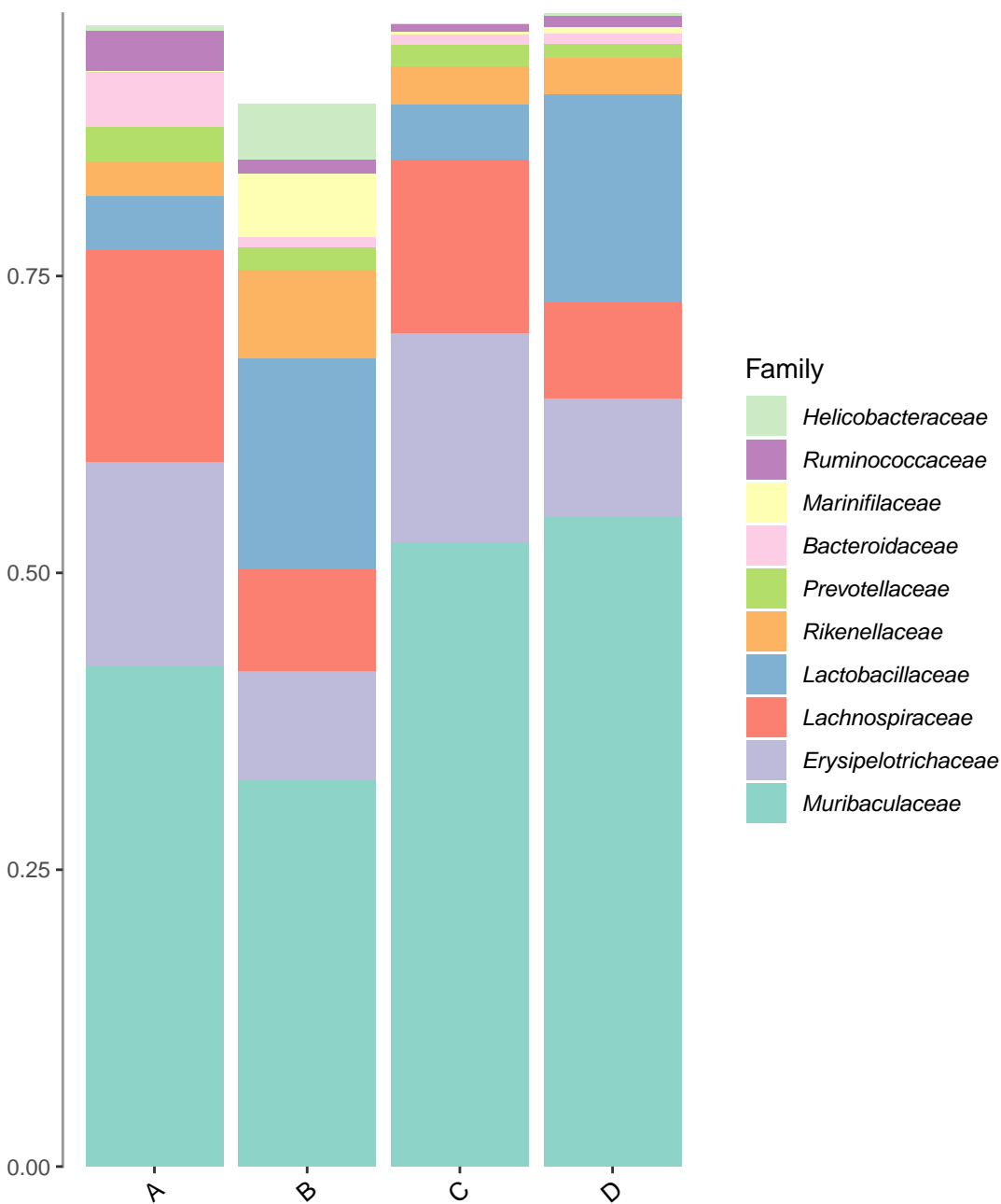

Supplement: Supplementary file 2 [file DataSheet1.zip › 16S rRNA/Images/Barplot_Family_mean.pdf]

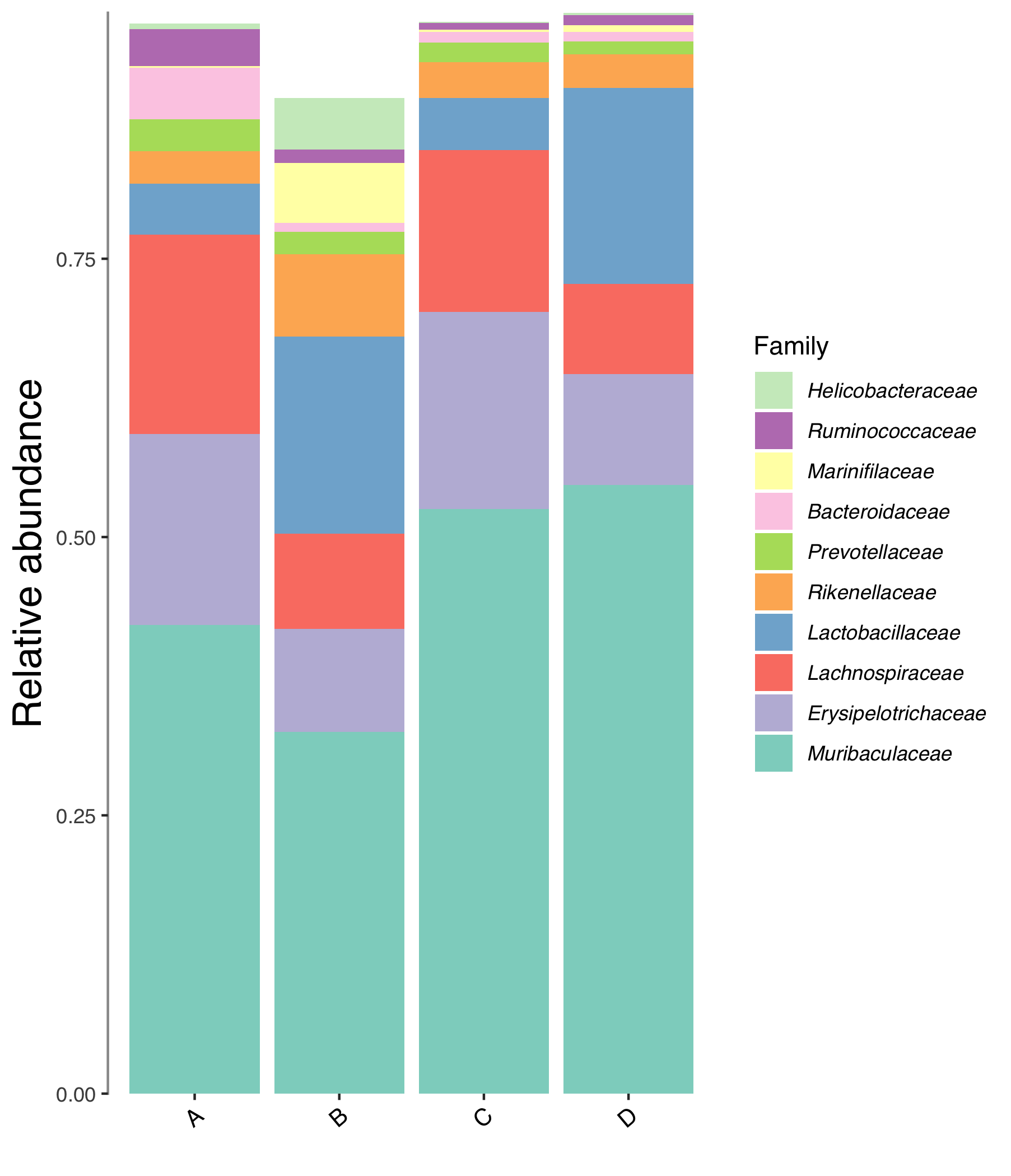

Supplement: Supplementary file 2 [file DataSheet1.zip › 16S rRNA/Images/Barplot_Family_mean.png]

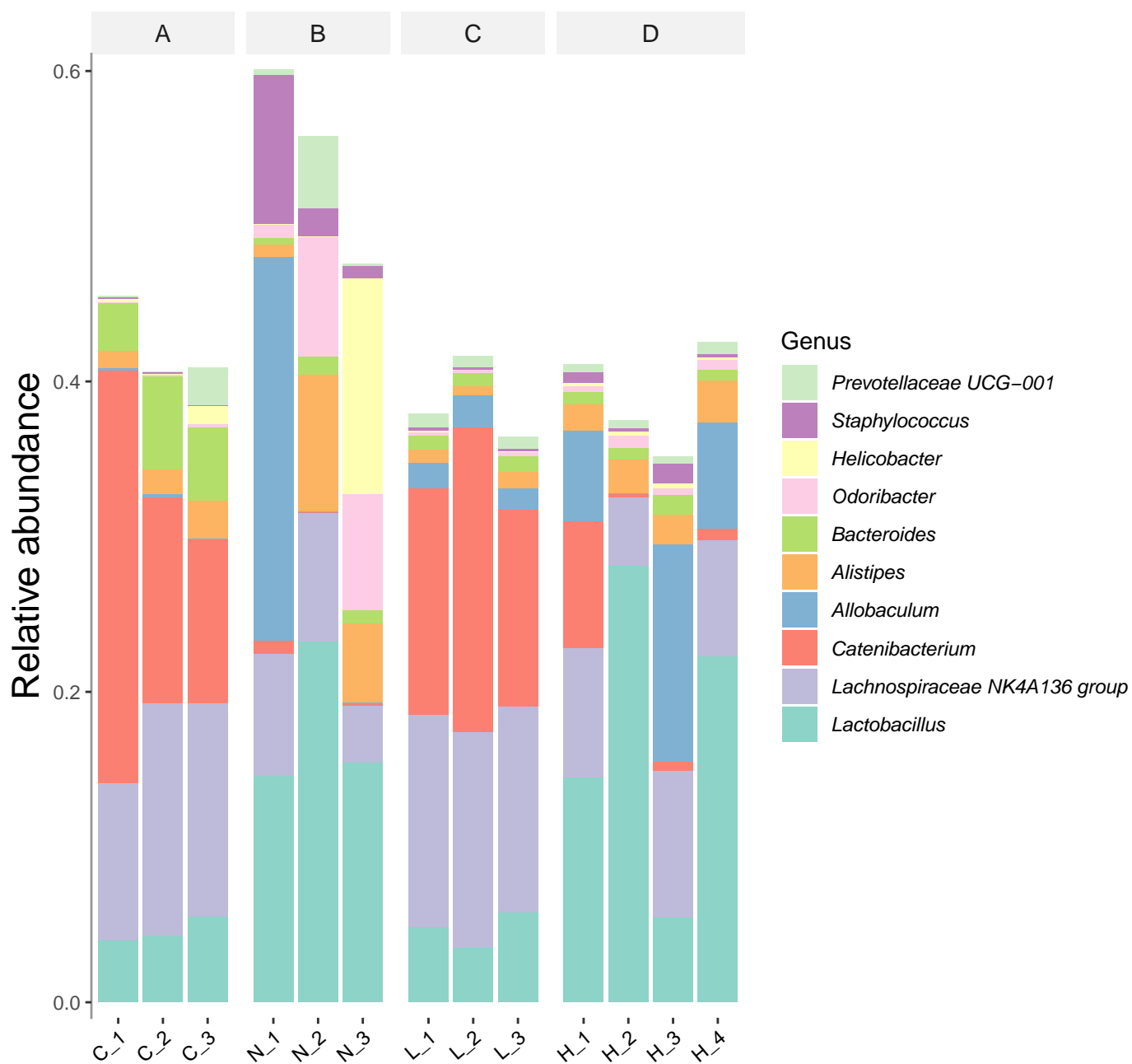

Supplement: Supplementary file 2 [file DataSheet1.zip › 16S rRNA/Images/Barplot_Genus.pdf]

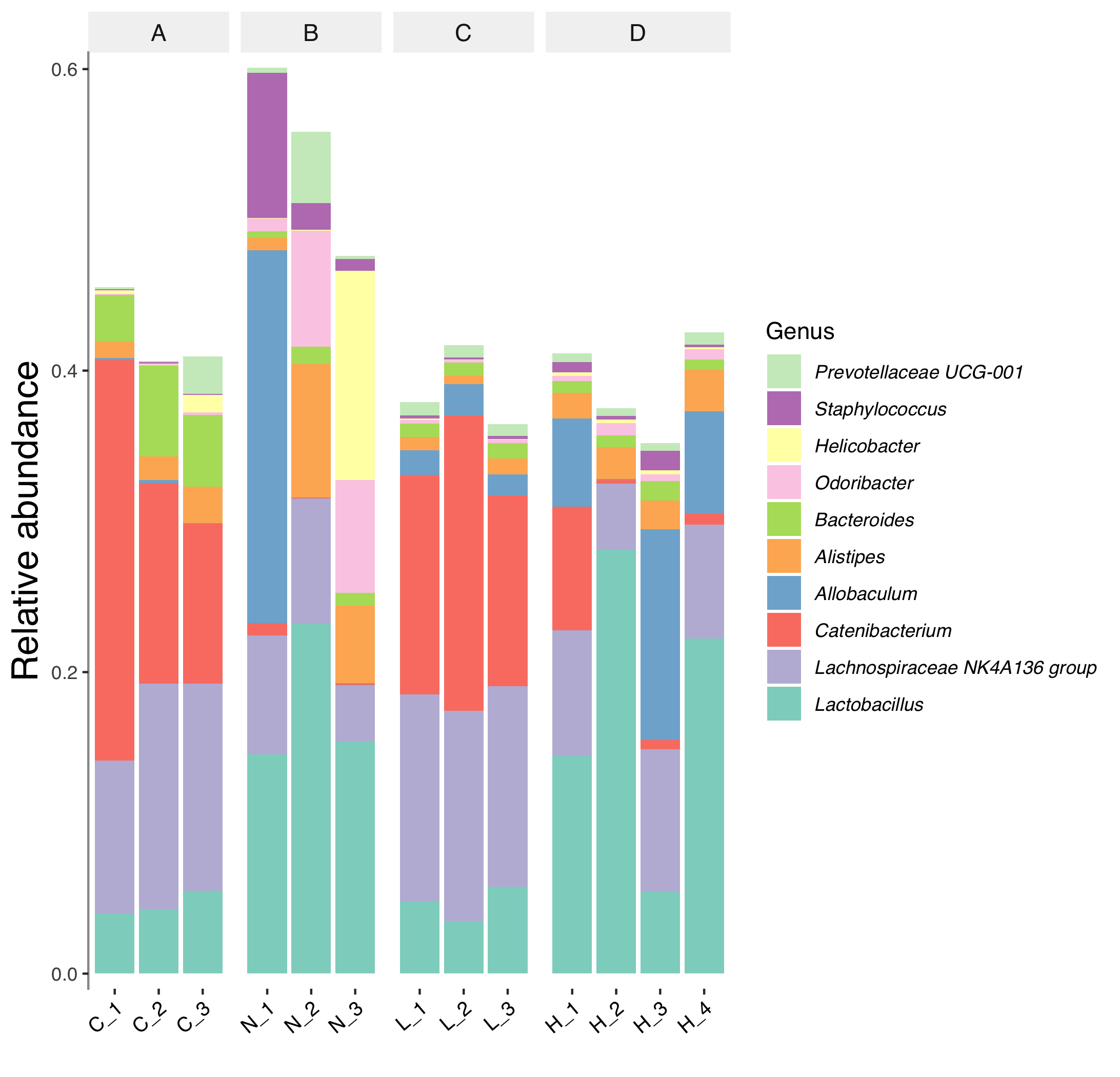

Supplement: Supplementary file 2 [file DataSheet1.zip › 16S rRNA/Images/Barplot_Genus.png]

Relative abundance

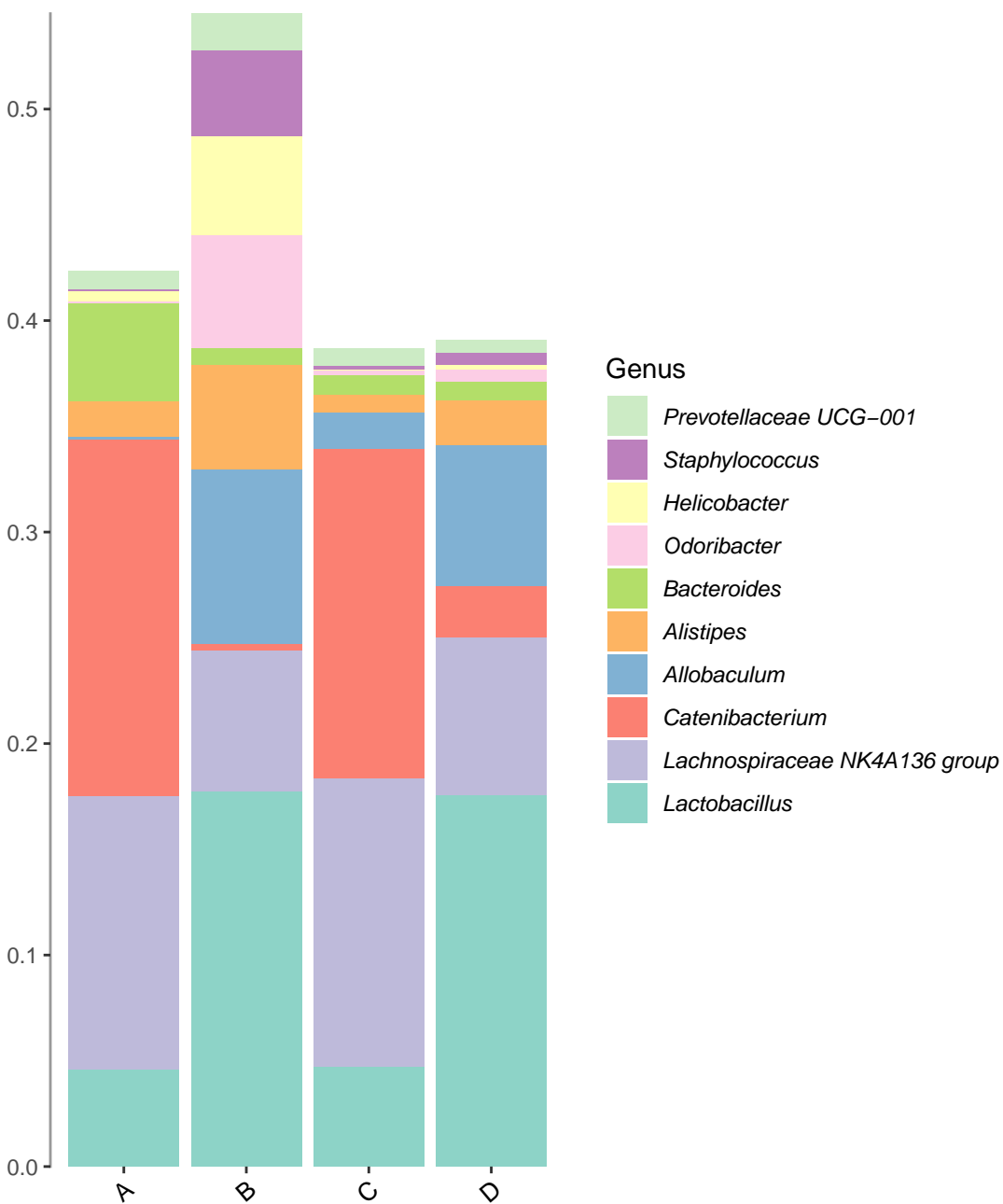

Supplement: Supplementary file 2 [file DataSheet1.zip › 16S rRNA/Images/Barplot_Genus_mean.pdf]

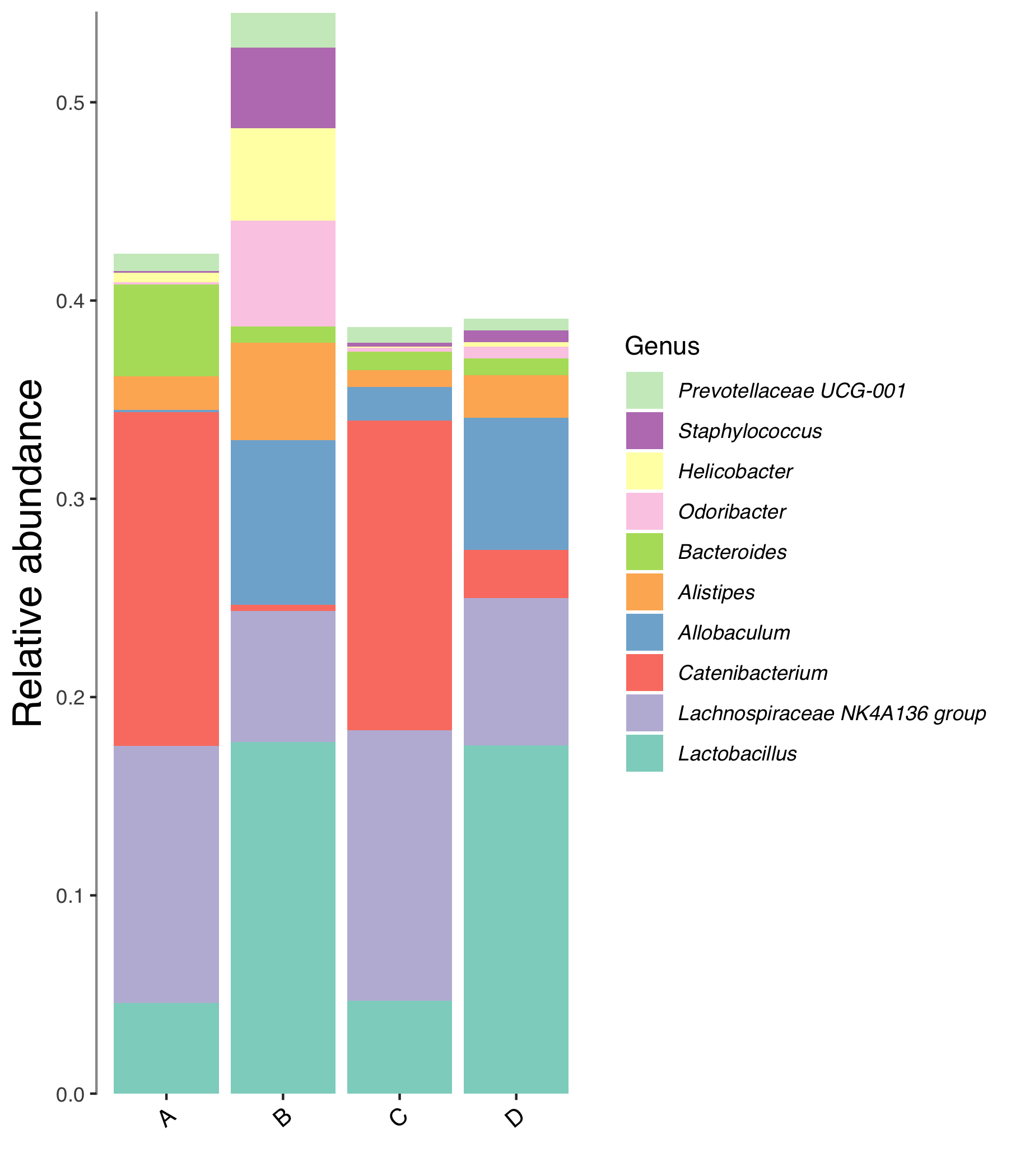

Supplement: Supplementary file 2 [file DataSheet1.zip › 16S rRNA/Images/Barplot_Genus_mean.png]

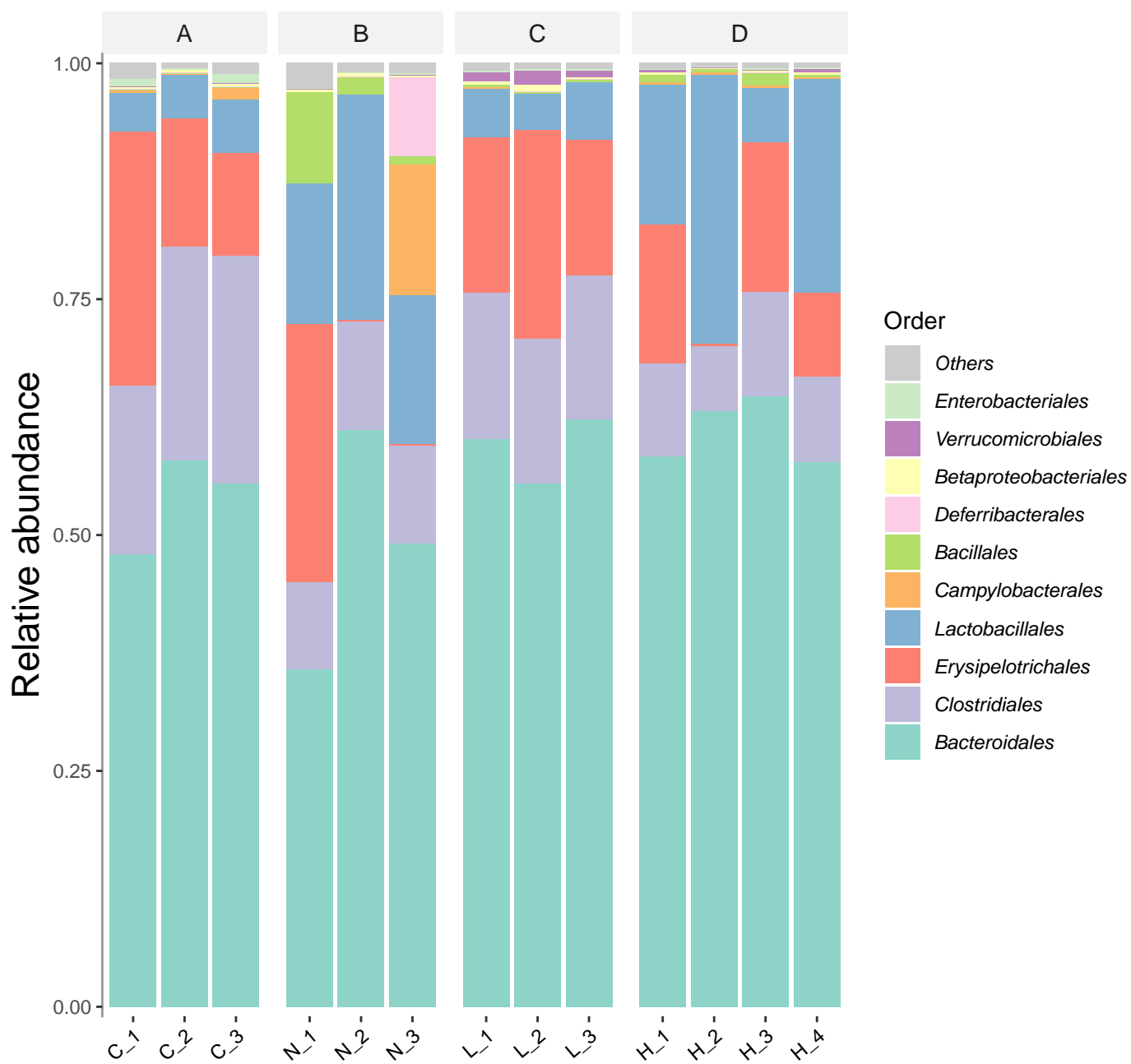

Supplement: Supplementary file 2 [file DataSheet1.zip › 16S rRNA/Images/Barplot_Order.pdf]

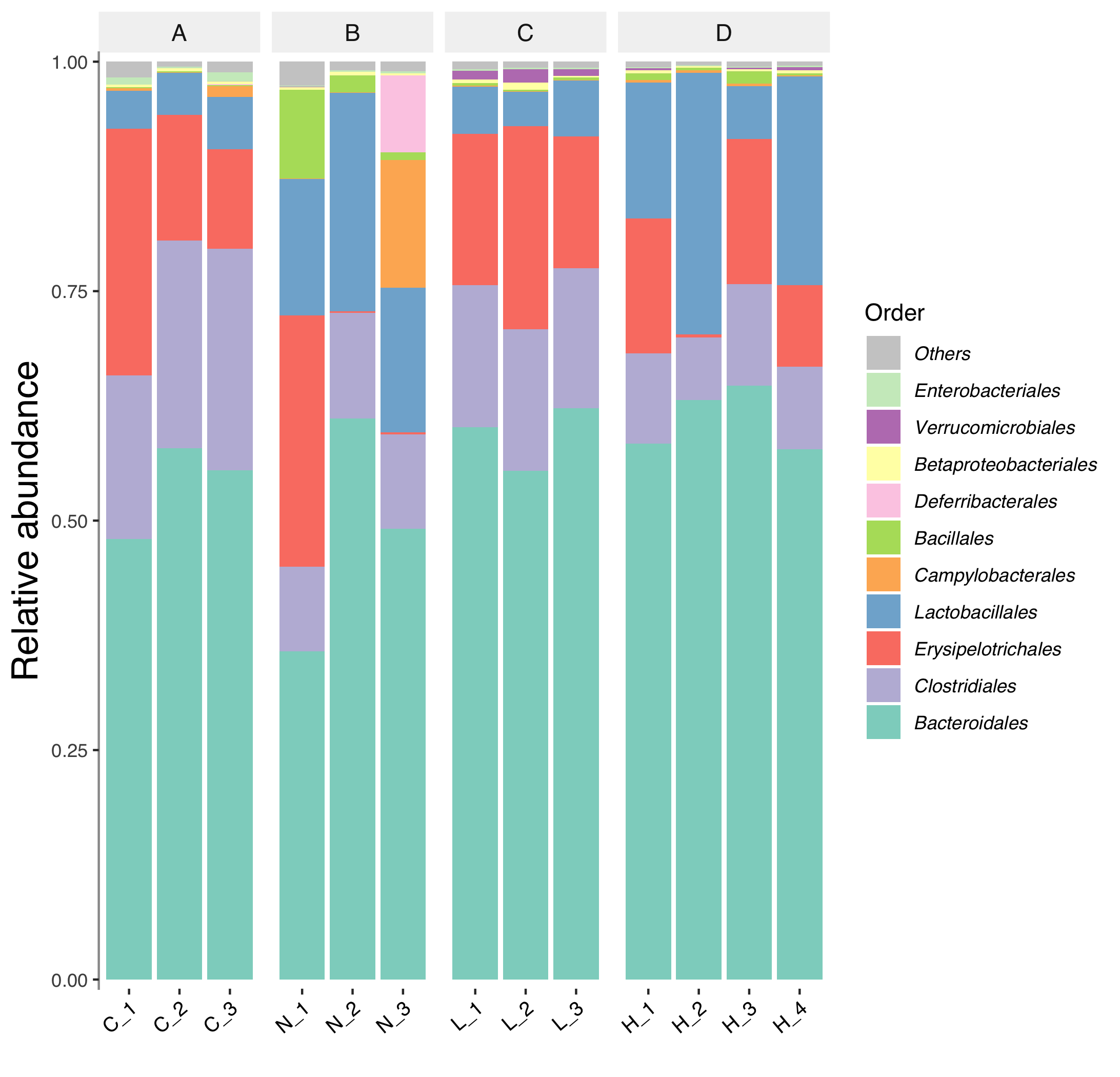

Supplement: Supplementary file 2 [file DataSheet1.zip › 16S rRNA/Images/Barplot_Order.png]

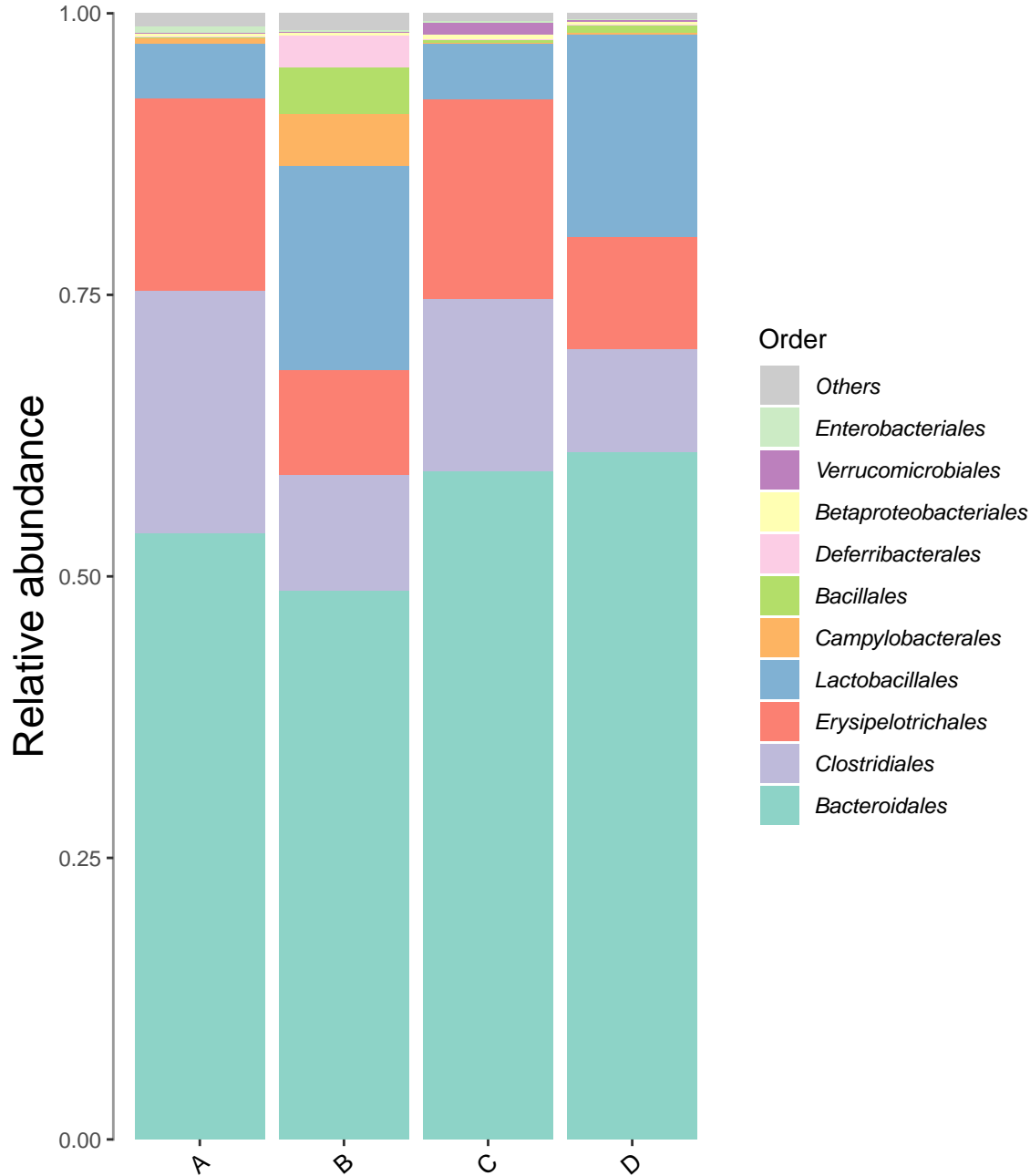

Supplement: Supplementary file 2 [file DataSheet1.zip › 16S rRNA/Images/Barplot_Order_mean.pdf]

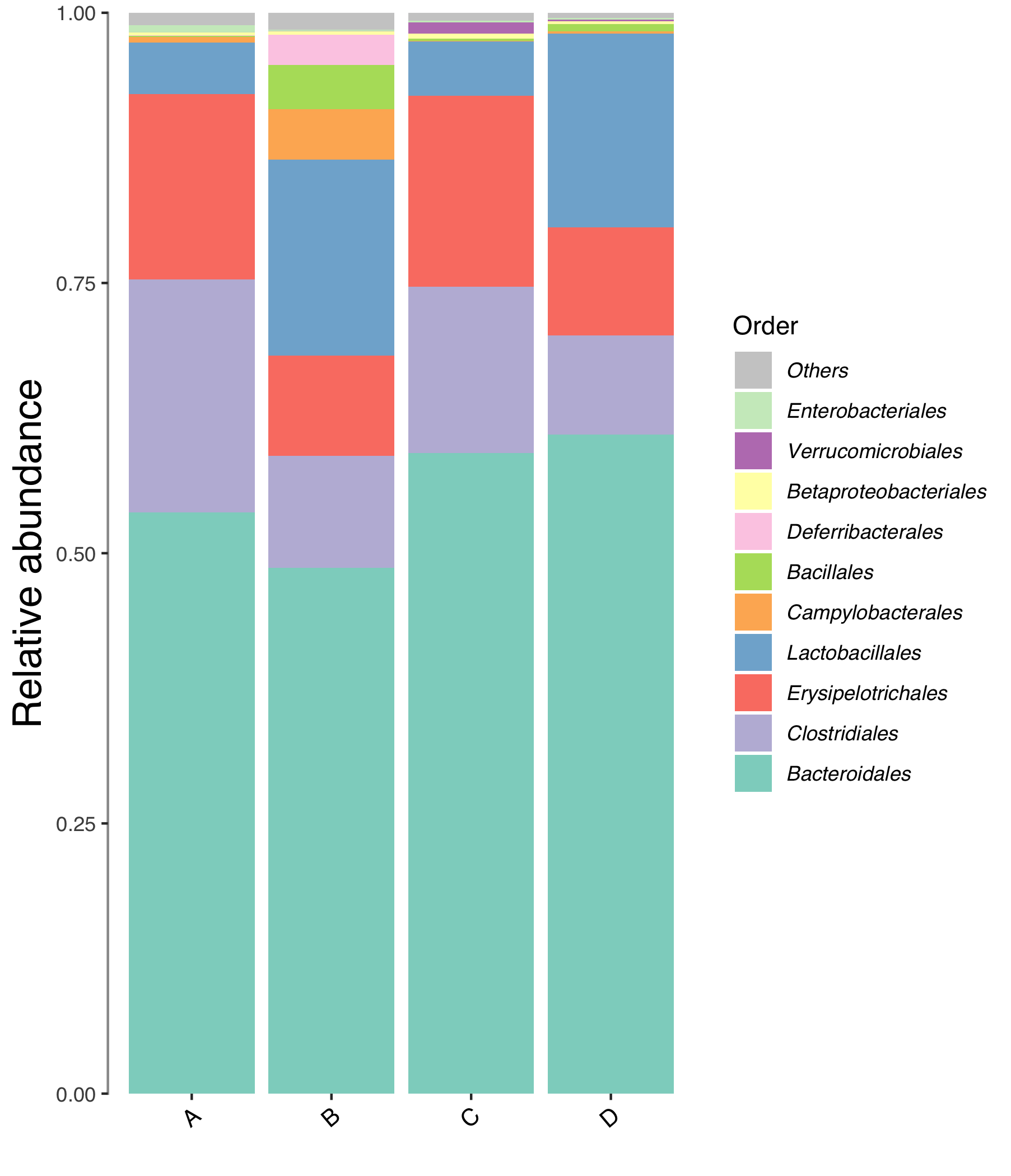

Supplement: Supplementary file 2 [file DataSheet1.zip › 16S rRNA/Images/Barplot_Order_mean.png]

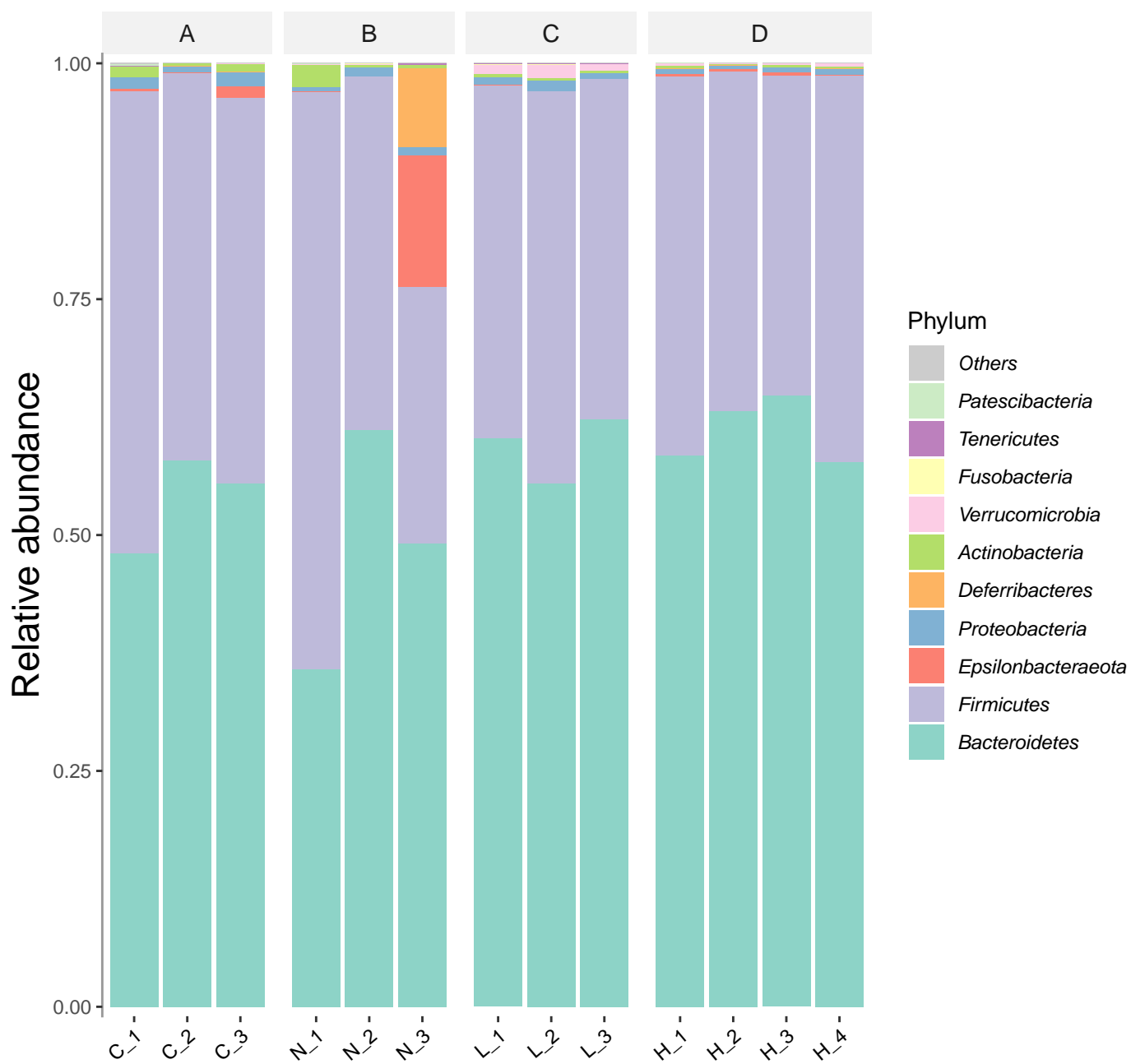

Supplement: Supplementary file 2 [file DataSheet1.zip › 16S rRNA/Images/Barplot_Phylum.pdf]

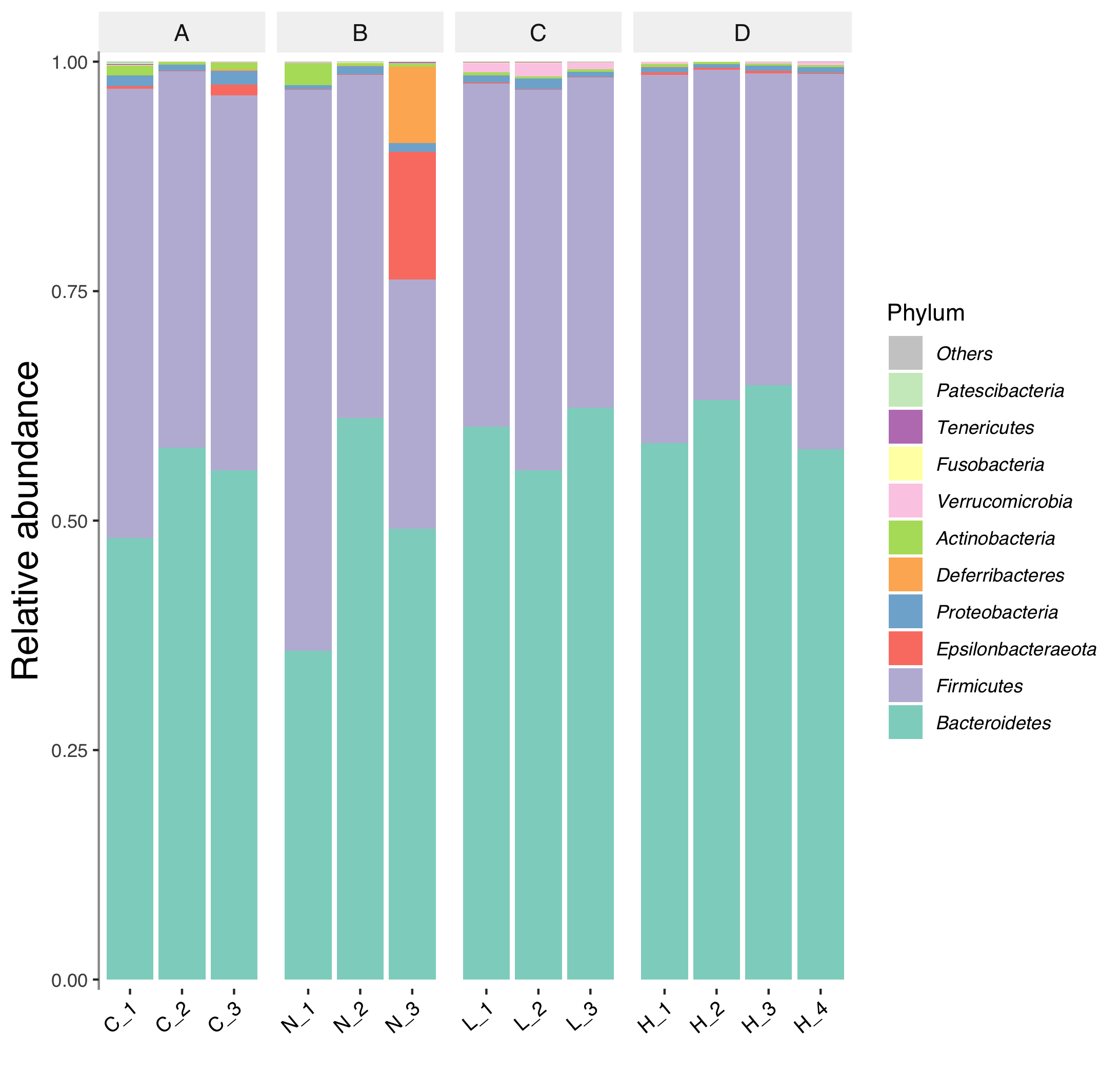

Supplement: Supplementary file 2 [file DataSheet1.zip › 16S rRNA/Images/Barplot_Phylum.png]

Relative abundance

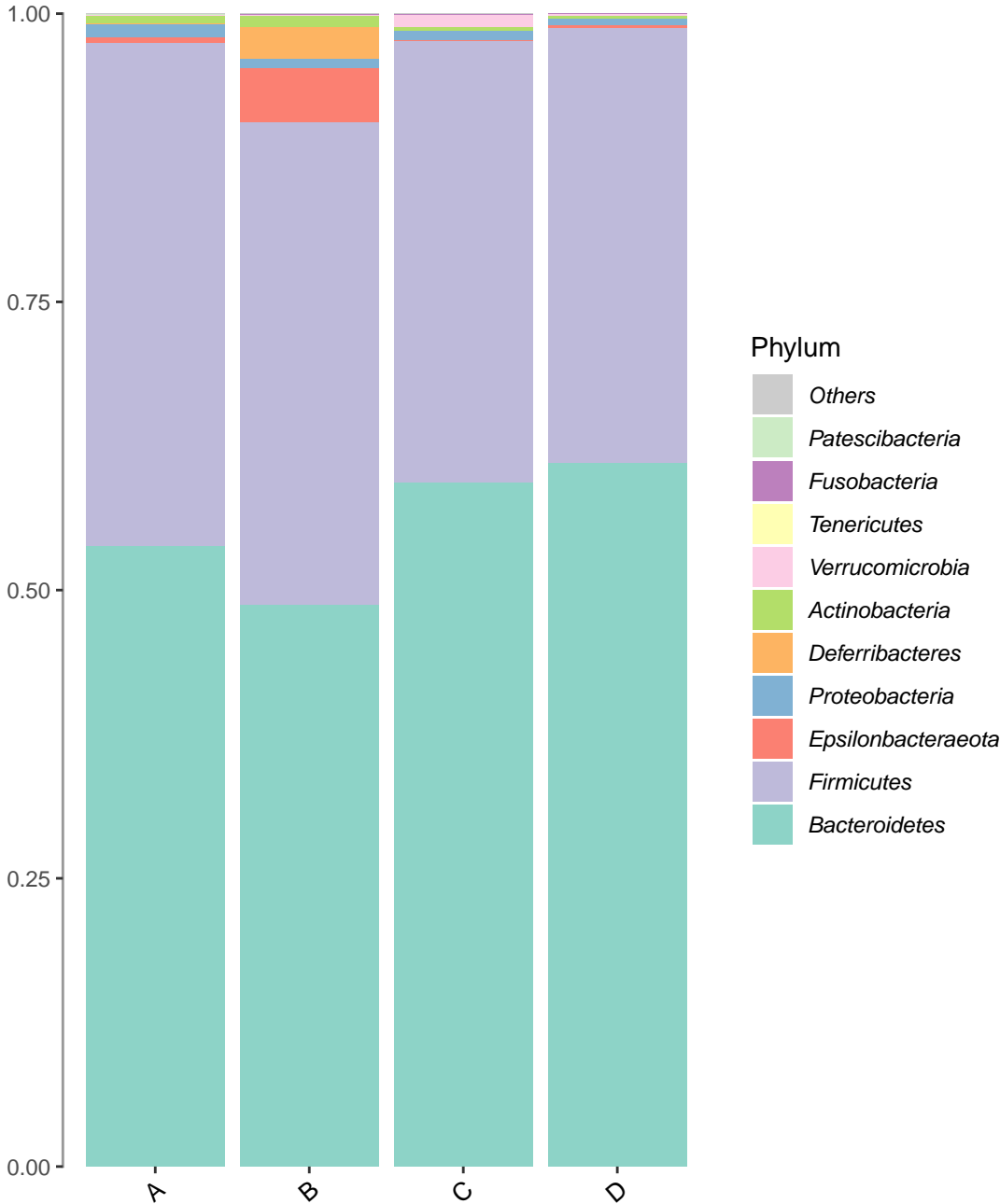

Supplement: Supplementary file 2 [file DataSheet1.zip › 16S rRNA/Images/Barplot_Phylum_mean.pdf]

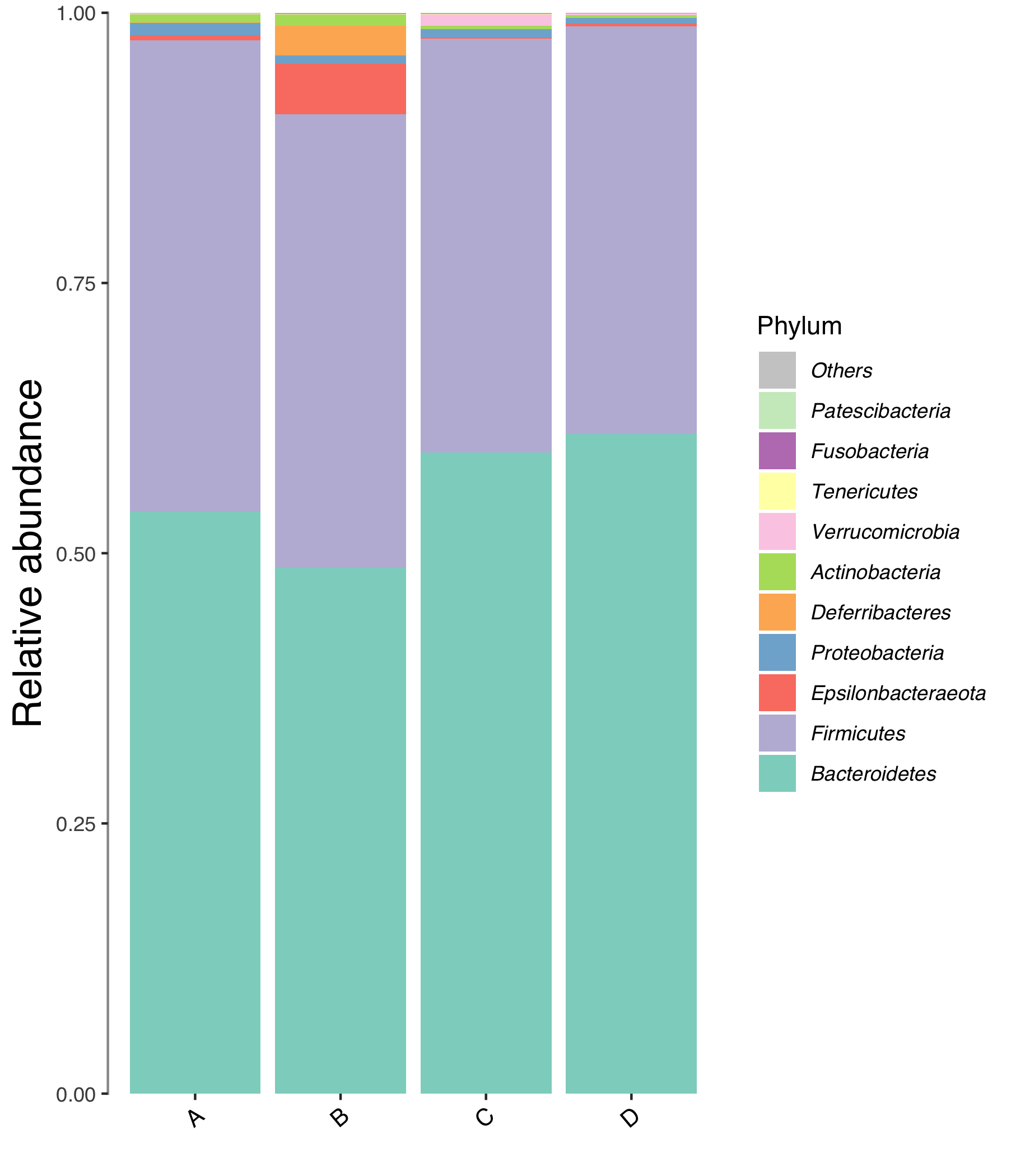

Supplement: Supplementary file 2 [file DataSheet1.zip › 16S rRNA/Images/Barplot_Phylum_mean.png]

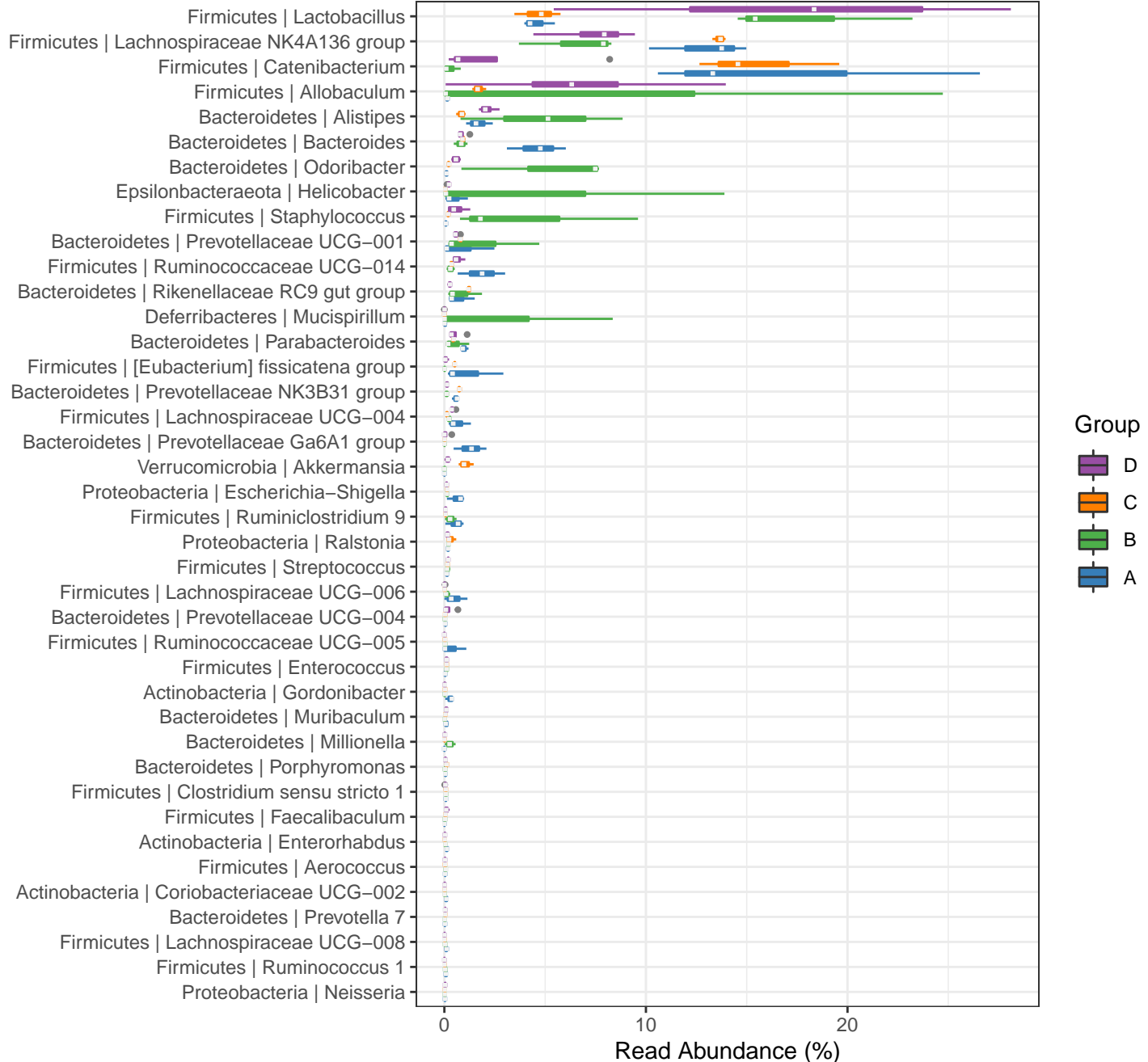

Supplement: Supplementary file 2 [file DataSheet1.zip › 16S rRNA/Images/Boxplot_Genus.pdf]

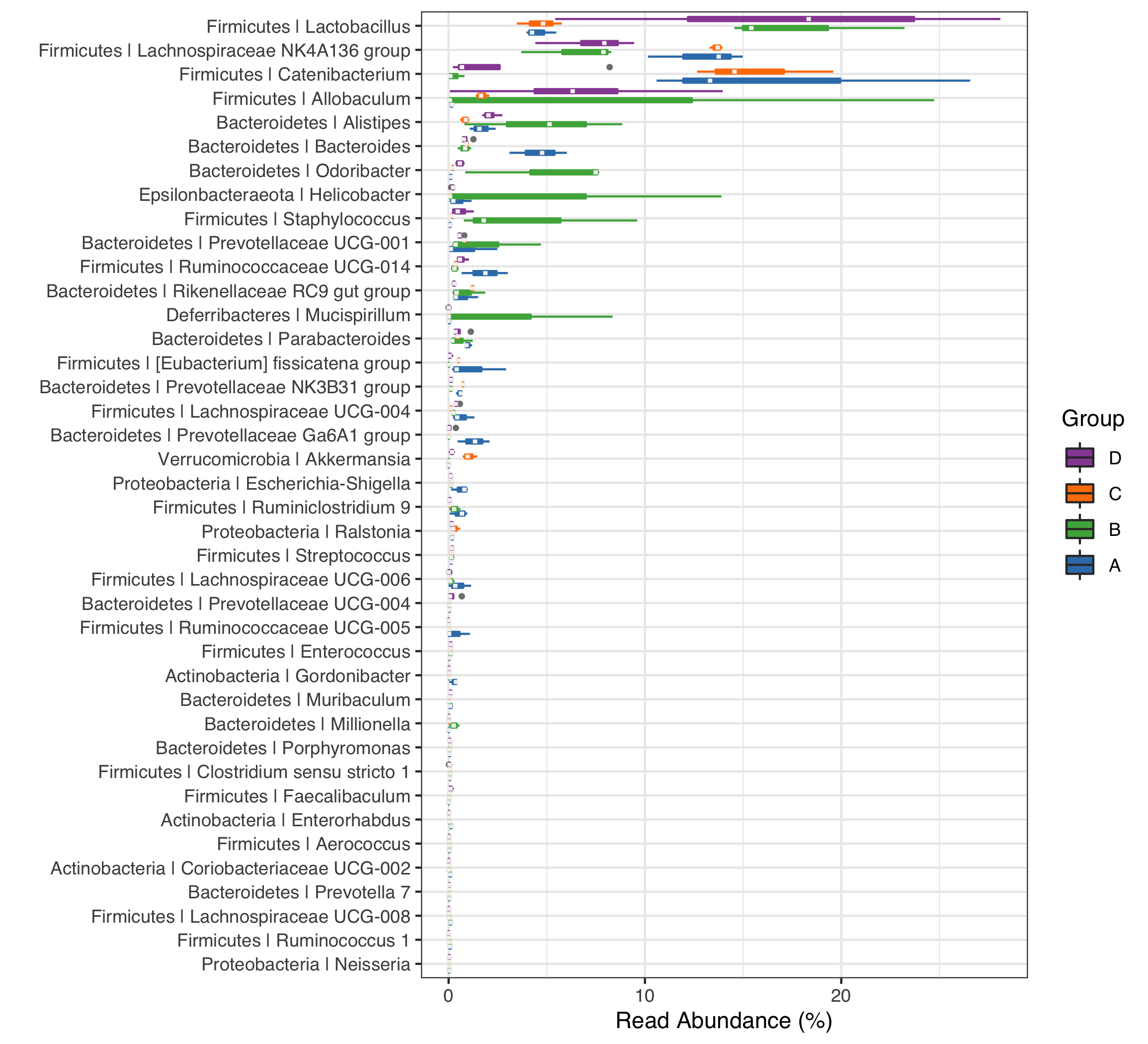

Supplement: Supplementary file 2 [file DataSheet1.zip › 16S rRNA/Images/Boxplot_Genus.png]
