# Supplementary material for: Neuroprotective Effects of Oligosaccharides From Periplaneta Americana on Parkinson’s Disease Models In Vitro and In Vivo
Source: Front Pharmacol. 2022 Jul 18;13:936818. doi: 10.3389/fphar.2022.936818 (PMC9340460; doi:10.3389/fphar.2022.936818)
Supplement: Supplementary file 2 [file DataSheet1.zip › 16S rRNA/Images/CV_table_genus_B.pdf]

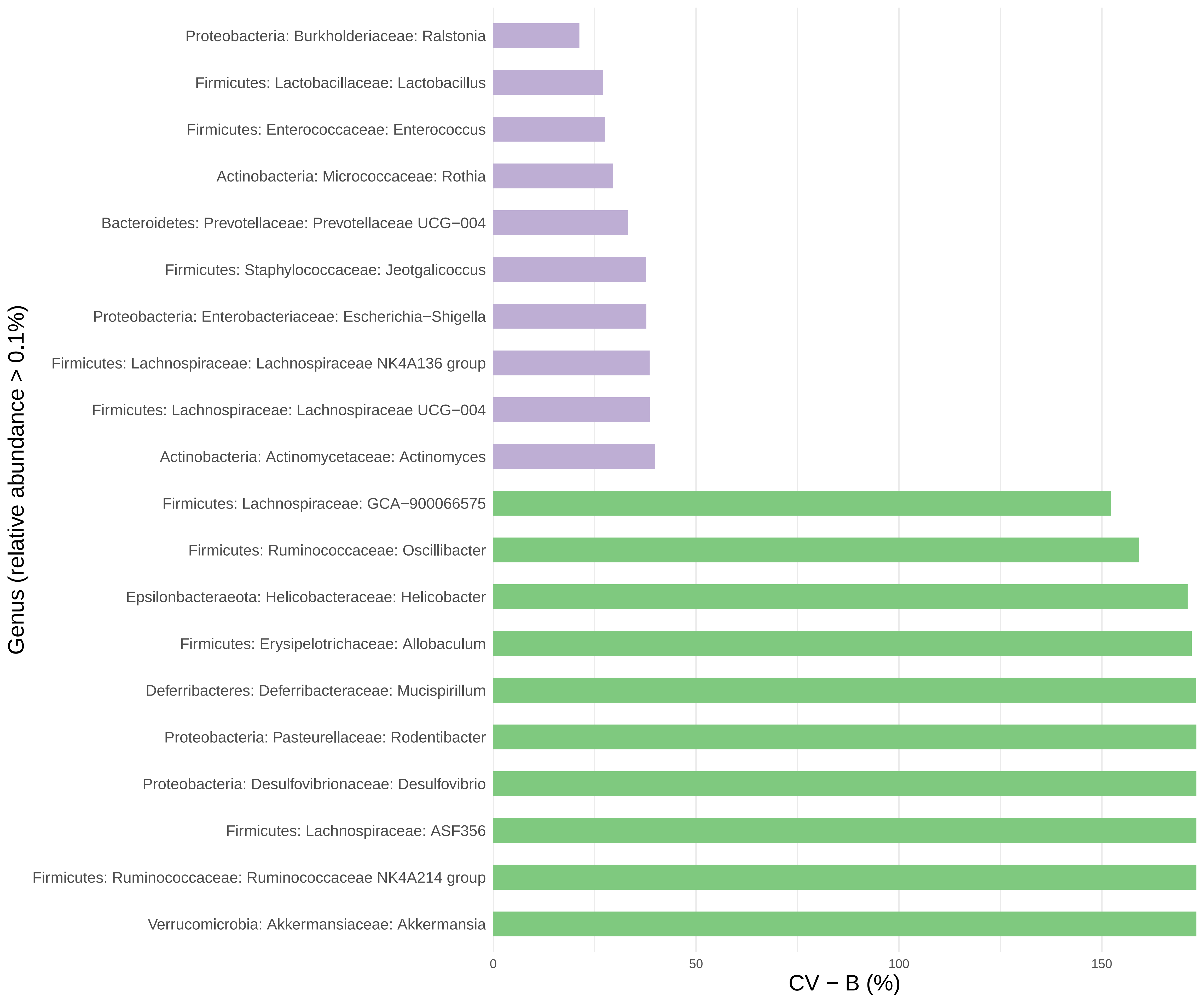
Proteobacteria: Burkholderiaceae: Ralstonia
Firmicutes: Lactobacillaceae: Lactobacillus
Firmicutes: Enterococcaceae: Enterococcus
Actinobacteria: Micrococcaceae: Rothia
Bacteroidetes: Prevotellaceae: Prevotellaceae UCG−004
Firmicutes: Staphylococcaceae: Jeotgalicoccus
Proteobacteria: Enterobacteriaceae: Escherichia−Shigella
Firmicutes: Lachnospiraceae: Lachnospiraceae NK4A136 group
Firmicutes: Lachnospiraceae: Lachnospiraceae UCG−004
Actinobacteria: Actinomycetaceae: Actinomyces
Firmicutes: Lachnospiraceae: GCA−900066575
Firmicutes: Ruminococcaceae: Oscillibacter
Epsilonbacteraeota: Helicobacteraceae: Helicobacter
Firmicutes: Erysipelotrichaceae: Allobaculum
Deferribacteres: Deferribacteraceae: Mucispirillum
Proteobacteria: Pasteurellaceae: Rodentibacter
Proteobacteria: Desulfovibrionaceae: Desulfovibrio
Firmicutes: Lachnospiraceae: ASF356
Firmicutes: Ruminococcaceae: Ruminococcaceae NK4A214 group
Verrucomicrobia: Akkermansiaceae: Akkermansia
Genus (relative abundance > 0.1%)
0
50
100
150
CV − B (%)
