# Supplementary material for: Neuroprotective Effects of Oligosaccharides From Periplaneta Americana on Parkinson’s Disease Models In Vitro and In Vivo
Source: Front Pharmacol. 2022 Jul 18;13:936818. doi: 10.3389/fphar.2022.936818 (PMC9340460; doi:10.3389/fphar.2022.936818)
Supplement: Supplementary file 2 [file DataSheet1.zip › 16S rRNA/Images/CV_table_genus_C.pdf]

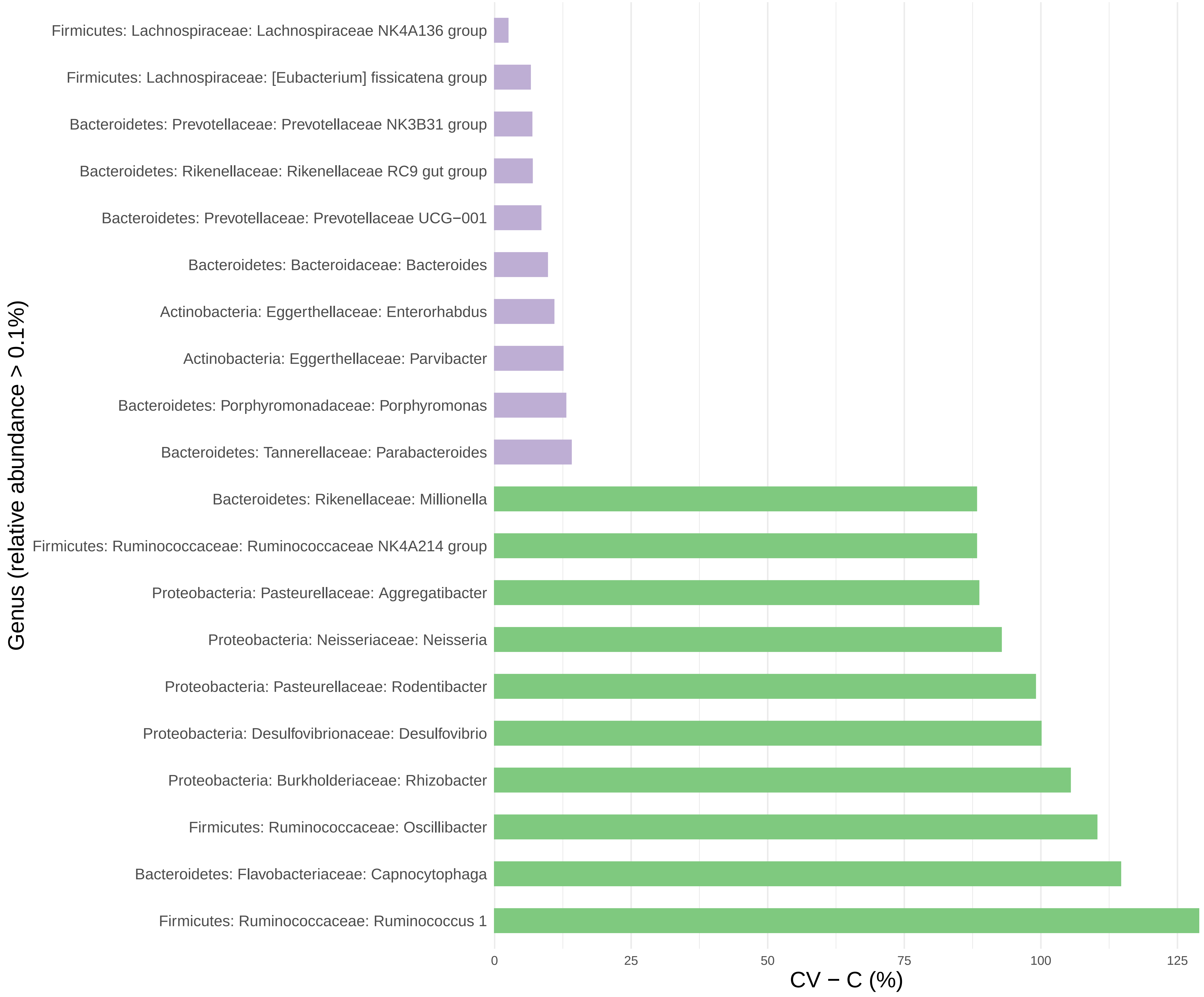
Firmicutes: Lachnospiraceae: Lachnospiraceae NK4A136 group
Firmicutes: Lachnospiraceae: [Eubacterium] fissicatena group
Bacteroidetes: Prevotellaceae: Prevotellaceae NK3B31 group
Bacteroidetes: Rikenellaceae: Rikenellaceae RC9 gut group
Bacteroidetes: Prevotellaceae: Prevotellaceae UCG−001
Bacteroidetes: Bacteroidaceae: Bacteroides
Actinobacteria: Eggerthellaceae: Enterorhabdus
Actinobacteria: Eggerthellaceae: Parvibacter
Bacteroidetes: Porphyromonadaceae: Porphyromonas
Bacteroidetes: Tannerellaceae: Parabacteroides
Bacteroidetes: Rikenellaceae: Millionella
Firmicutes: Ruminococcaceae: Ruminococcaceae NK4A214 group
Proteobacteria: Pasteurellaceae: Aggregatibacter
Proteobacteria: Neisseriaceae: Neisseria
Proteobacteria: Pasteurellaceae: Rodentibacter
Proteobacteria: Desulfovibrionaceae: Desulfovibrio
Proteobacteria: Burkholderiaceae: Rhizobacter
Firmicutes: Ruminococcaceae: Oscillibacter
Bacteroidetes: Flavobacteriaceae: Capnocytophaga
Firmicutes: Ruminococcaceae: Ruminococcus 1
Genus (relative abundance > 0.1%)
0
25
50
75
100
125
CV − C (%)
