# Supplementary material for: Neuroprotective Effects of Oligosaccharides From Periplaneta Americana on Parkinson’s Disease Models In Vitro and In Vivo
Source: Front Pharmacol. 2022 Jul 18;13:936818. doi: 10.3389/fphar.2022.936818 (PMC9340460; doi:10.3389/fphar.2022.936818)
Supplement: Supplementary file 2 [file DataSheet1.zip › 16S rRNA/Images/CV_table_genus_D.pdf]

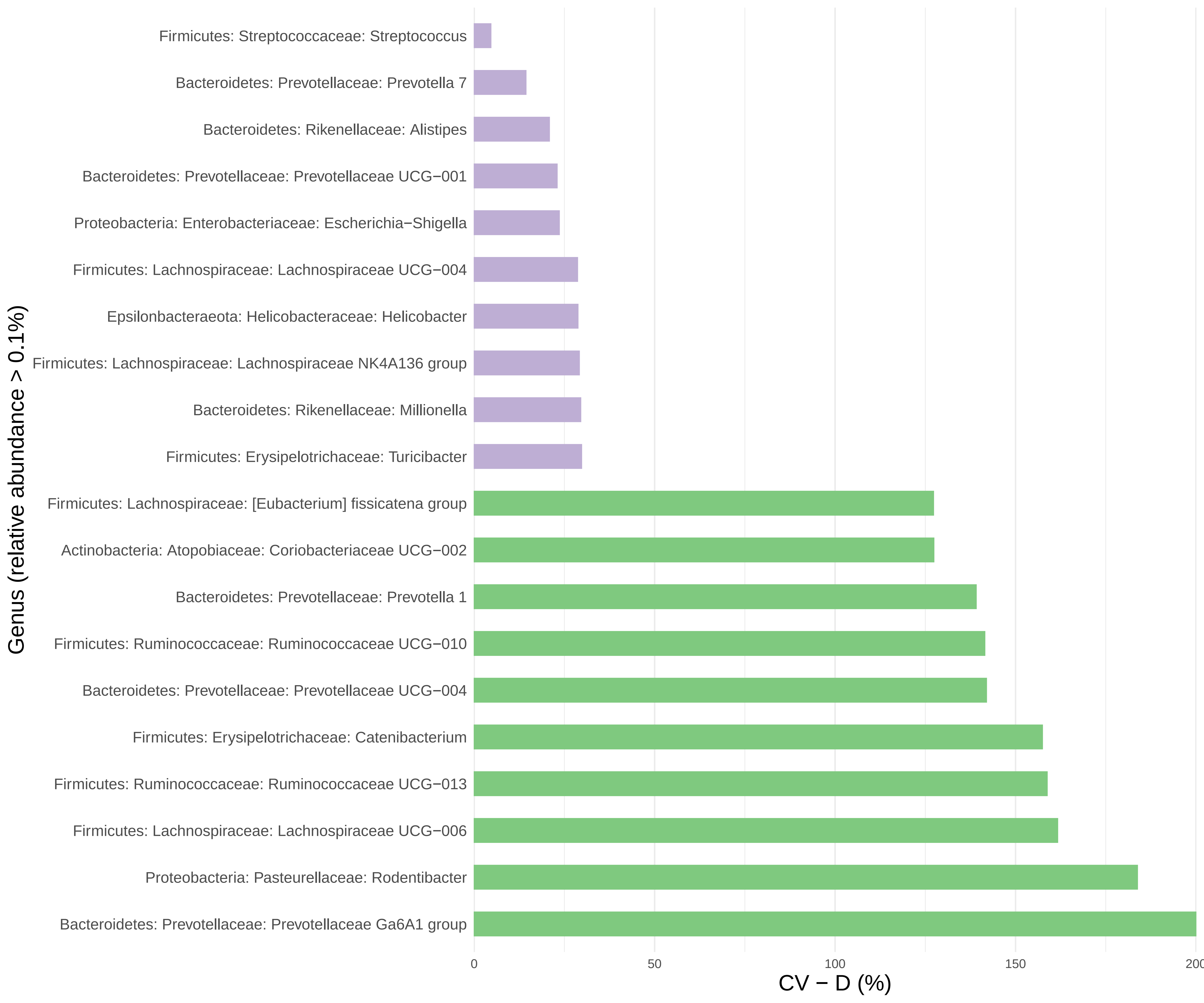
Firmicutes: Streptococcaceae: Streptococcus
Bacteroidetes: Prevotellaceae: Prevotella 7
Bacteroidetes: Rikenellaceae: Alistipes
Bacteroidetes: Prevotellaceae: Prevotellaceae UCG−001
Proteobacteria: Enterobacteriaceae: Escherichia−Shigella
Firmicutes: Lachnospiraceae: Lachnospiraceae UCG−004
Epsilonbacteraeota: Helicobacteraceae: Helicobacter
Firmicutes: Lachnospiraceae: Lachnospiraceae NK4A136 group
Bacteroidetes: Rikenellaceae: Millionella
Firmicutes: Erysipelotrichaceae: Turicibacter
Firmicutes: Lachnospiraceae: [Eubacterium] fissicatena group
Actinobacteria: Atopobiaceae: Coriobacteriaceae UCG−002
Bacteroidetes: Prevotellaceae: Prevotella 1
Firmicutes: Ruminococcaceae: Ruminococcaceae UCG−010
Bacteroidetes: Prevotellaceae: Prevotellaceae UCG−004
Firmicutes: Erysipelotrichaceae: Catenibacterium
Firmicutes: Ruminococcaceae: Ruminococcaceae UCG−013
Firmicutes: Lachnospiraceae: Lachnospiraceae UCG−006
Proteobacteria: Pasteurellaceae: Rodentibacter
Bacteroidetes: Prevotellaceae: Prevotellaceae Ga6A1 group
Genus (relative abundance > 0.1%)
0
50
100
150
200
CV − D (%)
