# Supplementary material for: Neuroprotective Effects of Oligosaccharides From Periplaneta Americana on Parkinson’s Disease Models In Vitro and In Vivo
Source: Front Pharmacol. 2022 Jul 18;13:936818. doi: 10.3389/fphar.2022.936818 (PMC9340460; doi:10.3389/fphar.2022.936818)
Supplement: Supplementary file 2 [file DataSheet1.zip › 16S rRNA/Images/Clustering_Jaccard.pdf]

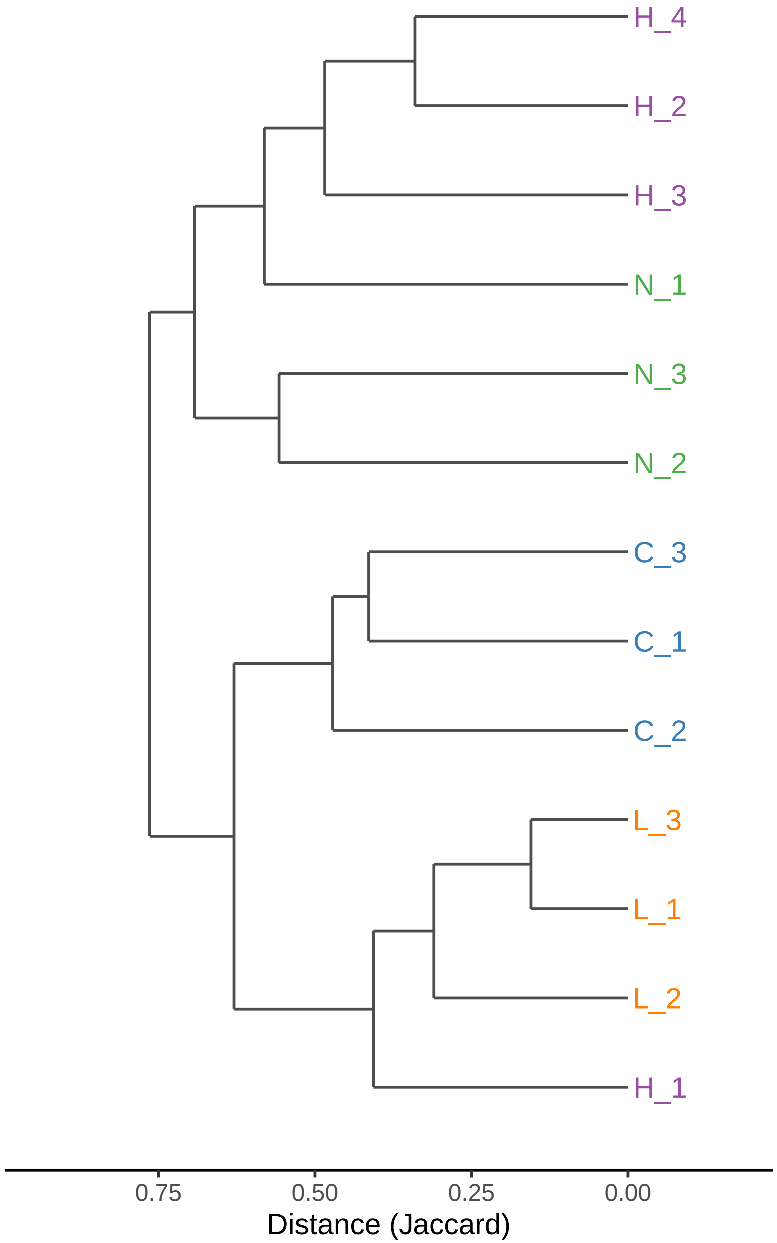
H_4
H_2
H_3
N_1
N_3
N_2
C_3
C_1
C_2
L_3
L_1
L_2
H_1
0.75
0.50
0.25
0.00
Distance (Jaccard)
