# Supplementary material for: Neuroprotective Effects of Oligosaccharides From Periplaneta Americana on Parkinson’s Disease Models In Vitro and In Vivo
Source: Front Pharmacol. 2022 Jul 18;13:936818. doi: 10.3389/fphar.2022.936818 (PMC9340460; doi:10.3389/fphar.2022.936818)
Supplement: Supplementary file 2 [file DataSheet1.zip › 16S rRNA/Images/Heatmap_Class_top50_clustering.pdf]

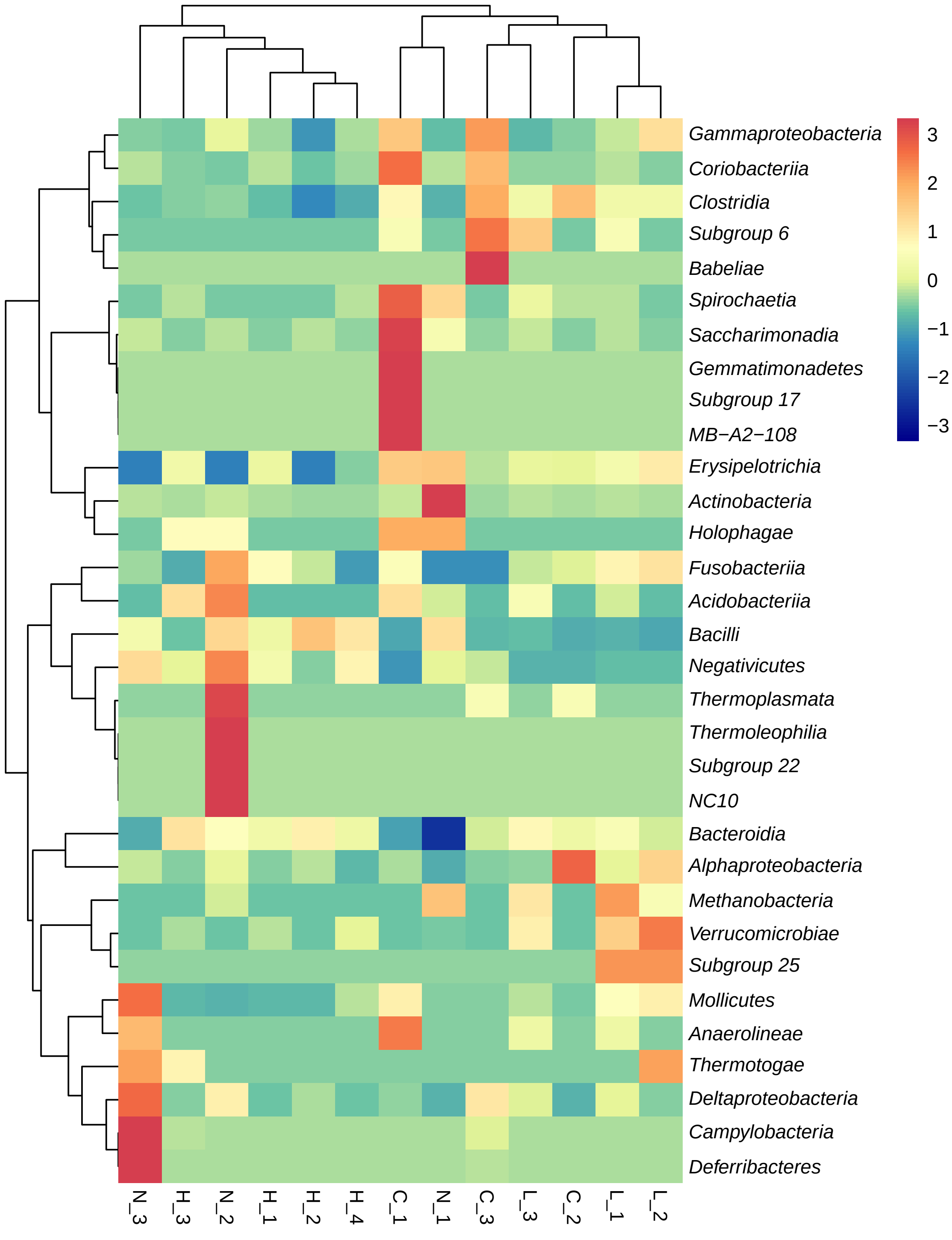
Gammaproteobacteria
Coriobacteriia
Clostridia
Subgroup 6
Babeliae
Spirochaetia
Saccharimonadia
Gemmatimonadetes
Subgroup 17
MB−A2−108
Erysipelotrichia
Actinobacteria
Holophagae
Fusobacteriia
Acidobacteriia
Bacilli
Negativicutes
Thermoplasmata
Thermoleophilia
Subgroup 22
NC10
Bacteroidia
Alphaproteobacteria
Methanobacteria
Verrucomicrobiae
Subgroup 25
Mollicutes
Anaerolineae
Thermotogae
Deltaproteobacteria
Campylobacteria
Deferribacteres
N_3
H_3
N_2
H_1
H_2
H_4
C_1
N_1
C_3
L_3
C_2
L_1
L_2
3
2
1
0
−1
−2
−3
