# Supplementary material for: Neuroprotective Effects of Oligosaccharides From Periplaneta Americana on Parkinson’s Disease Models In Vitro and In Vivo
Source: Front Pharmacol. 2022 Jul 18;13:936818. doi: 10.3389/fphar.2022.936818 (PMC9340460; doi:10.3389/fphar.2022.936818)
Supplement: Supplementary file 2 [file DataSheet1.zip › 16S rRNA/Images/Heatmap_Class_top50_mean_clustering.pdf]

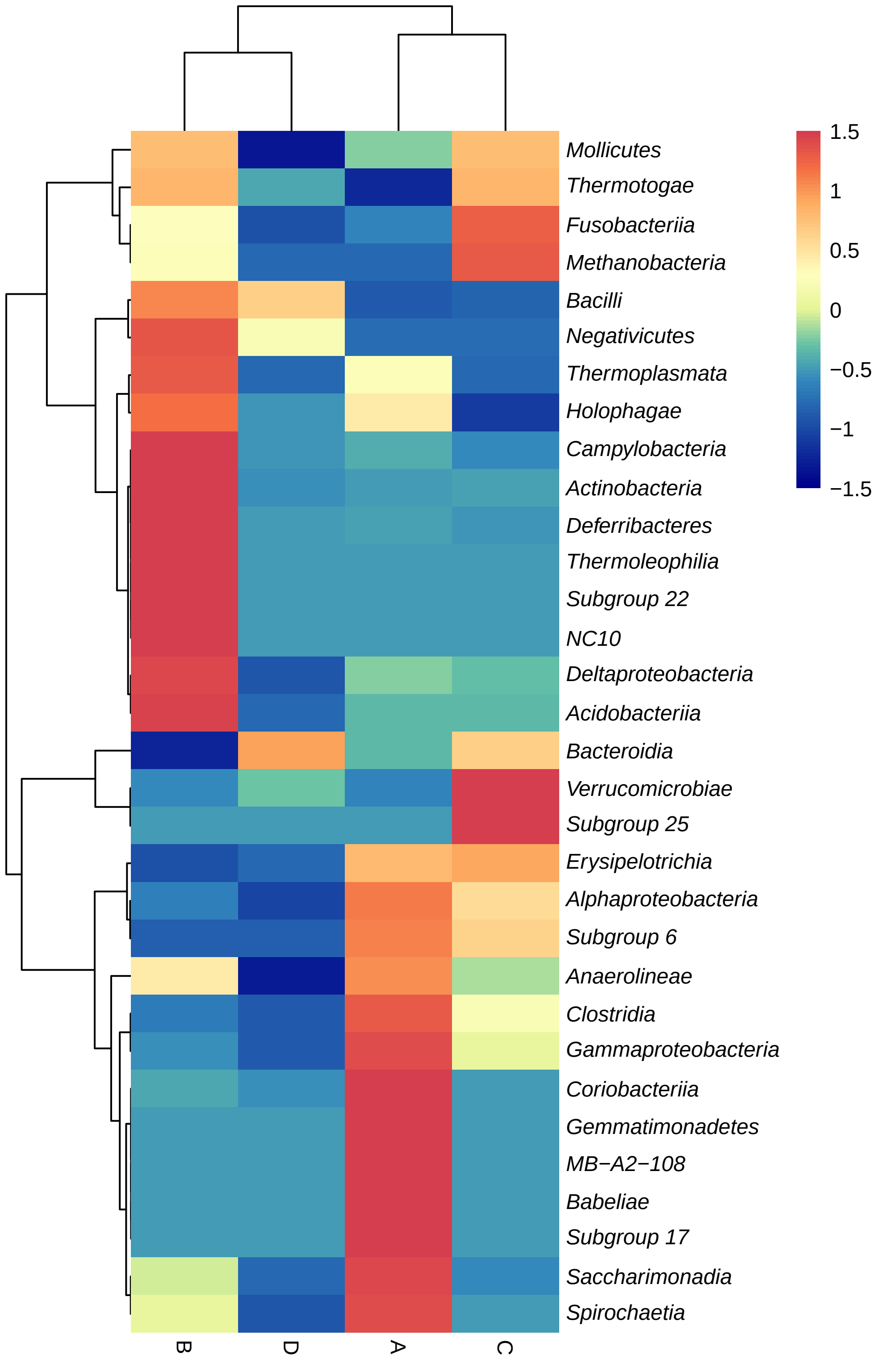

Mollicutes
Thermotogae
Fusobacteriia
Methanobacteria
Bacilli
Negativicutes
Thermoplasmata
Holophagae
Campylobacteria
Actinobacteria
Deferribacteres
Thermoleophilia
Subgroup 22
NC10
Deltaproteobacteria
Acidobacteriia
Bacteroidia
Verrucomicrobiae
Subgroup 25
Erysipelotrichia
Alphaproteobacteria
Subgroup 6
Anaerolineae
Clostridia
Gammaproteobacteria
Coriobacteriia
Gemmatimonadetes
MB−A2−108
Babeliae
Subgroup 17
Saccharimonadia
Spirochaetia
B
D
A
C
1.5
1
0.5
0
−0.5
−1
−1.5
