# Supplementary material for: Neuroprotective Effects of Oligosaccharides From Periplaneta Americana on Parkinson’s Disease Models In Vitro and In Vivo
Source: Front Pharmacol. 2022 Jul 18;13:936818. doi: 10.3389/fphar.2022.936818 (PMC9340460; doi:10.3389/fphar.2022.936818)
Supplement: Supplementary file 2 [file DataSheet1.zip › 16S rRNA/Images/Heatmap_Family_top50_clustering.pdf]

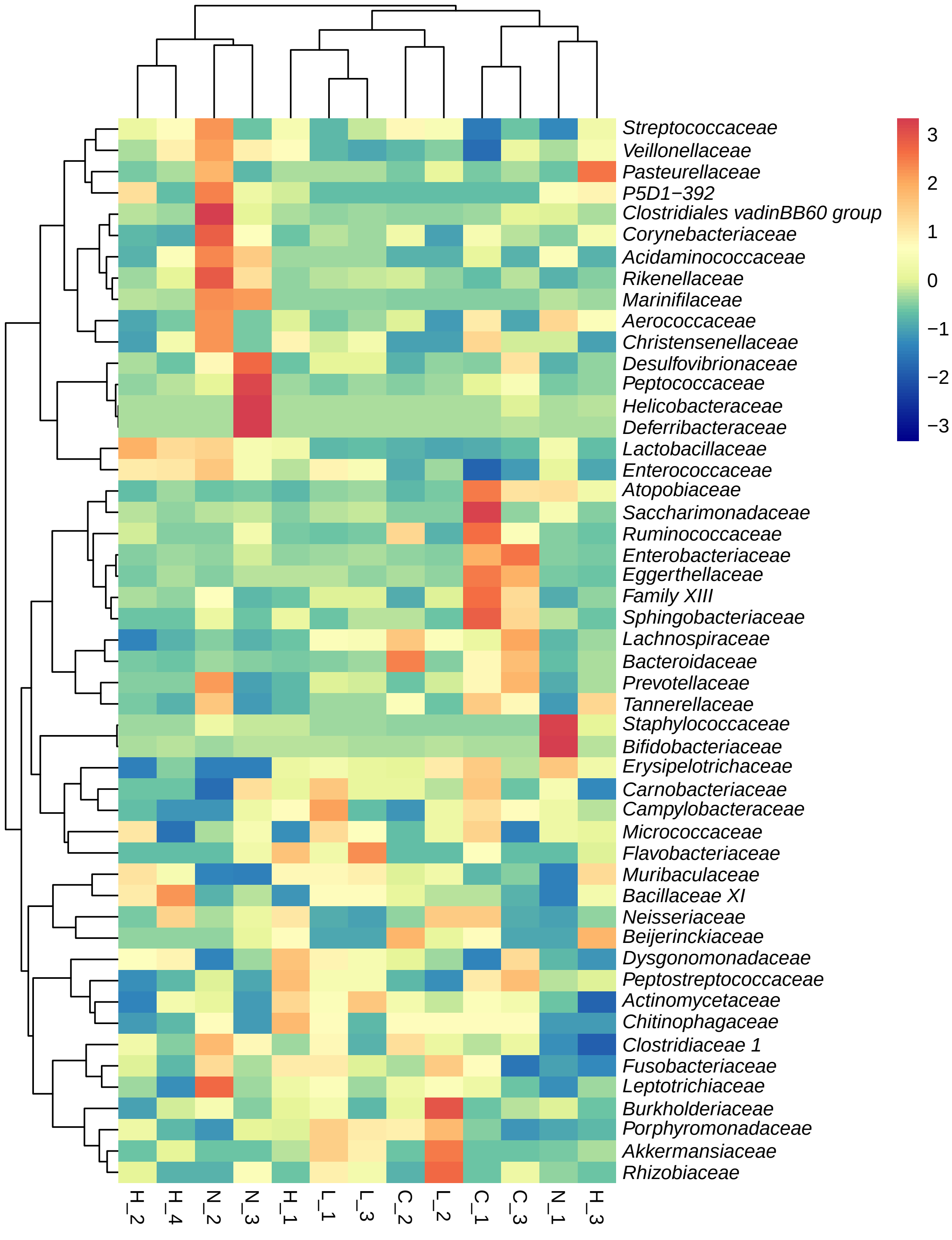
Streptococcaceae
Veillonellaceae
Pasteurellaceae
P5D1−392
Clostridiales vadinBB60 group
Corynebacteriaceae
Acidaminococcaceae
Rikenellaceae
Marinifilaceae
Aerococcaceae
Christensenellaceae
Desulfovibrionaceae
Peptococcaceae
Helicobacteraceae
Deferribacteraceae
Lactobacillaceae
Enterococcaceae
Atopobiaceae
Saccharimonadaceae
Ruminococcaceae
Enterobacteriaceae
Eggerthellaceae
Family XIII
Sphingobacteriaceae
Lachnospiraceae
Bacteroidaceae
Prevotellaceae
Tannerellaceae
Staphylococcaceae
Bifidobacteriaceae
Erysipelotrichaceae
Carnobacteriaceae
Campylobacteraceae
Micrococcaceae
Flavobacteriaceae
Muribaculaceae
Bacillaceae XI
Neisseriaceae
Beijerinckiaceae
Dysgonomonadaceae
Peptostreptococcaceae
Actinomycetaceae
Chitinophagaceae
Clostridiaceae 1
Fusobacteriaceae
Leptotrichiaceae
Burkholderiaceae
Porphyromonadaceae
Akkermansiaceae
Rhizobiaceae
H_2
H_4
N_2
N_3
H_1
L_1
L_3
C_2
L_2
C_1
C_3
N_1
H_3
3
2
1
0
−1
−2
−3
