# Supplementary material for: Neuroprotective Effects of Oligosaccharides From Periplaneta Americana on Parkinson’s Disease Models In Vitro and In Vivo
Source: Front Pharmacol. 2022 Jul 18;13:936818. doi: 10.3389/fphar.2022.936818 (PMC9340460; doi:10.3389/fphar.2022.936818)
Supplement: Supplementary file 2 [file DataSheet1.zip › 16S rRNA/Images/Heatmap_Family_top50_mean_clustering.pdf]

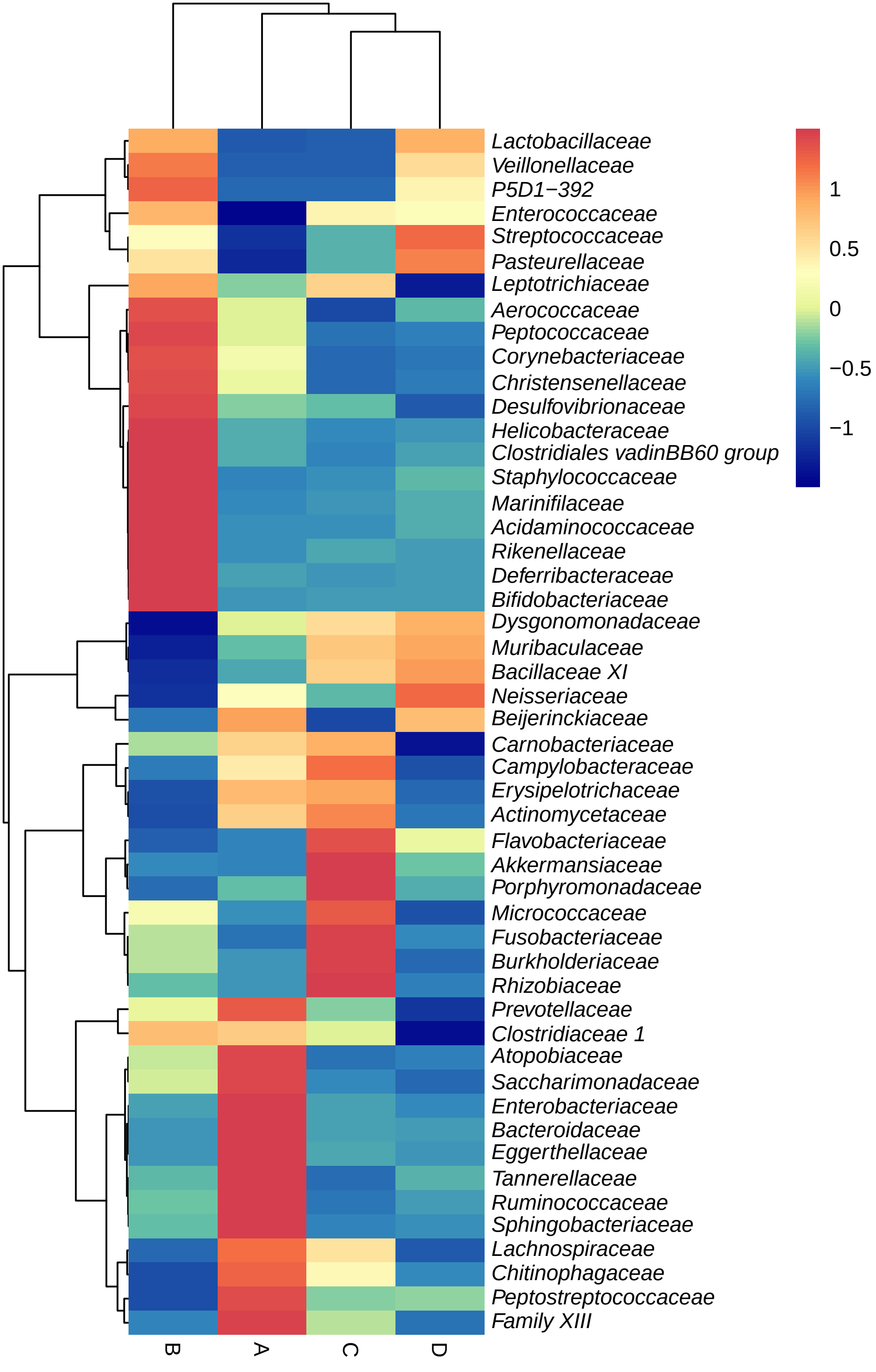
Lactobacillaceae
Veillonellaceae
P5D1−392
Enterococcaceae
Streptococcaceae
Pasteurellaceae
Leptotrichiaceae
Aerococcaceae
Peptococcaceae
Corynebacteriaceae
Christensenellaceae
Desulfovibrionaceae
Helicobacteraceae
Clostridiales vadinBB60 group
Staphylococcaceae
Marinifilaceae
Acidaminococcaceae
Rikenellaceae
Deferribacteraceae
Bifidobacteriaceae
Dysgonomonadaceae
Muribaculaceae
Bacillaceae XI
Neisseriaceae
Beijerinckiaceae
Carnobacteriaceae
Campylobacteraceae
Erysipelotrichaceae
Actinomycetaceae
Flavobacteriaceae
Akkermansiaceae
Porphyromonadaceae
Micrococcaceae
Fusobacteriaceae
Burkholderiaceae
Rhizobiaceae
Prevotellaceae
Clostridiaceae 1
Atopobiaceae
Saccharimonadaceae
Enterobacteriaceae
Bacteroidaceae
Eggerthellaceae
Tannerellaceae
Ruminococcaceae
Sphingobacteriaceae
Lachnospiraceae
Chitinophagaceae
Peptostreptococcaceae
Family XIII
B
A
C
D
1
0.5
0
−0.5
−1
