# Supplementary material for: Neuroprotective Effects of Oligosaccharides From Periplaneta Americana on Parkinson’s Disease Models In Vitro and In Vivo
Source: Front Pharmacol. 2022 Jul 18;13:936818. doi: 10.3389/fphar.2022.936818 (PMC9340460; doi:10.3389/fphar.2022.936818)
Supplement: Supplementary file 2 [file DataSheet1.zip › 16S rRNA/Images/Heatmap_Genus_top50_clustering.pdf]

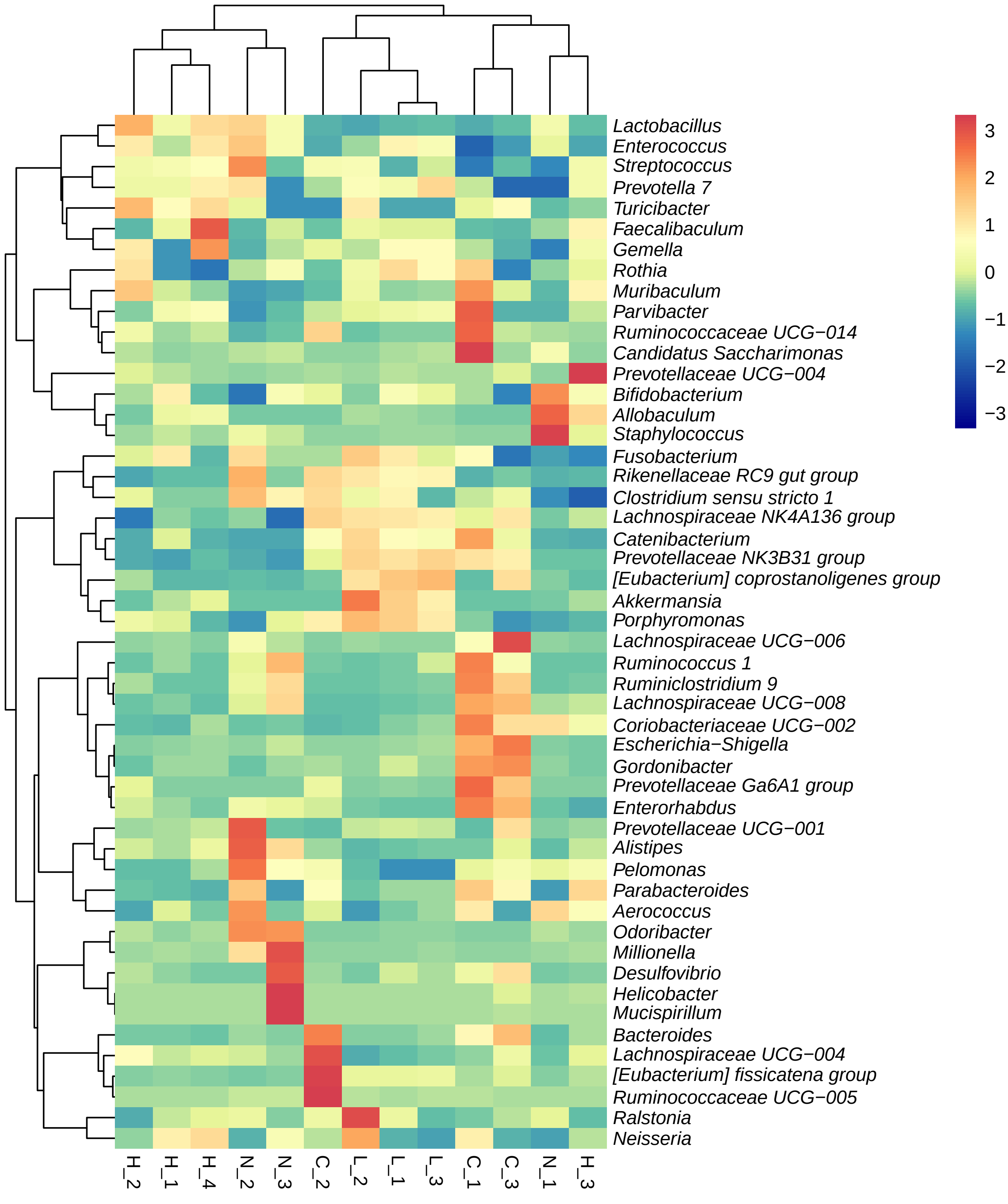

Lactobacillus
Enterococcus
Streptococcus
Prevotella 7
Turicibacter
Faecalibaculum
Gemella
Rothia
Muribaculum
Parvibacter
Ruminococcaceae UCG−014
Candidatus Saccharimonas
Prevotellaceae UCG−004
Bifidobacterium
Allobaculum
Staphylococcus
Fusobacterium
Rikenellaceae RC9 gut group
Clostridium sensu stricto 1
Lachnospiraceae NK4A136 group
Catenibacterium
Prevotellaceae NK3B31 group
[Eubacterium] coprostanoligenes group
Akkermansia
Porphyromonas
Lachnospiraceae UCG−006
Ruminococcus 1
Ruminiclostridium 9
Lachnospiraceae UCG−008
Coriobacteriaceae UCG−002
Escherichia−Shigella
Gordonibacter
Prevotellaceae Ga6A1 group
Enterorhabdus
Prevotellaceae UCG−001
Alistipes
Pelomonas
Parabacteroides
Aerococcus
Odoribacter
Millionella
Desulfovibrio
Helicobacter
Mucispirillum
Bacteroides
Lachnospiraceae UCG−004
[Eubacterium] fissicatena group
Ruminococcaceae UCG−005
Ralstonia
Neisseria
H_2
H_1
H_4
N_2
N_3
C_2
L_2
L_1
L_3
C_1
C_3
N_1
H_3
3
2
1
0
−1
−2
−3
