# Supplementary material for: Neuroprotective Effects of Oligosaccharides From Periplaneta Americana on Parkinson’s Disease Models In Vitro and In Vivo
Source: Front Pharmacol. 2022 Jul 18;13:936818. doi: 10.3389/fphar.2022.936818 (PMC9340460; doi:10.3389/fphar.2022.936818)
Supplement: Supplementary file 2 [file DataSheet1.zip › 16S rRNA/Images/Heatmap_Genus_top50_mean_clustering.pdf]

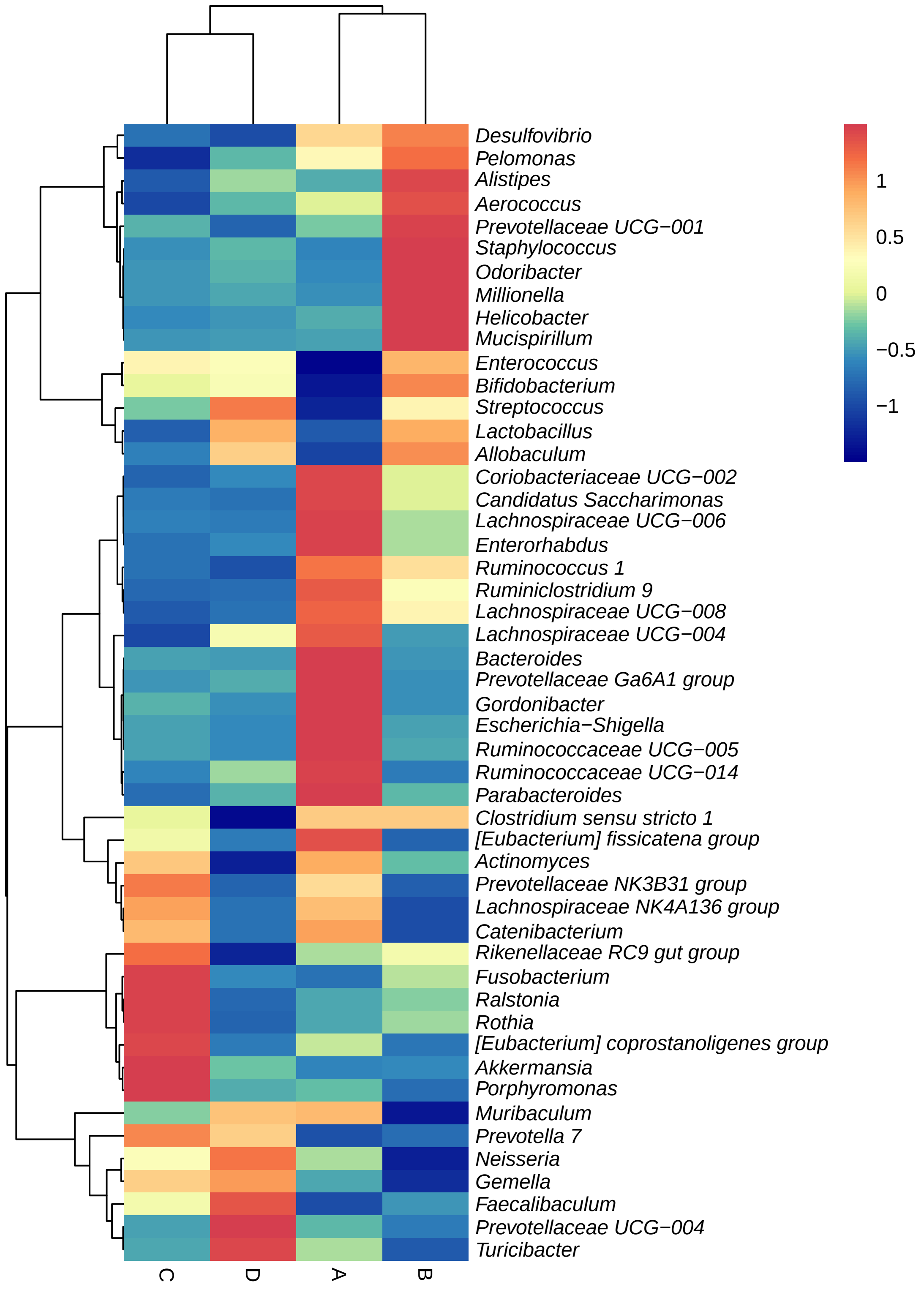
Desulfovibrio
Pelomonas
Alistipes
Aerococcus
Prevotellaceae UCG−001
Staphylococcus
Odoribacter
Millionella
Helicobacter
Mucispirillum
Enterococcus
Bifidobacterium
Streptococcus
Lactobacillus
Allobaculum
Coriobacteriaceae UCG−002
Candidatus Saccharimonas
Lachnospiraceae UCG−006
Enterorhabdus
Ruminococcus 1
Ruminiclostridium 9
Lachnospiraceae UCG−008
Lachnospiraceae UCG−004
Bacteroides
Prevotellaceae Ga6A1 group
Gordonibacter
Escherichia−Shigella
Ruminococcaceae UCG−005
Ruminococcaceae UCG−014
Parabacteroides
Clostridium sensu stricto 1
[Eubacterium] fissicatena group
Actinomyces
Prevotellaceae NK3B31 group
Lachnospiraceae NK4A136 group
Catenibacterium
Rikenellaceae RC9 gut group
Fusobacterium
Ralstonia
Rothia
[Eubacterium] coprostanoligenes group
Akkermansia
Porphyromonas
Muribaculum
Prevotella 7
Neisseria
Gemella
Faecalibaculum
Prevotellaceae UCG−004
Turicibacter
1
0.5
0
−0.5
−1
C
D
A
B
