# Supplementary material for: Neuroprotective Effects of Oligosaccharides From Periplaneta Americana on Parkinson’s Disease Models In Vitro and In Vivo
Source: Front Pharmacol. 2022 Jul 18;13:936818. doi: 10.3389/fphar.2022.936818 (PMC9340460; doi:10.3389/fphar.2022.936818)
Supplement: Supplementary file 2 [file DataSheet1.zip › 16S rRNA/Images/Heatmap_Order_top50_clustering.pdf]

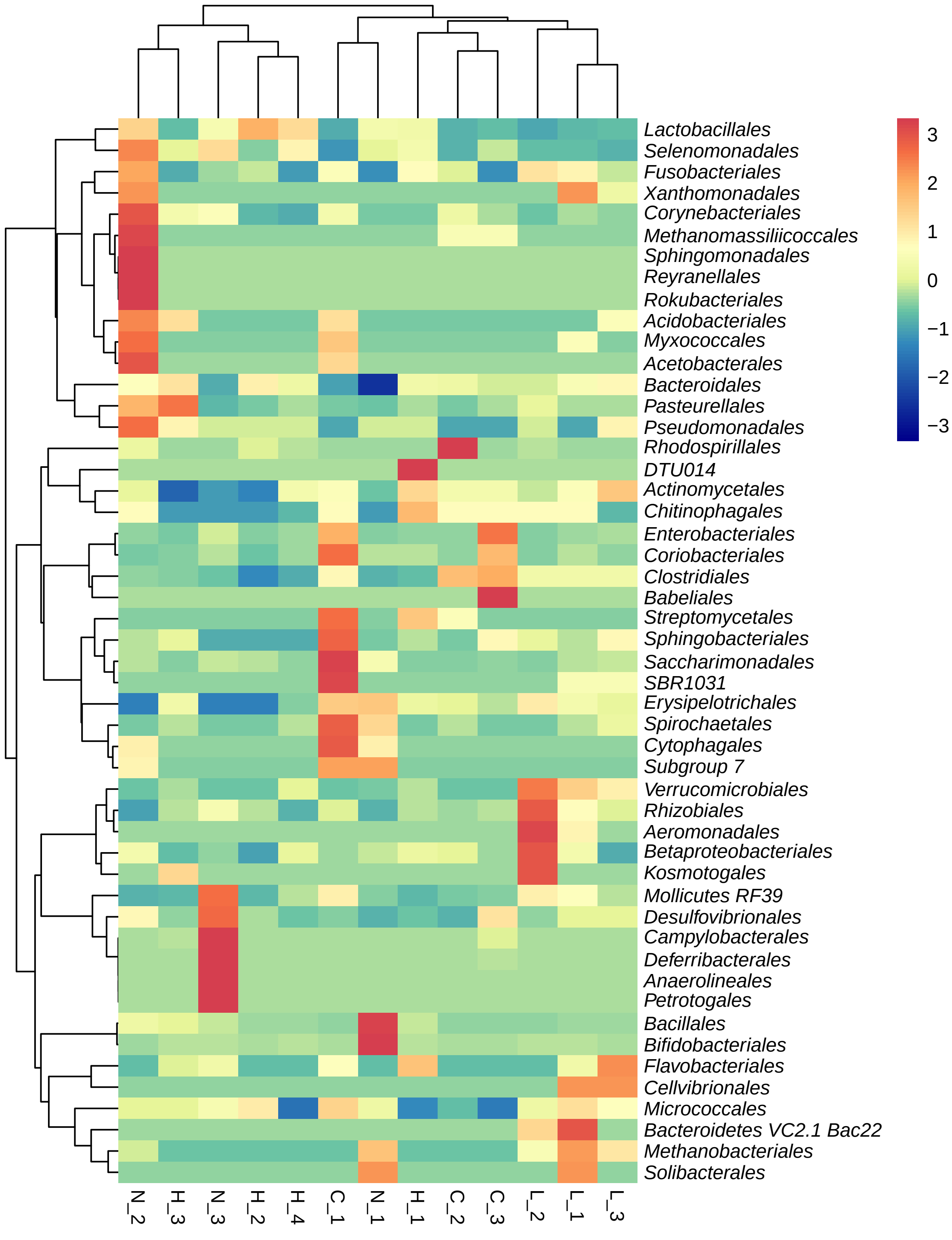

Lactobacillales
Selenomonadales
Fusobacteriales
Xanthomonadales
Corynebacteriales
Methanomassiliicoccales
Sphingomonadales
Reyranellales
Rokubacteriales
Acidobacteriales
Myxococcales
Acetobacterales
Bacteroidales
Pasteurellales
Pseudomonadales
Rhodospirillales
DTU014
Actinomycetales
Chitinophagales
Enterobacteriales
Coriobacteriales
Clostridiales
Babeliales
Streptomycetales
Sphingobacteriales
Saccharimonadales
SBR1031
Erysipelotrichales
Spirochaetales
Cytophagales
Subgroup 7
Verrucomicrobiales
Rhizobiales
Aeromonadales
Betaproteobacteriales
Kosmotogales
Mollicutes RF39
Desulfovibrionales
Campylobacterales
Deferribacterales
Anaerolineales
Petrotogales
Bacillales
Bifidobacteriales
Flavobacteriales
Cellvibrionales
Micrococcales
Bacteroidetes VC2.1 Bac22
Methanobacteriales
Solibacterales
N_2
H_3
N_3
H_2
H_4
C_1
N_1
H_1
C_2
C_3
L_2
L_1
L_3
3
2
1
0
−1
−2
−3
