# Supplementary material for: Neuroprotective Effects of Oligosaccharides From Periplaneta Americana on Parkinson’s Disease Models In Vitro and In Vivo
Source: Front Pharmacol. 2022 Jul 18;13:936818. doi: 10.3389/fphar.2022.936818 (PMC9340460; doi:10.3389/fphar.2022.936818)
Supplement: Supplementary file 2 [file DataSheet1.zip › 16S rRNA/Images/Heatmap_Order_top50_mean_clustering.pdf]

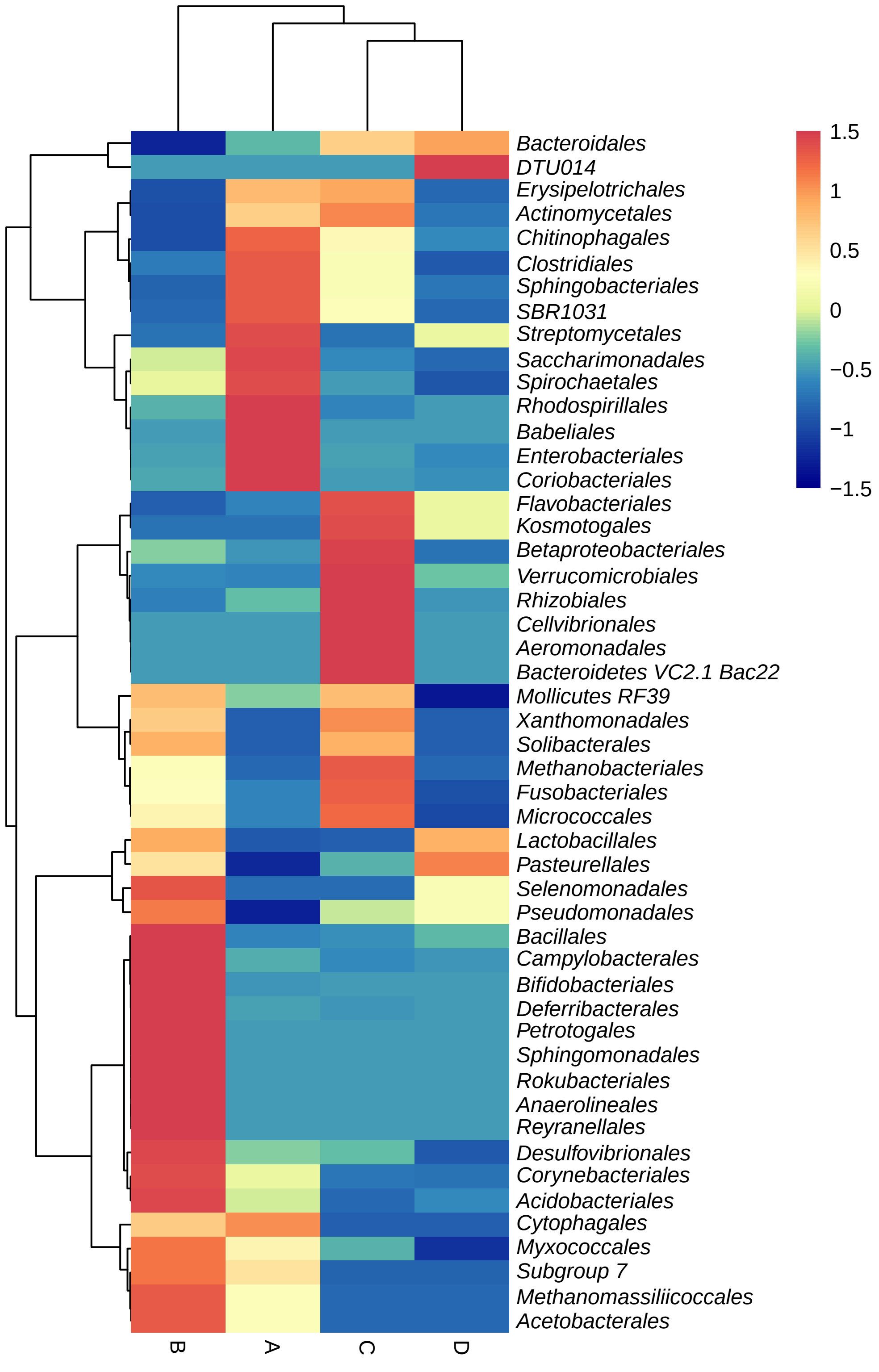

Bacteroidales
DTU014
Erysipelotrichales
Actinomycetales
Chitinophagales
Clostridiales
Sphingobacteriales
SBR1031
Streptomycetales
Saccharimonadales
Spirochaetales
Rhodospirillales
Babeliales
Enterobacteriales
Coriobacteriales
Flavobacteriales
Kosmotogales
Betaproteobacteriales
Verrucomicrobiales
Rhizobiales
Cellvibrionales
Aeromonadales
Bacteroidetes VC2.1 Bac22
Mollicutes RF39
Xanthomonadales
Solibacterales
Methanobacteriales
Fusobacteriales
Micrococcales
Lactobacillales
Pasteurellales
Selenomonadales
Pseudomonadales
Bacillales
Campylobacterales
Bifidobacteriales
Deferribacterales
Petrotogales
Sphingomonadales
Rokubacteriales
Anaerolineales
Reyranellales
Desulfovibrionales
Corynebacteriales
Acidobacteriales
Cytophagales
Myxococcales
Subgroup 7
Methanomassiliicoccales
Acetobacterales
B
A
C
D
1.5
1
0.5
0
−0.5
−1
−1.5
