# Supplementary material for: Neuroprotective Effects of Oligosaccharides From Periplaneta Americana on Parkinson’s Disease Models In Vitro and In Vivo
Source: Front Pharmacol. 2022 Jul 18;13:936818. doi: 10.3389/fphar.2022.936818 (PMC9340460; doi:10.3389/fphar.2022.936818)
Supplement: Supplementary file 2 [file DataSheet1.zip › 16S rRNA/Images/Heatmap_Phylum_top50_clustering.pdf]

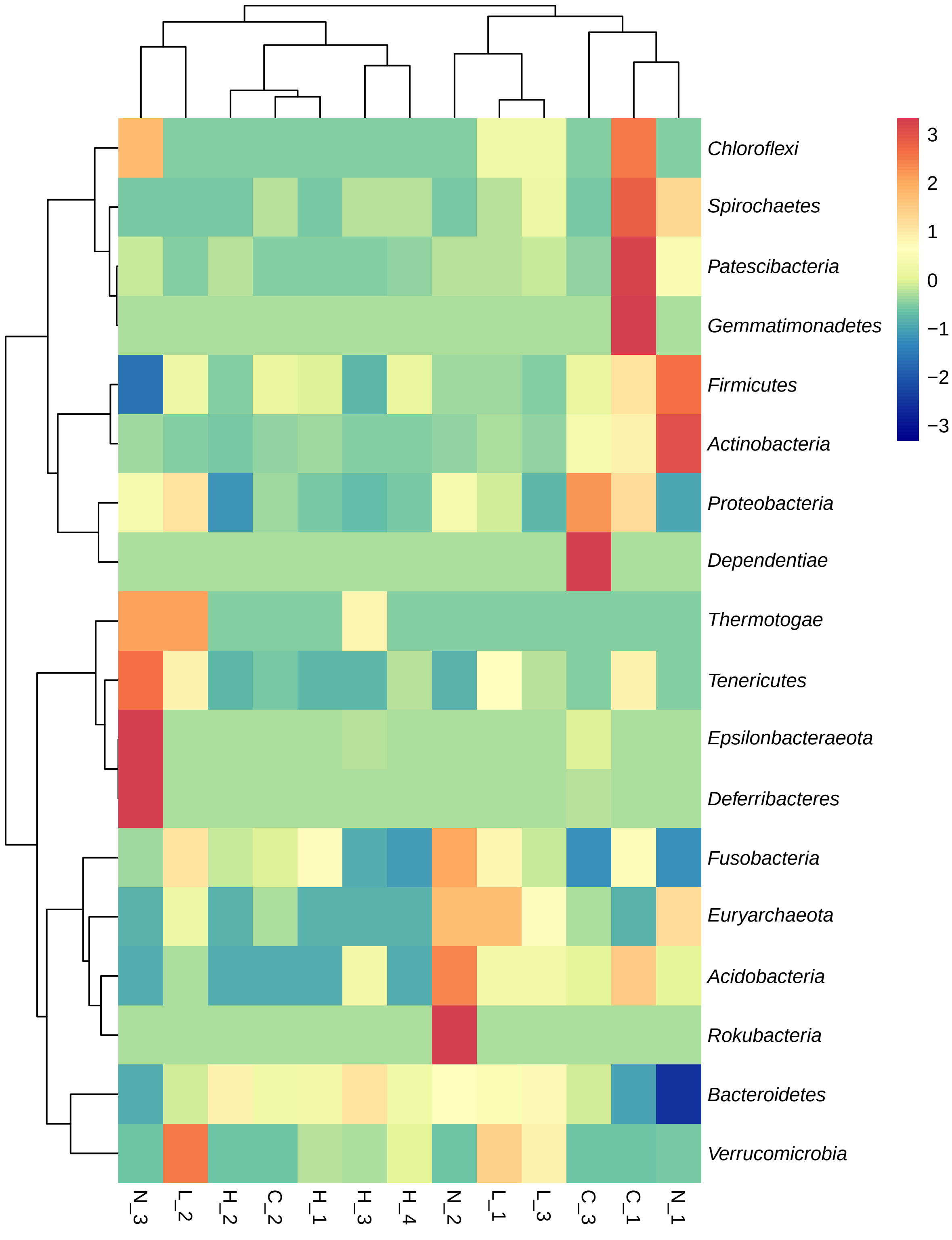

3
2
1
0
−1
−2
−3
Chloroflexi
Spirochaetes
Patescibacteria
Gemmatimonadetes
Firmicutes
Actinobacteria
Proteobacteria
Dependentiae
Thermotogae
Tenericutes
Epsilonbacteraeota
Deferribacteres
Fusobacteria
Euryarchaeota
Acidobacteria
Rokubacteria
Bacteroidetes
Verrucomicrobia
N_3
L_2
H_2
C_2
H_1
H_3
H_4
N_2
L_1
L_3
C_3
C_1
N_1
