# Supplementary material for: Neuroprotective Effects of Oligosaccharides From Periplaneta Americana on Parkinson’s Disease Models In Vitro and In Vivo
Source: Front Pharmacol. 2022 Jul 18;13:936818. doi: 10.3389/fphar.2022.936818 (PMC9340460; doi:10.3389/fphar.2022.936818)
Supplement: Supplementary file 2 [file DataSheet1.zip › 16S rRNA/Images/Heatmap_Phylum_top50_mean_clustering.pdf]

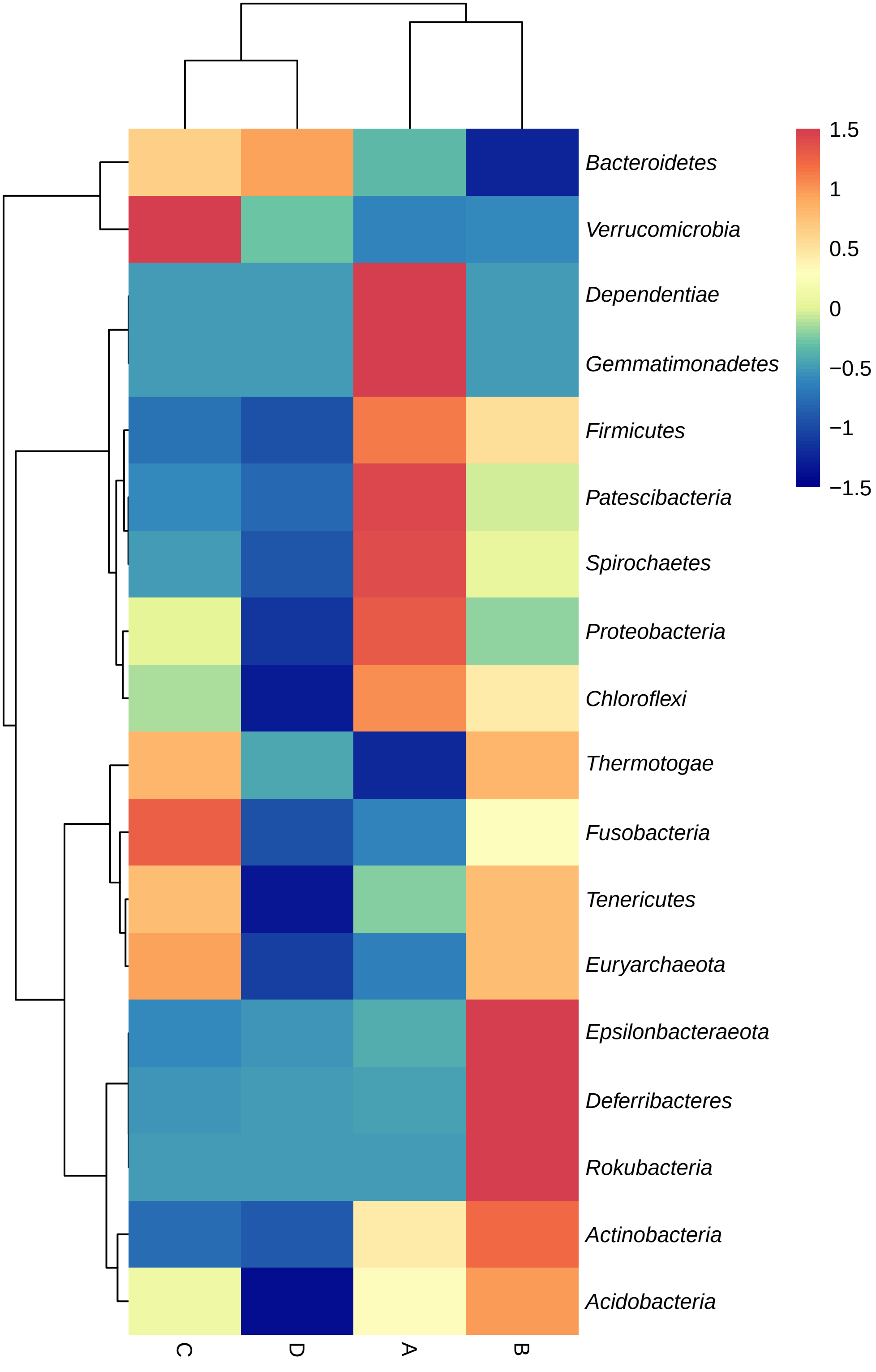

Bacteroidetes
Verrucomicrobia
Dependentiae
Gemmatimonadetes
Firmicutes
Patescibacteria
Spirochaetes
Proteobacteria
Chloroflexi
Thermotogae
Fusobacteria
Tenericutes
Euryarchaeota
Epsilonbacteraeota
Deferribacteres
Rokubacteria
Actinobacteria
Acidobacteria
C
D
A
B
1.5
1
0.5
0
−0.5
−1
−1.5
