# Supplementary material for: Neuroprotective Effects of Oligosaccharides From Periplaneta Americana on Parkinson’s Disease Models In Vitro and In Vivo
Source: Front Pharmacol. 2022 Jul 18;13:936818. doi: 10.3389/fphar.2022.936818 (PMC9340460; doi:10.3389/fphar.2022.936818)
Supplement: Supplementary file 2 [file DataSheet1.zip › 16S rRNA/Images/Heatmap_Species_top50_clustering.pdf]

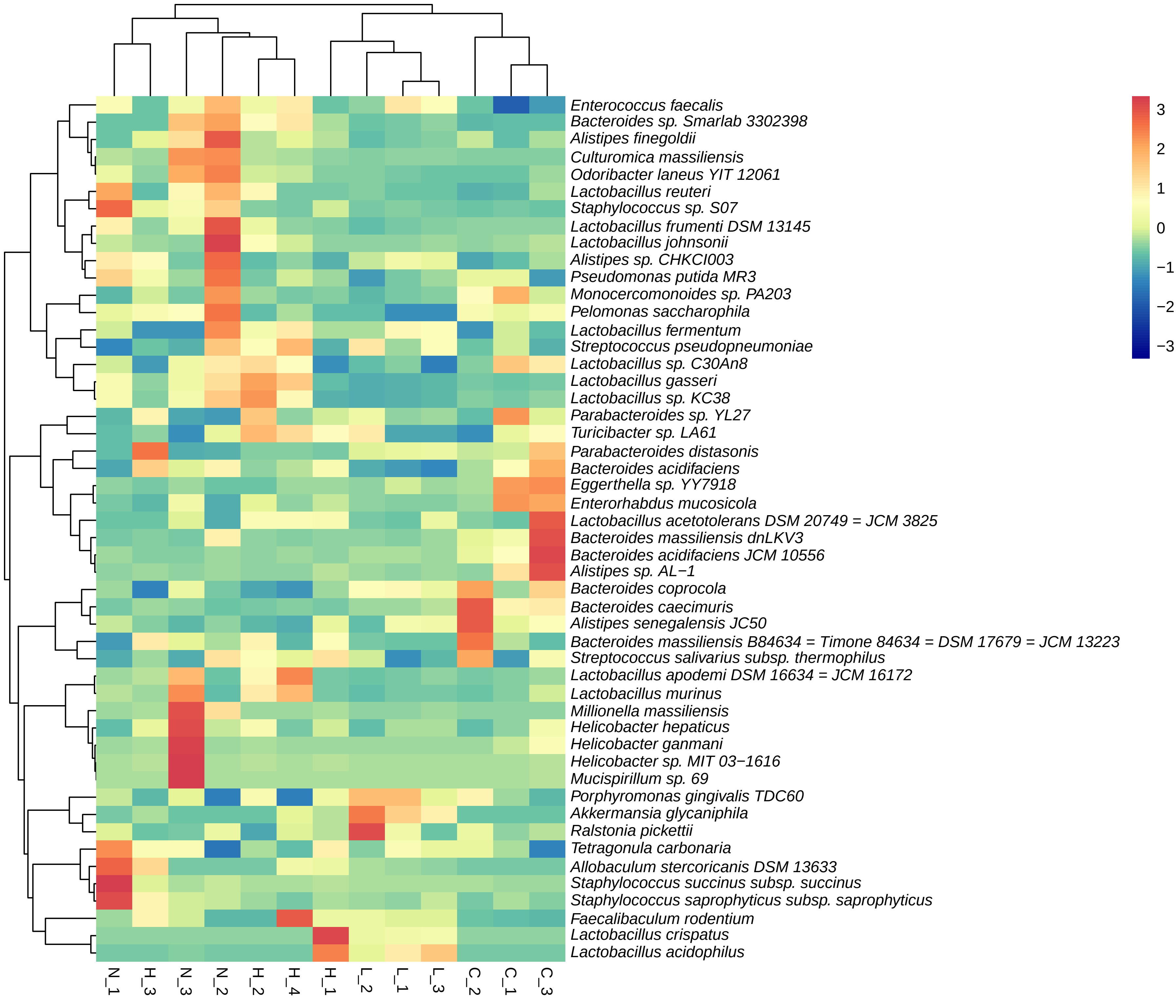
Enterococcus faecalis
Bacteroides sp. Smarlab 3302398
Alistipes finegoldii
Culturomica massiliensis
Odoribacter laneus YIT 12061
Lactobacillus reuteri
Staphylococcus sp. S07
Lactobacillus frumenti DSM 13145
Lactobacillus johnsonii
Alistipes sp. CHKCI003
Pseudomonas putida MR3
Monocercomonoides sp. PA203
Pelomonas saccharophila
Lactobacillus fermentum
Streptococcus pseudopneumoniae
Lactobacillus sp. C30An8
Lactobacillus gasseri
Lactobacillus sp. KC38
Parabacteroides sp. YL27
Turicibacter sp. LA61
Parabacteroides distasonis
Bacteroides acidifaciens
Eggerthella sp. YY7918
Enterorhabdus mucosicola
Lactobacillus acetotolerans DSM 20749 = JCM 3825
Bacteroides massiliensis dnLKV3
Bacteroides acidifaciens JCM 10556
Alistipes sp. AL−1
Bacteroides coprocola
Bacteroides caecimuris
Alistipes senegalensis JC50
Bacteroides massiliensis B84634 = Timone 84634 = DSM 17679 = JCM 13223
Streptococcus salivarius subsp. thermophilus
Lactobacillus apodemi DSM 16634 = JCM 16172
Lactobacillus murinus
Millionella massiliensis
Helicobacter hepaticus
Helicobacter ganmani
Helicobacter sp. MIT 03−1616
Mucispirillum sp. 69
Porphyromonas gingivalis TDC60
Akkermansia glycaniphila
Ralstonia pickettii
Tetragonula carbonaria
Allobaculum stercoricans DSM 13633
Staphylococcus succinus subsp. succinus
Staphylococcus saprophyticus subsp. saprophyticus
Faecalibaculum rodentium
Lactobacillus crispatus
Lactobacillus acidophilus
N_1
H_3
N_3
N_2
H_2
H_4
H_1
L_2
L_1
L_3
C_2
C_1
C_3
3
2
1
0
−1
−2
−3
