# Supplementary material for: Neuroprotective Effects of Oligosaccharides From Periplaneta Americana on Parkinson’s Disease Models In Vitro and In Vivo
Source: Front Pharmacol. 2022 Jul 18;13:936818. doi: 10.3389/fphar.2022.936818 (PMC9340460; doi:10.3389/fphar.2022.936818)
Supplement: Supplementary file 2 [file DataSheet1.zip › 16S rRNA/Images/Heatmap_Species_top50_mean_clustering.pdf]

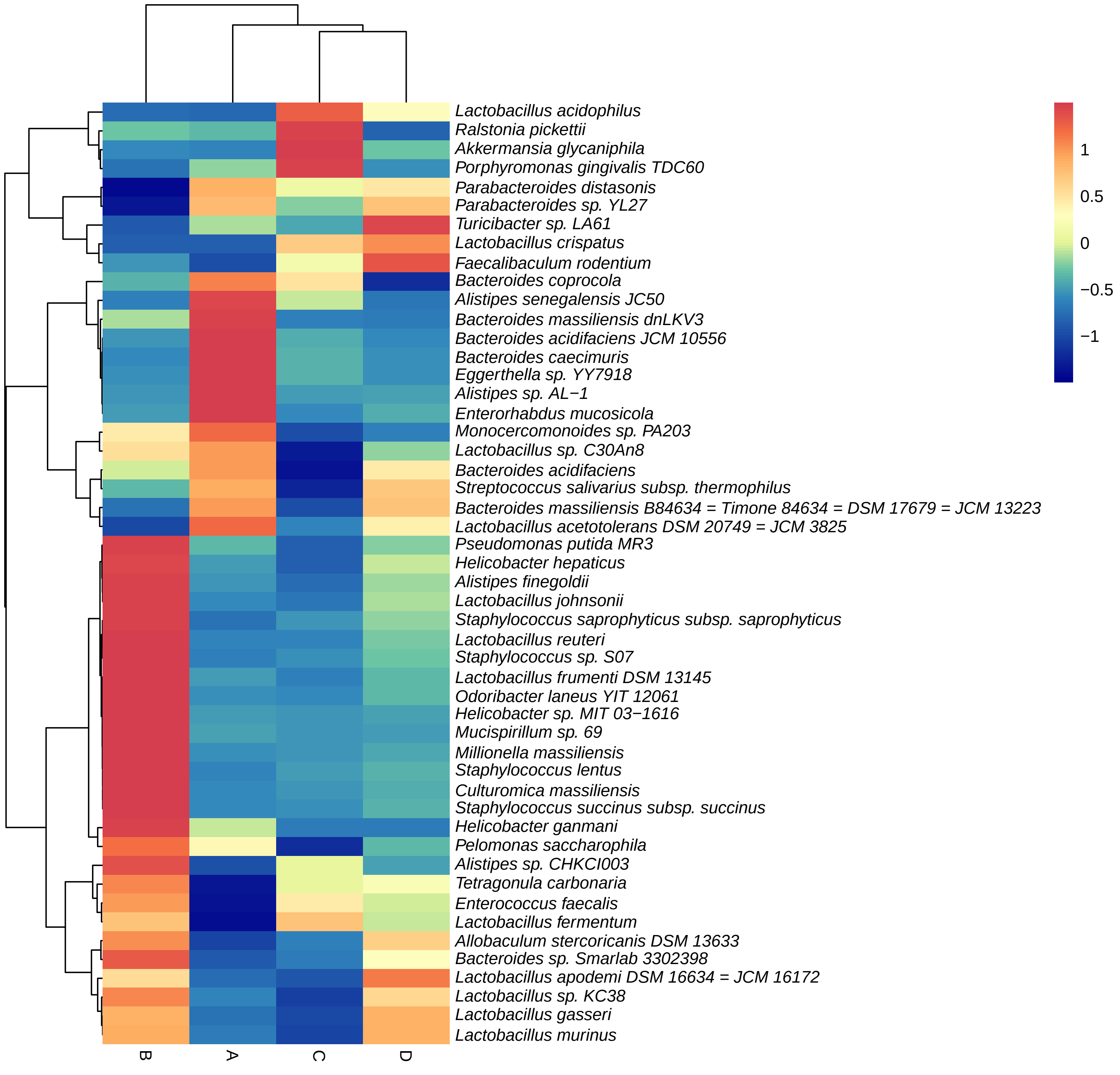

Lactobacillus acidophilus
Ralstonia pickettii
Akkermansia glycaniphila
Porphyromonas gingivalis TDC60
Parabacteroides distasonis
Parabacteroides sp. YL27
Turicibacter sp. LA61
Lactobacillus crispatus
Faecalibaculum rodentium
Bacteroides coprocola
Alistipes senegalensis JC50
Bacteroides massiliensis dnLKV3
Bacteroides acidifaciens JCM 10556
Bacteroides caecimuris
Eggerthella sp. YY7918
Alistipes sp. AL−1
Enterorhabdus mucosicola
Monocercomonoides sp. PA203
Lactobacillus sp. C30An8
Bacteroides acidifaciens
Streptococcus salivarius subsp. thermophilus
Bacteroides massiliensis B84634 = Timone 84634 = DSM 17679 = JCM 13223
Lactobacillus acetotolerans DSM 20749 = JCM 3825
Pseudomonas putida MR3
Helicobacter hepaticus
Alistipes finegoldii
Lactobacillus johnsonii
Staphylococcus saprophyticus subsp. saprophyticus
Lactobacillus reuteri
Staphylococcus sp. S07
Lactobacillus frumenti DSM 13145
Odoribacter laneus YIT 12061
Helicobacter sp. MIT 03−1616
Mucispirillum sp. 69
Millionella massiliensis
Staphylococcus lentus
Culturomica massiliensis
Staphylococcus succinus subsp. succinus
Helicobacter ganmani
Pelomonas saccharophila
Alistipes sp. CHKCI003
Tetragonula carbonaria
Enterococcus faecalis
Lactobacillus fermentum
Allobaculum stercoricanis DSM 13633
Bacteroides sp. Smarlab 3302398
Lactobacillus apodemi DSM 16634 = JCM 16172
Lactobacillus sp. KC38
Lactobacillus gasseri
Lactobacillus murinus
B
A
C
D
1
0.5
0
−0.5
−1
