# Supplementary material for: Neuroprotective Effects of Oligosaccharides From Periplaneta Americana on Parkinson’s Disease Models In Vitro and In Vivo
Source: Front Pharmacol. 2022 Jul 18;13:936818. doi: 10.3389/fphar.2022.936818 (PMC9340460; doi:10.3389/fphar.2022.936818)
Supplement: Supplementary file 2 [file DataSheet1.zip › 16S rRNA/Images/Heatmap_class_top50_all.pdf]

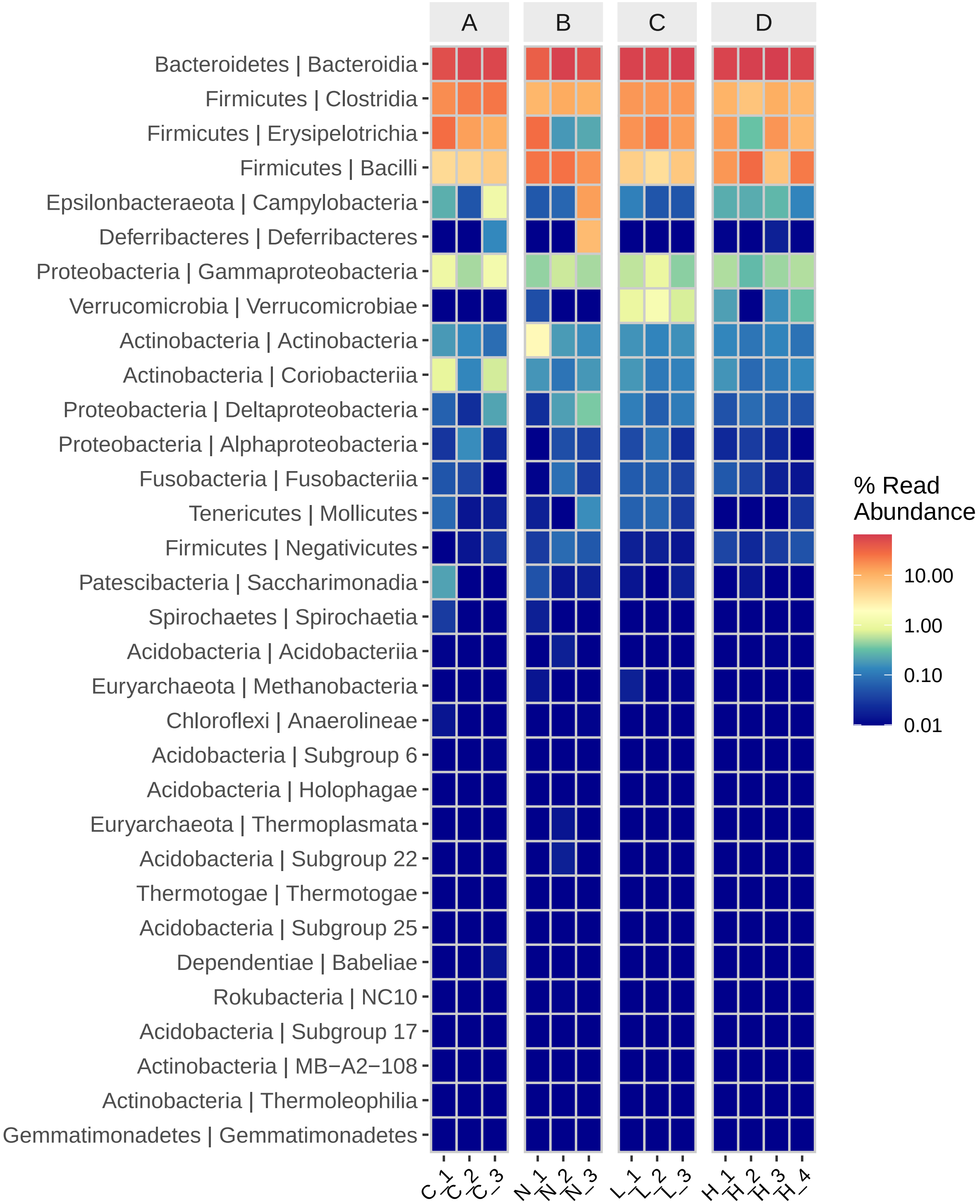

A
B
C
D
Bacteroidetes | Bacteroidia
Firmicutes | Clostridia
Firmicutes | Erysipelotrichia
Firmicutes | Bacilli
Epsilonbacteraeota | Campylobacteria
Deferribacteres | Deferribacteres
Proteobacteria | Gammaproteobacteria
Verrucomicrobia | Verrucomicrobiae
Actinobacteria | Actinobacteria
Actinobacteria | Coriobacteriia
Proteobacteria | Deltaproteobacteria
Proteobacteria | Alphaproteobacteria
Fusobacteria | Fusobacteriia
Tenericutes | Mollicutes
Firmicutes | Negativicutes
Patescibacteria | Saccharimonadia
Spirochaetes | Spirochaetia
Acidobacteria | Acidobacteriia
Euryarchaeota | Methanobacteria
Chloroflexi | Anaerolineae
Acidobacteria | Subgroup 6
Acidobacteria | Holophagae
Euryarchaeota | Thermoplasmata
Acidobacteria | Subgroup 22
Thermotogae | Thermotogae
Acidobacteria | Subgroup 25
Dependentiae | Babeliae
Rokubacteria | NC10
Acidobacteria | Subgroup 17
Actinobacteria | MB−A2−108
Actinobacteria | Thermoleophilia
Gemmatimonadetes | Gemmatimonadetes
C_1
C_2
C_3
N_1
N_2
N_3
L_1
L_2
L_3
H_1
H_2
H_3
H_4
% Read Abundance
10.00
1.00
0.10
0.01
