# Supplementary material for: Neuroprotective Effects of Oligosaccharides From Periplaneta Americana on Parkinson’s Disease Models In Vitro and In Vivo
Source: Front Pharmacol. 2022 Jul 18;13:936818. doi: 10.3389/fphar.2022.936818 (PMC9340460; doi:10.3389/fphar.2022.936818)
Supplement: Supplementary file 2 [file DataSheet1.zip › 16S rRNA/Images/Heatmap_family_top50_mean.pdf]

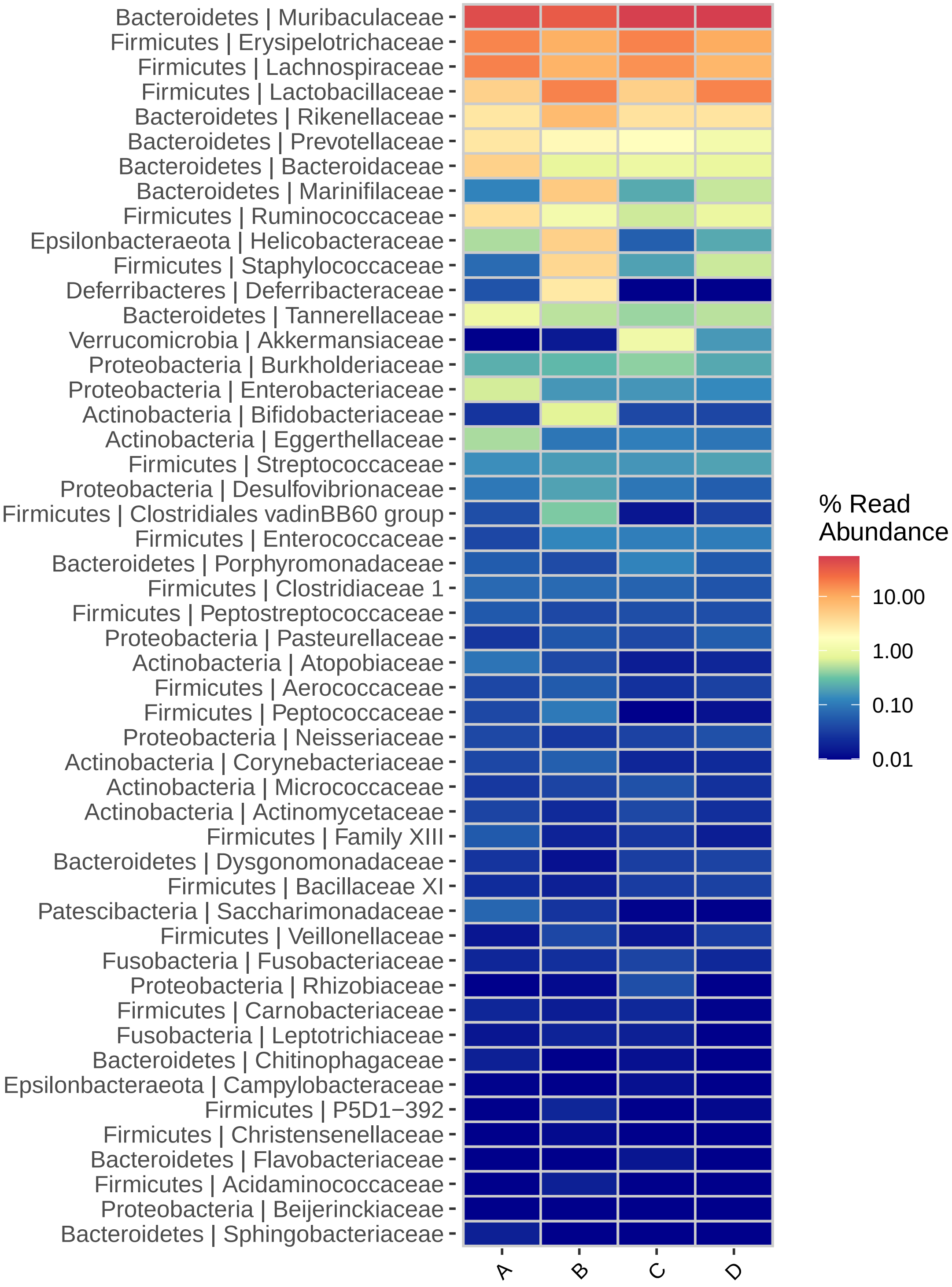
Bacteroidetes | Muribaculaceae
Firmicutes | Erysipelotrichaceae
Firmicutes | Lachnospiraceae
Firmicutes | Lactobacillaceae
Bacteroidetes | Rikenellaceae
Bacteroidetes | Prevotellaceae
Bacteroidetes | Bacteroidaceae
Bacteroidetes | Marinifilaceae
Firmicutes | Ruminococcaceae
Epsilonbacteraeota | Helicobacteraceae
Firmicutes | Staphylococcaceae
Deferribacteres | Deferribacteraceae
Bacteroidetes | Tannerellaceae
Verrucomicrobia | Akkermansiaceae
Proteobacteria | Burkholderiaceae
Proteobacteria | Enterobacteriaceae
Actinobacteria | Bifidobacteriaceae
Actinobacteria | Eggerthellaceae
Firmicutes | Streptococcaceae
Proteobacteria | Desulfovibrionaceae
Firmicutes | Clostridiales vadinBB60 group
Firmicutes | Enterococcaceae
Bacteroidetes | Porphyromonadaceae
Firmicutes | Clostridiaceae 1
Firmicutes | Peptostreptococcaceae
Proteobacteria | Pasteurellaceae
Actinobacteria | Atopobiaceae
Firmicutes | Aerococcaceae
Firmicutes | Peptococcaceae
Proteobacteria | Neisseriaceae
Actinobacteria | Corynebacteriaceae
Actinobacteria | Micrococcaceae
Actinobacteria | Actinomycetaceae
Firmicutes | Family XIII
Bacteroidetes | Dysgonomonadaceae
Firmicutes | Bacillaceae XI
Patescibacteria | Saccharimonadaceae
Firmicutes | Veillonellaceae
Fusobacteria | Fusobacteriaceae
Proteobacteria | Rhizobiaceae
Firmicutes | Carnobacteriaceae
Fusobacteria | Leptotrichiaceae
Bacteroidetes | Chitinophagaceae
Epsilonbacteraeota | Campylobacteraceae
Firmicutes | P5D1−392
Firmicutes | Christensenellaceae
Bacteroidetes | Flavobacteriaceae
Firmicutes | Acidaminococcaceae
Proteobacteria | Beijerinckiaceae
Bacteroidetes | Sphingobacteriaceae
A
B
C
D
% Read Abundance
10.00
1.00
0.10
0.01
