# Supplementary material for: Neuroprotective Effects of Oligosaccharides From Periplaneta Americana on Parkinson’s Disease Models In Vitro and In Vivo
Source: Front Pharmacol. 2022 Jul 18;13:936818. doi: 10.3389/fphar.2022.936818 (PMC9340460; doi:10.3389/fphar.2022.936818)
Supplement: Supplementary file 2 [file DataSheet1.zip › 16S rRNA/Images/Heatmap_genus_top50_all.pdf]

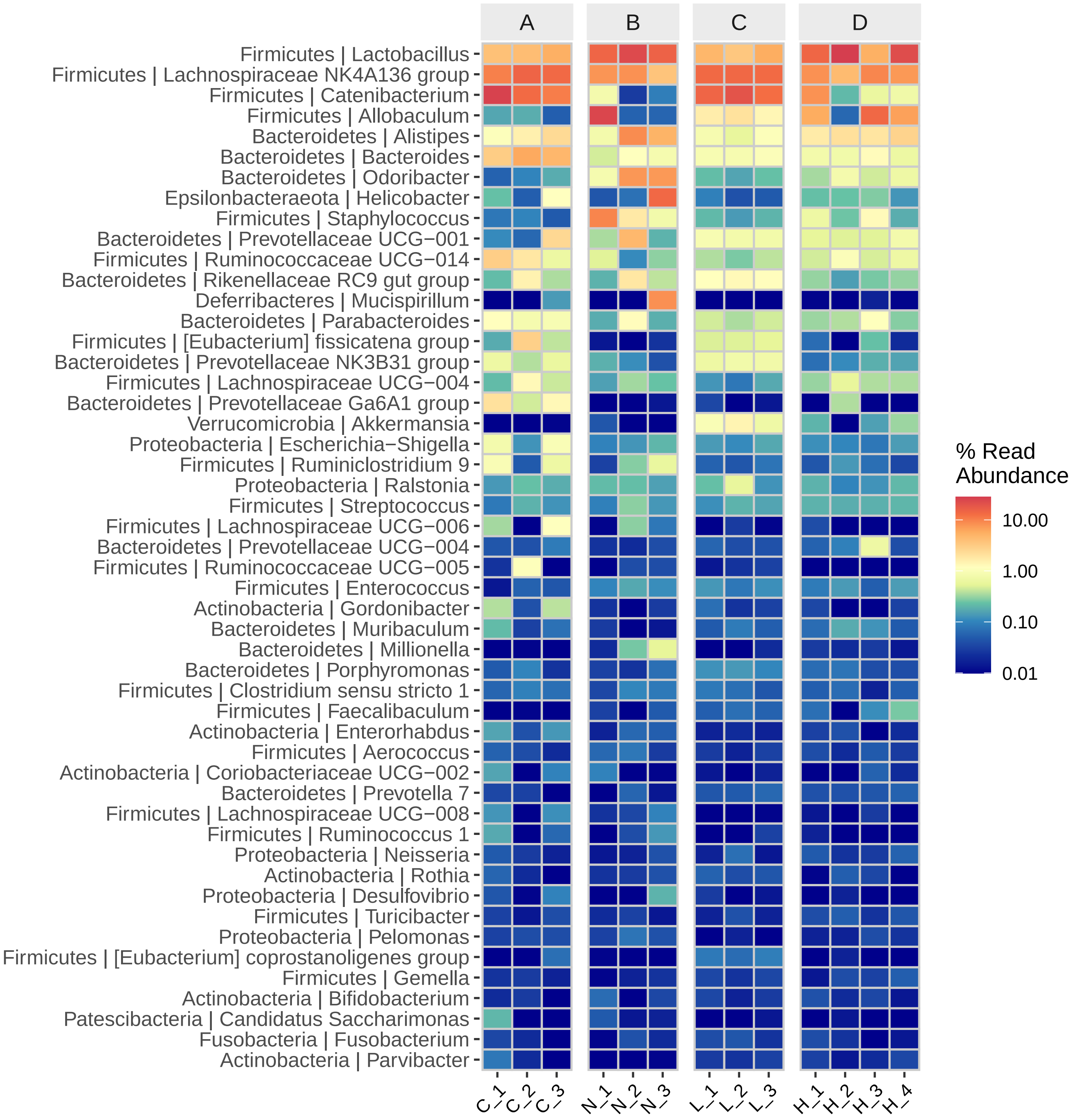

A
B
C
D
Firmicutes | Lactobacillus
Firmicutes | Lachnospiraceae NK4A136 group
Firmicutes | Catenibacterium
Firmicutes | Allobaculum
Bacteroidetes | Alistipes
Bacteroidetes | Bacteroides
Bacteroidetes | Odoribacter
Epsilonbacteraeota | Helicobacter
Firmicutes | Staphylococcus
Bacteroidetes | Prevotellaceae UCG−001
Firmicutes | Ruminococcaceae UCG−014
Bacteroidetes | Rikenellaceae RC9 gut group
Deferribacteres | Mucispirillum
Bacteroidetes | Parabacteroides
Firmicutes | [Eubacterium] fissicatena group
Bacteroidetes | Prevotellaceae NK3B31 group
Firmicutes | Lachnospiraceae UCG−004
Bacteroidetes | Prevotellaceae Ga6A1 group
Verrucomicrobia | Akkermansia
Proteobacteria | Escherichia−Shigella
Firmicutes | Ruminiclostridium 9
Proteobacteria | Ralstonia
Firmicutes | Streptococcus
Firmicutes | Lachnospiraceae UCG−006
Bacteroidetes | Prevotellaceae UCG−004
Firmicutes | Ruminococcaceae UCG−005
Firmicutes | Enterococcus
Actinobacteria | Gordonibacter
Bacteroidetes | Muribaculum
Bacteroidetes | Millionella
Bacteroidetes | Porphyromonas
Firmicutes | Clostridium sensu stricto 1
Firmicutes | Faecalibaculum
Actinobacteria | Enterorhabdus
Firmicutes | Aerococcus
Actinobacteria | Coriobacteriaceae UCG−002
Bacteroidetes | Prevotella 7
Firmicutes | Lachnospiraceae UCG−008
Firmicutes | Ruminococcus 1
Proteobacteria | Neisseria
Actinobacteria | Rothia
Proteobacteria | Desulfovibrio
Firmicutes | Turicibacter
Proteobacteria | Pelomonas
Firmicutes | [Eubacterium] coprostanoligenes group
Firmicutes | Gemella
Actinobacteria | Bifidobacterium
Patescibacteria | Candidatus Saccharimonas
Fusobacteria | Fusobacterium
Actinobacteria | Parvibacter
C_1
C_2
C_3
N_1
N_2
N_3
L_1
L_2
L_3
H_1
H_2
H_3
H_4
% Read Abundance
10.00
1.00
0.10
0.01
