# Supplementary material for: Neuroprotective Effects of Oligosaccharides From Periplaneta Americana on Parkinson’s Disease Models In Vitro and In Vivo
Source: Front Pharmacol. 2022 Jul 18;13:936818. doi: 10.3389/fphar.2022.936818 (PMC9340460; doi:10.3389/fphar.2022.936818)
Supplement: Supplementary file 2 [file DataSheet1.zip › 16S rRNA/Images/Heatmap_order_top50_mean.pdf]

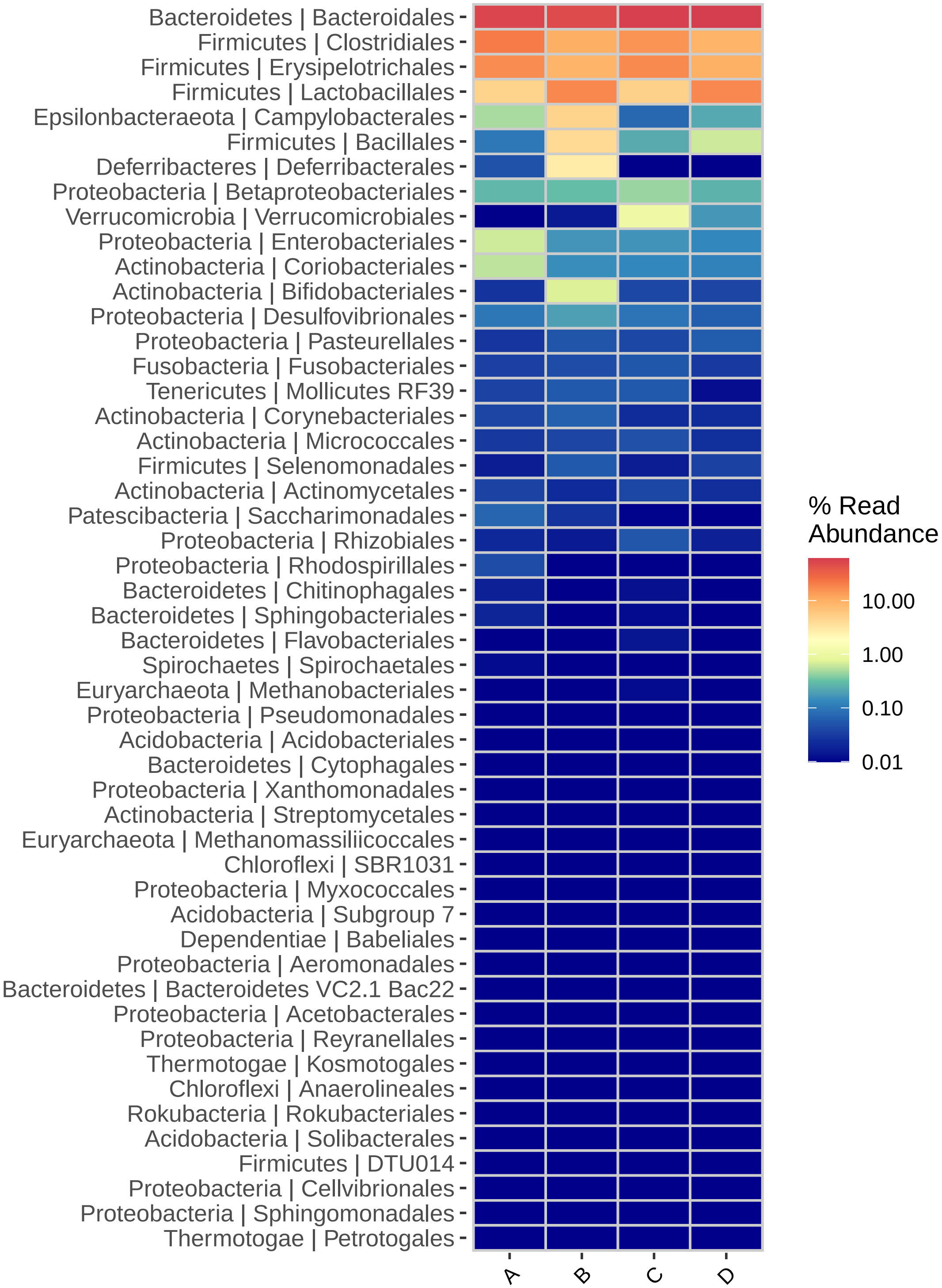
Bacteroidetes | Bacteroidales
Firmicutes | Clostridiales
Firmicutes | Erysipelotrichales
Firmicutes | Lactobacillales
Epsilonbacteraeota | Campylobacterales
Firmicutes | Bacillales
Deferribacteres | Deferribacterales
Proteobacteria | Betaproteobacteriales
Verrucomicrobia | Verrucomicrobiales
Proteobacteria | Enterobacteriales
Actinobacteria | Coriobacteriales
Actinobacteria | Bifidobacteriales
Proteobacteria | Desulfovibrionales
Proteobacteria | Pasteurellales
Fusobacteria | Fusobacteriales
Tenericutes | Mollicutes RF39
Actinobacteria | Corynebacteriales
Actinobacteria | Micrococcales
Firmicutes | Selenomonadales
Actinobacteria | Actinomycetales
Patescibacteria | Saccharimonadales
Proteobacteria | Rhizobiales
Proteobacteria | Rhodospirillales
Bacteroidetes | Chitinophagales
Bacteroidetes | Sphingobacteriales
Bacteroidetes | Flavobacteriales
Spirochaetes | Spirochaetales
Euryarchaeota | Methanobacteriales
Proteobacteria | Pseudomonadales
Acidobacteria | Acidobacteriales
Bacteroidetes | Cytophagales
Proteobacteria | Xanthomonadales
Actinobacteria | Streptomycetales
Euryarchaeota | Methanomassiliicoccales
Chloroflexi | SBR1031
Proteobacteria | Myxococcales
Acidobacteria | Subgroup 7
Dependentiae | Babeliales
Proteobacteria | Aeromonadales
Bacteroidetes | Bacteroidetes VC2.1 Bac22
Proteobacteria | Acetobacterales
Proteobacteria | Reyranellales
Thermotogae | Kosmotogales
Chloroflexi | Anaerolineales
Rokubacteria | Rokubacteriales
Acidobacteria | Solibacterales
Firmicutes | DTU014
Proteobacteria | Cellvibrionales
Proteobacteria | Sphingomonadales
Thermotogae | Petrotogales
A
B
C
D
% Read Abundance
10.00
1.00
0.10
0.01
