# Supplementary material for: Neuroprotective Effects of Oligosaccharides From Periplaneta Americana on Parkinson’s Disease Models In Vitro and In Vivo
Source: Front Pharmacol. 2022 Jul 18;13:936818. doi: 10.3389/fphar.2022.936818 (PMC9340460; doi:10.3389/fphar.2022.936818)
Supplement: Supplementary file 2 [file DataSheet1.zip › 16S rRNA/Images/Heatmap_phylum_top50_mean.pdf]

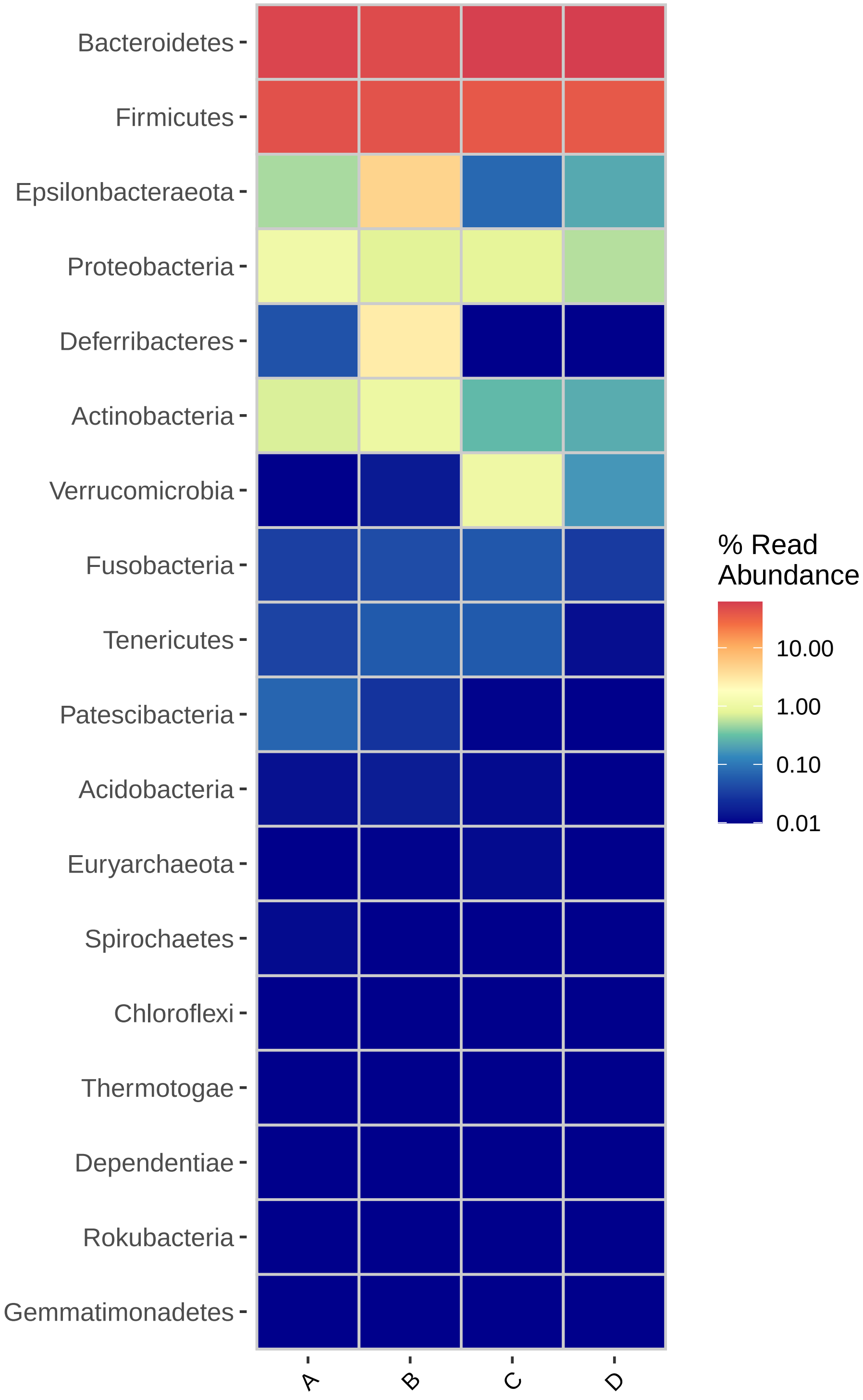
Bacteroidetes
Firmicutes
Epsilonbacteraeota
Proteobacteria
Deferribacteres
Actinobacteria
Verrucomicrobia
Fusobacteria
Tenericutes
Patescibacteria
Acidobacteria
Euryarchaeota
Spirochaetes
Chloroflexi
Thermotogae
Dependentiae
Rokubacteria
Gemmatimonadetes
A
B
C
D
% Read
Abundance
10.00
1.00
0.10
0.01
