# Supplementary material for: Neuroprotective Effects of Oligosaccharides From Periplaneta Americana on Parkinson’s Disease Models In Vitro and In Vivo
Source: Front Pharmacol. 2022 Jul 18;13:936818. doi: 10.3389/fphar.2022.936818 (PMC9340460; doi:10.3389/fphar.2022.936818)
Supplement: Supplementary file 2 [file DataSheet1.zip › 16S rRNA/Images/Heatmap_species_top50_all.pdf]

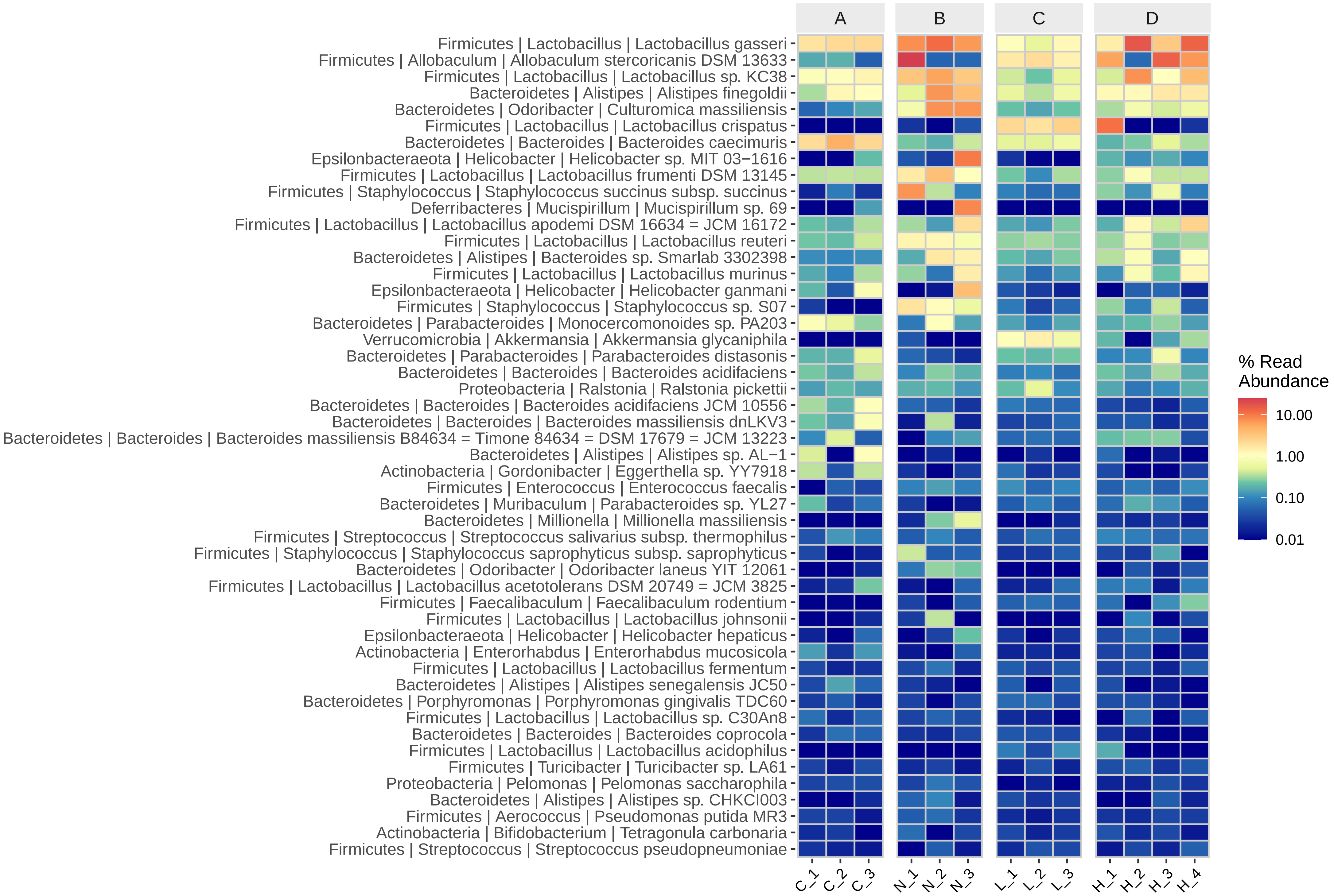
A
B
C
D
Firmicutes | Lactobacillus | Lactobacillus gasseri
Firmicutes | Allobaculum | Allobaculum stercoricanis DSM 13633
Firmicutes | Lactobacillus | Lactobacillus sp. KC38
Bacteroidetes | Alistipes | Alistipes finegoldii
Bacteroidetes | Odoribacter | Culturomica massiliensis
Firmicutes | Lactobacillus | Lactobacillus crispatus
Bacteroidetes | Bacteroides | Bacteroides caecimuris
Epsilonbacteraeota | Helicobacter | Helicobacter sp. MIT 03−1616
Firmicutes | Lactobacillus | Lactobacillus frumenti DSM 13145
Firmicutes | Staphylococcus | Staphylococcus succinus subsp. succinus
Deferribacteres | Mucispirillum | Mucispirillum sp. 69
Firmicutes | Lactobacillus | Lactobacillus apodemi DSM 16634 = JCM 16172
Firmicutes | Lactobacillus | Lactobacillus reuteri
Bacteroidetes | Alistipes | Bacteroides sp. Smarlab 3302398
Firmicutes | Lactobacillus | Lactobacillus murinus
Epsilonbacteraeota | Helicobacter | Helicobacter ganmani
Firmicutes | Staphylococcus | Staphylococcus sp. S07
Bacteroidetes | Parabacteroides | Monocercomonoides sp. PA203
Verrucomicrobia | Akkermansia | Akkermansia glycaniphila
Bacteroidetes | Parabacteroides | Parabacteroides distasonis
Bacteroidetes | Bacteroides | Bacteroides acidifaciens
Proteobacteria | Ralstonia | Ralstonia pickettii
Bacteroidetes | Bacteroides | Bacteroides acidifaciens JCM 10556
Bacteroidetes | Bacteroides | Bacteroides massiliensis dnLKV3
Bacteroidetes | Bacteroides | Bacteroides massiliensis B84634 = Timone 84634 = DSM 17679 = JCM 13223
Bacteroidetes | Alistipes | Alistipes sp. AL−1
Actinobacteria | Gordonibacter | Eggerthella sp. YY7918
Firmicutes | Enterococcus | Enterococcus faecalis
Bacteroidetes | Muribaculum | Parabacteroides sp. YL27
Bacteroidetes | Millionella | Millionella massiliensis
Firmicutes | Streptococcus | Streptococcus salivarius subsp. thermophilus
Firmicutes | Staphylococcus | Staphylococcus saprophyticus subsp. saprophyticus
Bacteroidetes | Odoribacter | Odoribacter laneus YIT 12061
Firmicutes | Lactobacillus | Lactobacillus acetotolerans DSM 20749 = JCM 3825
Firmicutes | Faecalibaculum | Faecalibaculum rodentium
Firmicutes | Lactobacillus | Lactobacillus johnsonii
Epsilonbacteraeota | Helicobacter | Helicobacter hepaticus
Actinobacteria | Enterorhabdus | Enterorhabdus mucosicola
Firmicutes | Lactobacillus | Lactobacillus fermentum
Bacteroidetes | Alistipes | Alistipes senegalensis JC50
Bacteroidetes | Porphyromonas | Porphyromonas gingivalis TDC60
Firmicutes | Lactobacillus | Lactobacillus sp. C30An8
Bacteroidetes | Bacteroides | Bacteroides coprocola
Firmicutes | Lactobacillus | Lactobacillus acidophilus
Firmicutes | Turicibacter | Turicibacter sp. LA61
Proteobacteria | Pelomonas | Pelomonas saccharophila
Bacteroidetes | Alistipes | Alistipes sp. CHKCI003
Firmicutes | Aerococcus | Pseudomonas putida MR3
Actinobacteria | Bifidobacterium | Tetragonula carbonaria
Firmicutes | Streptococcus | Streptococcus pseudopneumoniae
C_1
C_2
C_3
N_1
N_2
N_3
L_1
L_2
L_3
H_1
H_2
H_3
H_4
% Read
Abundance
10.00
1.00
0.10
0.01
