# Supplementary material for: Neuroprotective Effects of Oligosaccharides From Periplaneta Americana on Parkinson’s Disease Models In Vitro and In Vivo
Source: Front Pharmacol. 2022 Jul 18;13:936818. doi: 10.3389/fphar.2022.936818 (PMC9340460; doi:10.3389/fphar.2022.936818)
Supplement: Supplementary file 2 [file DataSheet1.zip › 16S rRNA/Images/MetagenomeSeq_A_vs_B.pdf]

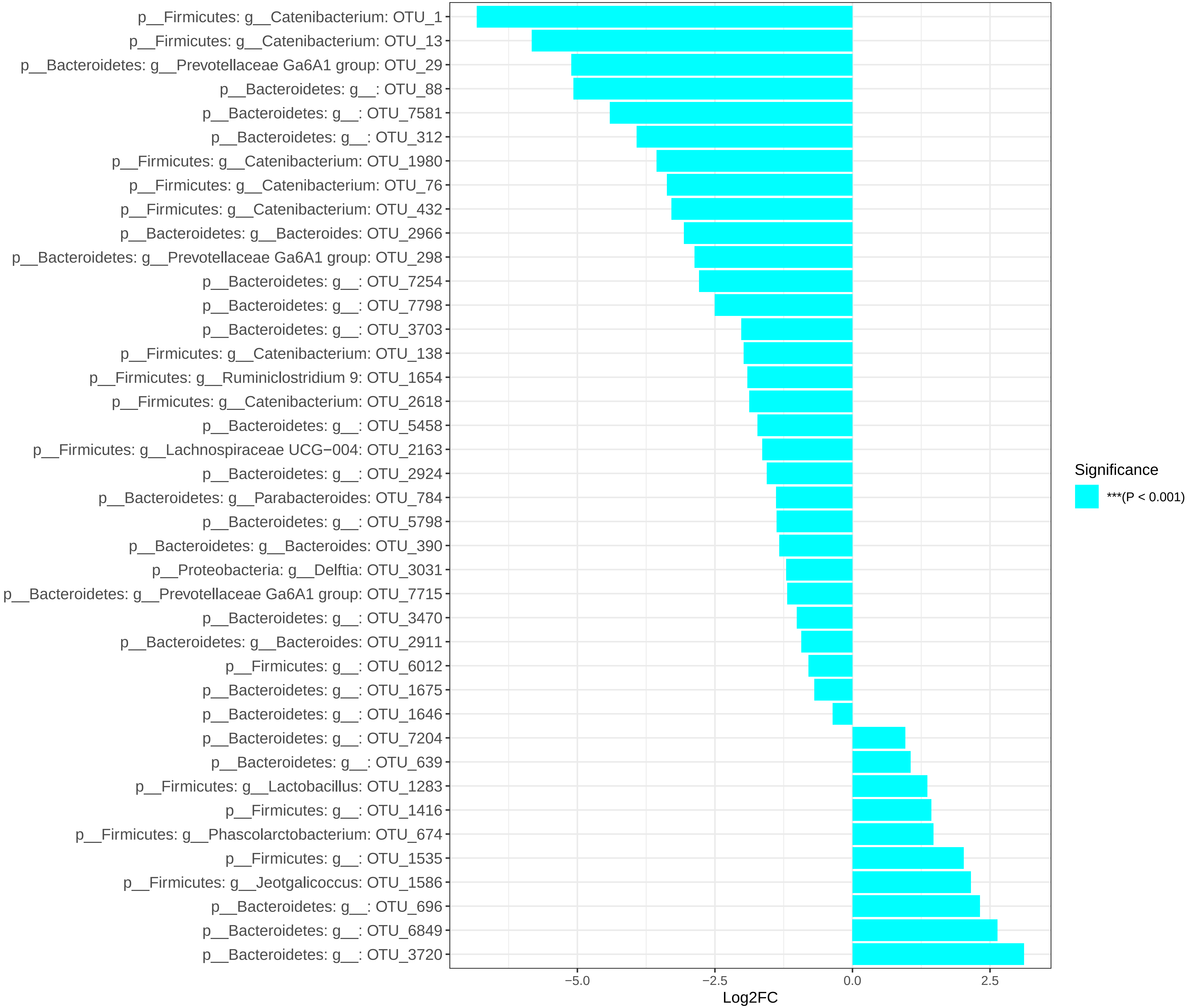

p__Firmicutes: g__Catenibacterium: OTU_1
p__Firmicutes: g__Catenibacterium: OTU_13
p__Bacteroidetes: g__Prevotellaceae Ga6A1 group: OTU_29
p__Bacteroidetes: g__: OTU_88
p__Bacteroidetes: g__: OTU_7581
p__Bacteroidetes: g__: OTU_312
p__Firmicutes: g__Catenibacterium: OTU_1980
p__Firmicutes: g__Catenibacterium: OTU_76
p__Firmicutes: g__Catenibacterium: OTU_432
p__Bacteroidetes: g__Bacteroides: OTU_2966
p__Bacteroidetes: g__Prevotellaceae Ga6A1 group: OTU_298
p__Bacteroidetes: g__: OTU_7254
p__Bacteroidetes: g__: OTU_7798
p__Bacteroidetes: g__: OTU_3703
p__Firmicutes: g__Catenibacterium: OTU_138
p__Firmicutes: g__Ruminiclostridium 9: OTU_1654
p__Firmicutes: g__Catenibacterium: OTU_2618
p__Bacteroidetes: g__: OTU_5458
p__Firmicutes: g__Lachnospiraceae UCG−004: OTU_2163
p__Bacteroidetes: g__: OTU_2924
p__Bacteroidetes: g__Parabacteroides: OTU_784
p__Bacteroidetes: g__: OTU_5798
p__Bacteroidetes: g__Bacteroides: OTU_390
p__Proteobacteria: g__Delftia: OTU_3031
p__Bacteroidetes: g__Prevotellaceae Ga6A1 group: OTU_7715
p__Bacteroidetes: g__: OTU_3470
p__Bacteroidetes: g__Bacteroides: OTU_2911
p__Firmicutes: g__: OTU_6012
p__Bacteroidetes: g__: OTU_1675
p__Bacteroidetes: g__: OTU_1646
p__Bacteroidetes: g__: OTU_7204
p__Bacteroidetes: g__: OTU_639
p__Firmicutes: g__Lactobacillus: OTU_1283
p__Firmicutes: g__: OTU_1416
p__Firmicutes: g__Phascolarctobacterium: OTU_674
p__Firmicutes: g__: OTU_1535
p__Firmicutes: g__Jeotgalicoccus: OTU_1586
p__Bacteroidetes: g__: OTU_696
p__Bacteroidetes: g__: OTU_6849
p__Bacteroidetes: g__: OTU_3720
−5.0
−2.5
0.0
2.5
Log2FC
Significance
***(P < 0.001)
