# Supplementary material for: Neuroprotective Effects of Oligosaccharides From Periplaneta Americana on Parkinson’s Disease Models In Vitro and In Vivo
Source: Front Pharmacol. 2022 Jul 18;13:936818. doi: 10.3389/fphar.2022.936818 (PMC9340460; doi:10.3389/fphar.2022.936818)
Supplement: Supplementary file 2 [file DataSheet1.zip › 16S rRNA/Images/MetagenomeSeq_A_vs_C.pdf]

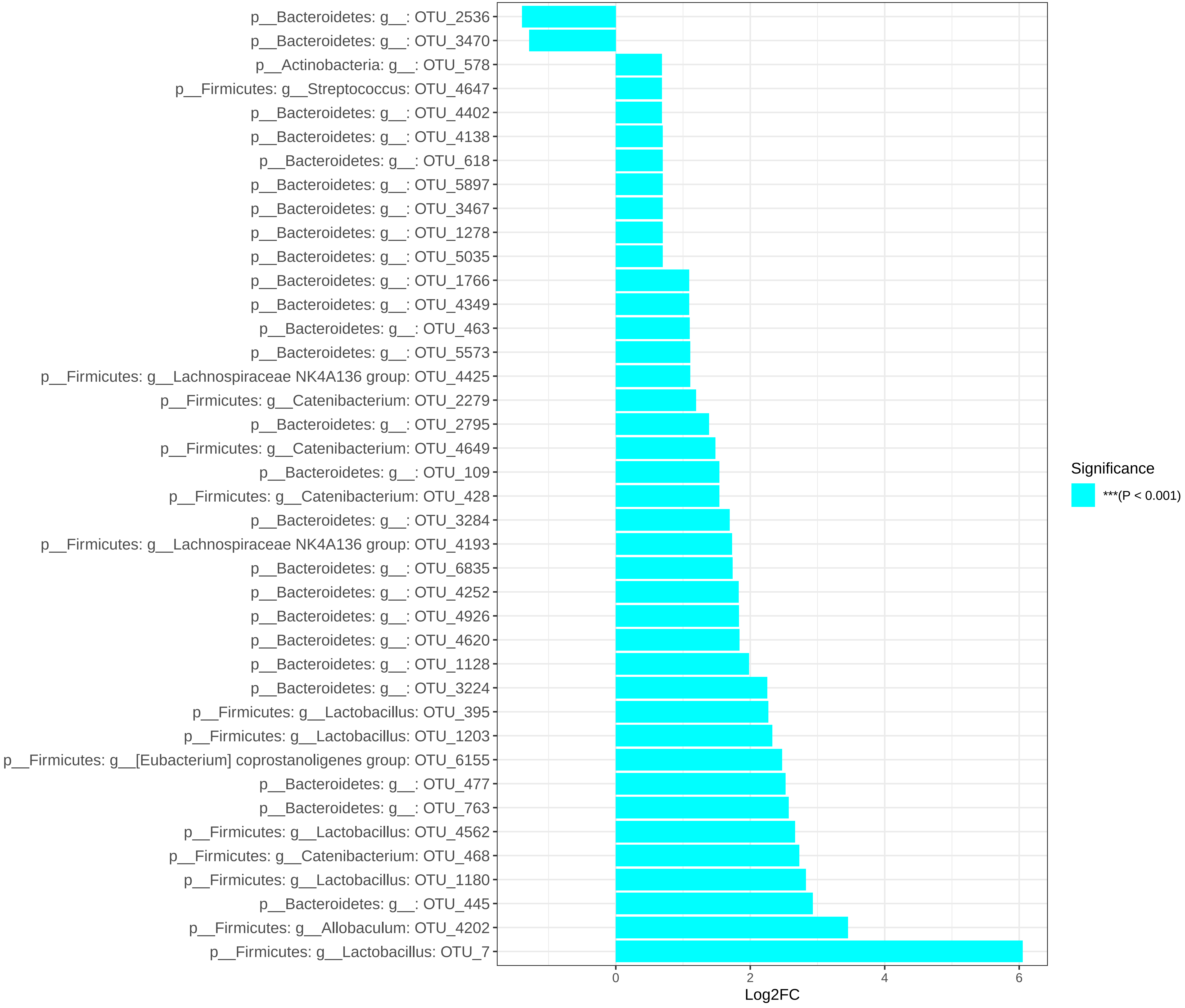
p__Bacteroidetes: g__: OTU_2536
p__Bacteroidetes: g__: OTU_3470
p__Actinobacteria: g__: OTU_578
p__Firmicutes: g__Streptococcus: OTU_4647
p__Bacteroidetes: g__: OTU_4402
p__Bacteroidetes: g__: OTU_4138
p__Bacteroidetes: g__: OTU_618
p__Bacteroidetes: g__: OTU_5897
p__Bacteroidetes: g__: OTU_3467
p__Bacteroidetes: g__: OTU_1278
p__Bacteroidetes: g__: OTU_5035
p__Bacteroidetes: g__: OTU_1766
p__Bacteroidetes: g__: OTU_4349
p__Bacteroidetes: g__: OTU_463
p__Bacteroidetes: g__: OTU_5573
p__Firmicutes: g__Lachnospiraceae NK4A136 group: OTU_4425
p__Firmicutes: g__Catenibacterium: OTU_2279
p__Bacteroidetes: g__: OTU_2795
p__Firmicutes: g__Catenibacterium: OTU_4649
p__Bacteroidetes: g__: OTU_109
p__Firmicutes: g__Catenibacterium: OTU_428
p__Bacteroidetes: g__: OTU_3284
p__Firmicutes: g__Lachnospiraceae NK4A136 group: OTU_4193
p__Bacteroidetes: g__: OTU_6835
p__Bacteroidetes: g__: OTU_4252
p__Bacteroidetes: g__: OTU_4926
p__Bacteroidetes: g__: OTU_4620
p__Bacteroidetes: g__: OTU_1128
p__Bacteroidetes: g__: OTU_3224
p__Firmicutes: g__Lactobacillus: OTU_395
p__Firmicutes: g__Lactobacillus: OTU_1203
p__Firmicutes: g__[Eubacterium] coprostanoligenes group: OTU_6155
p__Bacteroidetes: g__: OTU_477
p__Bacteroidetes: g__: OTU_763
p__Firmicutes: g__Lactobacillus: OTU_4562
p__Firmicutes: g__Catenibacterium: OTU_468
p__Firmicutes: g__Lactobacillus: OTU_1180
p__Bacteroidetes: g__: OTU_445
p__Firmicutes: g__Allobaculum: OTU_4202
p__Firmicutes: g__Lactobacillus: OTU_7
0
2
4
6
Log2FC
Significance
***(P < 0.001)
