# Supplementary material for: Neuroprotective Effects of Oligosaccharides From Periplaneta Americana on Parkinson’s Disease Models In Vitro and In Vivo
Source: Front Pharmacol. 2022 Jul 18;13:936818. doi: 10.3389/fphar.2022.936818 (PMC9340460; doi:10.3389/fphar.2022.936818)
Supplement: Supplementary file 2 [file DataSheet1.zip › 16S rRNA/Images/MetagenomeSeq_A_vs_D.pdf]

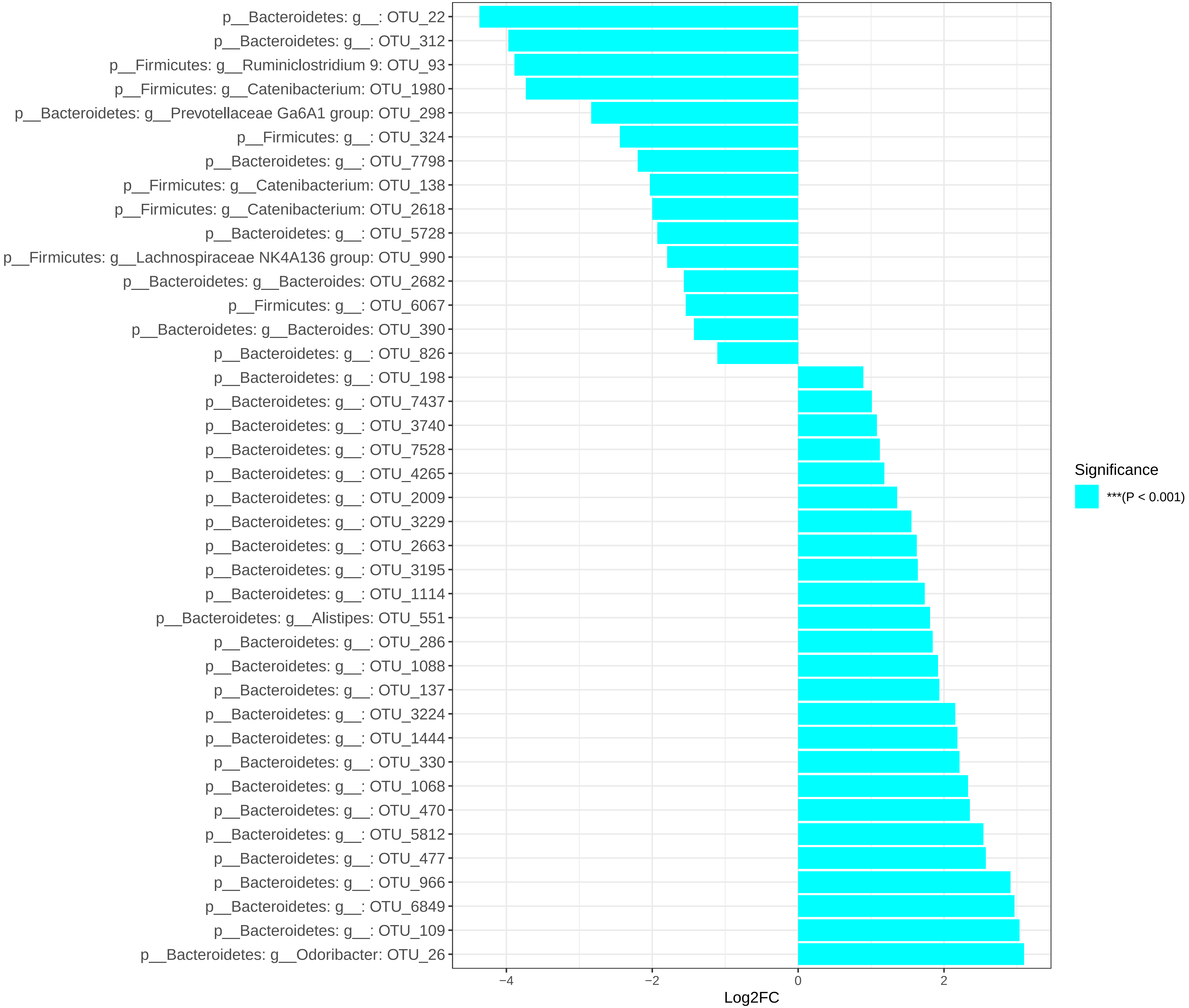
p__Bacteroidetes: g__: OTU_22
p__Bacteroidetes: g__: OTU_312
p__Firmicutes: g__Ruminiclostridium 9: OTU_93
p__Firmicutes: g__Catenibacterium: OTU_1980
p__Bacteroidetes: g__Prevotellaceae Ga6A1 group: OTU_298
p__Firmicutes: g__: OTU_324
p__Bacteroidetes: g__: OTU_7798
p__Firmicutes: g__Catenibacterium: OTU_138
p__Firmicutes: g__Catenibacterium: OTU_2618
p__Bacteroidetes: g__: OTU_5728
p__Firmicutes: g__Lachnospiraceae NK4A136 group: OTU_990
p__Bacteroidetes: g__Bacteroides: OTU_2682
p__Firmicutes: g__: OTU_6067
p__Bacteroidetes: g__Bacteroides: OTU_390
p__Bacteroidetes: g__: OTU_826
p__Bacteroidetes: g__: OTU_198
p__Bacteroidetes: g__: OTU_7437
p__Bacteroidetes: g__: OTU_3740
p__Bacteroidetes: g__: OTU_7528
p__Bacteroidetes: g__: OTU_4265
p__Bacteroidetes: g__: OTU_2009
p__Bacteroidetes: g__: OTU_3229
p__Bacteroidetes: g__: OTU_2663
p__Bacteroidetes: g__: OTU_3195
p__Bacteroidetes: g__: OTU_1114
p__Bacteroidetes: g__Alistipes: OTU_551
p__Bacteroidetes: g__: OTU_286
p__Bacteroidetes: g__: OTU_1088
p__Bacteroidetes: g__: OTU_137
p__Bacteroidetes: g__: OTU_3224
p__Bacteroidetes: g__: OTU_1444
p__Bacteroidetes: g__: OTU_330
p__Bacteroidetes: g__: OTU_1068
p__Bacteroidetes: g__: OTU_470
p__Bacteroidetes: g__: OTU_5812
p__Bacteroidetes: g__: OTU_477
p__Bacteroidetes: g__: OTU_966
p__Bacteroidetes: g__: OTU_6849
p__Bacteroidetes: g__: OTU_109
p__Bacteroidetes: g__Odoribacter: OTU_26
−4
−2
0
2
Log2FC
Significance
***(P < 0.001)
