# Supplementary material for: Neuroprotective Effects of Oligosaccharides From Periplaneta Americana on Parkinson’s Disease Models In Vitro and In Vivo
Source: Front Pharmacol. 2022 Jul 18;13:936818. doi: 10.3389/fphar.2022.936818 (PMC9340460; doi:10.3389/fphar.2022.936818)
Supplement: Supplementary file 2 [file DataSheet1.zip › 16S rRNA/Images/MetagenomeSeq_B_vs_D.pdf]

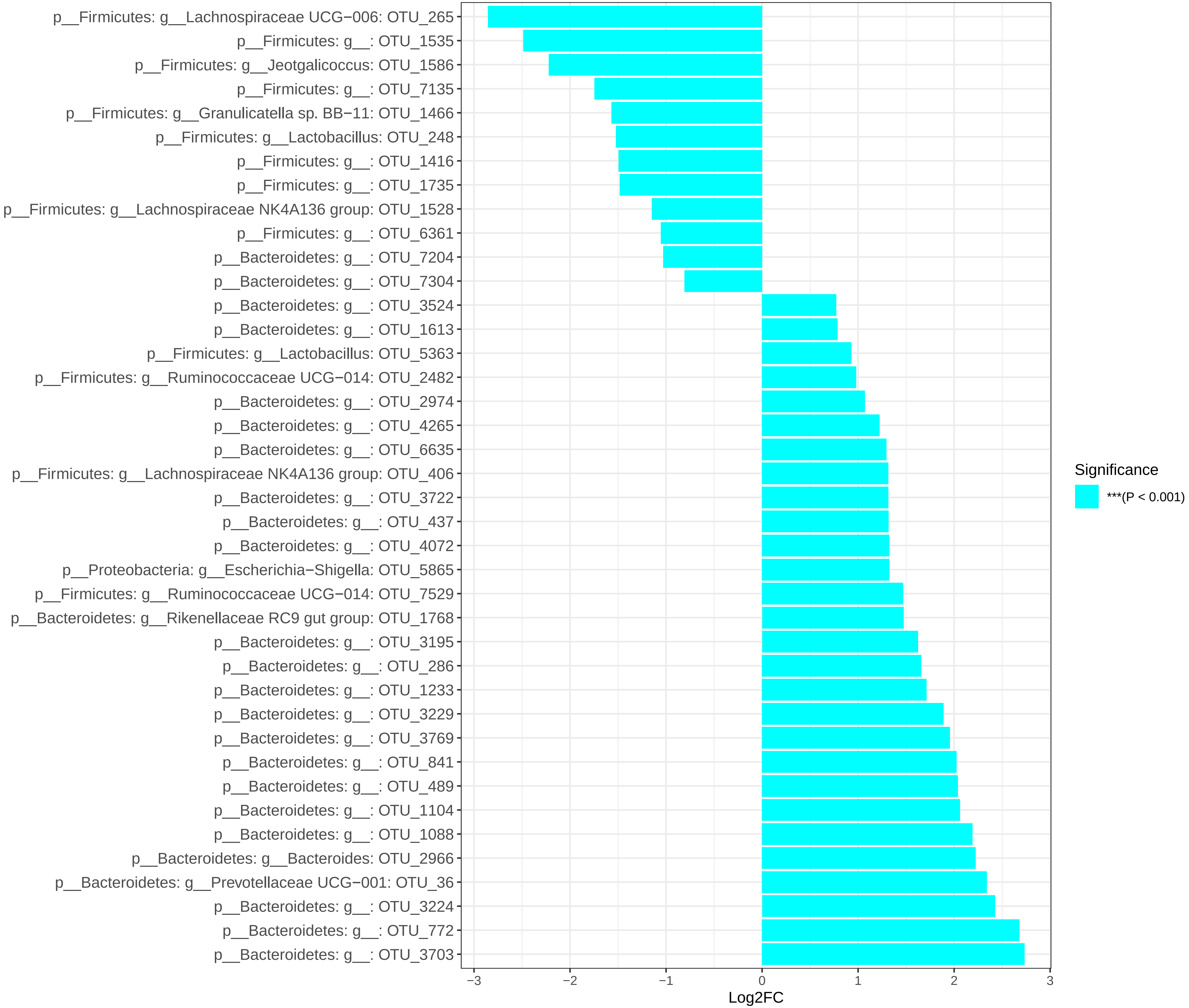
p__Firmicutes: g__Lachnospiraceae UCG−006: OTU_265
p__Firmicutes: g__: OTU_1535
p__Firmicutes: g__Jeotgalicoccus: OTU_1586
p__Firmicutes: g__: OTU_7135
p__Firmicutes: g__Granulicatella sp. BB−11: OTU_1466
p__Firmicutes: g__Lactobacillus: OTU_248
p__Firmicutes: g__: OTU_1416
p__Firmicutes: g__: OTU_1735
p__Firmicutes: g__Lachnospiraceae NK4A136 group: OTU_1528
p__Firmicutes: g__: OTU_6361
p__Bacteroidetes: g__: OTU_7204
p__Bacteroidetes: g__: OTU_7304
p__Bacteroidetes: g__: OTU_3524
p__Bacteroidetes: g__: OTU_1613
p__Firmicutes: g__Lactobacillus: OTU_5363
p__Firmicutes: g__Ruminococcaceae UCG−014: OTU_2482
p__Bacteroidetes: g__: OTU_2974
p__Bacteroidetes: g__: OTU_4265
p__Bacteroidetes: g__: OTU_6635
p__Firmicutes: g__Lachnospiraceae NK4A136 group: OTU_406
p__Bacteroidetes: g__: OTU_3722
p__Bacteroidetes: g__: OTU_437
p__Bacteroidetes: g__: OTU_4072
p__Proteobacteria: g__Escherichia−Shigella: OTU_5865
p__Firmicutes: g__Ruminococcaceae UCG−014: OTU_7529
p__Bacteroidetes: g__Rikenellaceae RC9 gut group: OTU_1768
p__Bacteroidetes: g__: OTU_3195
p__Bacteroidetes: g__: OTU_286
p__Bacteroidetes: g__: OTU_1233
p__Bacteroidetes: g__: OTU_3229
p__Bacteroidetes: g__: OTU_3769
p__Bacteroidetes: g__: OTU_841
p__Bacteroidetes: g__: OTU_489
p__Bacteroidetes: g__: OTU_1104
p__Bacteroidetes: g__: OTU_1088
p__Bacteroidetes: g__Bacteroides: OTU_2966
p__Bacteroidetes: g__Prevotellaceae UCG−001: OTU_36
p__Bacteroidetes: g__: OTU_3224
p__Bacteroidetes: g__: OTU_772
p__Bacteroidetes: g__: OTU_3703
−3
−2
−1
0
1
2
3
Log2FC
Significance
***(P < 0.001)
