# Supplementary material for: Neuroprotective Effects of Oligosaccharides From Periplaneta Americana on Parkinson’s Disease Models In Vitro and In Vivo
Source: Front Pharmacol. 2022 Jul 18;13:936818. doi: 10.3389/fphar.2022.936818 (PMC9340460; doi:10.3389/fphar.2022.936818)
Supplement: Supplementary file 2 [file DataSheet1.zip › 16S rRNA/Images/MetagenomeSeq_C_vs_B.pdf]

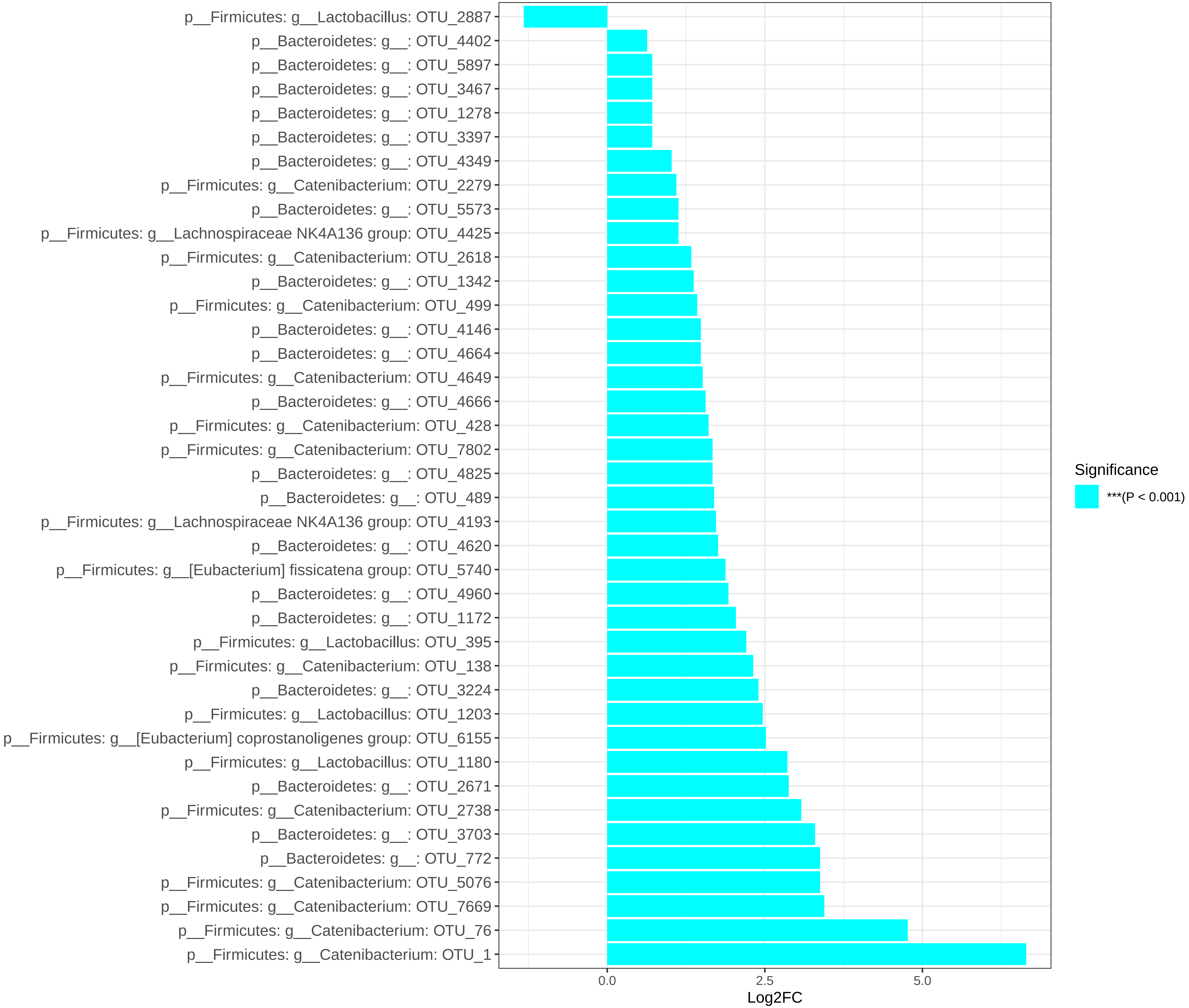
p__Firmicutes: g__Lactobacillus: OTU_2887
p__Bacteroidetes: g__: OTU_4402
p__Bacteroidetes: g__: OTU_5897
p__Bacteroidetes: g__: OTU_3467
p__Bacteroidetes: g__: OTU_1278
p__Bacteroidetes: g__: OTU_3397
p__Bacteroidetes: g__: OTU_4349
p__Firmicutes: g__Catenibacterium: OTU_2279
p__Bacteroidetes: g__: OTU_5573
p__Firmicutes: g__Lachnospiraceae NK4A136 group: OTU_4425
p__Firmicutes: g__Catenibacterium: OTU_2618
p__Bacteroidetes: g__: OTU_1342
p__Firmicutes: g__Catenibacterium: OTU_499
p__Bacteroidetes: g__: OTU_4146
p__Bacteroidetes: g__: OTU_4664
p__Firmicutes: g__Catenibacterium: OTU_4649
p__Bacteroidetes: g__: OTU_4666
p__Firmicutes: g__Catenibacterium: OTU_428
p__Firmicutes: g__Catenibacterium: OTU_7802
p__Bacteroidetes: g__: OTU_4825
p__Bacteroidetes: g__: OTU_489
p__Firmicutes: g__Lachnospiraceae NK4A136 group: OTU_4193
p__Bacteroidetes: g__: OTU_4620
p__Firmicutes: g__[Eubacterium] fissicatena group: OTU_5740
p__Bacteroidetes: g__: OTU_4960
p__Bacteroidetes: g__: OTU_1172
p__Firmicutes: g__Lactobacillus: OTU_395
p__Firmicutes: g__Catenibacterium: OTU_138
p__Bacteroidetes: g__: OTU_3224
p__Firmicutes: g__Lactobacillus: OTU_1203
p__Firmicutes: g__[Eubacterium] coprostanoligenes group: OTU_6155
p__Firmicutes: g__Lactobacillus: OTU_1180
p__Bacteroidetes: g__: OTU_2671
p__Firmicutes: g__Catenibacterium: OTU_2738
p__Bacteroidetes: g__: OTU_3703
p__Bacteroidetes: g__: OTU_772
p__Firmicutes: g__Catenibacterium: OTU_5076
p__Firmicutes: g__Catenibacterium: OTU_7669
p__Firmicutes: g__Catenibacterium: OTU_76
p__Firmicutes: g__Catenibacterium: OTU_1
0.0
2.5
5.0
Log2FC
Significance
***(P < 0.001)
