# Supplementary material for: Neuroprotective Effects of Oligosaccharides From Periplaneta Americana on Parkinson’s Disease Models In Vitro and In Vivo
Source: Front Pharmacol. 2022 Jul 18;13:936818. doi: 10.3389/fphar.2022.936818 (PMC9340460; doi:10.3389/fphar.2022.936818)
Supplement: Supplementary file 2 [file DataSheet1.zip › 16S rRNA/Images/MetagenomeSeq_C_vs_D.pdf]

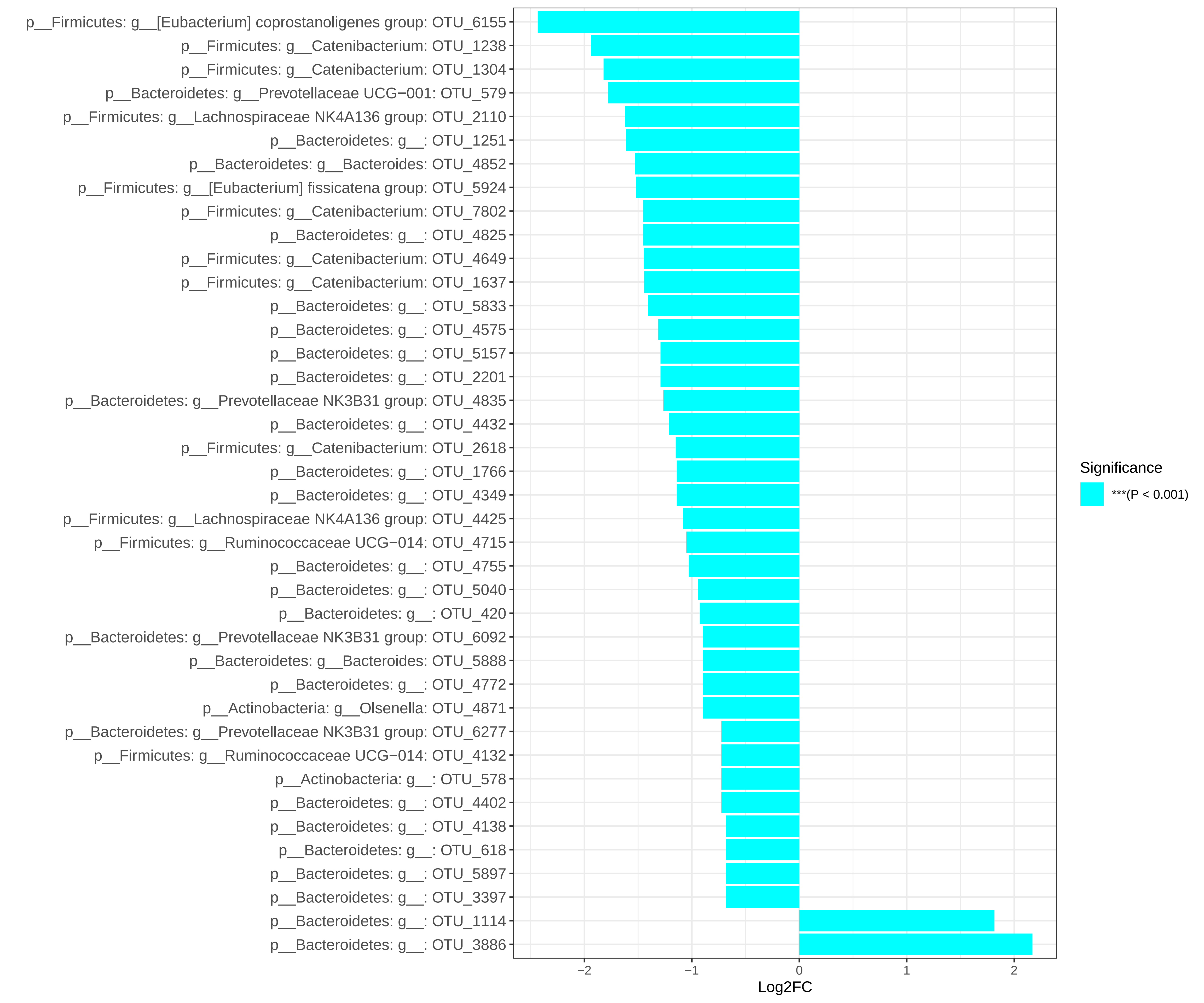

p__Firmicutes: g__[Eubacterium] coprostanoligenes group: OTU_6155
p__Firmicutes: g__Catenibacterium: OTU_1238
p__Firmicutes: g__Catenibacterium: OTU_1304
p__Bacteroidetes: g__Prevotellaceae UCG−001: OTU_579
p__Firmicutes: g__Lachnospiraceae NK4A136 group: OTU_2110
p__Bacteroidetes: g__: OTU_1251
p__Bacteroidetes: g__Bacteroides: OTU_4852
p__Firmicutes: g__[Eubacterium] fissicatena group: OTU_5924
p__Firmicutes: g__Catenibacterium: OTU_7802
p__Bacteroidetes: g__: OTU_4825
p__Firmicutes: g__Catenibacterium: OTU_4649
p__Firmicutes: g__Catenibacterium: OTU_1637
p__Bacteroidetes: g__: OTU_5833
p__Bacteroidetes: g__: OTU_4575
p__Bacteroidetes: g__: OTU_5157
p__Bacteroidetes: g__: OTU_2201
p__Bacteroidetes: g__Prevotellaceae NK3B31 group: OTU_4835
p__Bacteroidetes: g__: OTU_4432
p__Firmicutes: g__Catenibacterium: OTU_2618
p__Bacteroidetes: g__: OTU_1766
p__Bacteroidetes: g__: OTU_4349
p__Firmicutes: g__Lachnospiraceae NK4A136 group: OTU_4425
p__Firmicutes: g__Ruminococcaceae UCG−014: OTU_4715
p__Bacteroidetes: g__: OTU_4755
p__Bacteroidetes: g__: OTU_5040
p__Bacteroidetes: g__: OTU_420
p__Bacteroidetes: g__Prevotellaceae NK3B31 group: OTU_6092
p__Bacteroidetes: g__Bacteroides: OTU_5888
p__Bacteroidetes: g__: OTU_4772
p__Actinobacteria: g__Olsenella: OTU_4871
p__Bacteroidetes: g__Prevotellaceae NK3B31 group: OTU_6277
p__Firmicutes: g__Ruminococcaceae UCG−014: OTU_4132
p__Actinobacteria: g__: OTU_578
p__Bacteroidetes: g__: OTU_4402
p__Bacteroidetes: g__: OTU_4138
p__Bacteroidetes: g__: OTU_618
p__Bacteroidetes: g__: OTU_5897
p__Bacteroidetes: g__: OTU_3397
p__Bacteroidetes: g__: OTU_1114
p__Bacteroidetes: g__: OTU_3886
−2
−1
0
1
2
Log2FC
Significance
***(P < 0.001)
