# Supplementary material for: Neuroprotective Effects of Oligosaccharides From Periplaneta Americana on Parkinson’s Disease Models In Vitro and In Vivo
Source: Front Pharmacol. 2022 Jul 18;13:936818. doi: 10.3389/fphar.2022.936818 (PMC9340460; doi:10.3389/fphar.2022.936818)
Supplement: Supplementary file 2 [file DataSheet1.zip › 16S rRNA/Images/NMDS_Weighted_UniFrac.pdf]

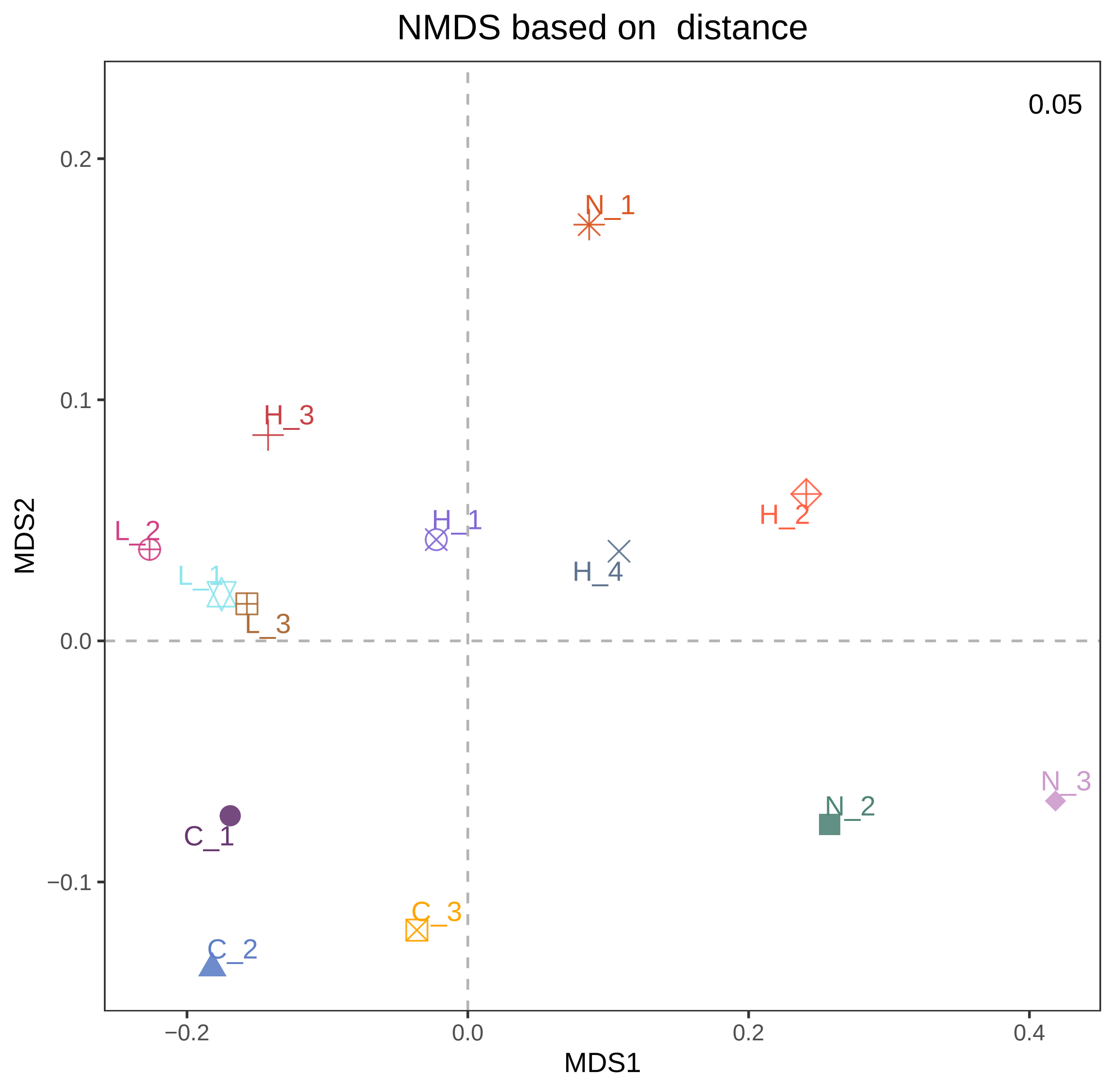
NMDS based on distance
0.05
N_1
H_3
H_2
L_2
H_1
H_4
L_1
L_3
N_3
N_2
C_1
C_3
C_2
0.2
0.1
0.0
−0.1
−0.2
0.0
0.2
0.4
MDS1
MDS2
