# Supplementary material for: Neuroprotective Effects of Oligosaccharides From Periplaneta Americana on Parkinson’s Disease Models In Vitro and In Vivo
Source: Front Pharmacol. 2022 Jul 18;13:936818. doi: 10.3389/fphar.2022.936818 (PMC9340460; doi:10.3389/fphar.2022.936818)
Supplement: Supplementary file 2 [file DataSheet1.zip › 16S rRNA/Images/PCA.pdf]

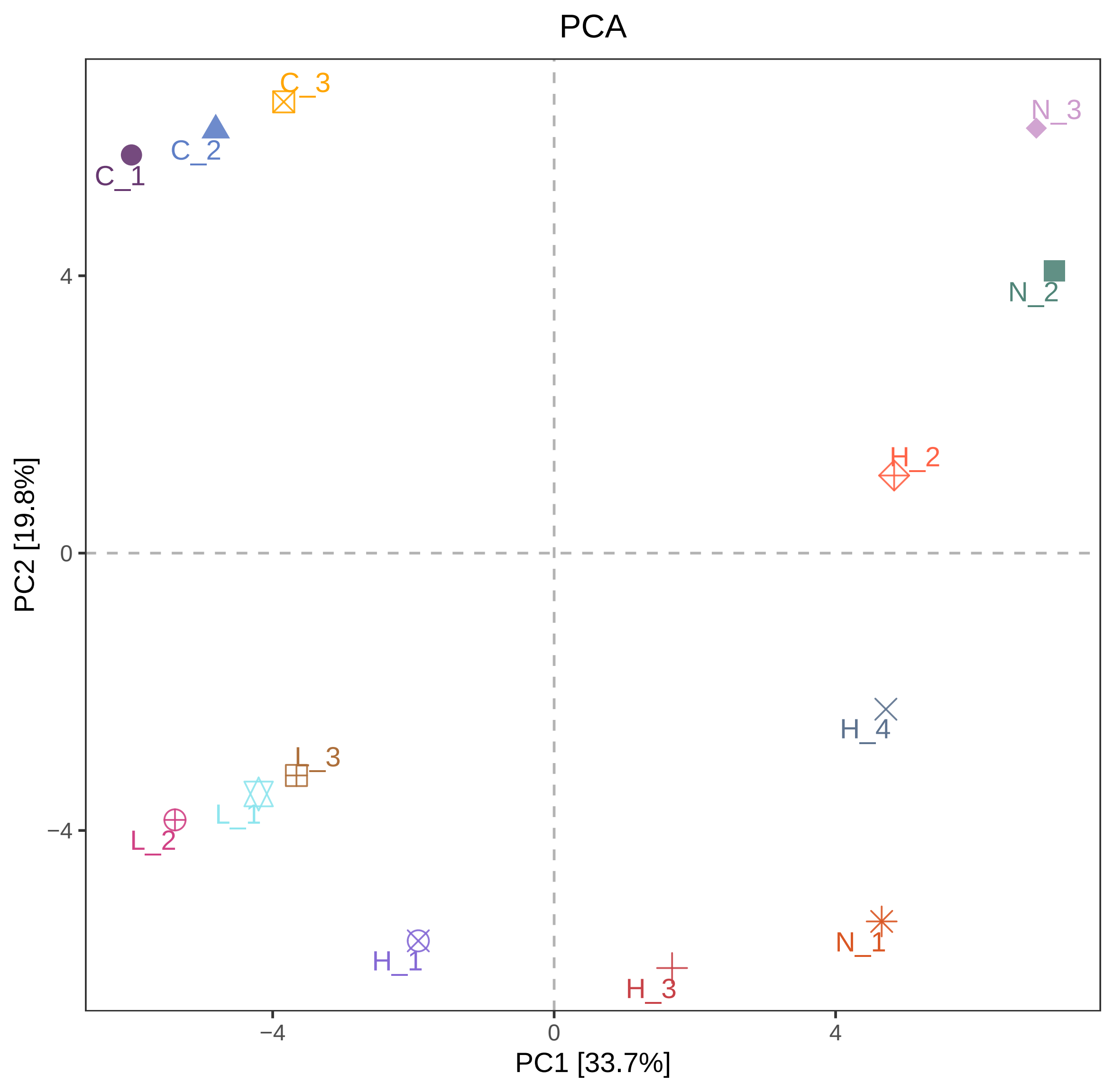
PCA
PC1 [33.7%]
PC2 [19.8%]
−4
0
4
4
0
−4
C_1
C_2
C_3
N_3
N_2
H_2
H_4
L_3
L_1
L_2
H_1
H_3
N_1
