# Supplementary material for: Neuroprotective Effects of Oligosaccharides From Periplaneta Americana on Parkinson’s Disease Models In Vitro and In Vivo
Source: Front Pharmacol. 2022 Jul 18;13:936818. doi: 10.3389/fphar.2022.936818 (PMC9340460; doi:10.3389/fphar.2022.936818)
Supplement: Supplementary file 2 [file DataSheet1.zip › 16S rRNA/Images/PCoA_Unweighted_UniFrac.pdf]

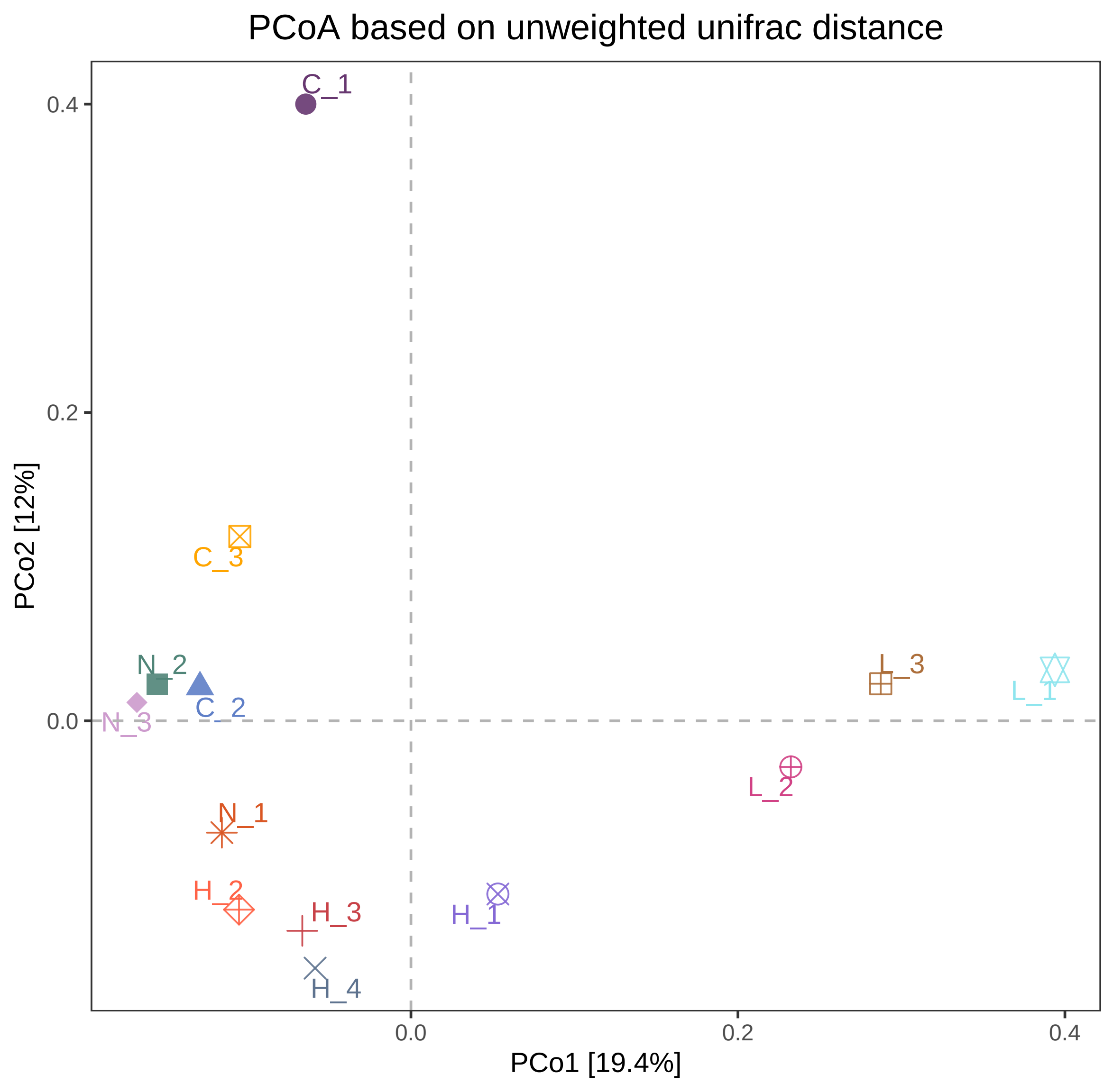
PCoA based on unweighted unifrac distance
PCo2 [12%]
PCo1 [19.4%]
0.4
0.2
0.0
0.0
0.2
0.4
C_1
C_3
N_2
C_2
N_3
L_3
L_1
L_2
N_1
H_2
H_3
H_1
H_4
