# Supplementary material for: Neuroprotective Effects of Oligosaccharides From Periplaneta Americana on Parkinson’s Disease Models In Vitro and In Vivo
Source: Front Pharmacol. 2022 Jul 18;13:936818. doi: 10.3389/fphar.2022.936818 (PMC9340460; doi:10.3389/fphar.2022.936818)
Supplement: Supplementary file 2 [file DataSheet1.zip › 16S rRNA/Images/PCoA_Weighted_UniFrac_Group.pdf]

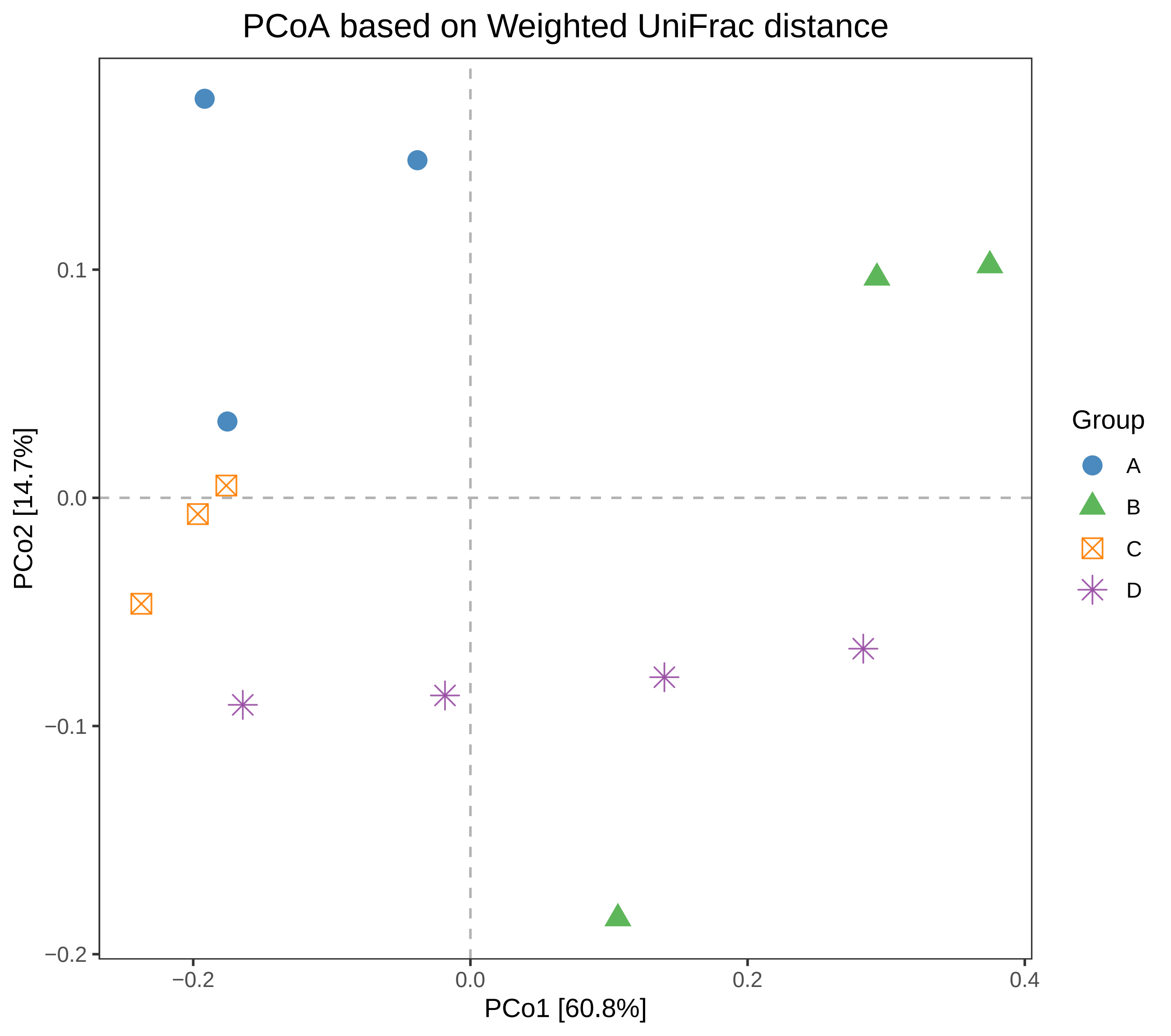

PCoA based on Weighted UniFrac distance
0.1
0.0
−0.1
−0.2
−0.2
0.0
0.2
0.4
PCo1 [60.8%]
PCo2 [14.7%]
Group
A
B
C
D
