# Supplementary material for: Neuroprotective Effects of Oligosaccharides From Periplaneta Americana on Parkinson’s Disease Models In Vitro and In Vivo
Source: Front Pharmacol. 2022 Jul 18;13:936818. doi: 10.3389/fphar.2022.936818 (PMC9340460; doi:10.3389/fphar.2022.936818)
Supplement: Supplementary file 2 [file DataSheet1.zip › 16S rRNA/Images/Phylogenetic_tree_top50_OTU.pdf]

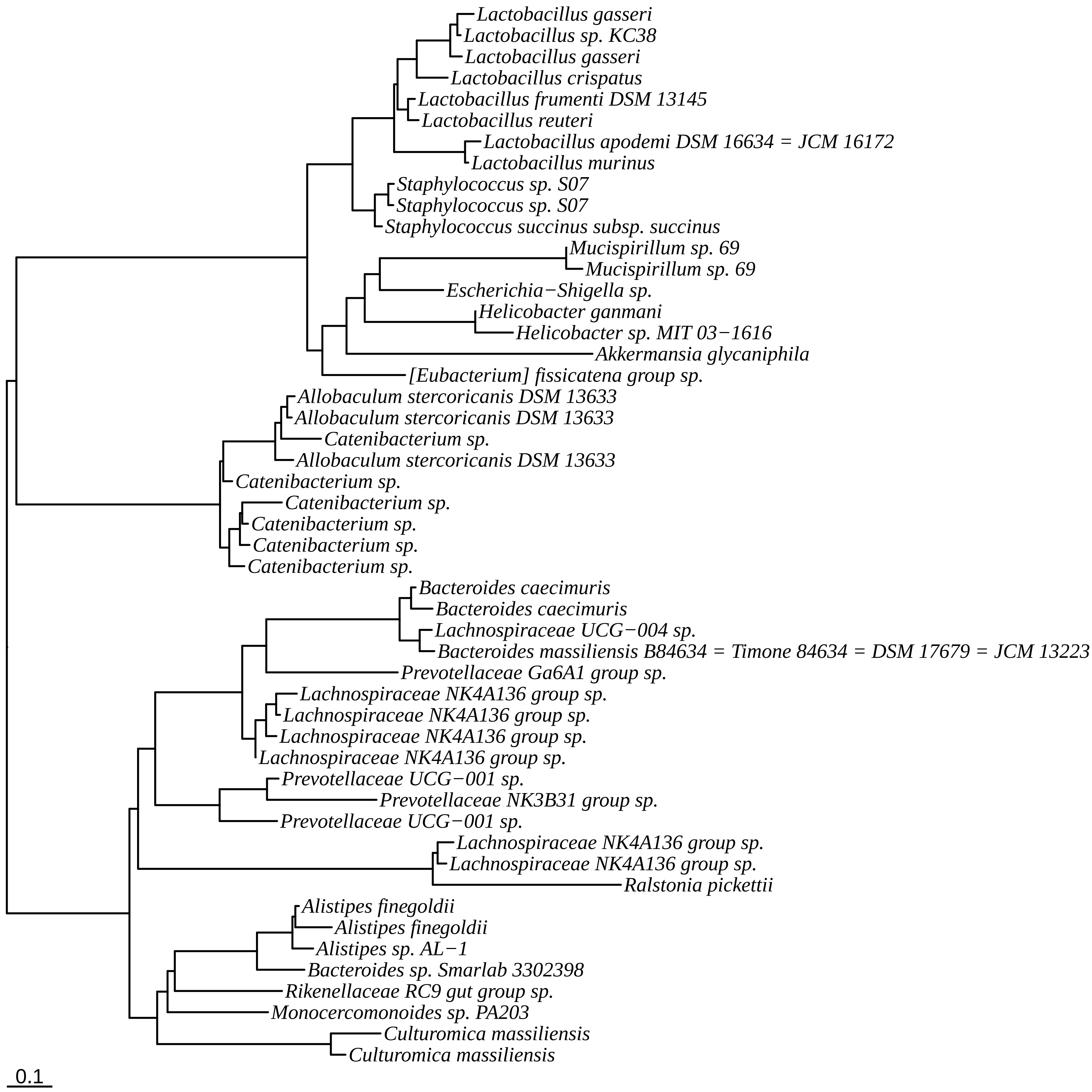
Lactobacillus gasseri
Lactobacillus sp. KC38
Lactobacillus gasseri
Lactobacillus crispatus
Lactobacillus frumenti DSM 13145
Lactobacillus reuteri
Lactobacillus apodemi DSM 16634 = JCM 16172
Lactobacillus murinus
Staphylococcus sp. S07
Staphylococcus sp. S07
Staphylococcus succinus subsp. succinus
Mucispirillum sp. 69
Mucispirillum sp. 69
Escherichia−Shigella sp.
Helicobacter ganmani
Helicobacter sp. MIT 03−1616
Akkermansia glycaniphila
[Eubacterium] fissicatena group sp.
Allobaculum stercoricanis DSM 13633
Allobaculum stercoricanis DSM 13633
Catenibacterium sp.
Allobaculum stercoricanis DSM 13633
Catenibacterium sp.
Catenibacterium sp.
Catenibacterium sp.
Catenibacterium sp.
Catenibacterium sp.
Bacteroides caecimuris
Bacteroides caecimuris
Lachnospiraceae UCG−004 sp.
Bacteroides massiliensis B84634 = Timone 84634 = DSM 17679 = JCM 13223
Prevotellaceae Ga6A1 group sp.
Lachnospiraceae NK4A136 group sp.
Lachnospiraceae NK4A136 group sp.
Lachnospiraceae NK4A136 group sp.
Lachnospiraceae NK4A136 group sp.
Prevotellaceae UCG−001 sp.
Prevotellaceae NK3B31 group sp.
Prevotellaceae UCG−001 sp.
Lachnospiraceae NK4A136 group sp.
Lachnospiraceae NK4A136 group sp.
Ralstonia pickettii
Alistipes finegoldii
Alistipes finegoldii
Alistipes sp. AL−1
Bacteroides sp. Smarlab 3302398
Rikenellaceae RC9 gut group sp.
Monocercomonoides sp. PA203
Culturomica massiliensis
Culturomica massiliensis
0.1
