# Supplementary material for: Neuroprotective Effects of Oligosaccharides From Periplaneta Americana on Parkinson’s Disease Models In Vitro and In Vivo
Source: Front Pharmacol. 2022 Jul 18;13:936818. doi: 10.3389/fphar.2022.936818 (PMC9340460; doi:10.3389/fphar.2022.936818)
Supplement: Supplementary file 2 [file DataSheet1.zip › 16S rRNA/Images/RandomForest_Gini.pdf]

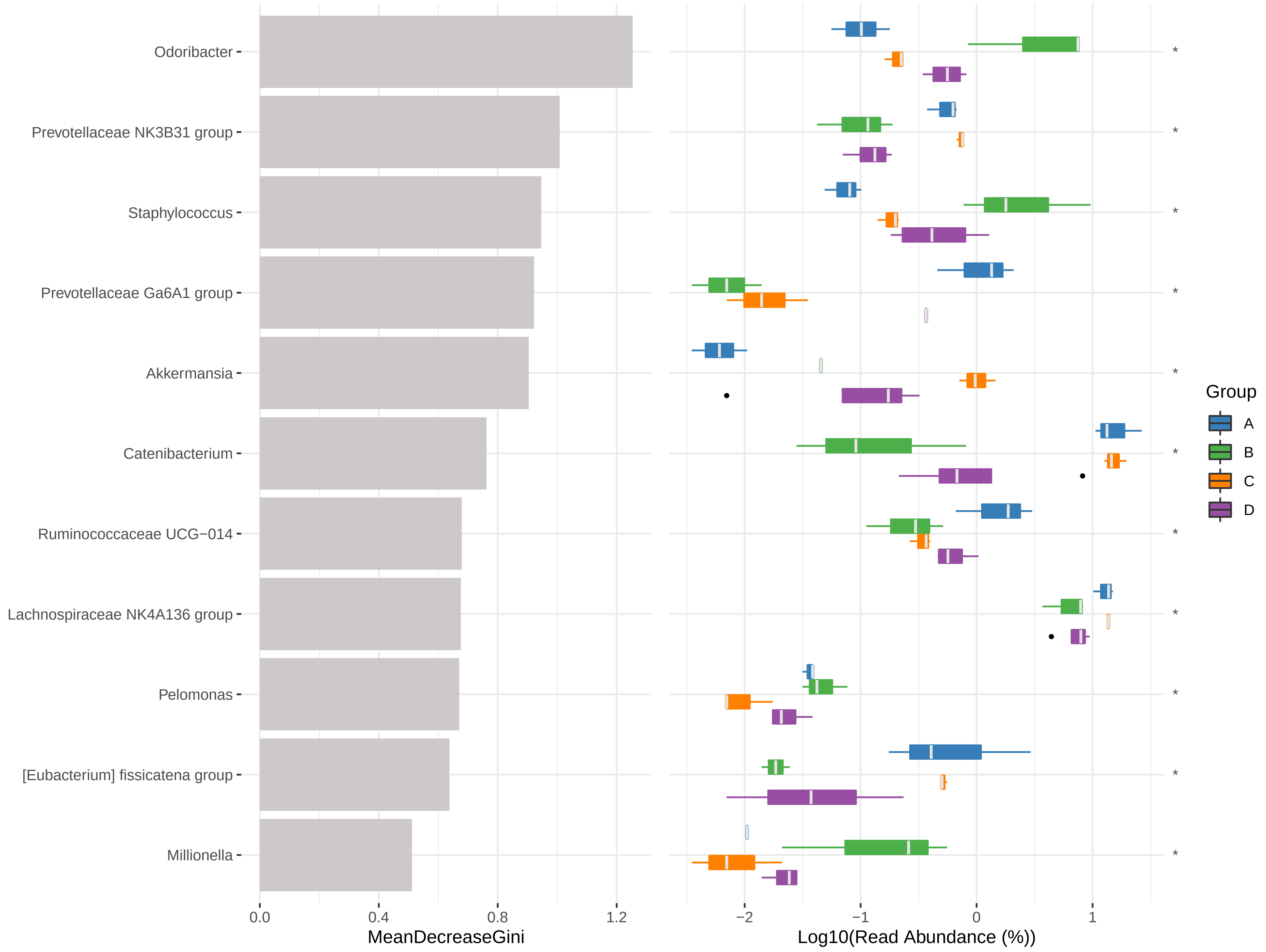

Odoribacter
Prevotellaceae NK3B31 group
Staphylococcus
Prevotellaceae Ga6A1 group
Akkermansia
Catenibacterium
Ruminococcaceae UCG−014
Lachnospiraceae NK4A136 group
Pelomonas
[Eubacterium] fissicatena group
Millionella
0.0
0.4
0.8
1.2
MeanDecreaseGini
−2
−1
0
1
Log10(Read Abundance (%))
*
Group
A
B
C
D
